# Bridged Pyrroloindole‐CAYC‐Gold Complexes: Harnessing Weak Secondary Intramolecular Au···H−C(*sp^3^*) Interactions in Gold(I) Catalysis

**DOI:** 10.1002/anie.202526091

**Published:** 2026-03-15

**Authors:** Sourav Sekhar Bera, Anurag Kumar, Pengcheng Gao, Sanghamitra Das, Roger Lalancette, Roman Szostak, Michal Szostak

**Affiliations:** ^1^ Department of Chemistry Rutgers University Newark New Jersey USA; ^2^ Department of Chemistry The Scripps Research Institute La Jolla California USA; ^3^ Department of Chemistry IIT Kharagpur Kharagpur West Bengal India; ^4^ Department of Chemistry Wroclaw University Wroclaw Poland

**Keywords:** σ‐donation, Au(I)‐catalysis, Au···H−C(*sp^3^*) interaction, bridged cyclic (amino)ylide carbene, ligand design

## Abstract

Gold(I) catalysis has been established as a tremendously powerful tool in organic synthesis and catalysis. Herein, we introduce a new class of electronically‐ and sterically‐hindered N‐heterocyclic carbene ligands, termed as bridged cyclic amino(ylide)carbene (^*b*^CAYC), containing 9H‐pyrrolo[1,2‐a]indoles as the carbene core and diaryl sulfonium as the ylide partner. Highly electron‐rich ^*b*^CAYC ligands are suitable for synthesizing the corresponding gold(I) complexes, which exhibit a weak intramolecular Au···H−C(sp^3^) interaction as a secondary interaction where the gold d‐orbitals act as electron donors and the C–H σ* orbitals act as acceptors. Properties and parameters of Au···H−C(sp^3^) interactions were investigated through X‐ray, NMR, DFT, AIM, and NBO studies. This study reports a remarkably short Au···H−C(sp^3^) distance of 2.26 Å, as determined through X‐ray crystallographic analysis. DFT and TEP data analysis revealed that ^*b*^CAYCs are among the most σ‐donating carbene ligands to date, than other widely used NHC ligands. The steric impact of ^*b*^CAYCs has shown excellent potential for steric shielding, with %V_bur_ reaching up to 42.6%. These unique properties were well‐reflected through the room‐temperature hydroamination and one‐pot C–N and C–C bond‐forming reactions.

## Graphical abstract

We present a new class of electronically and sterically hindered N‐heterocyclic carbenes, referred to as bridged cyclic (amino)ylide carbene (*^b^* CAYC), where 9H‐pyrrolo[1,2‐a]indole acted as carbene core and diaryl sulfonium as ylide partner. Corresponding *^b^* CAYC‐Au(I) exhibits a weak secondary intramolecular Au···H−C(sp^3^) interaction as a secondary interaction. DFT and TEP analysis indicate *^b^* CAYCs are among the strongest σ‐donating NHCs to date.

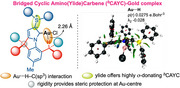

## 1 Introduction

**FIGURE 1:**
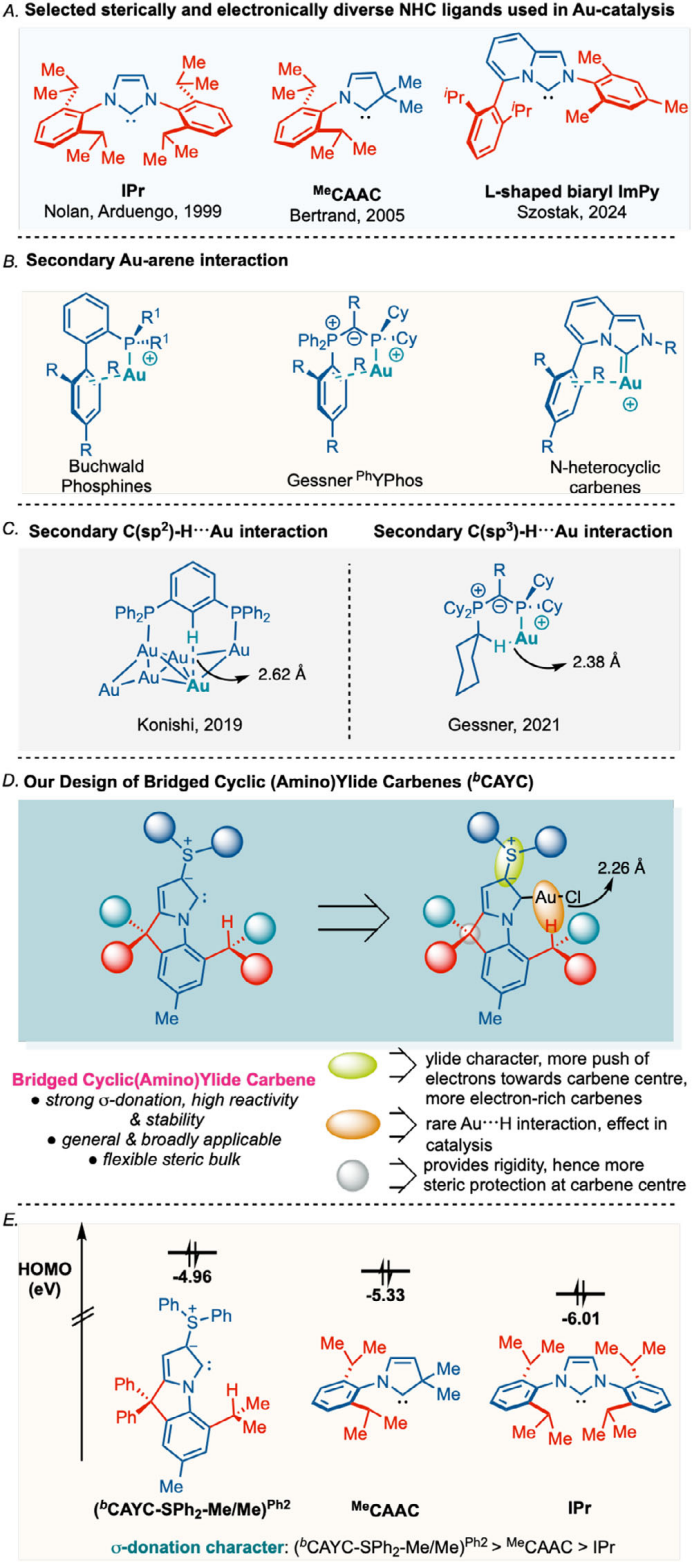
(A) Examples of gold‐arene interactions. (B) Examples of secondary Au···H─C interactions. (C) Selected sterically‐ and electronically‐diverse NHC ligands in Au‐catalysis. (D) Design of bridged CAYC ligands and gold complexes. (E) HOMO (σ‐donating orbitals) of *^b^* CAYC ligand and NHCs.

In the last twenty years, *N*‐heterocyclic carbenes (NHC) have been established as one of the most attractive classes of ligands in transition‐metal‐catalysis [1, 2, 3, 4, 5, 6, 7, 8, 9, 10, 11, 12, 13, 14, 15, 16, 17]. Among the several NHC‐metal catalysts, NHC‐gold complexes have been recognized as some of the most powerful catalysts in a variety of reactions, including alkene activation, enyne cycloisomerization, C–H activations, cross‐coupling reactions, hydroamination, and polymerization [18, 19, 20, 21, 22, 23, 24, 25, 26, 27]. Various electronically‐ and sterically‐diverse classes of NHC ligands, such as imidazolium carbenes (IPr, IMes) [28], cyclic (alkyl)(amino) carbenes (CAACs) [29, 30], L‐shaped biaryl imidazo[1,5‐*a*]pyridin‐3‐ylidenes (ImPy) have become indispensable ligands in gold‐catalysis (Figure 1A) [31, 32]. Tailoring the homogeneous gold(I) catalysis is primarily governed by ligand's steric and electronic tuning [33, 34, 35, 36, 37, 38, 39, 40]. Apart from these parameters, gold‐arene secondary interactions could also play a pivotal role in many successful catalytic transformations. The advantages of gold‐arene interactions in catalysis were previously illustrated in Buchwald's biarylphosphines [41, 42, 43, 44], Gessner's ^Ph^YPhos [45, 46], and L‐shaped NHCs [47, 48, 49], where the lateral arene ring is involved in a secondary weak bonding interaction with the catalytically active LAu^+^ species (Figure 1B).

The design of secondary weak bonding interactions for catalysis development could be an ideal advancement in ligand design for future paradigms. In 2017, a new secondary weak bonding mode was revealed by Konishi, where they showed evidence for a rare Au···H–C(sp^2^) hydrogen bonding interaction in divalent hexagold cluster ([Au_6_]^2+^) decorated diphosphine ligands (Figure 1C) [50]. Very recently, Gessner reported a catalytically active gold‐phosphine ligand, Au‐^cy^YPhos, where the Au···H–C(sp^3^) hydrogen bond effectively compensated for the gold‐arene interaction, whereas the adjacent onium centre assisted in polarization of the H–C bond [51]. Reports on such secondary interactions in catalytically active Au(I)–NHC complexes are still elusive. Gold is classified as one of the most electronegative elements and features occupied d‐orbitals, capable of exhibiting Au···H−C interactions [52]. These secondary Au···H–C(sp^3^) interactions in Au‐NHC species could establish a new route for high‐performance gold catalysis [51]. Herein, we report a study that provides an opportunity to introduce a new class of cyclic (amino)ylide carbenes, an unexplored class of carbenes in catalysis due to their low complex stability [53, 54, 55, 56], through steric and electronic assistance.

Cyclic (amino)ylide carbenes are analogues of *N*‐heterocyclic carbenes where one of the nitrogen atoms is replaced by ylide, which significantly increases their σ‐donating ability [57, 58, 59, 60]; however, it also compromises the stability of the carbene center [53]. In our bridged (amino)ylide carbenes, we have installed the sulfur ylide on novel 9*H*‐pyrrolo[1,2‐*a*]indole derivatives (Figure 1D). The pyrroloindole scaffold provides high rigidity through a bridged tertiary center and perfectly aligns itself towards C─H···Au interactions to stabilize the metal. The adjacent ylide carbon offers two advantages: (i) being a stable ylide, a polarized bond pushes the π‐electron toward carbene; hence, electron filling or occupancy increases. As a result, HOMO orbitals are further boosted, offering more σ‐donation ability; (ii) when compared to regular imidazolium carbenes, ylidic carbon has a lower electronegativity than the nitrogen atom; hence, the ‐I effect decreases, and the electron density increases at the carbene center. Thus, the σ‐donating character increases at the carbene center.

DFT studies also revealed that the HOMO‐orbital (σ‐donating orbital) of these cyclic amino(sulfur)ylide (*^b^* CAYC) carbenes (−4.96 to −5.07 eV) is higher than that of CAAC ligands (−5.33 eV) and regular IPr‐type ligands (−6.01 eV) (Figure 1E). While stabilizing such reactive carbenes is not an easy task [53], our *^b^* CAYC ligands can be successfully stabilized through their unique steric and secondary Au···H–C(sp^3^) interaction in Au‐complexes. The presence of this exclusive bonding feature was comprehensively investigated through X‐ray, NMR, and DFT studies. The stability of the gold complexes is further demonstrated through the room‐temperature hydroamination reactions, which are unsuccessful using other NHC‐gold complexes under similar conditions. This class of catalysts also offers remarkable catalytic activity, even at 100°C for tandem C–N and C–C bond formation.

## 2 Results and Discussion

### 2.1 Ligand Synthesis

**SCHEME 1:**
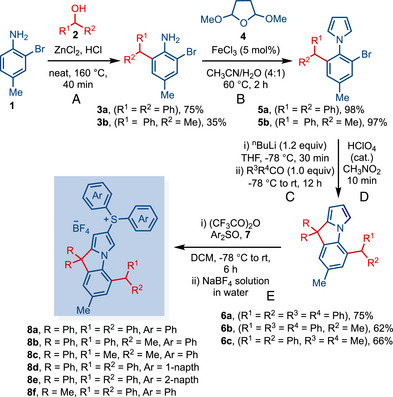
Synthesis of Bridged cyclic (amino)ylide Carbene Precursor.*^a‐e a^* Conditions: (A) **1** (1.0 equiv), diphenylmethanol (1.0 equiv), ZnCl_2_ (0.5 equiv), HCl (1.0 equiv), neat, 160°C, 40 min. (B) **3a** (1.0 equiv), 2,5 dimethoxytetrahydrofuran (1.2 equiv), FeCl_3•_6H_2_O (5 mol%), acetonitrile/water (4:1), 60°C, 2 h. (C) **5** (1.0 equiv), *N*BuLi (1.2 equiv), THF, −78°C, 30 min, then, R_2_C = O (1.0 equiv) −78°C to rt, 12 h. (D) perchloric acid (cat.), nitromethane, 10 min. (E) **6** (1.0 equiv), aryl sulphoxide **7** (1.0 mmol), −78°C, trifluoroacetic anhydride (1.0 equiv), DCM, −78°C to rt.

The ligand synthesis is initiated from the commercially available 2‐bromo‐4‐methylaniline **1**, which readily participates in Friedel–Crafts alkylation with benzyl alcohol **2** in the presence of zinc chloride and HCl to afford the products **3a** and **3b** on a multigram scale (35%–75%) (Scheme 1A) [61]. The reaction between anilines **3** and 2,5‐dimethoxytetrahydrofuran **4** provided the desired pyrrole products **5** (**5a**, 98% and **5b**, 97%) under Lewis acidic conditions in CH_3_CN/H_2_O (4:1) (Scheme 1B). The pyrrole products **5a‐b** were subjected to *^n^* BuLi at −78°C to afford the *ortho*‐lithiated species, providing the alcohol products by quenching with the corresponding ketones. Treatment with a catalytic amount of perchloric acid in nitromethane furnished the cyclized pyrrolo[1,2‐*a*]indole products **6** (Scheme 1C,D). The compounds **6** were subjected to trifluoroacetic anhydride and diarylsulfoxide **7** to afford sulfonium salts, which were further treated with aqueous NaBF_4_ to provide compounds **8** as desired ligands (Scheme 1e). Compound **8c** was synthesized through a different route because isopropanol was not compatible with step (a). Thus, **8c** was synthesized from 2,6‐dibromo‐4‐methylaniline using steps (B–D), Suzuki reaction, hydrogenation, and step (e) [61]. Overall, this developed synthetic route is highly practical and could be used on a multigram scale, starting from very inexpensive starting materials.

**SCHEME 2:**
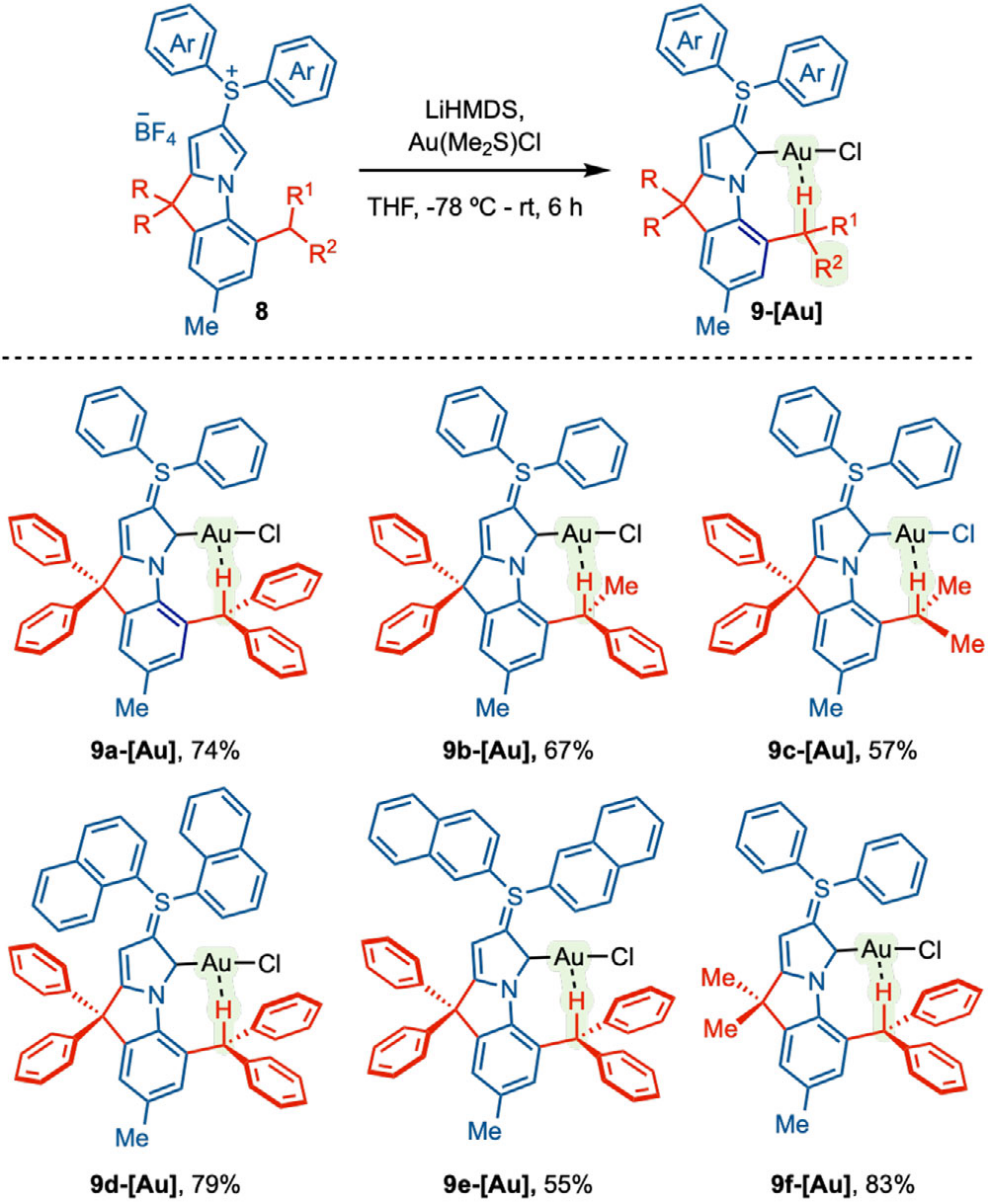
Synthesis of *^b^* CAYC‐Au Complexes.*^a^* *^a^* AuCl·Me_2_S (1.0 equiv), LiHMDS (3.0 equiv), THF, −78°C—rt, 6 h.

After securing a robust synthetic route, we started exploring the synthesis of gold complexes. After several optimizations, LiHMDS was found to be the best base for this reaction. We have synthesized six different *^b^* CAYC‐Au complexes **9a‐[Au]**–**9f‐[Au]** using Au(Me_2_S)Cl in THF by reacting at −78°C to rt for 6 h (Scheme 2). After solvent removal and filtration, *^b^* CAYC‐gold complexes were conveniently obtained through recrystallization from CH_2_Cl_2_/hexane (1:10). In the first three complexes **9a‐[Au]**–**9c‐[Au]**, we gradually decreased the steric impact at the *ortho*‐position from Ph/Ph, Ph/Me to Me/Me. The yield of the complexation reaction also gradually decreased in the order of **9a‐[Au]** (74%), **9b‐[Au]** (67%), and **9c‐[Au]** (57%). This can be attributed to the stability of Au‐complexes. As the steric hindrance at the C13 center decreases, the stability of Au‐complexes also decreases, which results in a decrease in yield. Subsequently, we also showed how steric effects the Au···H─C interactions as well as their effect on Au‐catalysis (*vide infra*). We also modulated the steric properties of sulfur ylides by replacing the phenyl group with 1‐naphthyl and 2‐naphthyl moieties and forming the corresponding gold complexes **9d‐[Au]** (79%) and **9e‐[Au]** (55%). The phenyl groups at the rigid tertiary center (C13) were also tuned by two methyl groups in complex **9f‐[Au]** (83%).

### 2.2 Crystallographic Analysis

**FIGURE 2:**
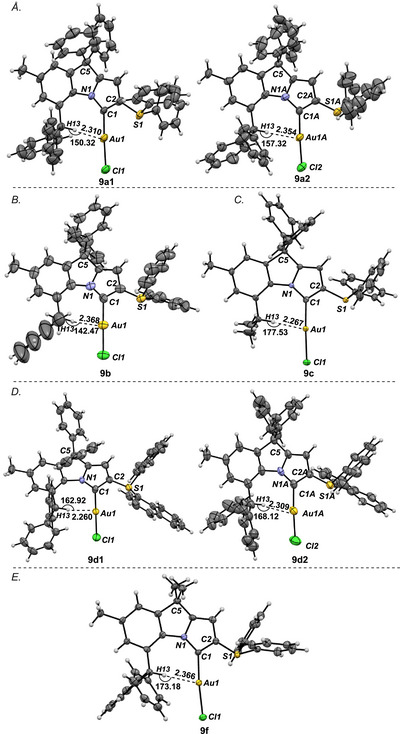
X‐ray structures of complexes **9a‐[Au]**–**9f‐[Au]**. (A). X‐ray crystal structure of complex **9a‐[Au]**. Hydrogen atoms and solvent molecules have been omitted for clarity. Selected bond lengths [Å] and angles [°]: **9a1‐[Au]**: Au1–Cl1, 2.289(2); Au1–C1, 2.012(7); C1–C2, 1.389(10); N1–C1, 1.376(8); S1–C2, 1.729(7), Au1–H13, 2.310; C1–Au1–C11, 177.38(19); N1–C1–C2 103.3(6); N1–C1–Au1, 134.1(5); C2–C1–Au1, 121.7(5); C13–H13–Au1 150.3. **9a2‐[Au]**: Au2–Cl2, 2.275(2); Au2–C1A, 2.011(7); C1A–C2A, 1.379(10); N1A–C1A, 1.381(8); S2–C2A, 1.725(7); Au2–H13A, 2.354; C1A–Au2–Cl2, 176.86(19); C2A–C1A–N1A 103.8(6); N1A–C1A–Au2, 133.1(5); C2A–C1A–Au2, 122.4(5); C13A–H13A–Au1A 157.3. (B). X‐ray crystal structure of complex **9b‐[Au]**. Selected bond lengths [Å] and angles [°]: Au1–Cl1, 2.298(4); Au1–C1, 2.044(13); C1–C2, 1.398(17); N1–C1, 1.342(17); S1–C2, 1.735(13), Au1–H13, 2.368; Cl–Au1–C11, 178.7(4); N1–C1–C2 104.9(11); N1–C1–Au1, 135.8(9); C2–C1–Au1, 119.2(10); C13–H13–Au1 142.5. (C). X‐ray crystal structure of complex **9c‐[Au]**. Selected bond lengths [Å] and angles [°]: Au1–Cl1, 2.2885(13); Au1–C1, 1.993(5); C1–C2, 1.389(7); N1–C1, 1.383(6); S1–C2, 1.725(5), Au1–H13, 2.267; Cl–Au1–C11, 177.04(14); N1–C1–C2 103.2(4); N1–C1–Au1, 133.4(3); C2–C1–Au1, 123.1(4); C13–H13–Au1 177.5. (D). X‐ray crystal structure of complex **9d‐[Au]**. Selected bond lengths [Å] and angles [°]: **9d1‐[Au]**: Au1–Cl1, 2.307(17); Au1–C1, 1.993(6); C1–C2, 1.408(8); N1–C1, 1.401(7); S1–C2, 1.727(6), Au1–H13, 2.260; C1–Au1–C11, 179.0(17); N1–C1–C2 102.9(5); N1–C1–Au1, 132.7(4); C2–C1–Au1, 124.3(4); C13–H13–Au1 162.9. **9d2‐[Au]**: Au2–Cl2, 2.280(2); Au2–C1A, 2.013(7); C1A–C2A, 1.398(8); N1A–C1A, 1.367(8); S2–C2A, 1.734(6); Au2–H13A, 2.309; C1A–Au2–Cl2, 176.42(17); C2A–C1A–N1A 103.3(5); N1A–C1A–Au2, 135.2(4); C2A–C1A–Au2, 121.2(5); C13A–H13A–Au1A 168.1. (E). X‐ray crystal structure of complex **9f‐[Au]**. Selected bond lengths [Å] and angles [°]: Au1–Cl1, 2.2907(10); Au1–C1, 1.992(4); C1–C2, 1.409(5); N1–C1, 1.382(5); S1–C2, 1.734(4), Au1–H13, 2.366; Cl–Au1–C11, 174.68(11); N1–C1–C2 102.9(3); N1–C1–Au1, 135.7(3); C2–C1–Au1, 121.0(3); C13–H13–Au1 173.2. CCDC 2467391 (**9a‐[Au]**), CCDC 2467754 (**9b‐[Au]**), CCDC 2467756 (**9c‐[Au]**), CCDC 2456304 (**9d‐[Au]**), CCDC 2467757 (**9f‐[Au]**). See ref. [68].

Five complexes were fully characterized by X‐ray crystallography (Figure 2). These linear Au(I) complexes were also used for quantifying the steric impact of *^b^* CAYC ligands. The X‐ray crystallographic data analysis of complexes (**9a‐[Au]**–**9d‐[Au]** and **9f‐[Au]**) showed that in all the cases, Au is linearly bound to the carbene and chlorine atoms (174.7° to 179.0°) (Figure 2). Two molecules are present in the asymmetric unit of **9a‐[Au]** (**9a1‐[Au]** and **9a2‐[Au]**) (Figure 2A) and **9d‐[Au]** (**9d1‐[Au]** and **9d2‐[Au]** (Figure 2D). Most notably, a remarkably short r_Au···H_ bond distance (2.26 Å–2.368 Å) was observed in all complexes, highlighting the possible presence of Au···H–C(sp^3^) interaction or anagostic interactions [62, 63, 64], where Au atom acts as an electron donor and the C–H moiety as an acceptor [52]. In general, Au···H–C interactions are very rare due to less polar H–C bonds compared to N–H and O–H bonds [65, 66, 67]. Sollogoub in a computational study, revealed that methane complexes of NHC‐Au‐Cl showed a similar interaction, highlighting that Au···H–C hydrogen bonds are not entirely the result of steric congestion [63, 64].

In the present case, Au···H distances for complex ([Au(*^b^* CAYC–SPh_2_–Ph/Ph)^Ph2^Cl] **9a‐[Au]** are 2.310 Å (**9a1‐[Au]**) and 2.354 Å (**9a2‐[Au]**). The two analogous complexes [Au(*^b^* CAYC–SPh_2_–Ph/Me)^Ph2^Cl] **9b‐[Au]** (Figure 2B) and ([Au(*^b^* CAYC–SPh_2_–Me/Me)^Ph2^Cl] **9c‐[Au]** (Figure 2C) also showed similar Au···H distances of 2.368 Å and 2.267 Å, respectively. 1‐Naphthyl‐substituted complex ([Au(*^b^* CAYC–S(1‐naph)_2_–Ph/Ph)^SPh2^Cl], **9d1‐[Au]** revealed the lowest r_Au···H_ distance of 2.260 Å among all *^b^* CYAC‐Au‐complexes (**9a‐[Au]**–**9d‐[Au]** and **9f‐[Au]**). The other molecule in the same asymmetric unit **9d2‐[Au]** showed higher r_Au···H_ = 2.309 Å. C5‐dimethyl‐substituted complex ([Au(*^b^* CAYC–SPh_2_–Ph/Ph)^Me2^Cl] **5f‐[Au]** (Figure 2E) indicated a similar Au···H–C distance of r_Au···H_ = 2.366 Å. The data suggested that these bond lengths are well shorter than the sum of the van der Waals radii (2.86 Å) and are clearly well within the range of the hydrogen bonding parameter [69]. These data represent the shorter Au···H distance (r_Au···H_ = 2.260 Å, for **9d1‐[Au]**), superseding the previous elegant reports by Gessner (r_Au···H_ = 2.38 Å) [51], Koshini (r_Au···H_ = 2.62 Å avg) [50], Inoue (r_Au···H_ = 2.70 Å) [70, 71, 72, 73] and others [71, 72, 73]. It is remarkable that the present Au···H–C distance is very close to the Au···H–N distance (2.24 Å) reported by Bourissou in Au‐MeDalphos complex [52], considering that N─H bond is more polar than C─H bond.

The bond angles between Au···H–C bond in Au‐complexes (**9a‐[Au]**–**9d‐[Au]** and **9f‐[Au]**) range from 142.5° to 177.5°. The Au···H–C bond angles are as follows: **9a1‐[Au]**, ∠C13–H13–Au1 = 150.3°; **9a2‐[Au]**, ∠C13A–H13A–Au2 = 157.3°; **9b‐[Au]**, ∠C13–H13–Au1 = 142.5°; **9c‐[Au]**, ∠C13–H13–Au1 = 177.5°; **9d1‐[Au]**, ∠C13–H13–Au1 = 162.9°; **9d2‐[Au]**, ∠C13A–H13A–Au2 = 168.1°; **9f‐[Au]**, ∠C13–H13–Au1 = 173.2°. In complex **9c‐[Au]**, the bond angle is nearly linear (177.5°), indicating suitability for bonding interaction with gold‐d‐orbitals. Because of the different substitutions at the C13, C5, and S1 positions, torsional strain is also different for each complex. Therefore, no linear relationship was established between the Au···H–C bond angles and Au‐H distances. In ligand **8a**, the torsional angle between the carbene plane and the C‐H plane is ∼45°. However, in [Au]‐complex, the angles come down to ∼21°, which means more alignment with the Au centre (see Supporting Information) [61].

### 2.3 Buried Volume Analysis

**FIGURE 3:**
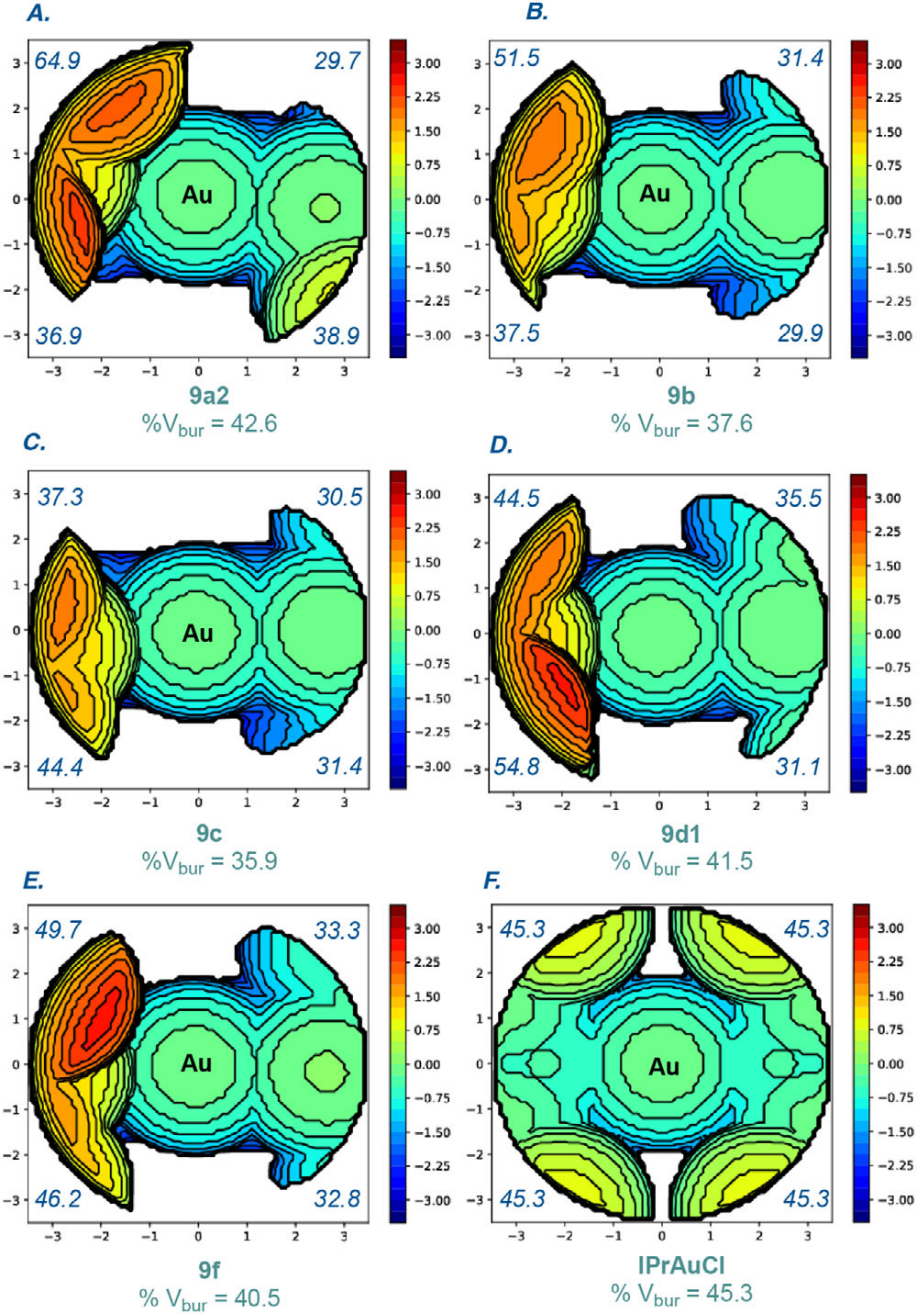
Topographical steric maps of **9a2‐[Au]**, **9b‐[Au]**, **9c‐[Au]**, **9d1‐[Au],** and **9f‐[Au]** showing %V*_bur_* per quadrant. See Supporting Information for additional details.

Next, we used the percentage buried volume (%V*_bur_*) method, pioneered by Nolan, Cavallo et al. to quantify the steric impact of bridged pyrroloindole CYAC ligands [74, 75, 76]. The %V*_bur_* of complex **9a2‐[Au]** (Figure 3A), **9b‐[Au]** (Figure 3B) and **9c‐[Au]** (Figure 3C) showed %V_bur_ = 42.6% (SW, 36.9%; NW, 64.9%; NE, 29.7%; SE, 38.9%), 37.6% (SW, 37.5%; NW, 51.5%; NE, 31.4%; SE, 29.9%), 35.9% (SW, 44.4%; NW, 37.3%; NE, 30.5%; SE, 31.4%) respectively. The results are well in accordance with the gradual increase in steric in **9c‐[Au]** to **9a2‐[Au]**. The largest difference in steric impact in complex **9a2** was observed between NW (64.9%) versus NE (29.7%), for **9b‐[Au]** NW (51.5%) versus SE (29.9%), and for **9c‐[Au]** SW (44.4%) versus NE (30.5%).

We observed that with decreasing steric in **9a2‐[Au]** → **9b‐[Au]** → **9c‐[Au]**, quadrants NW and SW mostly showed the deviation, decreased in the NW quadrant (65.0% vs. 51.5% vs. 37.3%), and increased in the SW quadrant (36.9% vs. 37.5% vs. 44.4%). The least effect of the steric impact was in the NE quadrants (29.7% vs. 31.4% vs. 30.5%). The other two gold complexes, **9d1‐[Au]** (Figure 3D) and **9f ‐[Au]** (Figure 3E), which feature differences at sulfur substitution and C5 backbone, showed the %V*_bur_* of 41.5% (SW, 54.8%; NW, 44.4%; NE, 35.5%; SE, 31.1%) and 40.5% (SW, 46.2%; NW, 49.7%; NE, 33.3%; SE, 32.8%) respectively. Interestingly, phenyl to methyl substitution at C5 backbone, **9a2‐[Au]** → **9f‐[Au]** starkly affected the steric impact at NW (65.0% vs. 49.7%) and SW (36.9% vs. 46.2%) quadrant. The comparison between the steric map of IPrAuCl emphasized (Figure 3F) the difference in the unsymmetrical steric distribution in our complexes.

### 2.4 Electronic Property

**SCHEME 3:**
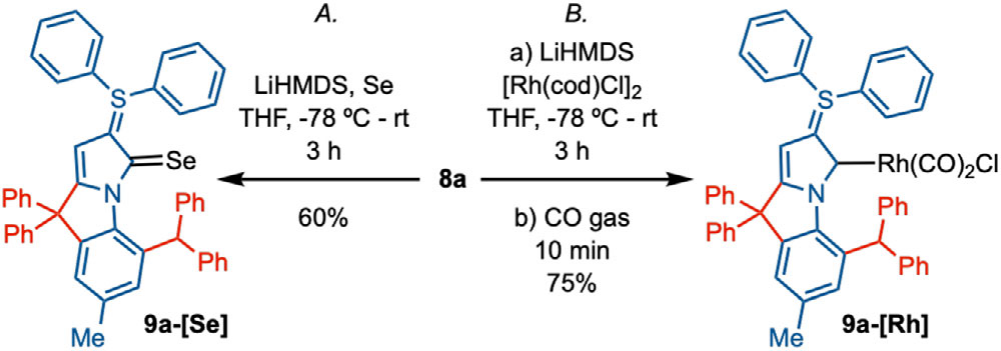
(A) Synthesis of *^b^* CAYC‐Se Complexes, Se (3.0 equiv.), LiHMDS (2.0 equiv), THF, −78°C—rt, 3 h, 60%. (B) [Rh(cod)Cl]_2_ (0.6 equiv.), LiHMDS (2.0 equiv), THF, −78°C—rt, 3 h., then CO gas, 10 min, 75%.

To evaluate the electronic properties, the *^b^* CAYC‐Se complex [Se(*^b^* CAYC–SPh_2_–Ph/Ph)^Ph2^] **9a‐[Se]** was synthesized by reacting ligand **8a** with LiHMDS in the presence of selenium in 60% yield (Scheme 3A.). The ^77^Se NMR value of 177.8 ppm (CDCl_3_) indicates good π‐acceptor property of the ligand [34, 77, 78]. The reaction of **8a** with [Rh(cod)Cl]_2_ in the presence of LiHMDS, followed by the insertion of carbon monoxide (CO), delivered the [Rh(*^b^* CAYC–SPh_2_–Ph/Ph)^Ph2^(CO)_2_Cl] **9a‐[Rh]** in 75% yield. TEP (Tolman electronic parameter) of **9a‐[Rh]** has been measured, and it corresponds to 2042 cm^−1^. This value indicates one of the strongest σ‐donating NHC ligands prepared to date [34].

### 2.5 NMR Analysis

**FIGURE 4:**
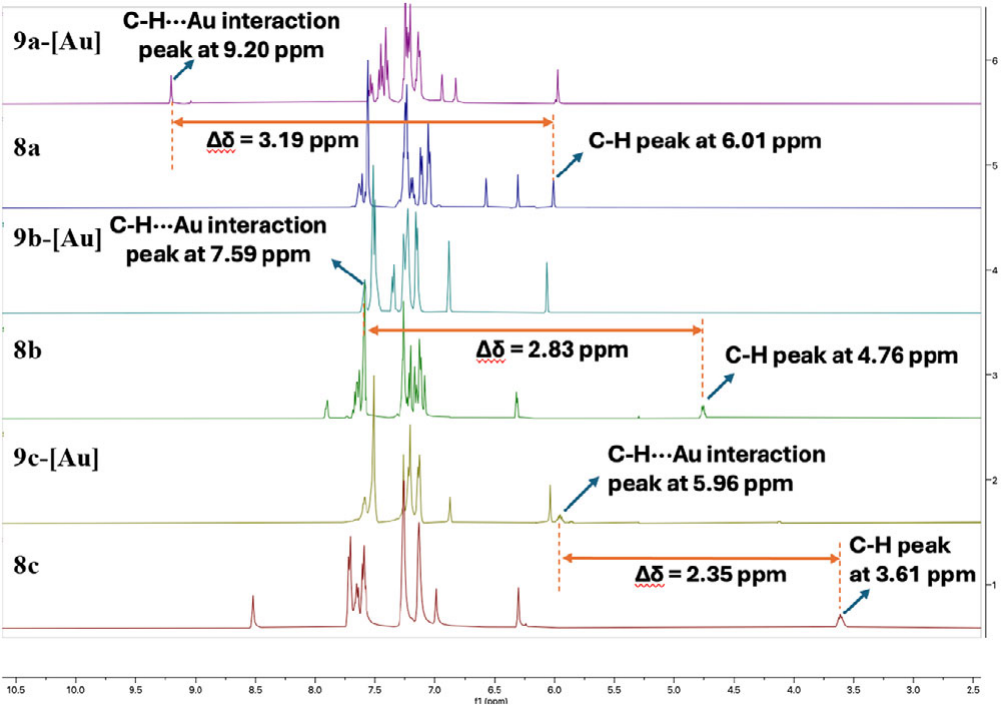
Downfield shift in ^1^H NMR for complexes **9a‐[Au]**, **9b‐[Au]**, and **9c‐[Au]** with their corresponding ligands **8a**, **8b**, and **8c** in Δδ ppm.

After finding evidence for the presence of C−H···Au(I) intramolecular interactions in five different Au‐carbene complexes in the solid state through single‐crystal X‐ray studies, we investigated the existence of the C−H···Au(I) interactions in solution via NMR spectroscopy. ^1^H NMR signals of the gold‐neighboring wingtip sp^3^ C−H bond are crucial in mapping the interaction. In the CDCl_3_ solution, ^1^H NMR of complex **9a‐[Au]** showed a remarkable downfield shift of δ = 9.20 ppm for the sp^3^ C–H bond (Figure 4), indicating the interaction with the proximal gold center. A significant difference of Δδ = 3.19 ppm was observed for complex **9a‐[Au]** and ligand **8a**, portraying the consequence of the introduction of Au‐center.

The downfield shift in ^1^H NMR for other complexes was also quite noteworthy: δ = 4.76 ppm to δ = 7.59 ppm for **9b‐[Au]** (Δδ = 2.83 ppm), δ = 3.61 ppm to δ = 5.96 ppm for **9c‐[Au]** (Δδ = 2.35 ppm). The gradual increase in the difference in the downfield shift from **9a‐[Au]** to **9c‐[Au]** can be rationalized through the pK*_a_* values. From the hyperconjugation viewpoint, the triaryl sp^3^ C–H bond offers a more acidic, more polarized bond, which brings greater polarization with the gold center. This consequence was further supported by DFT analysis, which showed a longer bond length for **9a‐[Au]** (1.095 Å) than for **9c‐[Au]** (1.091 Å) [61].

To measure the 𝜎‐donating capability, ^1^J_CH_ coupling constant was measured at the precarbenic center of the **8a** ligand through ^1^H‐^13^C HSQC (coupled) NMR. The ^1^J_CH_ coupling constant value was 198.2 Hz (in CDCl_3_) and 201.0 Hz (in DMSO), predicting a strong 𝜎‐donating character of our ligand [77, 78]. Another ligand, **8f**, also showed a ^1^JCH coupling constant of 197.3 Hz (CDCl_3_), providing a similar range. ^1^J_CH_ for the interacting C(13)‐H(13) in gold‐complex **9a‐[Au]** was measured through ^1^H‐^13^C HSQC (coupled) NMR and valued as 123.5 Hz, compared with its corresponding non‐interacting C–H in ligand **8a** as 128.3 Hz. A decrease in the ^1^J_CH_ coupling constant in gold‐complex indicates that the interacting C─H bond weakens in gold‐complex **9a‐[Au]** compared to ligand **9a**, a proof for weak Au···H–C(sp^3^) interaction [50].

**SCHEME 4:**
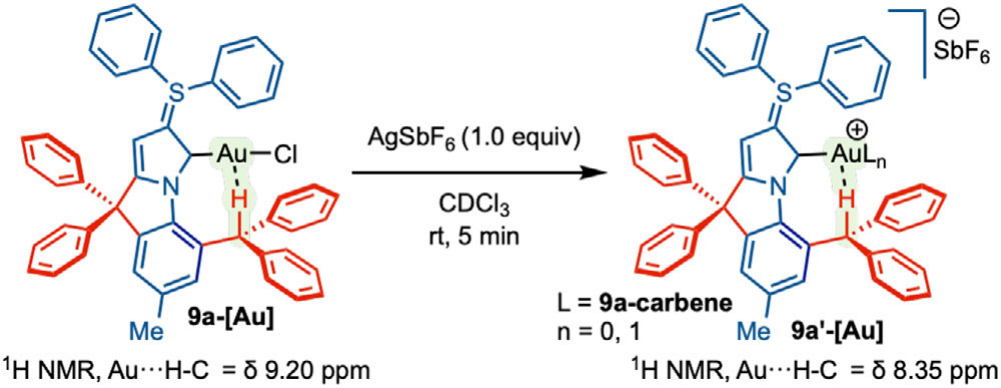
^1^H NMR Study of Au···H–C Bond in **9a**‐[Au] and possible cationic complex **9a'**‐[Au].

Furthermore, we attempted to synthesize cationic gold‐complex **9a'‐[Au]** [Au(NHC)^2+^SbF_6_^−^ or Au(NHC)^+^SbF_6_^−^] from the corresponding complex **9a‐[Au]** in the presence of silver hexafluoroantimonate (Scheme 4). The ^1^H NMR data of **9a'‐[Au]** suggested that the C–H···Au peak was shifted to δ = 8.35 ppm, a significant upfield shift of (Δδ = 0.85 ppm compared to **9a‐[Au]** (δ = 9.20 ppm) [61]. This result can be rationalized through ‘contra‐electrostatic’ behavior of C‐H···Au interaction, which involves an interplay between n_M_ → σ*_C‐H_ stabilizing hyperconjugation and repulsive/destabilizing electrostatics [63, 64]. As the cationic character increases at the Au‐center, the power of electron donation to the σ* (C‐H) decreases, and the destabilizing repulsive electrostatic force also increases, eventually leading to a weaker hydrogen bond and upfield shift.

**FIGURE 5:**
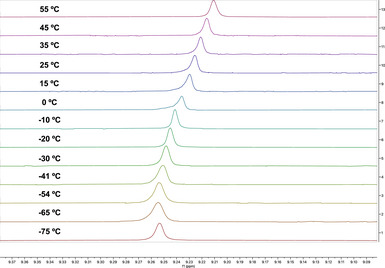
The upfield shift in ^1^H NMR from a lower to a higher temperature for C−H···Au(I) bond in complex **9a‐[Au]**.

The variable temperature ^1^H NMR data, from −75°C to 55°C, showed an upfield deviation of the C–H bond at wingtips (Figure 5). In general, from lower to higher temperatures, the influence of hydrogen bonding drops due to the increase in vibrational motion and kinetic energy.

### 2.6 DFT Analysis

**FIGURE 6:**
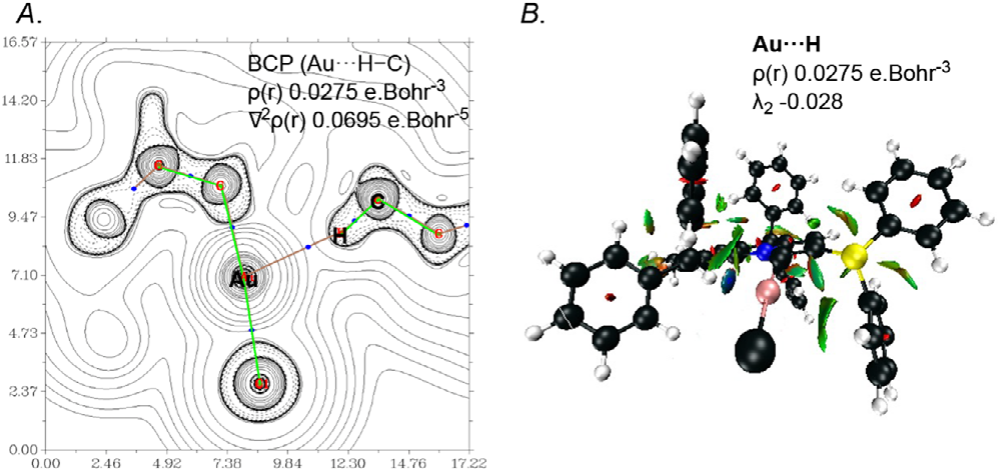
(A) Contour plot of the Laplacian distribution ∇2ρ(r) for **9a‐[Au]** with relevant bond paths and BCPs (blue spheres). Hydrogen atoms have been omitted for clarity, except for that on the carbon atom (13C). (B) NCI plots for **9a‐[Au]**. Coloured in a blue‐green‐red scheme over the range of (−0.05 < sign(*λ*_2_)*ρ* <0.05 a.u.) and isosurface of RDG = 0.5. Blue indicates strong attraction, green indicates weak interaction, and red indicates repulsion.

To prove the bonding interactions, we analyzed the bonding through atoms in molecules (AIM), noncovalent interaction (NCI) and natural bond orbital (NBO) analyses of the gold complexes **9a‐[Au]**, **9b‐[Au]**, **9c‐[Au]**, and their corresponding carbenes. This theoretical method, which focuses on electron density, offers valuable insights for analyzing chemical bonding, including even the subtleties of weak interactions. AIM data in gold complexes shows that the hydrogen‐gold bond distances are in a similar range (2.3113–2.3663 Å) in comparison to their X‐ray analysis data. AIM analysis calculations indicate the presence of a bond critical point (BCP) between gold (Au) and hydrogen (H) in all three complexes. This is characterized by an electron density of ρ(r) = 0.0275 ebohr^−3^ and a positive Laplacian of ∇^2^ρ(r) = 0.0695 ebohr^−5^ (Table 1). The Laplacian is positive, while the second Hessian eigenvalue λ_2_ is negative (−0.028) for **9a‐[Au]**, which aligns with the bonding noncovalent Au∙∙∙H interaction. This picture is validated by the NCI plot (Figure 6). The negative value λ_2_ between Au and H is indicative of attractive NCI and is consistent with H···Au hydrogen bonding, suggesting a moderate hydrogen bond between Au and H [51, 52].

**TABLE 1:** Computational studies on the secondary ligand gold interactions in the gold complexes **9a‐[Au]**, **9b‐[Au]**, **9c‐[Au]**, and the corresponding free carbenes at the B3LYP 6–311++G(d,p) using Grimme D3 correction. For details, see the Supporting Information.

Compound	d_C‐H_ [Å]	d_H‐Au_ [Å]	*ρ*(r) [e bohr^−3^]	∇^2^ *ρ*(r) [e bohr^−5^]	λ_2_	*ν* (CH) [cm^−1^]	*Dν* (CH) [cm^−1^]	Wiberg bond order C‐Au	Wiberg bond order H‐Au
**9a‐[Au]**	1.0960	2.3113	0.0275	0.0695	−0.028	2969.3	−58.7	0.8419	0.0399
**9a‐carbene**						3028.0		0.8553	
**9b‐[Au]**	1.0949	2.3253	0.0268	0.0685	−0.027	2988.8	−43.7	0.8557	0.0363
**9b‐carbene**						3032.5		0.8622	
**9c‐[Au]**	1.0948	2.3663	0.0249	0.0651	−0.024	2997.1	−52.1	0.8670	0.0312
**9c‐carbene**						3049.3		0.8709	

Other two complexes **9b‐[Au]** and **9c‐[Au]** also showed similar results, **9b‐[Au]**: ρ(r) = 0.0268 ebohr^−3^, ∇^2^ρ(r) = 0.0685 ebohr^−5^, λ_2_ = −0.027; **9c‐[Au]**: ρ(r) = 0.0249 ebohr^−3^, ∇^2^ρ(r) = 0.0651 ebohr^−5^, λ_2_ = −0.024. ρ(r) and ∇^2^ρ(r) are gradually decreases in the order from **9a‐[Au]** → **9b‐[Au]** → **9c‐[Au]**, suggesting the lowering of Au···H–C interaction. Likewise, the C–H bonds participating in the bonds appear to be slightly elongated when compared to the non‐interacting C–H bonds of free carbene, with values shifting from 1.0946 to 1.0960 Å (see Supporting Information). Historically, the observation of bond critical points and deshielding effects has sparked thoughtful discussion, with various interpretations. Some researchers attribute these phenomena to steric compression within a complex, rather than solely to Au‐H hydrogen bonding [79, 80]. Steric compression generally leads to a stiffer C‐H vibration, whereas hydrogen bonding tends to weaken and soften the C‐H vibrational mode. The vibration in gold‐complex **9a‐[Au]** was red‐shifted by 58.7 cm^−1^ compared to the corresponding free ligand, supporting the presence of a weak hydrogen bond. The other two gold complexes, **9b‐[Au]** and **9c‐[Au]**, were also red‐shifted by 43.7 cm^−1^ and 52.1 cm^−1^.

The Wiberg Bond Index (WBI) for the C–H bond shows a decrease from 0.86 to 0.84 in compounds from free carbene to the corresponding gold complex **9a‐[Au]**. It is also important to highlight that the WBI for the Au···H interaction in **9a‐[Au]**, although relatively small, is nonetheless significant considering the H−C(*sp^3^*) bond, recorded at 0.04. A similar trend was also observed for the complexes **9b‐[Au]** and **9c‐[Au]**. Overall, these studies indicate weakening of the C–H bond following its interaction with gold.

**FIGURE 7:**
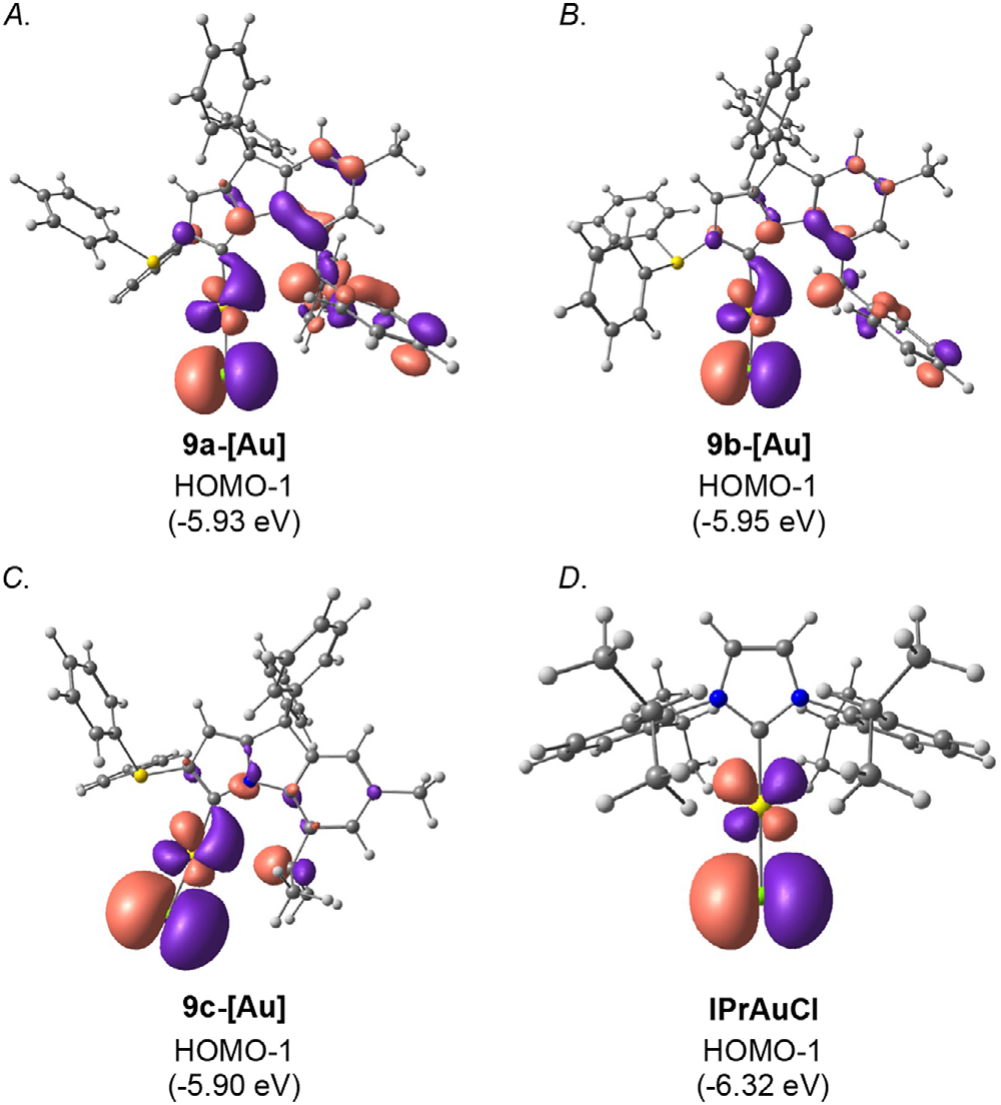
HOMO‐1 orbital of *^b^* CAYC ligands. B3LYP 6–311++g(d,p) level. See Supporting Information for details.

Further, to probe the electronic properties of gold complexes **9a‐[Au]**–**9c‐[Au]**, HOMO and LUMO energy levels and diagrams were determined at the B3LYP 6–311++g(d,p) level (Figure 7 and Supporting Information). The HOMO‐1 levels of **9a‐[Au]** (−5.93 ev), **9b‐[Au]** (−5.95 ev), and **9c‐[Au]** (−5.90 ev) (Figures 7A–C) clearly show an anisotropic distribution of gold d‐orbital towards the C–H bond in all the complexes. C–H orbital also showed the perturbation towards the metal center. Comparison with the HOMO‐1 of IPrAuCl which clearly showed no orbital anisotropy in gold‐d orbitals (Figure 5D). Hydrogen bonding is more complex than typical dipole‐dipole interactions, typically involving orbital interactions (d_Au_ → σ*_CH_) and quantum mechanical delocalization, which means it functions as a resonance‐assisted interaction rather than just a basic electrostatic attraction.

### 2.7 Catalytic Studies

**SCHEME 5:**
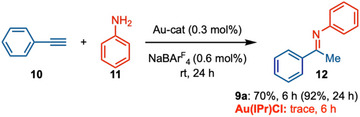
Comparative Study between **9a‐[Au]Cl** and IPrAuCl in Alkyne Hydroamination.

The catalytic performance of our new ligand was evaluated in gold‐catalyzed alkyne hydroamination. Gold complexes are particularly valuable for activating p‐bonds in a variety of processes that have found broad industrial, medicinal, and academic applications. In early studies, alkyne hydroamination reactions mainly relied on Au‐phosphine complexes. In recent years, Au‐NHCs have been recognized as a viable alternative to Au‐phosphines [81, 82]. We evaluated the performance of Au‐*^b^* CAYC catalysts in gold‐catalyzed alkyne‐amine hydroamination reactions. To our delight, catalyst **9a‐[Au]** worked at room temperature with phenylacetylene **10** and aniline **11** at 0.3 mol% catalyst loading to afford the hydroamination product **12** in 72% yield in 6 h and 94% yield in 24 h (Scheme 5). The regular gold‐carbene complex, IPrAuCl, did not produce any product at room temperature under identical conditions.

**SCHEME 6:**
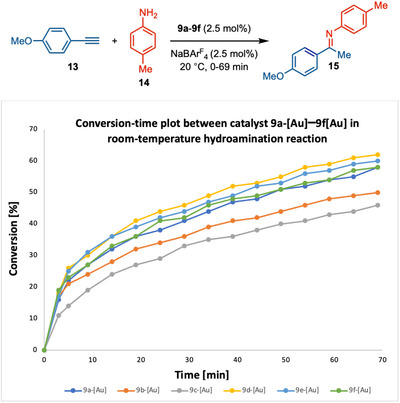
Comparative Study between *^b^* CAYC‐AuCl Catalysts **9a‐[Au**]–**9f‐[Au]** in Alkyne Hydroamination.

We have conducted an in situ kinetic study to gain insight into the catalyst reactivity [83]. We have monitored imine formation in the hydroamination of *para*‐(methoxyphenyl)acetylene **13** and *para*‐toluidine **14** in the presence of *^b^* CAYC‐Au‐catalyst **9a‐[Au]**–**9f‐[Au]** and NaBAr^F^_4_ through ^1^H NMR spectroscopy at 20°C. First, we compared the three catalysts **9a‐[Au]**–**9c‐[Au]** and found that **9a‐[Au]** catalyst was the most efficient among the three catalysts (Scheme 6). Hence, stericity at the C13 center plays an important role in catalyst reactivity. Further, catalyst **9f‐[Au]** showed similar reactivity to complex **9a‐[Au]**, which indicates that steric hindrance at C5 does not have a pronounced effect on catalysis. Complexes with 1‐naphthyl **9d‐[Au]** and 2‐naphthyl sulfur ylide **9e‐[Au]** enhanced the reactivity, and **9d‐[Au]** proved to be the best catalyst among all six complexes. In similar reaction conditions, catalyst **9d‐[Au]** showed better reactivity than *^iPr^* BiCAAC, which was the best catalyst among the various cyclic (alkyl)(amino) carbene for the same reaction [82].

**SCHEME 7:**
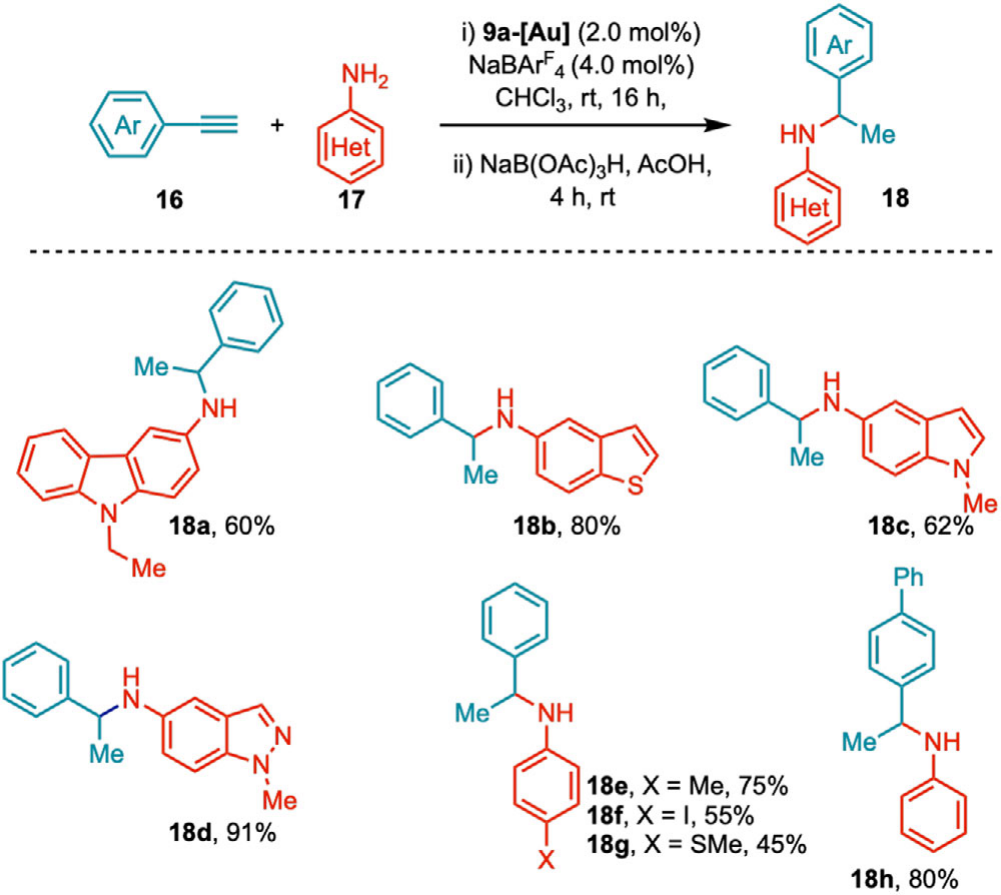
Scope of hydroamination of alkynes through *^b^* CAYC‐Au‐catalysis.

While NHC ligands generally increase the stability of gold complexes, room‐temperature NHCAuCl‐catalyzed hydroamination is a major challenge [51]. Thus, *^b^* CAYC‐Au complexes open an avenue for the room‐temperature hydroamination of challenging substrates, including temperature‐sensitive heterocycles. We have examined different primary amine‐containing carbazole, indole, benzo[*b*]thiophene, indazole moieties, and all worked well under the reaction conditions to afford the products **18a**–**18d** (62%–91%) (Scheme 7). Various 4‐substituted anilines, *p*‐toluidine, 4‐phenylaniline, 4‐(methylthio)aniline, 4‐iodoaniline, also afforded **18e**–**18 h** in good yields (55%–80%).

**SCHEME 8:**
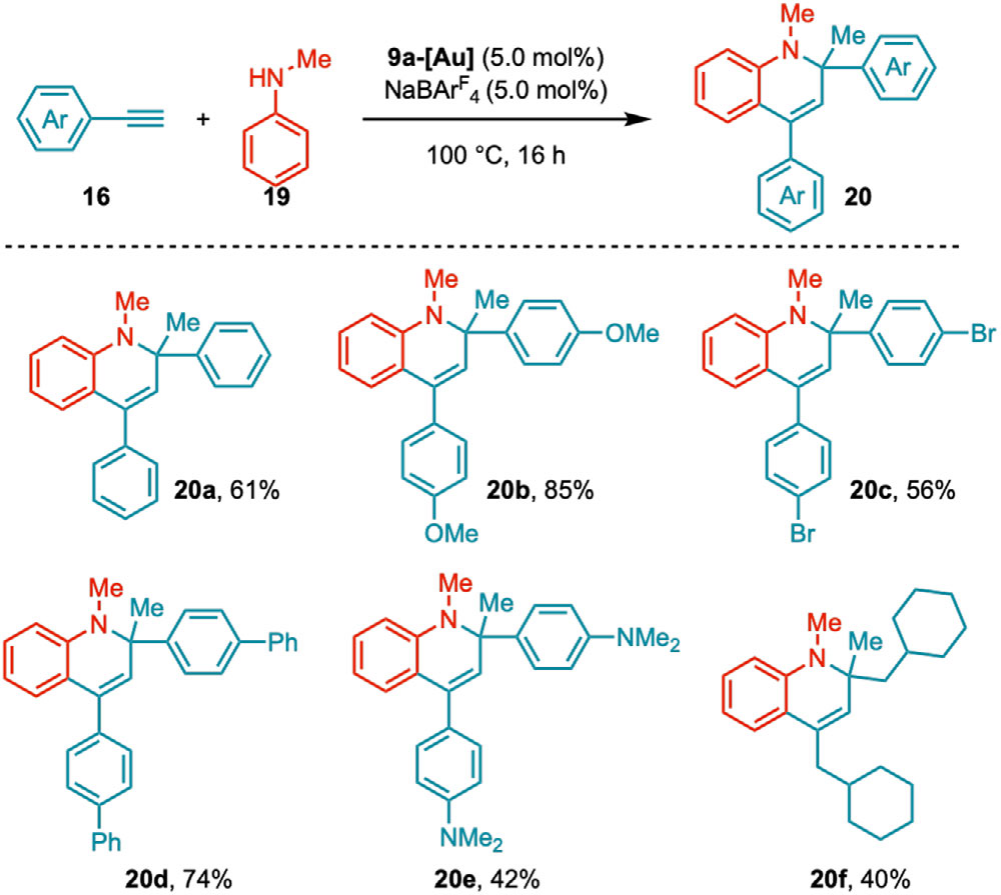
Scope of Tandem C–N and C–C Activation through *^b^* CAYC‐Au‐Catalysis.

*^b^* CAYC‐Au complexes also showcased their efficiency in tandem C–N and C–C bond‐forming reactions for the synthesis of 1,2‐dihydroquinolines (Scheme 8). In this two‐fold activation, hydroamination is followed by C‐H activation to form 1,2‐dihydroquinolines [84]. Electronically diverse, electron‐rich, and electron‐deficient aromatic terminal alkynes were subjected to the reaction and efficiently afforded 1,2‐dihydroquinoline derivatives **20a**‐**20 g** (42%‐85%). Electronically neutral aliphatic alkyne, prop‐2‐yn‐1‐ylcyclohexane, also reacted well to furnish **20** **h** (40%).

### 2.8 Electronic Properties of Exceedingly Strongly‐**σ**‐Donating *^b^* CAYC Ligands

**FIGURE 8:**
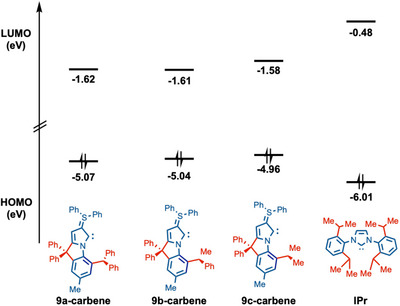
HOMO and LUMO energy levels (eV) at B3LYP 6–311++g(d,p) level.

To gain further insight into the electronic properties of the *^b^* CAYC ligands, HOMO and LUMO energy levels were determined at B3LYP 6–311++g(d,p) level. In order to evaluate the nucleophilicity (more σ‐donating, higher HOMO) and electrophilicity (more σ‐accepting, lower LUMO) of NHC ligands, HOMO and LUMO energy levels are an established method to provide the most accurate results (Figure 8). Thus, a comparison was drawn between the HOMO of **9a‐carbene** (−5.07 eV), **9b‐carbene** (−5.04 eV), **9c‐carbene** (−4.96 eV), and IPr (−6.01 eV). It is evident that HOMO values of *^b^* CAYC ligands are much higher than IPr‐type ligands, suggesting significantly stronger σ‐donicity, which was reflected through successful catalysis. Notably, among the three different *^b^* CAYCs, **9c‐carbene** exhibited the highest HOMO value. All the HOMOs & LUMOs orbitals associated with carbenes **9a**, **9b**, and **9c** were discussed in the Supporting Information with their corresponding energy value [61].

Finally, to eliminate effects potentially resulting from crystal packing, the %percentage buried volume (%V*_bur_*) was calculated from the optimized structures of Au‐complexes **9a‐[Au]**–**9c‐[Au]** at the B3LYP 6–311++g(d,p) level (see Supporting Information) [61]. The calculation showed the %V*_bur_* of the NHC in **9a‐[Au]** as 39.2% (SW, 36.0%; NW, 59.1%; NE, 32.4%; SE, 29.2%); in **9b‐[Au]** as 38.3% (SW, 35.1%; NW, 47.9%; NE, 30.8%; SE, 39.5%); in **9c‐[Au]** as 36.2% (SW, 44.3%; NW, 35.7%; NE, 34.4%; SE, 30.2%). The data clearly show that %V*_bur_* decreases as the steric decreases from **9a‐[Au]** → **9b‐[Au]** → **9c‐[Au]** (39.2%, 38.3% vs. 36.2%). The trends are well‐matched with the %V*_bur_* from crystal structures. Significant differentiation was noticed with a gradual decrease in the NW quadrant from **9a‐[Au]** → **9b‐[Au]** → **9c‐[Au]**: 59.1% versus 47.9% versus 35.7%. A noteworthy change was also observed for **9a‐[Au]** and **9c‐[Au]** at SW quadrant 36.0% versus 44.3%. Additionally, the NE quadrant was the least affected by steric variation from **9a‐[Au]** → **9b‐[Au]** → **9c‐[Au]**: 32.4% versus 30.8% versus 34.4%. This unsymmetrical distribution of flexible steric hindrance in combination with the exceedingly strong σ‐donation are key factor in catalysis.

## 3 Conclusion

Gold catalysis has emerged as an increasingly powerful platform in organic synthesis. In this manuscript, we have reported unique electronically and sterically designed bridged cyclic (amino)ylide carbene ligands (*^b^* CAYC) and their corresponding gold complexes, featuring a weak intramolecular Au···H−C(sp^3^) interaction as a secondary interaction. Studies by X‐ray crystallography, NMR, DFT, AIM, NBO, and NCI plot all support the presence of the Au···H−C(sp^3^) interaction. The Au···H−C bond distances and C–H–Au bond angles align with the properties of Au···H−C(sp^3^) interaction. This study reports the remarkably short Au···H−C(sp^3^) distance of 2.26 Å. To quantify the steric impact, the %percentage buried volume (%V*_bur_*) was calculated through the Cavallo method, with %V*_bur_* up to 42.6%, showcasing the prevailing steric impact. From the electronic viewpoint, these *^b^* CAYC carbenes supersede other strongly σ‐donating carbenes, such as cyclic (alkyl)(amino) carbenes, mesoionic carbenes, and imidazolium carbenes, indicating some of the most strongly σ‐donating ligands to date. The overall influence of the steric, electronic, and Au···H−C(sp^3^) interactions impacts the catalytic performance, presenting successful room‐temperature hydroamination and tandem C–N and C–C bond formation.

## Conflicts of Interest

The authors declare no conflicts of interest.

## Supporting information




**Supporting File1**: anie71581‐sup‐0001‐SuppMat.pdf.

**Supporting File2**: anie71581‐sup‐0002‐Data.zip.

## Data Availability

The data that support the findings of this study are available in the supplementary material of this article.
