# Supplementary material for: Bridged Pyrroloindole‐CAYC‐Gold Complexes: Harnessing Weak Secondary Intramolecular Au···H−C(sp3 ) Interactions in Gold(I) Catalysis
Source: Angew Chem Int Ed Engl. 2026 Mar 15;65(17):e26091. doi: 10.1002/anie.202526091 (PMC12994864; doi:10.1002/anie.202526091)
Supplement: Supplementary file 1 — Supporting File1: anie71581‐sup‐0001‐SuppMat.pdf. [file ANIE-65-e26091-s001.pdf]

Supporting Information

Bridged Pyrroloindole-CAYC-Gold Complexes: Harnessing Weak Secondary Intramolecular Au···H−C(*sp*^{3}) Interactions in Gold(I) Catalysis

Sourav Sekhar Bera,*[a,b] Anurag Kumar,[a] Pengcheng Gao,[a] Sanghamitra Das,[c] Roger Lalancette,[a] Roman Szostak,[d] and Michal Szostak*[a]

[a] *Department of Chemistry, Rutgers University, 73 Warren Street, Newark, NJ 07102, USA*

[b] *Department of Chemistry, The Scripps Research Institute, La Jolla, California 92037, USA*

[c] *Department of Chemistry, IIT Kharagpur, Kharagpur, W.B. India, 721302*

[d] *Department of Chemistry, Wroclaw University, F. Joliot-Curie 14, Wroclaw 50-383, Poland*

sbera@scripps.edu; michal.szostak@rutgers.edu

Table of Contents

1. General Information S3

2. Experimental Procedures and Characterization Data S4

- 2.1. General Procedure for Substituted Aniline Synthesis S4
- 2.2. General Procedure for Pyrrole Synthesis S5
- 2.3. General Procedure for 9*H*-pyrrolo[1,2-*a*]indole Scaffold Synthesis S6
- 2.4. Synthesis of Aryl Sulphoxide S11
- 2.5. General Procedure for the Preparation of Ligands S12
- 2.6. General Procedure for the Synthesis of Au-complexes S15
- 2.7. Synthesis of Selenium-Complex Se-complexes S19
- 2.8. Synthesis of Selenium-Complex Rh-complexes S20
- 2.9. General Procedure for the Hydroamination of Alkynes S20
- 2.10. General Procedure for the Synthesis of 1,2-dihydroquinoline derivatives S22
- 2.11. Comparative Catalysis Study S25

3. Crystallographic Studies S27

- 3.1. Crystal Data and Structure Refinement Summaries S28
- 3.2. ORTEP Structures of Au-Complexes S32
- 3.3. X-Ray Crystallographic Comparison S39
- 3.4. Topographical Steric Maps from Crystallographic Data S39
- 3.5. The Torsional Angle Between Carbene Plane and Analogous C-H Plane S40

4. NMR Studies S41

- 4.1. C−H···Au(I) Interactions in Solution via NMR Spectroscopy Study S41
- 4.2. ^{1}H NMR Analysis of Cationic Au-complex S43
- 4.3. Measurement of $^{1}J_{CH}$ coupling constant in complex **8a** S44
- 4.4. Measurement of $^{1}J_{CH}$ coupling constant in complex **9a-[Au]** S46
- 4.5. Variable Temperature Experiment S47

5. Computational Methods S48

- 5.1. AIM, NBO and NCI Analysis S49
- 5.2. HOMO-LUMO Orbitals and Corresponding Energy Levels S51
- 5.3. Topographical Steric Maps from DFT Calculations S54

6. References S56

7. ^{1}H and ^{13}C NMR Spectra S57

8. Cartesian Coordinates with Zero-Point Energies and Thermal Corrections S99

1. General Information

All starting materials reported in the manuscript have been previously described in the literature and prepared by the method reported previously unless stated otherwise. All experiments were performed using standard Schlenk techniques under nitrogen or argon unless stated otherwise. All solvents were purchased at the highest commercial grade and used as received or after purification by passing through activated alumina columns or distillation from sodium/benzophenone under nitrogen. All solvents were deoxygenated prior to use. All other chemicals were purchased at the highest commercial grade and used as received. Reaction glassware was oven-dried at 140 °C for at least 24 h or flame-dried prior to use, allowed to cool under vacuum, and purged with argon (three cycles). All products were identified using ^{1}H NMR analysis and comparison with authentic samples. GC and/or GC/MS analysis was used for volatile products. All yields refer to yields determined by ^{1}H NMR and/or GC or GC/MS using an internal standard (optimization) and isolated yields (preparative runs) unless stated otherwise. ^{1}H NMR and ^{13}C NMR spectra were recorded in $CDCl_3$ on Bruker spectrometers at 500 (^{1}H NMR) and 125 MHz (^{13}C NMR). All shifts are reported in parts per million (ppm) relative to the residual $CHCl_3$ peak (7.26 and 77.2 ppm, ^{1}H NMR and ^{13}C NMR, respectively). All coupling constants (J) are reported in hertz (Hz). Abbreviations are: s, singlet; d, doublet; t, triplet; q, quartet; brs, broad singlet. GC-MS chromatography was performed using Agilent HP6890 GC System and Agilent 5973A inert XL EI/CI MSD using helium as the carrier gas at a flow rate of 1 mL/min and an initial oven temperature of 50 °C. The injector temperature was 250 °C. The detector temperature was 250 °C. For runs with the initial oven temperature of 50 °C, temperature was increased with a 10 °C/min ramp after 50 °C hold for 3 min to a final temperature of 220 °C, then hold at 220 °C for 15 min (splitless mode of injection, total run time of 22.0 min). High-resolution mass spectra (HRMS) were measured on a 7T Bruker Daltonics FT-MS instrument. All flash chromatography was performed using silica gel, 60 Å, 300 mesh. TLC analysis was carried out on glass plates coated with silica gel 60 F254, 0.2 mm thickness. The plates were visualized using a 254 nm UV lamp or aqueous potassium permanganate. ^{1}H NMR and ^{13}C NMR data are given for all compounds in the Supporting Experimental for characterization purposes. ^{1}H NMR, ^{13}C NMR, and HRMS data are given for all new compounds. All products have been previously reported, unless stated otherwise.

2. Experimental Procedures and Characterization Data

General Procedures for the Synthesis of Bridged Cyclic Amino(ylide)Carbenes precursor (8)

2.1a. 2-Benzhydryl-6-bromo-4-methylaniline (3a)

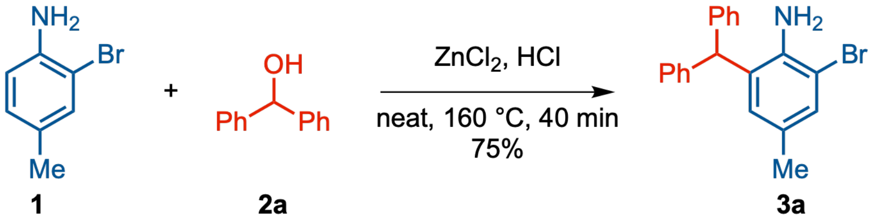

2-Benzhydryl-6-bromo-4-methylaniline **3a** was synthesized according to a modified route from the previous literature procedure.[1] In an oven-dried 100 mL pressure tube, 2-Bromo-4-methylaniline **1** (13.02 g, 70 mmol, 1.0 equiv) and diphenylmethanol (12.90 g, 70 mmol, 1.0 equiv) were charged with a stir bar and placed in an oil bath at 100 °C. After that, anhydrous $ZnCl_2$ (4.77 g, 35 mmol, 0.5 equiv) and HCl (aq., 36%, 6 mL, 1.0 equiv) were added respectively into the melt, and the reaction was heated at 160 °C for 40 minutes. The reaction was cooled down to room temperature, the solid was dissolved in 200 mL of CH_2Cl_2, and washed sequentially with saturated $NaHCO_3$ solution and brine. After drying over anhydrous Na_2SO_4, the solution was evaporated to dryness and subjected to silica gel column chromatography using 2-5% DCM/Hexane as eluent to offer 2-benzhydryl-6-bromo-4-methylaniline **3a** as a colorless powder in 75% (18.50 g) yield.

^{1}H NMR (500 MHz, $CDCl_3$) δ 7.30 (t, J = 7.3 Hz, 4H), 7.26 – 7.23 (m, 2H), 7.18 (s, 1H), 7.11 (d, J = 7.5 Hz, 4H), 6.40 (s, 1H), 5.46 (s, 1H), 3.78 (brs, 2H), 2.11 (s, 3H). **^{13}C NMR** (126 MHz, $CDCl_3$) δ 142.1 (C_q-Ar), 139.6 (C_q-Ar), 131.1 (CH-Ar), 130.5 (C_q-Ar), 130.0 (CH-Ar), 129.6 (CH-Ar), 128.8 (CH-Ar), 128.5 (C_q-Me), 127.0 (CH-Ar), 111.0 (C_q-Ar), 53.1 (CH-Ph_2), 20.5 (CH_3). HRMS (ESI): calcd for $C_{20}H_{19}BrN$ $[M + H]^+$ 352.0695, found 352.0700.

2.1b. 2-bromo-4-methyl-6-(1-phenylethyl)aniline (3b)

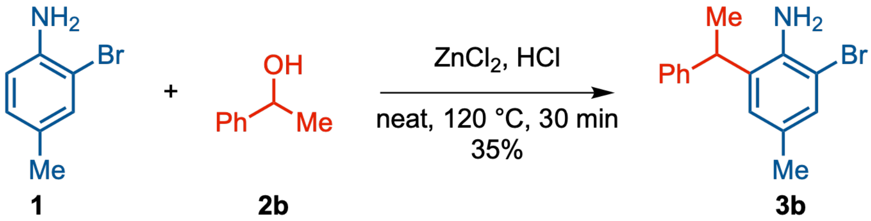

In an oven-dried 100 mL pressure tube, 2-Bromo-4-methylaniline **1** (2.79 g, 15 mmol, 1.0 equiv) and 1-phenylethan-1-ol (1.83 g, 15 mmol, 1.0 equiv) were charged with a stir bar and placed in an oil bath at 60 °C. After that, anhydrous $ZnCl_2$ (1 g, 7.5 mmol, 0.5 equiv) and HCl (aq., 36%, 1.3 mL, 1.0 equiv) were added respectively into the melt, and the reaction was heated at 120 °C for 30 minutes. The reaction was cooled down to room temperature, the solid was dissolved in 50 mL of CH_2Cl_2 and washed sequentially with saturated $NaHCO_3$ solution and brine. After drying over anhydrous Na_2SO_4, the solution was evaporated to dryness and subjected to silica gel column chromatography using 2-5% DCM/Hexane as eluent to offer 2-bromo-4-methyl-6-(1-phenylethyl)aniline **3b** as a colorless powder in 35% (1.52 g) yield.

^{1}H NMR (500 MHz, $CDCl_3$) δ 7.29 (t, J = 7.5 Hz, 2H), 7.22 – 7.18 (m, 4H), 7.04 (s, 1H), 4.07 (q, J = 7.3 Hz, 1H), 3.79 (brs, 2H), 2.29 (s, 3H), 1.61 (d, J = 7.1 Hz, 3H). **^{13}C NMR** (126 MHz, $CDCl_3$) δ 145.1 (C_q-Ar), 139.7 (C_q-Ar), 131.2 (C_q-Ar), 130.9 (CH-Ar), 129.0 (CH-Ar), 128.5 (C_q-Ar), 127.6 (CH-Ar), 127.4 (CH-Ar), 126.8 (CH-Ar), 111.0 (C_q-Ar), 41.3 (CH-(PhMe)), 22.1 (CH_3), 20.6 (CH_3). HRMS (ESI): calcd for $C_{15}H_{17}BrN$ $[M + H]^+$ 290.0539, found 290.0544.

2.2. General Procedure A for Pyrrole Synthesis

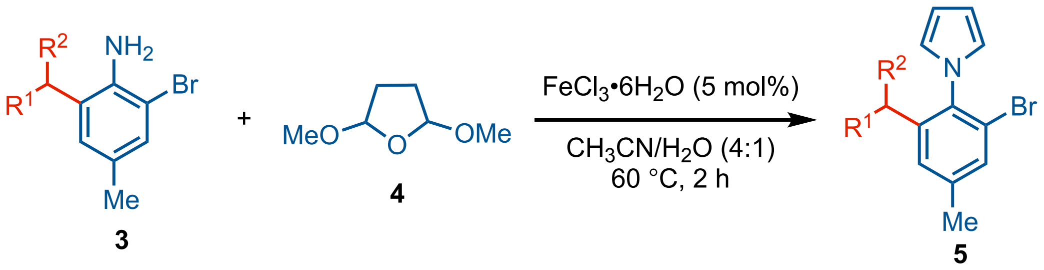

Compound **5** was synthesized according to a modified route from the previous literature procedure.[2] In an oven-dried 250 mL round-bottom flask, aniline **3** (1 equiv), 2,5 dimethoxytetrahydrofuran (1.2 equiv) and $FeCl_3•6H_2O$ (5 mol%) were added together and dissolved with acetonitrile/water (4:1) solution (0.5 M). The mixture was placed in an oil bath and stirred at 60 °C for 2 h. After completion of the reaction, the solvent was evaporated under reduced pressure and dissolved again in DCM. The organic layer was extracted with H_2O, brine and dried over Na_2SO_4. The mixture was evaporated under reduced pressure to get the solid product **5** and was used for the next step without further purification.

2.2a. 1-(2-Benzhydryl-6-bromo-4-methylphenyl)-1H-pyrrole (5a)

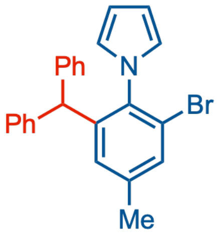

5a

Prepared from 2-benzhydryl-6-bromo-4-methylaniline **3a** (17.6 g, 50 mmol), 2,5 dimethoxytetrahydrofuran (7.93 g, 60 mmol) and $FeCl_3 \cdot 6H_2O$ (0.675 g, 5 mol%) in 200 mL of acetonitrile/water (4:1) solution following General Procedure A. The mixture was evaporated under reduced pressure to get the colorless solid product 1-(2-benzhydryl-6-bromo-4-methylphenyl)-1H-pyrrole **5a** (19.7 g, 98%).

^{1}H NMR (500 MHz, $CDCl_3$) δ 7.42 (s, 1H), 7.29 – 7.26 (m, 4H), 7.24 – 7.21 (m, 2H), 7.00 – 6.99 (m, 4H), 6.93 (s, 1H), 6.45 – 6.44 (m, 2H), 6.29 – 6.28 (m, 2H), 5.20 (s, 1H), 2.33 (s, 3H). **^{13}C NMR** (126 MHz, $CDCl_3$) δ 144.2 (C_q-Ar), 143.2 (C_q-Ar), 139.8 (C_q-Ar), 137.2 (C_q-Ar), 131.8 (CH-Ar), 130.4 (CH-Ar), 129.4 (CH-Ar), 128.4 (CH-Ar), 126.6 (CH-Ar), 123.7 (C_q-Ar), 122.4 (CH-Ar), 109.0 (CH-Ar), 51.6 (CH-(Ph_2)), 21.3 (CH_3). HRMS (ESI): calcd for $C_{24}H_{21}BrN$ $[M + H]^+$ 402.0852, found 402.0849.

2.2b. 1-(2,6-dibromo-4-methylphenyl)-1*H*-pyrrole (5d)

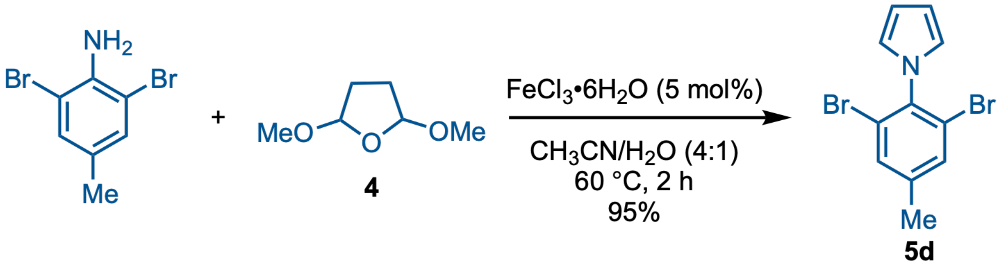

Compound **5d** was synthesized starting from commercially available 2,6-dibromo-4-methylaniline using general procedure A on a 30 mmol scale. The mixture was evaporated under reduced pressure. Yield = 95% (9.00 g). **^{1}H NMR** (500 MHz, $CDCl_3$) δ 7.46 (s, 2H), 6.67 (t, J = 2.1 Hz, 2H), 6.37 (t, J = 2.1 Hz, 2H), 2.39 (s, 3H). **^{13}C NMR** (126 MHz, $CDCl_3$) δ 141.4 (C_q-Ar), 137.2 (C_q-Ar), 132.9 (CH-Ar), 123.7 (C_q-Ar), 121.8 (CH-Ar), 109.2 (CH-Ar), 20.7 (CH_3).

2.3. General Procedure B for the Synthesis of 9*H*-Pyrrolo[1,2-*a*]indole scaffold

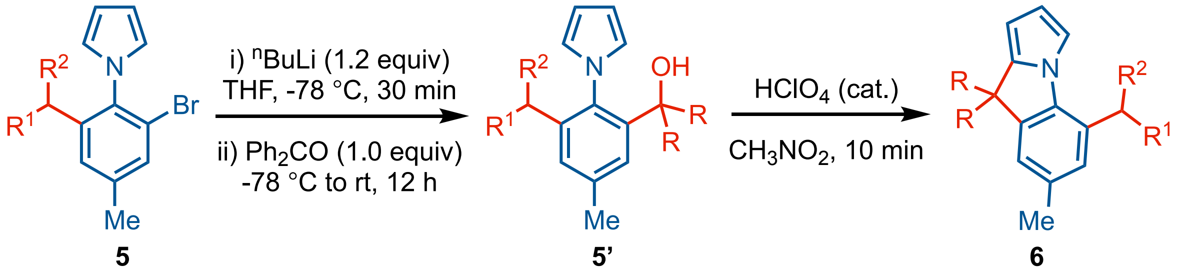

To a flame-dried two-neck round-bottom flask, **5** (1.0 equiv) was added in 120 mL of dry THF under an inert atmosphere. The solution was then cooled to -78 °C, and n-butyllithium (2.5 M hexane solution, 1.2 equiv) was added dropwise over 15 minutes. The reaction mixture was stirred for an additional 30 minutes at -78 °C, before adding a solution of benzophenone (1.0 equiv) in 30 mL of THF. The reaction mixture was allowed to warm to room temperature and stirred for 12 h. The reaction mixture was quenched with water and extracted with dichloromethane three times. The combined organic layer was washed with 100 mL of brine twice. The solution was dried over Na_2SO_4 and evaporated under reduced pressure to get the product, which was directly used for the next step.

The previous reaction mixture was taken in a 250 mL round-bottom flask and dissolved with 100 mL of nitromethane, CH_3NO_2, as solvent. After that, a catalytic amount of perchloric acid was added dropwise to the mixture, and precipitation of the product started forming after a few minutes. After 10 minutes, the precipitated product was filtered off and washed with CH_3NO_2 three times to get the pure cyclized product **6**.

2.3a. 5-Benzhydryl-7-methyl-9,9-diphenyl-9H-pyrrolo[1,2-a]indole (6a)

Compounds **5a'** and **6** were synthesized using the general procedure B. Compound **5a** (18.1 g, 45 mmol) was added in 120 mL of dry THF under an inert atmosphere. The solution was then cooled to -78 °C, and n-butyllithium (2.5 M hexane solution, 21.6 mL, 54 mmol, 1.2 equiv) was added dropwise over 15 minutes. The reaction mixture was stirred for an additional 30 minutes at -78 °C before adding a solution of benzophenone (8.2 g, 45 mmol, 1.0 equiv) in 30 mL of THF. After that, general procedure B was followed to get the product **5a'**, which was used for the next step without further purification.

One small portion of the mixture was purified by silica gel column chromatography using DCM/Hexane (5:95) as eluent to offer colorless solid (3-benzhydryl-5-methyl-2-(1H-pyrrol-1-yl)phenyl)diphenylmethanol **5a'**.

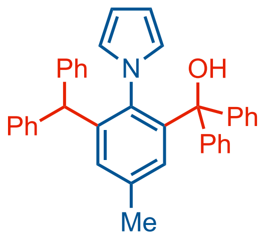

5a'

^{1}H NMR (500 MHz, $CDCl_3$) δ 7.28 – 7.25 (m, 6H), 7.23 – 7.20 (m, 4H), 7.18 (d, J = 7.1 Hz, 2H), 7.15 – 7.14 (m, 4H), 6.91 – 6.89 (m, 5H), 6.45 (d, J = 1.5 Hz, 1H), 6.00 (t, J = 2.1 Hz, 2H), 5.91 (t, J = 2.1 Hz, 2H), 4.64 (s, 1H), 2.94 (s, 1H), 2.16 (s, 3H). **^{13}C NMR** (126 MHz, $CDCl_3$) δ 146.9 (C_q-Ar), 144.8 (C_q-Ar), 144.5 (C_q-Ar), 143.9 (C_q-Ar), 137.2 (C_q-Ar), 136.3 (C_q-Ar), 130.8 (CH-Ar), 130.1 (CH-Ar), 129.4 (CH-Ar), 128.2 (CH-Ar), 128.0 (CH-Ar), 127.9 (CH-Ar), 127.4 (CH-Ar), 126.4 (CH-Ar), 123.9 (CH-Ar), 109.4 (CH-Ar), 83.0 (C_q-

(Ph2(OH))), 50.1 (C_q-(Ph2)), 21.8 (CH_3). HRMS (ESI): calcd for $C_{37}H_{32}NO$ $[M + H]^+$ 506.2478, found 506.2474.

The previous reaction mixture was taken in a 250 mL round-bottom flask and dissolved with 80 mL of nitromethane. After that, perchloric acid (0.5 mL) was added dropwise to the mixture, and precipitation of the product started forming after a few minutes. After 10 minutes, the precipitated product was filtered off and washed with CH_3NO_2 (15 mL x 3) three times to get the pure cyclized product **6a**.

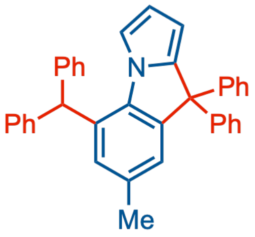

6a

Yield = 70% (15.4 g) over two-step, **^{1}H NMR** (500 MHz, $CDCl_3$) δ 7.32 (t, J = 7.4 Hz, 4H), 7.27 – 7.21 (m, 12H), 7.14 (d, J = 7.3 Hz, 4H), 7.09 (s, 1H), 7.01 (d, J = 2.2 Hz, 1H), 6.55 (s, 1H), 6.24 (t, J = 3.1 Hz, 1H), 6.12 (s, 1H), 6.05 (d, J = 3.1 Hz, 1H), 2.21 (s, 3H). **^{13}C NMR** (126 MHz, $CDCl_3$) δ 146.4 (C_q-Ar), 143.8 (C_q-Ar), 142.8 (C_q-Ar), 142.6 (C_q-Ar), 136.4 (C_q-Ar), 133.0 (C_q-Ar), 130.4 (CH-Ar), 129.7 (CH-Ar), 128.7 (CH-Ar), 128.4 (CH-Ar), 128.2 (CH-Ar), 127.3 (C_q-Ar), 126.8 (CH-Ar), 126.7 (CH-Ar), 126.4 (CH-Ar), 113.8 (CH-Ar), 113.3 (CH-Ar), 103.0 (CH-Ar), 58.7 (C_q-(Ph2)), 52.0 (C_q-(Ph2)), 21.6 (CH_3). HRMS (ESI): calcd for $C_{37}H_{30}N$ $[M + H]^+$ 488.2373, found 488.2361.

2.3b. 7-methyl-9,9-diphenyl-5-(1-phenylethyl)-9H-pyrrolo[1,2-a]indole (6b)

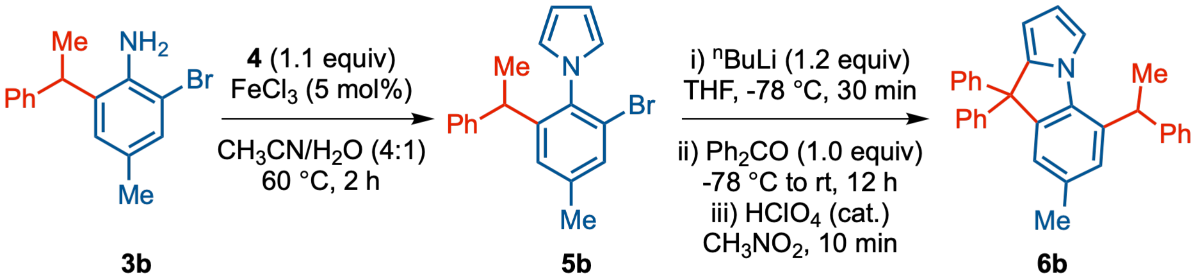

Compound **6b** was synthesized according to the general procedures A and B from compound **3b** on a 5.0 mmol scale.

Yield = 62% (1.32 g) over two steps. **^{1}H NMR** (500 MHz, $CDCl_3$) δ 7.34 – 7.29 (m, 4H), 7.27 – 7.26 (d, J = 5.7 Hz, 4H), 7.25 – 7.20 (m, 7H), 7.16 (dd, J = 2.8, 0.9 Hz, 1H), 7.07 (d, J = 0.9 Hz, 1H), 6.92 (s, 1H), 6.33 (d, J = 3.0 Hz, 1H), 6.08 (dd, J = 3.5, 1.0 Hz, 1H), 4.81 (q, J = 7.2 Hz, 1H), 2.30 (s, 3H), 1.75 (d, J = 7.2 Hz, 3H). **^{13}C NMR** (126 MHz, $CDCl_3$) δ 146.5 (C_q-Ar), 144.8 (C_q-Ar), 144.1 (C_q-Ar), 142.7 (C_q-Ar), 136.0 (C_q-Ar), 133.2 (C_q-Ar), 129.8 (C_q-Ar), 128.8 (CH-Ar), 128.4 (CH-Ar), 128.2 (CH-Ar), 128.2 (CH-Ar), 128.0 (CH-Ar), 127.9 (CH-Ar), 126.7 (CH-Ar), 126.7 (CH-Ar), 126.5 (CH-Ar), 126.1 (CH-Ar), 113.6 (CH-Ar), 113.4

(CH-Ar), 103.0 (CH-Ar), 58.7 (C_q-(Ph_2)), 39.5 (CH-(PhMe)), 22.1 (CH_3), 21.6 (CH_3). HRMS (ESI): calcd for $C_{32}H_{28}BrN$ $[M + H]^+$ 426.2216, found 426.2226.

2.3c. 5-benzhydryl-7,9,9-trimethyl-9H-pyrrolo[1,2-a]indole (6c)

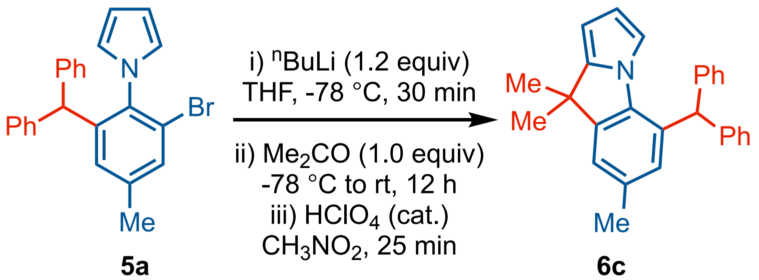

Compound **6c** was synthesized using the general procedure B, starting from compound **5a** and using acetone as a ketone on a 10 mmol scale. The perchloric acid cyclization step was run for 25 minutes and cooled at 0 °C to get the pure solid product.

Yield = 66% yield (2.4 g). **^{1}H NMR** (500 MHz, $CDCl_3$) δ 7.31 (t, J = 7.4 Hz, 4H), 7.24 (t, J = 7.3 Hz, 2H), 7.13 (d, J = 7.3 Hz, 4H), 7.04 (s, 1H), 6.96 (d, J = 2.1 Hz, 1H), 6.53 (s, 1H), 6.22 (t, J = 3.1 Hz, 1H), 6.08 (s, 1H), 5.99 (dd, J = 3.3, 0.7 Hz, 1H), 2.27 (s, 3H), 1.50 (s, 6H). **^{13}C NMR** (126 MHz, $CDCl_3$) δ 147.0 (C_q-Ar), 146.4 (C_q-Ar), 142.9 (C_q-Ar), 136.0 (C_q-Ar), 132.8 (C_q-Ar), 129.7 (CH-Ar), 129.7 (CH-Ar), 128.7 (CH-Ar), 127.0 (C_q-Ar), 126.8 (CH-Ar), 122.4 (CH-Ar), 113.2 (CH-Ar), 112.5 (CH-Ar), 98.6 (CH-Ar), 51.9 (CH-(Ph_2)), 40.5 (C_q-(Me_2)), 28.6 (CH_3), 21.5 (CH_3). HRMS (ESI): calcd for $C_{27}H_{26}N$ $[M + H]^+$ 364.2060, found 364.2070.

2.3d. 5-bromo-7-methyl-9,9-diphenyl-9*H*-pyrrolo[1,2-*a*]indole (5da)

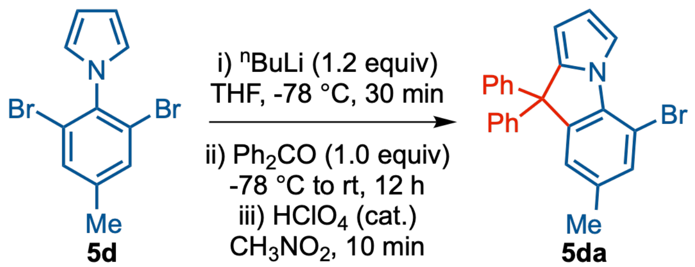

Compound **5da** was synthesized using Compound **5d** (28.5 mmol) following the general procedure B. The perchloric acid cyclization step was run for 10 minutes and cooled at 0 °C to get the pure solid product. Yield = 64% (7.3 g). **^{1}H NMR** (500 MHz, $CDCl_3$) δ 7.74 (d, J = 3.4 Hz, 1H), 7.24 – 7.19 (m, 11H), 7.09 (s, 1H), 6.39 (t, J = 3.2 Hz, 1H), 6.12 (d, J = 2.1 Hz, 1H), 2.29 (s, 3H). **^{13}C NMR** (126 MHz, $CDCl_3$) δ 145.7 (C_q-Ar), 144.5 (C_q-Ar), 143.6 (C_q-Ar), 136.2 (C_q-Ar), 134.6 (C_q-Ar), 132.7 (CH-Ar), 128.5 (CH-Ar), 128.1 (CH-Ar), 127.1 (CH-Ar),

126.9 (CH-Ar), 113.5 (CH-Ar), 113.2 (CH-Ar), 104.2 (C_q-Ar), 103.8 (CH-Ar), 59.2 (C_q-(Ph_2)), 21.0 (CH_3). HRMS (ESI): calcd for $C_{24}H_{19}BrN$ $[M + H]^+$ 400.0695, found 400.0691.

2.3e. 5-isopropyl-7-methyl-9,9-diphenyl-9*H*-pyrrolo[1,2-*a*]indole (6d)

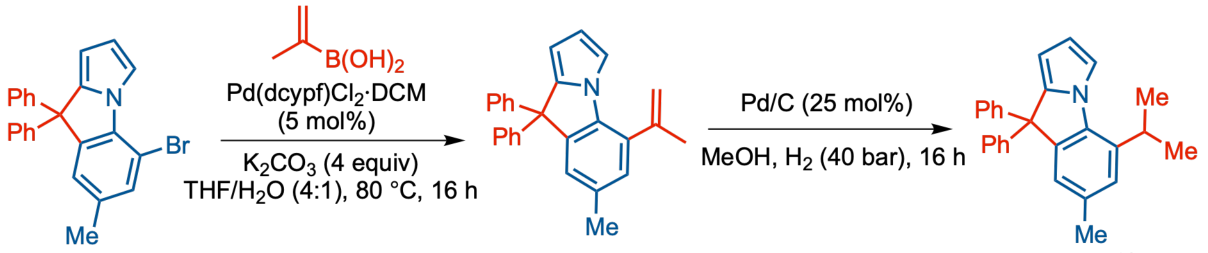

5db was synthesized according to the previous literature procedure.[3] In a round-bottom flask purged with argon, compound **5da** (1.0 g, 2.5 mmol, 1.0 equiv), prop-1-en-2-ylboronic acid (0.5 g, 3.00 mmol, 1.2 equiv) in THF/H_2O (4:1, 15 mL), Pd(dcypf)Cl_2·DCM (102 mg, 0.125 mmol, 5 mol%), K_2CO_3 (1.38 g, 10 mmol, 4 equiv) was added. The solution was degassed by argon bubbling for 30 min. After connecting the condenser to the flask, the reaction mixture was heated at 80 °C in an oil bath for 16 h. Upon completion by TLC, the crude mixture was filtered through a celite pad with ethyl acetate (10 mL x 3). The filtrate was washed with H_2O (10 mL × 3) and brine (10 mL × 3). The combined organic layers were dried over anhydrous Na_2SO_4, concentrated under reduced pressure, filtered, and concentrated in vaccuo. The resulting residue was purified via silica gel flash column chromatography using an ethyl acetate/hexane solvent system (5:95) to yield the desired product **5db**. Yield = 80% (725 mg). **^{1}H NMR** (500 MHz, $CDCl_3$) δ 7.30 – 7.25 (m, 8H), 7.24 – 7.20 (m, 3H), 7.10 (s, 1H), 6.89 (s, 1H), 6.33 (t, *J* = 3.1 Hz, 1H), 6.10 (d, *J* = 2.7 Hz, 1H), 5.39 (s, 1H), 5.16 (s, 1H), 2.33 (s, 3H), 2.19 (s, 3H). **^{13}C NMR** (126 MHz, $CDCl_3$) δ 146.3 (C_q-Ar), 143.7 (C_q-Ar), 142.6 (C_q-Ar), 142.5 (C_q-Ar), 134.1 (C_q-Ar), 132.9 (C_q-Ar), 128.8 (CH-Ar), 128.5 (CH-Ar), 128.4 (CH-Ar), 128.2 (C_q-Ar), 126.7 (CH-Ar), 126.6 (CH-Ar), 116.8 (CH_2-Ar), 112.9 (CH-Ar), 112.9 (CH-Ar), 103.2 (CH-Ar), 58.8 (C_q-(Ph_2)), 24.6 (CH_3), 21.3 (CH_3). HRMS (ESI): calcd for $C_{27}H_{24}N$ $[M + H]^+$ 362.1903, found 362.1910.

Compound **5db** (0.36 g, 1.0 mmol) and Pd/C (25 mol%) were taken in a parr apparatus vial and anhydrous methanol was (3 mL) added to it. The resulting suspension was put into a high-pressure hydrogenation apparatus, which was charged to 40 bar with hydrogen. After 12 h, the pressure was released. The reaction mixture was filtered through a pad of celite, and filtrate concentrated in vacuo. Flash chromatography, using a gradient of ethyl acetate-hexanes (1%, then 5%, then 10%) as eluant, afforded 0.22 g (61% yield) of **6d** as a clear colorless oil.

^{1}H NMR (500 MHz, $CDCl_3$) δ 7.25 – 7.24 (m, 8H), 7.23 – 7.18 (m, 3H), 7.03 (s, 1H), 7.02 (s, 1H), 6.40 (t, J = 3.1 Hz, 1H), 6.11 (dd, J = 3.4, 0.9 Hz, 1H), 3.57 (hept, J = 6.9 Hz, 1H), 2.33 (s, 3H), 1.39 (d, J = 6.8 Hz, 6H). **^{13}C NMR** (126 MHz, $CDCl_3$) δ 146.5 (C_q-Ar), 144.1 (C_q-Ar), 142.3 (C_q-Ar), 135.5 (C_q-Ar), 133.3 (C_q-Ar), 132.5 (C_q-Ar), 128.3 (CH-Ar), 128.2 (CH-Ar), 126.7 (CH-Ar), 125.5 (CH-Ar), 125.5 (CH-Ar), 113.4 (CH-Ar), 113.3 (CH-Ar), 102.9 (CH-Ar), 58.7 (C_q-(Ph_2)), 28.4 (CH-(Me_2)), 22.9 (CH_3), 21.6 (CH_3). HRMS (ESI): calcd for $C_{27}H_{26}N$ $[M + H]^+$ 364.2060, found 364.2064.

2.4. General Procedure C for Preparation of Aryl Sulphoxide 7

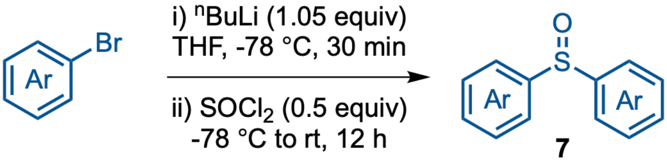

Sulphoxides 7 were synthesized according to the previous literature procedure.[4] To a flame-dried 500 mL of two-neck round-bottom flask, aryl bromide (100 mmol, 1 equiv) was added in 200 mL of dry THF under an inert atmosphere. The solution was then cooled to -78 °C and n-butyllithium (2.5 M hexanes solution, 42 mL, 105 mmol, 1.05 equiv) was added dropwise over 15 minutes. The reaction mixture was stirred for an additional 30 minutes at -78 °C before adding a solution of $SOCl_2$ (5.95 g, 50 mmol, 0.5 equiv) in 30 mL of THF. The reaction mixture was allowed to warm to room temperature and stirred for 12 h. The reaction mixture was quenched with water and extracted with dichloromethane three times. The combined organic layer was washed with 100 mL of brine twice. The solution was dried over Na_2SO_4 and was evaporated under reduced pressure. After evaporation of the solvent, the crude product was purified by silica gel column chromatography using DCM/Hexane (1:4) as eluent.

2.4a. 1,1'-sulfinyldinaphthalene (7a)

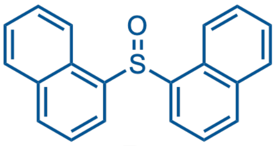

7a

Compound **7a** was synthesized using the General Procedure C. Yield = 30% (9.0 g). **^{1}H NMR** (500 MHz, $CDCl_3$) δ 8.36 – 8.34 (m, 2H), 8.05 (d, J = 7.3 Hz, 2H), 7.95 (d, J = 8.2 Hz, 2H), 7.91 – 7.89 (m, 2H), 7.57 (t, J = 7.8 Hz, 2H), 7.54 – 7.54 (m, 4H). **^{13}C NMR** (126 MHz, $CDCl_3$) δ 139.9 (C_q-Ar), 133.8 (C_q-Ar), 132.1 (CH-Ar), 130.1 (C_q-Ar), 129.0 (CH-Ar), 127.7 (CH-Ar), 126.8 (CH-Ar), 125.73 (CH-Ar), 125.72 (CH-Ar), 122.7 (CH-Ar).

2.4b. 2,2'-sulfinyldinaphthalene (7b)

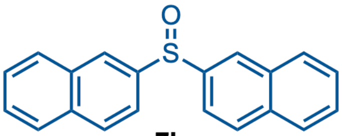

7b

Yield = 48% (14.5 g). **^{1}H NMR** (500 MHz, $CDCl_3$) δ 8.37 (s, 2H), 7.97 – 7.96 (m, 2H), 7.86 – 7.83 (m, 4H), 7.58 – 7.57 (m, 4H), 7.53 – 7.51 (m, 2H). **^{13}C NMR** (126 MHz, $CDCl_3$) δ 142.5 (C_q-Ar), 134.5 (C_q-Ar), 132.9 (C_q-Ar), 129.9 (CH-Ar), 128.8 (CH-Ar), 128.2 (CH-Ar), 128.1 (CH-Ar), 127.4 (CH-Ar), 125.7 (CH-Ar), 121.0 (CH-Ar).

2.5. General Procedure D for the Preparation of Ligands (8a-8f)

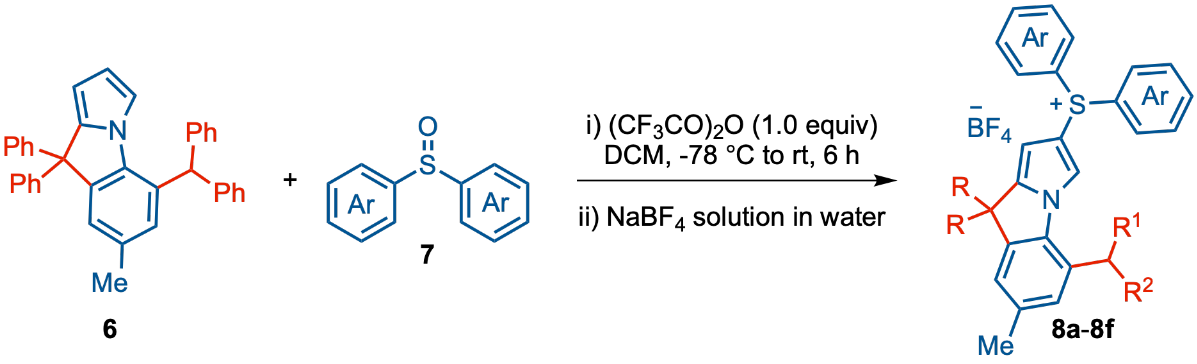

An oven-dried round-bottom flask equipped with a stir bar was charged with **6** (3 mmol, 1 equiv) and aryl sulphoxide **7** (6 mmol, 2.0 equiv). The mixture was dissolved in 12 mL dichloromethane (DCM) and cooled to -78 °C. After that, trifluoro acetic anhydride (6 mmol, 2.0 equiv) was dissolved in 3 mL of DCM and dropwise added to the mixture over 15 minutes. The mixture was then slowly allowed to come at room temperature. After reaching at room temperature, the mixture was shaken with a saturated solution of sodium tetrafluoroborate (10 mL) and the aqueous phase extracted with CH_2Cl_2 (2 x 10 mL). The combined organic layers were dried over Na_2SO_4 and evaporated under reduced pressure. The residue was further recrystalized in DCM/hexane sovent to get the pure product.

2.5a. (5-benzhydryl-7-methyl-9,9-diphenyl-9H-pyrrolo[1,2-a]indol-2-yl)diphenylsulfonium tetrafluoroborate (8a)

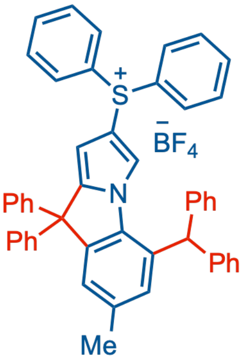

8a

Compound **8a** was synthesized using the general procedure D on a 3.0 mmol scale. Yield = 74% (1.68 g). **^{1}H NMR** (500 MHz, $CDCl_3$) δ 7.65 – 6.63 (m, 2H), 6.61 (s, 1H), 7.58 – 7.56 (m, 7H), 7.26 – 7.22 (m, 11H), 7.20 – 7.17 (m, 2H), 7.11 (d, J = 6.9 Hz, 4H), 7.06 – 7.04 (m, 5H), 6.57 (s, 1H), 6.31 (s, 1H), 6.01 (s, 1H), 2.20 (s, 3H). **^{13}C NMR** (126 MHz, $CDCl_3$) δ 148.3 (C_q-pyrrole), 144.3 (C_q-Ar), 142.3 (C_q-Ar), 141.7 (C_q-Ar), 136.7 (C_q-Ar), 134.3 (C_q-Ar), 133.9 (CH-Ar), 131.3 (CH-Ar), 130.3 (CH-Ar), 130.2 (C_q-Ar), 129.8 (CH-Ar), 128.93 (CH-Ar), 128.89 (CH-

Ar), 127.9 (CH-Ar), 127.64 (C_q-Ar), 127.60 (CH-Ar), 127.1 (CH-Ar), 126.5 (CH-Ar), 122.5 (CH-precarbene), 104.2 (CH-pyrrole), 103.3 (C_q-pyrrole), 59.6 (C_q-(Ph_2)), 51.9 (CH-(Ph_2)), 21.8 (CH_3). HRMS (ESI): calcd for $C_{49}H_{38}NS^+$ [M^+ - BF_4^-] 672.2719, found 672.2701.

2.5b. (7-methyl-9,9-diphenyl-5-(1-phenylethyl)-9H-pyrrolo[1,2-a]indol-2-yl)diphenylsulfonium tetrafluoroborate (8b)

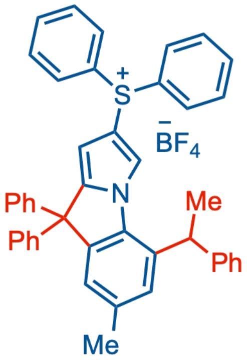

8b

Compound **8b** was synthesized using the general procedure D on a 0.5 mmol scale. Yield = 61% (212.5 mg). **^{1}H NMR** (500 MHz, $CDCl_3$) δ 7.92 – 7.90 (m, 1H), 7.68 – 7.63 (m, 4H), 7.61 – 7.58 (m, 6H), 7.27 – 7.23 (m, 5H), 7.21 – 7.20 (m, 3H), 7.17 – 7.09 (m, 9H), 6.32 – 6.31 (m, 1H), 4.76 (q, J = 6.8 Hz, 1H) 2.39 (s, 3H), 1.72 (d, J = 7.0 Hz, 3H). **^{13}C NMR** (126 MHz, $CDCl_3$) δ 148.4 (C_q-pyrrole), 144.32 (C_q-Ar), 144.25 (C_q-Ar), 144.23 (C_q-Ar), 142.17 (C_q-Ar), 142.15 (C_q-Ar), 136.8 (C_q-Ar), 134.01 (C_q-Ar), 133.95 (C_q-Ar), 133.92 (CH-Ar), 133.87 (CH-Ar), 133.8 (CH-Ar), 131.59 (C_q-Ar), 131.57 (C_q-Ar), 131.34 (CH-Ar), 131.32 (CH-Ar), 131.28 (CH-Ar), 131.26 (CH-Ar), 130.3 (CH-Ar), 129.9 (CH-Ar), 129.0 (CH-Ar), 128.8 (CH-Ar), 127.8 (CH-Ar), 127.8 (CH-Ar), 127.73 (CH-Ar), 127.65 (CH-Ar), 127.6 (CH-Ar), 127.5 (CH-Ar), 126.58 (CH-Ar), 126.57 (CH-Ar), 126.22 (CH-Ar), 126.20 (CH-Ar), 122.83 (CH-precarbene), 122.76 (CH-precarbene), 103.99 (CH-pyrrole), 103.95 (CH-pyrrole), 103.12 (C_q-(SPh_2)), 103.09 (C_q-pyrrole), 59.5 (C_q-(Ph_2)), 39.6 (CH-(PhMe)), 22.7 (CH_3-(CHPh)), 21.8 (CH_3). HRMS (ESI): calcd for $C_{44}H_{36}NS^+$ [M^+ - BF_4^-] 610.2563, found 610.2564.

2.5c. (5-isopropyl-7-methyl-9,9-diphenyl-9*H*-pyrrolo[1,2-*a*]indol-2-yl)diphenylsulfonium tetrafluoroborate (8c)

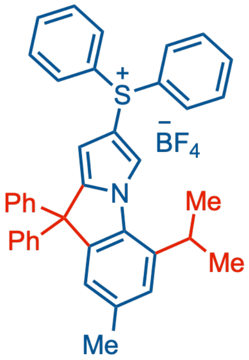

8c

Compound **8c** was synthesized using the general procedure D on a 0.5 mmol scale. Yield = 44% (140 mg). **^{1}H NMR** (500 MHz, $CDCl_3$) δ 8.52 (s, 1H), 7.72 – 7.70 (m, 4H), 7.67 – 7.64 (m, 2H), 7.62 – 6.58 (m, 4H), 7.27 – 7.26 (m, 6H), 7.14 – 7.12 (m, 5H), 6.99 (s, 1H), 6.30 (s, 1H), 3.64 – 6.58 (m, 1H), 2.35 (s, 3H), 1.40 (d, J = 6.9 Hz, 6H). **^{13}C NMR** (126 MHz, $CDCl_3$) δ 149.0 (C_q-pyrrole), 144.4 (C_q-Ar), 141.7 (C_q-Ar), 137.0 (C_q-Ar), 135.6 (C_q-Ar), 133.7 (CH-Ar), 133.3 (C_q-Ar), 131.2 (CH-Ar), 130.1 (CH-Ar), 128.8 (CH-Ar), 128.5 (C_q-Ar), 127.9 (CH-Ar), 127.5 (CH-Ar), 126.7 (CH-Ar), 125.4 (CH-Ar), 124.0 (CH-precarbene), 103.3 (CH-pyrrole), 103.0

(C_q-pyrrole), 59.6 (C_q-(Ph_2)), 28.3 (CH-(Me_2)), 22.6 ($2CH_3$), 21.8 (CH_3). HRMS (ESI): calcd for $C_{39}H_{34}NS^+$ [M^+ - BF_4^-] 548.2406, found 548.2411.

2.5d. (5-benzhydryl-7-methyl-9,9-diphenyl-9*H*-pyrrolo[1,2-*a*]indol-2-yl)di(naphthalen-1-yl)sulfonium tetrafluoroborate (8d)

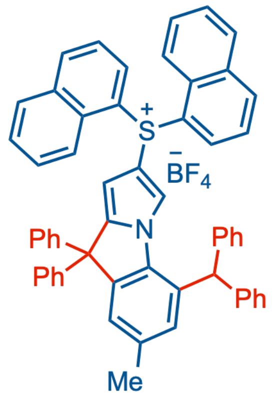

8d

Compound **8d** was synthesized using the general procedure D on a 1.0 mmol scale. Yield = 60% (516 mg). **^{1}H NMR** (500 MHz, $CDCl_3$) δ 8.16 (s, 4H), 7.99 (s, 2H), 7.62 – 7.56 (m, 7H), 7.48 (d, *J* = 6.6 Hz, 2H), 7.26 – 7.25 (m, 6H), 7.13 – 7.01 (m, 15H), 6.55 (s, 1H), 6.41 (s, 1H), 6.03 (s, 1H), 2.19 (s, 3H). **^{13}C NMR** (126 MHz, $CDCl_3$) δ 148.3 (C_q-pyrrole), 144.1 (C_q-Ar), 142.2 (C_q-Ar), 141.5 (C_q-Ar), 136.6 (C_q-Ar), 135.1 (C_q-Ar), 134.4 (C_q-Ar), 134.2 (C_q-Ar), 131.0 (CH-Ar), 130.7 (CH-Ar), 130.1 (CH-Ar), 129.9 (C_q-Ar), 129.8 (CH-Ar), 129.6 (CH-Ar), 129.5 (CH-Ar), 128.8 (CH-Ar), 128.7 (CH-Ar), 128.3 (CH-Ar), 127.7 (CH-Ar), 127.5 (CH-Ar), 126.8 (C_q-Ar), 126.5 (CH-Ar), 126.4 (CH-Ar), 122.9 (CH-precarbene), 122.0 (CH-Ar), 121.5 (CH-Ar), 105.0 (CH-pyrrole), 99.7 (C_q-pyrrole), 59.5 (C_q-(Ph_2)), 51.7 (CH-(Ph_2)), 21.7 (CH_3). HRMS (ESI): calcd for $C_{57}H_{42}NS^+$ [M^+ - BF_4^-] 772.3032, found 772.3014.

2.5e. (5-benzhydryl-7-methyl-9,9-diphenyl-9H-pyrrolo[1,2-a]indol-2-yl)di(naphthalen-2-yl)sulfonium tetrafluoroborate (8e)

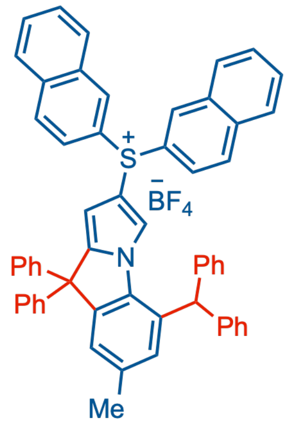

8e

Compound **8e** was synthesized using the general procedure D on a 0.5 mmol scale. Yield = 51% (219 mg). **^{1}H NMR** (500 MHz, $CDCl_3$) δ 8.38 (s, 2H), 8.00 (d, *J* = 8.9 Hz, 2H), 7.94 (d, *J* = 8.2 Hz, 2H), 7.90 (d, *J* = 8.1 Hz, 2H), 7.72 (s, 1H), 7.68 (*t*, J = 7.5 Hz, 2H), 7.62 (t, *J* = 7.5 Hz, 2H), 7.49 (dd, *J* = 8.8, 1.5 Hz, 2H), 7.31 – 7.26 (m, 6H), 7.18 (d, J = 7.1 Hz, 4H), 7.13 – 7.10 (m, 7H), 7.04 (d, J = 7.6 Hz, 4H), 6.60 (s, 1H), 6.42 (s, 1H), 6.07 (s, 1H). 2.23 (s, 3H). **^{13}C NMR** (126 MHz, $CDCl_3$) δ 148.3 (C_q- pyrrole), 144.3 (C_q-Ar), 142.3 (C_q-Ar), 141.7 (C_q-Ar), 136.6 (C_q-Ar), 135.0 (C_q-Ar), 134.3 (C_q-Ar), 133.2 (C_q-Ar), 133.0 (CH-Ar), 131.7 (CH-Ar), 131.3 (CH-Ar), 130.1 (CH-Ar), 129.7 (CH-Ar), 129.5 (CH-Ar), 128.9 (CH-Ar), 128.8 (CH-Ar), 128.6 (CH-Ar), 128.2 (CH-Ar), 127.9 (CH-Ar), 127.6 (CH-Ar), 127.0 (CH-Ar), 126.5 (CH-Ar), 123.9 (C_q-Ar), 123.5 (CH-Ar), 122.4 (CH-precarbene), 104.1 (C_q-pyrrole), 104.0 (CH-pyrrole), 59.6 (C_q-(Ph_2)), 51.8 (CH-(Ph_2)), 21.7 (CH_3). HRMS (ESI): calcd for $C_{57}H_{42}NS^+$ [M^+ - BF_4^-] 772.3033, found 772.3038.

2.5f. (5-benzhydryl-7,9,9-trimethyl-9*H*-pyrrolo[1,2-*a*]indol-2-yl)diphenylsulfonium tetrafluoroborate (8f)

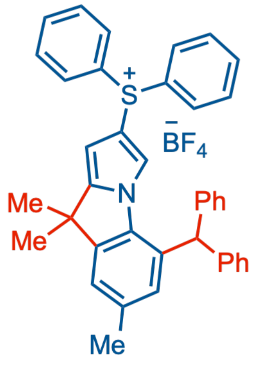
8f

Compound **8f** was synthesized using the general procedure D on 0.5 mmol scale. Yield = 77% (245 mg). **^{1}H NMR** (500 MHz, $CDCl_3$) δ 7.68 – 6.64 (m, 2H), 7.62 – 7.60 (m, 8H), 7.59 (d, *J* = 1.2 Hz, 1H), 7.29 – 7.26 (m, 4H), 7.21 (t, *J* = 7.3 Hz, 2H), 7.08 (d, *J* = 6.9 Hz, 5H), 6.57 (s, 1H), 6.41 (s, 1H), 5.96 (s, 1H), 2.28 (s, 3H), 1.52 (s, 6H). **^{13}C NMR** (126 MHz, $CDCl_3$) δ 151.3 (C_q-pyrrole), 145.8 (C_q-Ar), 141.8 (C_q-Ar), 136.6 (C_q-Ar), 133.9 (CH-Ar), 133.9 (CH-Ar), 131.3 (CH-Ar), 130.5 (CH-Ar), 130.1 (CH-Ar), 129.7 (CH-Ar), 129.5 (C_q-Ar), 128.9 (CH-Ar), 128.0 (C_q-Ar), 127.1 (CH-Ar), 122.8 (CH-Ar), 121.8 (CH, precarbene), 102.2 (C_q-pyrrole), 101.0 (CH-pyrrole), 51.9 (CH-(Ph_2)), 41.8 (C_q-(Me_2)), 28.1 ($(CH_3)_2$), 21.6 (CH_3). HRMS (ESI): calcd for $C_{39}H_{34}NS^+$ [M^+ - BF_4^-] 548.2407, found 548.2412.

2.6. General Procedure E for the Synthesis of Gold-Complexes (9-[Au])

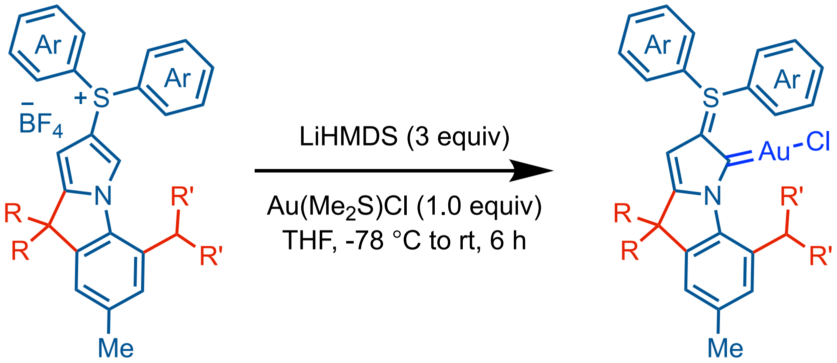

8 **9-[Au]**

An oven-dried reaction tube equipped with a stir bar was charged with **8** (0.20 mmol, 1.0 equiv) and [Au(Me_2S)Cl] (59.0 mg, 0.2 mmol, 1.0 equiv) under the argon atmosphere. Dry and degassed THF (2 mL) was added to the mixture and stirred at -78 °C temperature for 20 min. After that, LiHMDS (1 M in THF, 3.0 equiv) was added dropwise and the reaction mixture was stirred at room temperature for 6 h. The reaction mixture was filtered through celite using 10 mL of DCM. The solution was then concentrated under the reduced pressure and reprecipitated from DCM/hexanes (1:10 v/vol) to offer colorless solid Au-complex **9-[Au]**.

2.6a. Synthesis of Au-complex 9a-[Au]

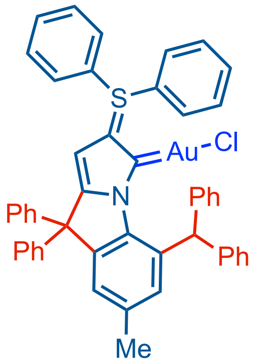

9a-[Au]

Complex **9a-[Au]** was synthesized using the general procedure E. Yield = 74% (134 mg). **^{1}H NMR** (500 MHz, $CDCl_3$) δ 9.20 (s, 1H), 7.54 (t, J = 7.3 Hz, 2H), 7.45 (t, J = 7.7 Hz, 4H), 7.40 (d, J = 7.9 Hz, 4H), 7.25 – 7.20 (m, 14H), 7.16 – 7.13 (m, 6H), 6.94 (s, 1H), 6.83 (s, 1H), 5.97 (s, 1H), 2.18 (s, 3H). **^{13}C NMR** (126 MHz, $CDCl_3$) δ 157.4 (C_q-Au), 152.4 (C_q-Pyrrole), 146.2 (C_q-Ar), 144.0 (C_q-Ar), 143.0 (C_q-Ar), 137.9 (C_q-Ar), 134.5 (C_q-Ar), 132.7 (CH-Ar), 132.4 (CH-Ar), 130.9 (C_q-Ar), 130.6 (CH-Ar), 130.5 (C_q-Ar), 130.4 (CH-Ar), 129.5 (CH-Ar), 128.4 (CH-Ar), 128.1 (CH-Ar), 126.9 (CH-Ar), 126.1 (CH-Ar), 126.1 (CH-Ar), 109.7 (C_q-pyrrole), 101.8 (CH-pyrrole), 58.2 (C_q-(Ph_2)), 52.3 (CH-(Ph_2)), 21.4 (CH_3). HRMS (ESI): calcd for $C_{49}H_{38}AuClNS$ $[M + H]^+$ 904.2074, found 904.2090.

2.6b. Synthesis of Au-complex 9b-[Au]

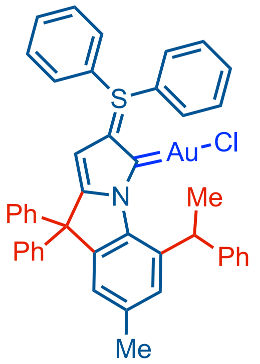

9b-[Au]

Complex **9b-[Au]** was synthesized using the general procedure E. Yield = 67% (112.7 mg). **^{1}H NMR** (500 MHz, $CDCl_3$) δ 7.63 – 7.59 (m, 2H), 7.51 – 7.50 (m, 8H), 7.49 – 7.46 (m, 1H), 7.35 (d, J = 7.5 Hz, 2H), 7.26 – 7.22 (m, 8H), 7.16 – 7.15 (m, 5H), 6.88 (s, 2H), 6.06 (s, 1H), 2.19 (s, 3H), 1.80 (d, J = 7.0 Hz, 3H). **^{13}C NMR** (126 MHz, $CDCl_3$) δ 157.4 (C_q-Au), 152.6 (C_q-pyrrole), 146.4 (C_q-Ar), 146.2 (C_q-Ar), 145.7 (C_q-Ar), 142.4 (C_q-Ar), 137.2 (C_q-Ar), 134.9 (C_q-Ar), 134.8 (C_q-Ar), 132.8 (CH-Ar), 132.7 (CH-Ar), 130.7 (CH-Ar), 130.6 (CH-Ar), 130.4 (C_q-Ar), 130.2 (CH-Ar), 129.6 (CH-Ar), 129.5 (CH-Ar), 128.45 (CH-Ar), 128.43 (CH-Ar), 128.16 (CH-Ar), 128.13 (CH-Ar), 126.89 (CH-Ar), 126.88 (CH-Ar), 125.8 (CH-Ar), 125.7 (CH-Ar), 109.8 (C_q-pyrrole), 101.9 (CH-pyrrole), 58.1 (C_q-(Ph_2)), 40.4 (CH-(PhMe)), 22.5 (CH_3), 21.3 (CH_3). HRMS (ESI): calcd for $C_{44}H_{36}AuClNS$ $[M + H]^+$ 842.1917, found 842.1923.

2.6c. Synthesis of Au-complex 9c-[Au]

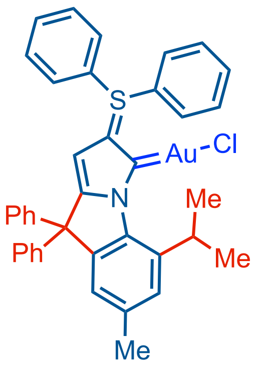

9c-[Au]

Complex **9c-[Au]** was synthesized using the general procedure E. Yield = 57% (89 mg). **^{1}H NMR** (500 MHz, $CDCl_3$) δ 7.62 – 7.57 (m, 3H), 7.55 – 7.51 (m, 7H), 7.22 – 7.19 (m, 6H), 7.15 – 7.13 (m, 5H), 6.87 (s, 1H), 6.04 (s, 1H), 5.97 – 5.94 (m, 1H), 2.29 (s, 3H), 1.39 (d, J = 6.8 Hz, 6H). **^{13}C NMR** (126 MHz, $CDCl_3$) δ 157.7 (C_q-Au), 152.6 (C_q-pyrrole), 146.4 (C_q-Ar), 142.3 (C_q-Ar), 137.2 (C_q-Ar), 136.8 (C_q-Ar), 134.8(C_q-Ar), 132.7 (CH-Ar), 130.71 (C_q-Ar), 130.65 (CH-Ar), 129.5 (CH-Ar), , 128.4

(CH-Ar), 128.2 (CH-Ar), 127.4 (CH-Ar), 126.8 (CH-Ar), 125.2 (CH-Ar), 109.6 (C_q-pyrrole), 101.8 (CH-pyrrole), 58.2 (Cq-(Ph_2)), 30.4 (Cq-(Me_2)), 24.4 (CH_3), 21.4 (CH_3). HRMS (ESI): calcd for $C_{39}H_{34}AuClNS$ $[M + H]^+$ 780.1761, found 780.1777.

2.6d. Synthesis of Au-complex 9d-[Au]

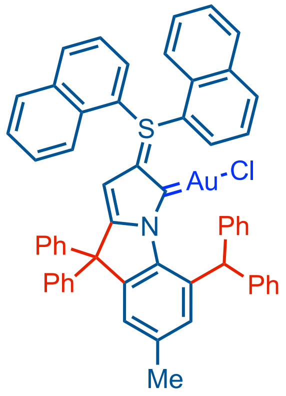

9d-[Au]

Complex **9d-[Au]** was synthesized using the general procedure E. Yield = 79% (158.5 mg). **^{1}H NMR** (500 MHz, $CDCl_3$) δ 9.24 (s, 1H), 8.24 – 8.22 (m, 2H), 8.04 (d, *J* = 8.1 Hz, 2H), 7.96 – 7.94 (m, 2H), 7.64 – 7.60 (m, 4H), 7.39 (t, *J* = 7.8 Hz, 2H), 7.32 (d, *J* = 7.4 Hz, 2H), 7.28 – 7.23 (m, 8H), 7.18 – 7.13 (m, 8H), 7.03 – 7.01 (m, 4H), 6.93 (s, 1H), 6.82 (s, 1H), 5.82 (s, 1H), 2.19 (s, 3H). **^{13}C NMR** (126 MHz, $CDCl_3$) δ 157.5 (C_q-Au), 151.8 (C_q-pyrrole), 146.2 (C_q-Ar), 144.0 (C_q-Ar), 143.0 (C_q-Ar), 138.1 (C_q-Ar), 134.4 (C_q-Ar), 134.3 (C_q-Ar), 133.7 (C_q-Ar), 132.2 (C_q-Ar), 131.0 (C_q-Ar), 130.4 (CH-Ar), 130.1 (CH-Ar), 129.6 (CH-Ar), 129.4 (CH-Ar), 129.3 (CH-Ar), 128.4 (CH-Ar), 128.13 (CH-Ar), 128.09 (CH-Ar), 128.0 (CH-Ar), 126.8 (CH-Ar), 126.1 (CH-Ar), 126.0 (CH-Ar), 125.6 (CH-Ar), 125.2 (CH-Ar), 123.3 (CH-Ar), 106.3 (C_q-pyrrole), 103.2 (CH-pyrrole), 58.1 (Cq-(Ph_2)), 52.4 (CH-(Ph_2)), 21.4 (CH_3). HRMS (ESI): calcd for $C_{57}H_{42}AuClNS^+$ $[M + H]^+$ 1004.2387, found 1004.2402.

2.6e. Synthesis of Au-complex 9e-[Au]

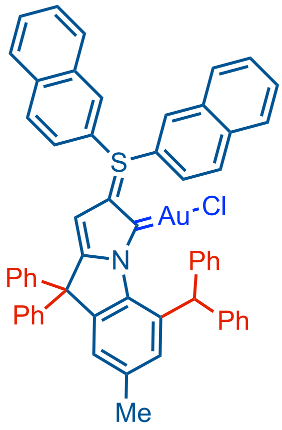

9e-[Au]

Complex **9e-[Au]** was synthesized using the general procedure E. Yield = 55% (110.4 mg). **^{1}H NMR** (500 MHz, $CDCl_3$) δ 9.27 (s, 1H), 8.03 (s, 2H), 7.88 (t, *J* = 8.2 Hz, 4H), 7.75 (d, *J* = 8.0 Hz, 2H), 7.65 (t, *J* = 7.4 Hz, 2H), 7.61 (t, *J* = 7.4 Hz, 2H), 7.35 (dd, *J* = 8.8, 1.3 Hz, 2H), 7.29 – 7.20 (m, 14H), 7.19 – 7.14 (m, 6H), 6.96 (s, 1H), 6.86 (s, 1H), 6.15 (s, 1H), 2.20 (s, 3H). **^{13}C NMR** (126 MHz, $CDCl_3$) δ 157.5 (C_q-Au), 152.7 (C_q-pyrrole), 146.3 (C_q-Ar), 144.1 (C_q-Ar), 143.1 (C_q-Ar), 138.0 (C_q-Ar), 134.5 (C_q-Ar), 133.0 (C_q-Ar), 132.4 (CH-Ar), 131.4 (CH-Ar), 131.0 (CH-Ar), 130.95 (C_q-Ar), 130.4 (CH-Ar), 129.5 (CH-Ar), 129.0 (CH-Ar), 128.5 (CH-Ar), 128.3 (CH-Ar), 128.19 (CH-Ar), 128.17 (CH-Ar), 128.15 (CH-Ar), 127.2 (C_q-Ar), 126.9 (CH-Ar), 126.13 (CH-Ar), 126.10 (CH-Ar), 123.8 (CH-Ar), 110.1 (C_q-pyrrole), 101.8 (CH-pyrrole), 58.3 (C_q-(Ph_2)), 52.3 (CH-(Ph_2)), 21.4 (CH_3). HRMS (ESI): calcd for $C_{57}H_{42}AuClNS$ $[M + H]^+$ 1004.2387, found 1004.2399.

2.6f. Synthesis of Au-complex 9f-[Au]

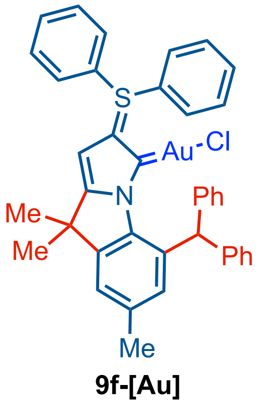

9f-[Au]

Complex **9f-[Au]** was synthesized using the general procedure E. Yield = 83% (129.2 mg). **^{1}H NMR** (500 MHz, $CDCl_3$) δ 9.19 (s, 1H), 7.60 (t, J = 7.1 Hz, 2H), 7.56 – 7.51 (m, 8H), 7.30 – 7.28 (m, 4H), 7.26 – 7.23 (m, 4H), 7.15 (t, J = 7.1 Hz, 2H), 6.98 (s, 1H), 6.85 (s, 1H), 5.96 (s, 1H), 2.27 (s, 3H), 1.42 (s, 6H). **^{13}C NMR** (126 MHz, $CDCl_3$) δ 156.7 (C_q-Au), 155.2 (C_q-pyrrole), 146.4 (C_q-Ar), 144.2 (C_q-Ar), 137.5 (C_q-Ar), 134.2 (C_q-Ar), 132.6 (CH-Ar), 131.9 (CH-Ar), 130.8 (C_q-Ar), 130.6 (CH-Ar), 130.4 (CH-Ar), 129.6 (CH-Ar), 128.1 (CH-Ar), 126.0 (CH-Ar), 122.1 (CH-Ar), 108.7 (Cq-pyrrole), 97.6 (CH-pyrrole), 52.0 (CH-(Ph_2)), 39.7 (C_q-(Me_2)), 29.3 (2CH_3), 21.3 (CH_3). HRMS (ESI): calcd for $C_{39}H_{34}AuClNS$ $[M + H]^+$ 780.1761, found 780.1774.

2.7. Synthesis of Selenium-Complex (9a-[Se])

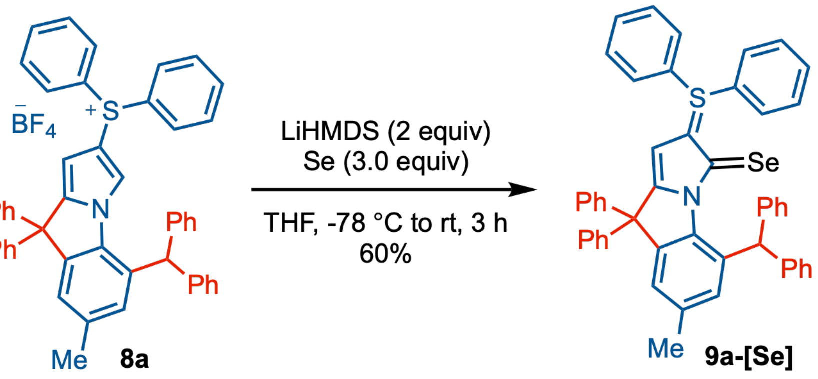

An oven-dried reaction tube equipped with a stir bar was charged with **8a** (100 mg, 0.148 mmol). It was dissolved in dry and degassed THF (2 mL) and LiHMDS (1 M in THF, 2.0 equiv) was added under the argon atmosphere at –78 °C. After 1 h stirring at –78 °C, Se (35 mg, 0.444 mmol, 3 equiv) was added to the mixture under the argon atmosphere at –78 °C. The reaction mixture was gradually warmed up to room temperature and stirred for 2 h. The reaction mixture was filtered through celite using 10 mL of DCM. The solution was then concentrated under reduced pressure and reprecipitated from DCM/hexanes (1:10 v/vol) to offer colorless solid Se-complex **9a-[Se]**. Yield = 60% (66 mg). **^{1}H NMR** (500 MHz, $CDCl_3$) δ 9.88 (s, 1H), 7.53 (t, J = 7.3 Hz, 2H), 7.45 (t, J = 7.6 Hz, 4H), 7.39 (d, J = 7.7 Hz, 4H), 7.26 – 7.19 (m, 14H), 7.16 – 7.12 (m, 6H), 6.88 (s, 1H), 6.80 (s, 1H), 5.50 (s, 1H), 2.19 (s, 1H). **^{13}C NMR** (126 MHz, $CDCl_3$) δ 148.5 (C_q-Ar), 146.5 (C_q-Ar), 145.3 (C_q-Ar), 143.1 (C_q-Ar), 138.6 (C_q-Ar), 134.4 (C_q-Ar), 132.6 (C_q-Ar), 132.2 (CH-Ar), 131.9 (C_q-Ar), 130.4 (CH-Ar), 130.2 (CH-Ar), 129.9

(CH-Ar), 128.27 (CH-Ar), 128.25 (CH-Ar), 127.9 (CH-Ar), 126.6 (CH-Ar), 125.8 (CH-Ar), 125.5 (CH-Ar), 103.3 (C_q-Ar), 58.5 (C_q-(Ph_2)), 51.8 (CH-(Ph_2)), 21.4 (CH_3). **^{77}Se NMR** (95 MHz, $CDCl_3$) δ 177.77. HRMS (ESI): calcd for $C_{49}H_{38}NSSe$ $[M+H]^+$ 752.1885, found 752.1890.

2.8. Synthesis of Rhodium-Complex (9a-[Rh])

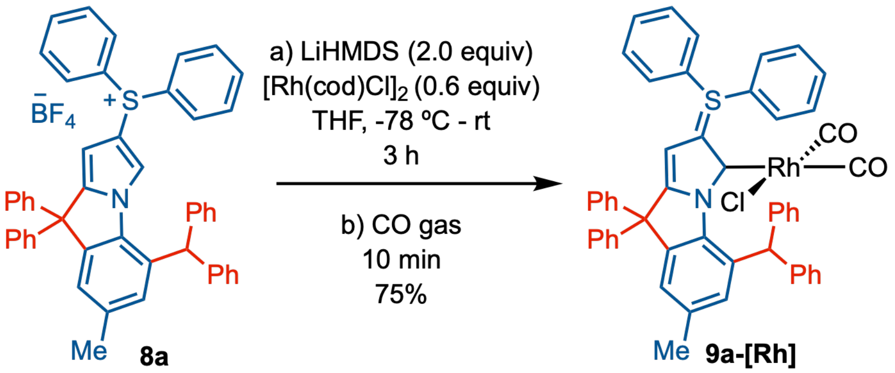

An oven-dried reaction tube equipped with a stir bar was charged with **8a** (100 mg, 0.148 mmol). It was dissolved in dry and degassed THF (2 mL) and LiHMDS (1 M in THF, 2.0 equiv) was added under the argon atmosphere at –78 °C. After 1 h stirring at –78 °C, [Rh(cod)Cl]2 (44 mg, 0.088 mmol, 0.6 equiv) was added to the mixture under the argon atmosphere at –78 °C. The reaction mixture was gradually warmed up to room temperature and stirred for 2 h. To the same reaction mixture CO gas was bubbled for 10 min. The reaction mixture was filtered through celite using 10 mL of DCM. The solution was then concentrated under the reduced pressure and reprecipitated from DCM/hexanes (1:10 v/vol) to offer yellow solid Rh-complex **9a-[Rh]**. Yield = 75% (96 mg). **^{1}H NMR** (500 MHz, $CDCl_3$) δ 9.21 (s, 1H), 7.57 – 7.55 (m, 2H), 7.52 – 7.45 (m, 8H), 7.30 – 7.27 (m, 4H), 7.25 – 7.12 (m, 16H), 6.93 (s, 1H), 6.89 (s, 1H), 5.97 (s, 1H), 2.17 (s, 3H). **^{13}C NMR** (126 MHz, $CDCl_3$) δ 186.74 (CO, d, J = 54.0 Hz), 183.27 (CO, d, J = 78.1 Hz), 162.72 (C_q-Rh, d, J = 36.8 Hz), 154.0 (C_q-Ar), 146.5 (C_q-Ar), 146.4 (C_q-Ar), 146.0 (C_q-Ar), 144.5 (C_q-Ar), 143.0 (C_q-Ar), 137.9 (C_q-Ar), 134.3 (C_q-Ar), 132.6 (CH-Ar), 132.5 (CH-Ar), 132.2 (CH-Ar), 131.5 (C_q-Ar), 130.9 (C_q-Ar), 130.7 (CH-Ar), 130.6 (CH-Ar), 130.3 (CH-Ar), 130.3 (CH-Ar), 129.72 (CH-Ar), 129.69 (CH-Ar), 128.8 (CH-Ar), 128.44 (CH-Ar), 128.38 (CH-Ar), 128.3 (CH-Ar), 128.2 (CH-Ar), 128.1 (CH-Ar), 127.8 (CH-Ar), 126.8 (CH-Ar), 126.7 (CH-Ar), 126.2 (CH-Ar), 126.1 (CH-Ar), 125.9 (CH-Ar), 109.1 (C_q-Ar), 102.9 (CH-Ar), 58.3 (C_q-(Ph_2)), 52.1 (CH-(Ph_2)), 21.4 (CH_3). HRMS (ESI): calcd for $C_{51}H_{37}RhNSO_2$ $[M - Cl]^+$ 830.1595, found 830.1619.

2.9. General Procedure for the Hydroamination of Alkynes

General procedure: An oven-dried 10 mL reaction tube equipped with a stir bar was charged with terminal alkyne **16** (0.21 mmol, 1.05 equiv), amine **17** (0.20 mmol, 1.0 equiv), Au-catalyst, **9a-[Au]** (3.6 mg, 0.004 mmol, 2 mol%), and sodium tetrakis[3,5-bis(trifluoromethyl)phenyl]borate, $NaBAr^F_4$ (7.1 mg, 0.008 mmol, 4 mol%) as additive. The mixture was stirred at room temperature in chloroform (0.1 mL) for 16 h. After the reaction the the mixture was diluted CH_2Cl_2 (1 mL) and was quantitatively transferred to a screw-capped vial containing sodium triacetoxyborohydride (85 mg, 0.4 mmol) followed by the addition of acetic acid (0.023 mL, 0.4 mmol). The suspension was then stirred at ambient temperature for 4h. The reaction was quenched by the addition of 1M NaOH (aq) (1 mL). The phases were separated and the aqueous layer extracted with two further portions of CH_2Cl_2. The combined organic phases were dried over Na_2SO_4 and filtered. After removal of the solvent, the resulting tertiary amine products were purified by column chromatography on silica gel.

2.9a. 9-ethyl-*N*-(1-phenylethyl)-9*H*-carbazol-3-amine (18a)

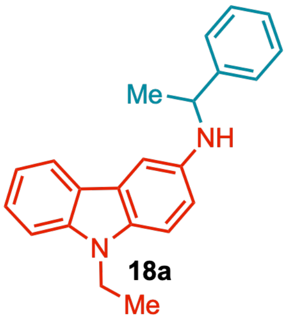

18a

Yield = 60%, **^{1}H NMR** (500 MHz, $CDCl_3$) δ 7.90 (d, *J* = 7.8 Hz, 1H), 7.46 (d, *J* = 7.2 Hz, 2H), 7.38 (t, *J* = 7.6 Hz, 1H), 7.35 – 7.29 (m, 3H), 7.24 – 7.21 (m, 2H), 7.17 (d, *J* = 8.5 Hz, 1H), 7.10 (t, *J* = 7.4 Hz, 1H), 6.81 (dd, *J* = 8.6, 2.5 Hz, 1H), 4.60 (q, *J* = 6.5 Hz, 1H), 4.26 (q, *J* = 7.4 Hz, 2H), 3.97 (brs, 1H), 1.58 (d, *J* = 6.7 Hz, 3H), 1.36 (t, *J* = 7.2 Hz, 3H). **^{13}C NMR** (126 MHz, $CDCl_3$) δ 145.8 (C_q-Ar), 140.4 (C_q-Ar), 134.0 (C_q-Ar), 128.8 (CH-Ar), 127.0 (CH-Ar), 126.2 (CH-Ar), 125.4 (CH-Ar), 123.6 (C_q-Ar), 122.7 (CH-Ar), 120.4 (CH-Ar), 117.9 (CH-Ar), 114.8 (C_q-Ar), 109.1 (CH-Ar), 108.4 (CH-Ar), 104.4 (C_q-Ar), 54.9 (CH), 37.6 (CH_2), 25.2 (CH_3), 14.0 (CH_3). HRMS (ESI): calcd for $C_{22}H_{23}N_2$ $[M + H]^+$ 315.1856, found 315.1859.

2.9b. *N*-(1-phenylethyl)benzo[*b*]thiophen-5-amine (18b)

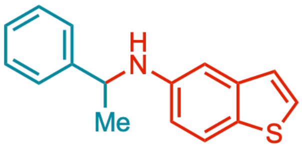

18b

Yield = 80%, **^{1}H NMR** (500 MHz, $CDCl_3$) δ 7.57 (d, *J* = 8.7 Hz, 1H), 7.41 – 7.40 (m, 2H), 7.34 – 7.30 (m, 3H), 7.23 (t, *J* = 7.3 Hz, 1H), 7.06 (d, *J* = 5.3 Hz, 1H), 6.82 (d, *J* = 2.6 Hz, 1H), 6.70 (dd, *J* = 8.6, 2.5 Hz, 1H), 4.55 (q, *J* = 6.7 Hz, 1H), 4.08 (s, 1H), 1.56 (d, *J* = 6.7 Hz, 3H). **^{13}C NMR** (126 MHz, $CDCl_3$) δ 145.3 (C_q-Ar), 144.9 (C_q-Ar), 141.0 (C_q-Ar), 129.3 (C_q-Ar), 128.8 (CH-Ar), 127.1 (CH-Ar), 126.8 (CH-Ar), 126.0 (CH-Ar), 123.5 (CH-Ar),

122.8 (CH-Ar), 114.3 (CH-Ar), 105.9 (CH-Ar), 54.0 (CH), 25.2 (CH_3). HRMS (ESI): calcd for $C_{16}H_{16}NS$ $[M + H]^+$ 254.0998, found 254.1003.

2.9c. 1-methyl-*N*-(1-phenylethyl)-1*H*-indol-5-amine (18c)

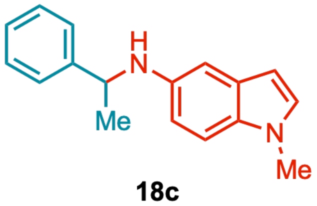

18c

Yield = 62%, ^{1}H NMR (500 MHz, $CDCl_3$) δ 7.42 (d, *J* = 8.1 Hz, 2H), 7.31 (t, *J* = 7.7 Hz, 2H), 7.21 (t, *J* = 7.3 Hz, 1H), 7.09 (d, *J* = 8.4 Hz, 1H), 6.90 (d, *J* = 3.1 Hz, 1H), 6.67 (d, *J* = 2.7 Hz, 1H), 6.62 (dd, *J* = 8.7, 3.1 Hz, 1H), 6.21 (d, *J* = 3.1 Hz, 1H), 4.53 (q, *J* = 6.7 Hz, 1H), 3.69 (s, 3H), 1.54 (d, *J* = 6.6 Hz, 3H). ^{13}C NMR (126 MHz, $CDCl_3$) δ 146.0 (C_q-Ar), 141.2 (C_q-Ar), 131.3 (C_q-Ar), 129.3 (C_q-Ar), 128.9 (CH-Ar), 128.7 (CH-Ar), 126.8 (CH-Ar), 126.1 (CH-Ar), 112.1 (CH-Ar), 109.8 (CH-Ar), 103.6 (CH-Ar), 99.9 (CH-Ar), 54.7 (CH), 33.0 (CH_3(N)), 25.3 (CH_3). HRMS (ESI): calcd for $C_{17}H_{19}N_2$ $[M + H]^+$ 251.1543, found 251.1543.

2.9d. 1-methyl-*N*-(1-phenylethyl)-1*H*-indazol-5-amine (18d)

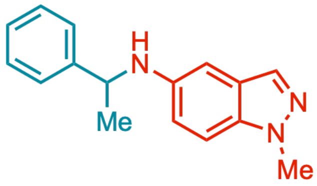

18d

Yield = 91%, **^{1}H NMR** (500 MHz, $CDCl_3$) δ 7.67 (s, 1H), 7.40 (d, *J* = 7.2 Hz, 2H), 7.32 (t, *J* = 7.7 Hz, 2H), 7.23 (t, *J* = 6.6 Hz, 1H), 7.17 (d, *J* = 8.9 Hz, 1H), 6.83 (d, *J* = 6.6 Hz, 1H), 6.57 (s, 1H), 4.50 (q, *J* = 6.6 Hz, 1H), 3.97 (s, 3H), 1.55 (d, *J* = 6.7 Hz, 3H). **^{13}C NMR** (126 MHz, $CDCl_3$) δ 145.3 (C_q-Ar), 141.7 (C_q-Ar), 135.2 (C_q-Ar), 131.4 (CH-Ar), 128.8 (CH-Ar), 127.0 (CH-Ar), 126.0 (CH-Ar), 125.0 (C_q-Ar), 118.3 (CH-Ar), 109.7 (CH-Ar), 100.7 (CH-Ar), 54.4 (CH), 35.6 (CH_3(N)), 25.3 (CH_3). HRMS (ESI): calcd for $C_{16}H_{18}N_3$ $[M + H]^+$ 252.1495, found 252.1497.

2.9e. 4-methyl-*N*-(1-phenylethyl)aniline (18e)

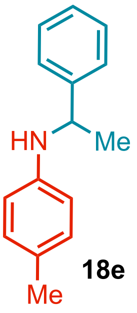

18e

Yield = 75%, **^{1}H NMR** (500 MHz, $CDCl_3$) δ 7.39 (d, *J* = 7.3 Hz, 2H), 7.35 – 7.32 (m, 2H), 7.26 – 7.22 (m, 1H), 6.93 (d, *J* = 8.5 Hz, 2H), 6.47 (d, *J* = 8.5 Hz, 2H), 4.48 (q, *J* = 6.8 Hz, 1H), 3.99 (s, 1H), 2.21 (s, 3H), 1.53 (d, *J* = 6.7 Hz, 3H). **^{13}C NMR** (126 MHz, $CDCl_3$) δ 145.5 (C_q-Ar), 145.1 (C_q-Ar), 129.7 (CH-Ar), 128.7 (CH-Ar), 126.9 (CH-Ar), 126.6 (C_q-Ar), 126.0 (CH-Ar), 113.6 (CH-Ar), 53.9 (CH), 25.1 (CH_3), 20.5 (CH_3). HRMS (ESI): calcd for $C_{15}H_{18}N$ $[M + H]^+$ 212.1434, found 212.1440.

2.9f. 4-iodo-*N*-(1-phenylethyl)aniline (18f)

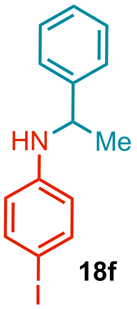

18f

Yield = 55%, **^{1}H NMR** (500 MHz, $CDCl_3$) δ 7.34 – 7.32 (m, 6H), 7.25 – 7.22 (m, 1H), 6.30 (d, *J* = 8.9 Hz, 2H), 4.44 (q, *J* = 6.6 Hz, 1H), 4.21 (brs, 1H), 1.52 (d, *J* = 6.7 Hz, 3H). **^{13}C NMR** (126 MHz, $CDCl_3$) δ 146.8 (C_q-Ar), 144.6 (C_q-Ar), 137.8 (CH-Ar), 128.9 (CH-Ar), 127.2 (CH-Ar), 125.9 (CH-Ar), 115.8 (CH-Ar), 78.2 (C_q-Ar), 53.6 (CH), 25.0 (CH_3). HRMS (ESI): calcd for $C_{14}H_{15}IN$ $[M + H]^+$ 324.0244, found 324.0258.

2.9g. 4-(methylthio)-*N*-(1-phenylethyl)aniline (18g)

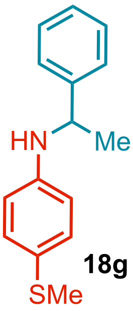

18g

Yield = 45%, **^{1}H NMR** (500 MHz, $CDCl_3$) δ ^{1}H NMR (500 MHz, $CDCl_3$) δ 7.36 – 7.30 (m, 4H), 7.25 – 7.22 (m, 1H), 7.13 (d, *J* = 8.7 Hz, 2H), 6.47 (d, *J* = 8.7 Hz, 2H), 4.46 (q, *J* = 6.7 Hz, 1H), 4.24 (brs, 1H), 2.36 (s, 3H), 1.52 (d, *J* = 6.7 Hz, 3H). **^{13}C NMR** (126 MHz, $CDCl_3$) δ 146.1 (C_q-Ar), 144.9 (C_q-Ar), 131.4 (CH-Ar), 128.8 (CH-Ar), 127.2 (CH-Ar), 126.0 (CH-Ar), 114.2 (CH-Ar), 53.8 (CH), 25.0 (CH_3), 19.2 (CH_3). HRMS (ESI): calcd for $C_{15}H_{18}NS$ $[M + H]^+$ 244.1154, found 244.1165.

2.9h. *N*-(1-([1,1'-biphenyl]-4-yl)ethyl)aniline (18h)

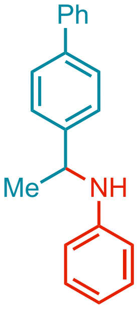

18h

Yield = 80%, **^{1}H NMR** (500 MHz, $CDCl_3$) δ 7.60 – 7.55 (m, 4H), 7.46 – 7.42 (m, 4H), 7.34 (t, *J* = 8.2 Hz, 1H), 7.14 – 7.11 (m, 2H), 6.69 (t, *J* = 7.3 Hz, 1H), 6.58 (d, *J* = 7.3 Hz, 2H), 4.55 (q, *J* = 6.7 Hz, 1H), 4.30 (brs, 1H), 1.58 (d, *J* = 6.9 Hz, 3H). **^{13}C NMR** (126 MHz, $CDCl_3$) δ 147.1 (C_q-Ar), 144.2 (C_q-Ar), 141.1 (C_q-Ar), 140.0 (C_q-Ar), 129.3 (CH-Ar), 128.9 (CH-Ar), 127.5 (CH-Ar), 127.3 (CH-Ar), 127.2 (CH-Ar), 126.5 (CH-Ar), 117.7 (CH-Ar), 113.7 (CH-Ar), 53.6 (C_q), 25.0 (CH_3). HRMS (ESI): calcd for $C_{20}H_{20}N$ $[M + H]^+$ 274.1490, found 274.1602.

2.10. General Procedure for the Au-Catalyzed Synthesis of 1,2-Dihydroquinoline Derivatives

An oven-dried 10 mL reaction tube equipped with a stir bar was charged with terminal alkyne **16** (102 mg, 1 mmol, 4.0 equiv), *N*-methylaniline **17** (26.8 mg, 0.25 mmol, 1.0 equiv), Au-catalyst, **9a-[Au]** (11.3 mg, 0.0125 mmol, 5 mol%), and sodium tetrakis[3,5-bis(trifluoromethyl)phenyl]borate, $NaBAr^F{}_4$ (11.1 mg, 0.0125 mmol, 5 mol%) as additive. The

reaction mixture was heated at 100 °C for 16 h. The products were purified by column chromatography.

2.10a. 1,2-dimethyl-2,4-diphenyl-1,2-dihydroquinoline (20a)

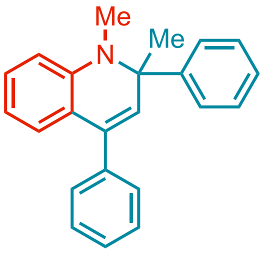

20a

Yield = 61%. **^{1}H NMR** (500 MHz, $CDCl_3$) δ 7.59 (dd, *J* = 8.4, 1.4 Hz, 2H), 7.37 – 7.30 (m, 7H), 7.23 – 7.25 (m, 1H), 7.15 (td, *J* = 7.5, 1.7 Hz, 1H), 6.91 (dd, *J* = 7.9, 1.6 Hz, 1H), 6.58 – 6.55 (m, 2H), 5.31 (s, 1H), 2.60 (s, 3H), 1.79 (s, 3H). **^{13}C NMR** (126 MHz, $CDCl_3$) δ 147.7 (C_q-Ar), 145.3 (C_q-Ar), 139.7 (C_q-Ar), 134.0 (C_q-Ar), 130.6 (CH-Ar), 129.5 (CH-Ar), 129.2 (CH-Ar), 128.5 (CH-Ar), 128.3 (CH-Ar), 127.4 (CH-Ar), 127.0 (CH-Ar), 127.0 (CH-Ar), 126.1 (CH-Ar), 121.1 (C_q-Ar), 116.1 (CH-Ar), 110.3 (CH-Ar), 63.5 (C_q), 33.0 (CH_3(N)), 23.3 (CH_3).

2.10b. 2,4-bis(4-methoxyphenyl)-1,2-dimethyl-1,2-dihydroquinoline (20b)

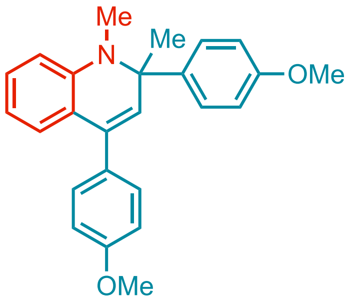

20b

Yield = 85%, **^{1}H NMR** (500 MHz, $CDCl_3$) δ 7.50 (d, *J* = 8.7 Hz, 2H), 7.26 (d, *J* = 8.7 Hz, 2H), 7.15 (t, *J* = 7.4 Hz, 1H), 6.95 (d, *J* = 7.5 Hz, 1H), 6.91 – 6.88 (m, 4H), 6.58 (t, *J* = 7.5 Hz, 2H), 5.30 (s, 1H), 3.83 (s, 3H), 3.81 (s, 3H), 2.60 (s, 3H), 1.75 (s, 3H). **^{13}C NMR** (126 MHz, $CDCl_3$) δ 159.0 (C_q-Ar), 158.6 (C_q-Ar), 145.2 (C_q-Ar), 140.0 (C_q-Ar), 133.3 (C_q-Ar), 132.0 (C_q-Ar), 130.5 (CH-Ar), 130.3 (CH-Ar), 129.3 (CH-Ar), 128.1 (CH-Ar), 126.0 (CH-Ar), 121.4 (C_q-Ar), 116.0 (CH-Ar), 113.65 (CH-Ar), 113.69 (CH-Ar), 110.4 (CH-Ar), 62.9 (C_q), 55.43 (OMe), 55.40 (OMe), 32.8 (CH_3(N)), 23.4 (CH_3).

2.10c. 2,4-bis(4-bromophenyl)-1,2-dimethyl-1,2-dihydroquinoline (20c)

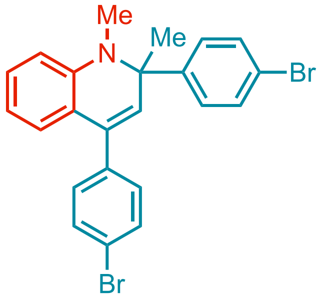

20c

Yield = 56%, **^{1}H NMR** (500 MHz, $CDCl_3$) δ 7.49 – 7.44 (m, 6H), 7.20 – 7.15 (m, 3H), 6.85 (d, *J* = 7.6 Hz, 1H), 6.59 (t, *J* = 7.6 Hz, 2H), 5.25 (s, 1H), 2.59 (s, 3H), 1.76 (s, 3H). **^{13}C NMR** (126 MHz, $CDCl_3$) δ 146.6 (C_q-Ar), 145.0 (C_q-Ar), 138.3 (C_q-Ar), 133.5 (C_q-Ar), 131.6 (CH-Ar), 131.5 (CH-Ar), 130.8 (CH-Ar), 130.0 (CH-Ar), 129.9 (CH-Ar), 128.7 (CH-Ar), 126.0 (CH-Ar), 121.5 (C_q-Ar), 121.3 (C_q-Ar), 120.6 (C_q-Ar), 116.5 (CH-Ar), 110.6 (CH-Ar), 63.3 (C_q), 33.0 (CH_3(N)), 23.3 (CH_3). HRMS (ESI): calcd for $C_{35}H_{30}N$ $[M + H]^+$ 469.9937, found 469.9936.

2.10d. 2,4-di([1,1'-biphenyl]-4-yl)-1,2-dimethyl-1,2-dihydroquinoline (20d)

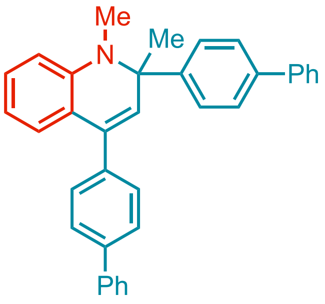

20d

Yield = 74%, ^{1}**H NMR** (500 MHz, $CDCl_3$) δ ^{1}H NMR (500 MHz, $CDCl_3$) δ 7.67 (d, J = 8.4 Hz, 2H), 7.63 – 7.59 (m, 8H), 7.46 – 7.43 (m, 6H), 7.37 – 7.33 (m, 2H), 7.19 (t, J = 7.7 Hz, 1H), 7.03 (d, J = 7.8 Hz, 1H), 6.64 – 6.61 (m, 2H), 5.42 (s, 1H), 2.68 (s, 3H), 1.85 (s, 3H). ^{13}**C NMR** (126 MHz, $CDCl_3$) δ 146.6 (C_q-Ar), 145.3 (C_q-Ar), 141.1 (C_q-Ar), 140.9 (C_q-Ar), 140.4 (C_q-Ar), 139.9 (C_q-Ar), 138.6 (C_q-Ar), 133.8 (C_q-Ar), 133.1 (CH-Ar), 130.5 (CH-Ar), 129.6 (CH-Ar), 129.1 (CH-Ar), 128.9 (CH-Ar), 127.4 (CH-Ar), 127.3 (CH-Ar), 127.2 (CH-Ar), 127.1 (CH-Ar), 126.1 (CH-Ar), 121.09 (C_q-Ar), 116.3 (CH-Ar), 110.5 (CH-Ar), 63.5 (C_q), 33.2 (CH_3(N)), 23.4 (CH_3). HRMS (ESI): calcd for $C_{35}H_{30}N$ $[M + H]^+$ 464.2373, found 464.2373.

2.10e. 4,4'-(1,2-dimethyl-1,2-dihydroquinoline-2,4-diyl)bis(*N*,*N*-dimethylaniline) (20e)

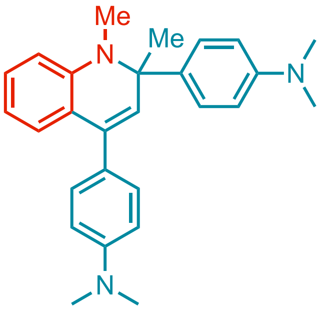

20e

Yield = 42%, ^{1}**H NMR** (500 MHz, $CDCl_3$) δ 7.43 (d, J = 8.9 Hz, 2H), 7.22 (d, J = 8.9 Hz, 2H), 7.13 – 7.10 (m, 1H), 7.02 (d, J = 7.6 Hz, 1H), 6.73 – 6.70 (m, 4H), 6.57 – 6.53 (m, 2H), 5.31 (s, 1H), 2.96 (s, 6H), 2.94 (s, 6H), 2.60 (s, 3H), 1.72 (s, 3H). ^{13}**C NMR** (126 MHz, $CDCl_3$) δ 145.5 (C_q-Ar), 133.8 (C_q-Ar), 133.3 (C_q-Ar), 130.7 (C_q-Ar), 130.5 (CH-Ar), 129.9 (CH-Ar), 129.0 (CH-Ar), 127.8 (CH-Ar), 126.0 (CH-Ar), 121.8 (C_q-Ar), 115.7 (CH-Ar), 112.4 (CH-Ar), 112.3 (CH-Ar), 111.9 (C_q-Ar), 110.8 (C_q-Ar), 110.2 (CH-Ar), 62.7 (CH), 40.9 (CH_3), 40.8 (CH_3), 32.7 (CH_3), 29.9 (CH_3), 23.4 (CH_3). HRMS (ESI): calcd for $C_{27}H_{32}N_3$ $[M + H]^+$ 398.2591, found 398.2596.

2.10f. 2,4-bis(cyclohexylmethyl)-1,2-dimethyl-1,2-dihydroquinoline (20f)

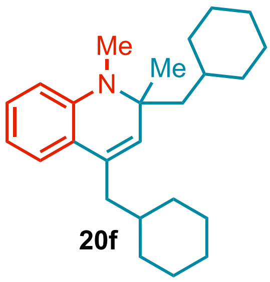

20f

Yield = 40%, ^{1}**H NMR** (500 MHz, $CDCl_3$) δ 7.07 – 7.06 (m, 1H), 7.02 (d, J = 7.6 Hz, 1H), 6.57 (td, J = 7.3, 1.1 Hz, 1H), 6.44 (d, J = 8.1 Hz, 1H), 5.08 (s, 1H), 2.69 (s, 3H), 2.31 – 2.27 (m, 1H), 2.13 – 2.09 (m, 1H), 1.80 – 1.77 (m, 3H), 1.75 – 1.67 (m, 4H), 1.64 – 1.57 (m, 5H), 1.33 – 1.29 (m, 2H), 1.19 (s, 3H), 1.18 – 1.08 (m, 6H), 0.97 – 0.88 (m, 4H). ^{13}**C NMR** (126 MHz, $CDCl_3$) δ 146.0 (C_q-Ar), 130.5 (C_q-Ar), 129.9 (CH-Ar), 128.5 (CH-Ar), 123.4 (CH-Ar), 121.6 (C_q-Ar), 115.4 (CH-Ar), 110.0 (CH-Ar), 60.0 (C_q), 48.6 (CH), 40.8 (CH), 36.3 (CH_2), 35.3 (CH_2), 34.3 (CH_3), 33.9 (CH_2), 33.7 (CH_2),

31.1 (CH_2), 27.7 (CH_2), 26.8 (CH_2), 26.7 (CH_2), 26.62 (CH_2), 26.56 (CH_3), 26.5 (CH_2), 26.4 (CH_2). HRMS (ESI): calcd for $C_{25}H_{38}N$ $[M + H]^+$ 352.2999, found 352.2977.

2.11. Comparative Catalysis Study

2.11a. Comparative Study Between 9a-[Au] and IPrAuCl in Alkyne Hydroamination

Two oven-dried 10 mL reaction tube equipped with a stir bar was charged with phenylacetylene **16** (53.6 mg 0.525 mmol, 1.05 equiv), aniline **17** (92 mg, 0.5 mmol, 1.0 equiv), and sodium tetrakis[3,5-bis(trifluoromethyl)phenyl]borate, $NaBAr^F{}_4$ (2.7 mg, 0.003 mmol, 0.6 mol%) as additive. In the first reaction tube, **9a-[Au]** (1.4 mg, 0.0015 mmol, 0.3 mol%) was added, and the reaction was stirred at room temperature for 24 h. The second reaction tube, IPrAuCl (0.9 mg, 0.0015 mmol, 0.3 mol%) and the reaction was stirred at room temperature for 24 h. The yield of the reaction was measured after 6 h and 24 h through ^1H NMR using dibromomethane as an internal standard.

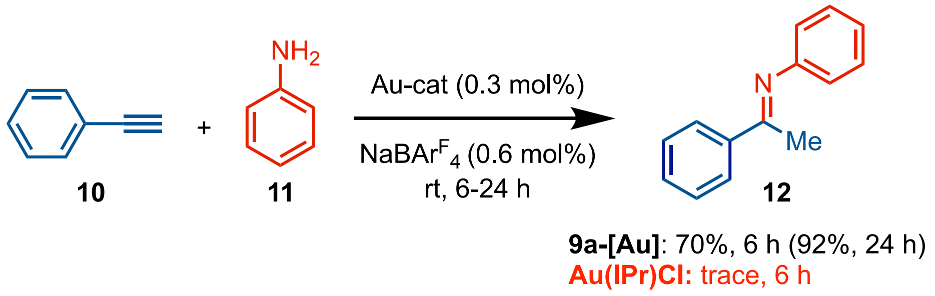

2.11b. Comparative Study between *b*CAYC-AuCl Catalysts 9a-[Au]–9f-[Au] in Alkyne Hydroamination

This experiment was conducted using the previous literature procedures.[5] All the kinetic measurements were performed in an NMR tube at room temperature on a Bruker 500MHz NMR machine. In a typical experiment, the NMR tube was charged with the six different Au catalysts **9a-[Au]–9f-[Au]** and $NaB(C_6F_5)_4$ (1:1 ratio), 4-ethynylanisole (0.047 g, 0.356 mmol, 1.0 eq.), and 0.2 mL $CDCl_3$ was added to it. The tube was locked and shimmed (T0) before starting the kinetic measurement. Following the addition of toluidine (0.356 mmol, 1.0 eq.) the reaction was monitored as a function of time at a constant temperature of 20 °C. The yield of the reaction was tracked by the change in characteristic signals for the starting material and the product (imine).

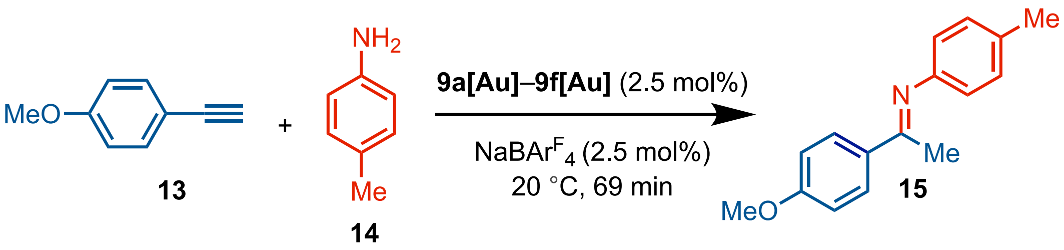

MeO
13
+
NH2
Me
14
9a[Au]–9f[Au] (2.5 mol%)
NaBArF4 (2.5 mol%)
20 °C, 69 min
N
Me
Me
MeO
15

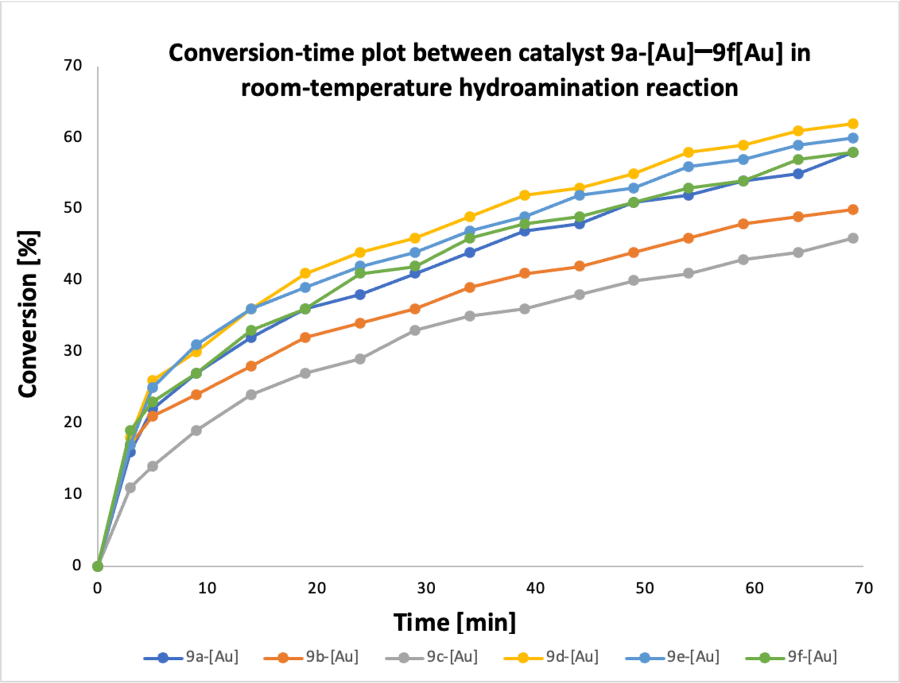

Conversion-time plot between catalyst 9a-[Au]–9f[Au] in room-temperature hydroamination reaction
Conversion [%]
Time [min]
0
10
20
30
40
50
60
70
9a-[Au]
9b-[Au]
9c-[Au]
9d-[Au]
9e-[Au]
9f-[Au]

3. Details of Crystal Structure Analysis of 9a-[Au], 9b-[Au], 9c-[Au], 9d-[Au], 9f-[Au]

All compounds were colorless single crystals. Full datasets were collected using graphite-monochromated CuKα radiation (λ = 1.54178 Å) on a XtaLAB Synergy single crystal diffractometer. X-rays were provided by a fine-focus sealed X-ray tube operated at 48kV and 30mA. Lattice constants were all determined using the SCALE3 ABSPACK software package using all available reflections (after data collection, ORTEP files, see Figures S1-S5).

All data were corrected for absorption by measuring the faces of each crystal and doing a numerical absorption correction. The Bruker software package SHELXTL-2014 was used to solve all of the structures using the direct methods technique and difference electron density maps. All stages of weighted full-matrix least-squares refinement were conducted using F_o^2 data with the same software package. The final structural model for each compound was refined using anisotropic thermal parameters for all non-hydrogen atoms; all of the H atoms were located in difference maps, but were placed in geometrically idealized positions and allowed to "ride" on their parent C, O or N atoms, with bond lengths of 0.95, 1.00, 0.99, 0.98, and 0.84 Å for aromatic, methine, methylene, methyl, and hydroxyl, respectively. The isotropic thermal parameters for these H atoms were fixed to be 1.2 times the U_{iso} for C or N and 1.5 times the U_{iso} for O.

Details for all of the structures are given in Table S1 and Table S2. Also included in this table are the largest shifts/s.u. for the final cycle of refinement and the largest maxima and minima in any of the final difference maps.

3. Crystallographic Studies

3.1. Crystal Data and Structure Refinement Summaries[6]

Table S1. Crystal Data and Structure Refinement Summaries for **9a-[Au]**, **9b-[Au],** and **9c-[Au]**.

Compound	9a-[Au]	9b-[Au]	9c-[Au]
Chemical formula	$C_{49}H_{37}AuClNS$	$C_{44}H_{35}AuClNS$	$C_{39}H_{33}AuClNS$
Formula weight	904.27	842.20	780.19
Crystal system, space group	Monoclinic, $P2_1/c$	Monoclinic, $I2/a$	Monoclinic, $P2_1/n$
Temperature (K)	101	101	100
a, *b*, *c* (Å)	17.0040 (2), 22.8804 (2), 21.3562 (4)	14.5394 (2), 17.8252 (2), 29.3972 (4)	10.1508 (1), 22.8766 (4), 14.0625 (2)
α (°)	90	90	90
β (°)	112.033 (2)	102.348 (1)	100.706 (1)
γ (°)	90	90	90
V (Å^3)	7702.0 (2)	7442.56 (17)	3208.69 (8)
Z	8	8	4
ρ_{calc} g/cm^3	1.560	1.503	1.615
Radiation type	Cu *K*α (λ = 1.54184)	Cu *K*α (λ = 1.54184)	Cu *K*α (λ = 1.54184)
μ (mm^{-1})	8.59	8.84	10.20
Crystal size (mm)	0.09 × 0.07 × 0.03	0.36 × 0.12 × 0.1	0.23 × 0.12 × 0.07
Diffractometer	XtaLAB Synergy, Dualflex, HyPix	XtaLAB Synergy, Dualflex, HyPix	XtaLAB Synergy, Dualflex, HyPix
Absorption correction	Multi-scan *CrysAlis PRO* 1.171.43.105a (Rigaku Oxford Diffraction, 2024) Spherical absorption correction using equivalent radius and absorption	Multi-scan *CrysAlis PRO* 1.171.43.120a (Rigaku Oxford Diffraction, 2024) Empirical absorption correction using spherical harmonics,	Multi-scan *CrysAlis PRO* 1.171.43.120a (Rigaku Oxford Diffraction, 2024) Empirical absorption correction using spherical harmonics,

	coefficient. Empirical absorption correction using spherical harmonics, implemented in SCALE3 ABSPACK scaling algorithm.	implemented in SCALE3 ABSPACK scaling algorithm.	implemented in SCALE3 ABSPACK scaling algorithm.
T_{min}, T_{max}	0.666, 0.687	0.205, 1.000	0.255, 1.000
No. of measured, independent and observed [$I \geq 2u(I)$] reflections	139273, 15383, 12280	59885, 7482, 6369	36067, 6381, 5887
R_{int}	0.109	0.107	0.054
(sin θ/λ)$_{max}$ (Å^{-1})	0.633	0.629	0.629
Final R indexes [I>=2σ (I)]	R_1 = 0.0552, wR_2 = 0.1303	R_1 = 0.0958, wR_2 = 0.2368	R_1 = 0.0404, wR_2 = 0.1044
Final R indexes [all data]	R_1 = 0.0712, wR_2 = 0.1413	R_1 = 0.1077, wR_2 = 0.2459	R_1 = 0.0431, wR_2 = 0.1062
Goodness of fit on F^2	1.027	1.060	1.0731
$R[F^2 > 2\sigma(F^2)]$, $wR(F^2)$, S	0.055, 0.141, 1.03	0.096, 0.246, 1.06	0.040, 0.106, 1.07
No. of reflections	15383	7482	6381
No. of parameters	951	405	391
H-atom treatment	H-atom parameters constrained<br>$w = 1/[\sigma^2(F_o^2) + (0.052P)^2 + 51.2473P]$ where $P = (F_o^2 + 2F_c^2)/3$	H-atom parameters constrained<br>$w = 1/[\sigma^2(F_o^2) + (0.0853P)^2 + 379.4062P]$ where $P = (F_o^2 + 2F_c^2)/3$	H-atom parameters constrained<br>$w = 1/[\sigma^2(F_o^2) + (0.0522P)^2 + 15.5758P]$ where $P = (F_o^2 + 2F_c^2)/3$
$\Delta\rho_{max}$, $\Delta\rho_{min}$ (e Å^{-3})	3.98, −1.97	5.88, −4.95	1.77, −1.49

Computer programs: *CrysAlis PRO* 1.171.42.58a (Rigaku OD, 2022), *SHELXL2018*/3 (Sheldrick, 2018), *SHELXTL* olex2.refine 1.5 (Bourhis *et al.*, 2015), Olex2 1.5 (Dolomanov *et al.*, 2009).

Table S2. Crystal Data and Structure Refinement Summaries for **9d-[Au]**, and **9f-[Au]**.

Parameter	**9d-[Au]**	**9f-[Au]**
Chemical formula	$C_{57}H_{41}AuClNS$	$C_{39}H_{33}AuClNS$
Formula weight	1004.38	780.14
Crystal system, space group	Triclinic, $P\bar{1}$	Triclinic, $P\bar{1}$
Temperature (K)	100	101
a, *b*, *c* (Å)	8.7115 (1), 24.1259 (4), 25.1427 (5)	8.5735 (2), 9.7268 (2), 20.3196 (3)
α (°)	112.807 (2)	79.369 (1)
β (°)	99.967 (2)	89.130 (1)
γ (°)	98.444 (1)	72.389 (2)
V (Å^3)	4662.55 (15)	1585.87 (6)
Z	4	2
ρ_{calc} g/cm^3	1.431	1.634
Radiation type	Cu *K*α (λ = 1.54184)	Cu *K*α (λ = 1.54184)
μ (mm^{-1})	7.16	10.32
Crystal size (mm)	0.16 × 0.02 × 0.01	0.30 × 0.09 × 0.02
Diffractometer	XtaLAB Synergy, Dualflex, HyPix	XtaLAB Synergy, Dualflex, HyPix
Absorption correction	Multi-scan *CrysAlis PRO* 1.171.42.102a (Rigaku Oxford Diffraction, 2023) Empirical absorption correction using spherical harmonics, implemented in SCALE3 ABSPACK scaling algorithm.	Multi-scan *CrysAlis PRO* 1.171.43.115a (Rigaku Oxford Diffraction, 2024) Empirical absorption correction using spherical harmonics, implemented in SCALE3 ABSPACK scaling algorithm.
T_{min}, T_{max}	0.694, 1.000	0.373, 1.000
No. of measured, independent and observed [$I > 2\sigma(I)$] reflections	125431, 18895, 14241	35382, 6309, 5953

R_{int}	0.102	0.075
(sin θ/λ)$_{max}$ (Å^{-1})	0.630	0.629
Final R indexes [I>=2σ (I)]	R_1 = 0.0526, wR_2 = 0.1329	R_1 = 0.0351, wR_2 = 0.0929
Final R indexes [all data]	R_1 = 0.0737, wR_2 = 0.1462	R_1 = 0.0371, wR_2 = 0.0946
Goodness of fit on F^2	1.035	1.074
$R[F^2 > 2\sigma(F^2)]$, $wR(F^2)$, S	0.053, 0.146, 1.03	0.035, 0.095, 1.07
No. of reflections	18895	6309
No. of parameters	1101	391
H-atom treatment	H-atom parameters constrained $w = 1/[\sigma^2(F_o^2) + (0.0781P)^2 + 6.9617P]$ where $P = (F_o^2 + 2F_c^2)/3$	H-atom parameters constrained $w = 1/[\sigma^2(F_o^2) + (0.0613P)^2 + 1.0855P]$ where $P = (F_o^2 + 2F_c^2)/3$
$\Delta\rho_{max}$, $\Delta\rho_{min}$ (e Å^{-3})	3.60, −2.64	2.04, −1.65

Computer programs: *CrysAlis PRO* 1.171.42.58a (Rigaku OD, 2022), *SHELXL2018*/3 (Sheldrick, 2018), *SHELXTL* olex2.refine 1.5 (Bourhis *et al.*, 2015), Olex2 1.5 (Dolomanov *et al.*, 2009).

3.2. ORTEP Structures of 9a-[Au], 9b-[Au], 9c-[Au], 9d-[Au] and 9f-[Au] (Figures S1-S5)

Figure S1. *ORTEP Structure of **9a-[Au]** (50% ellipsoids).* (Crystallographic data has been deposited with the Cambridge Crystallographic Data Center as supplementary publication no. CCDC 2467391).

I) 1st molecule, **9a1-[Au]**, Front View:

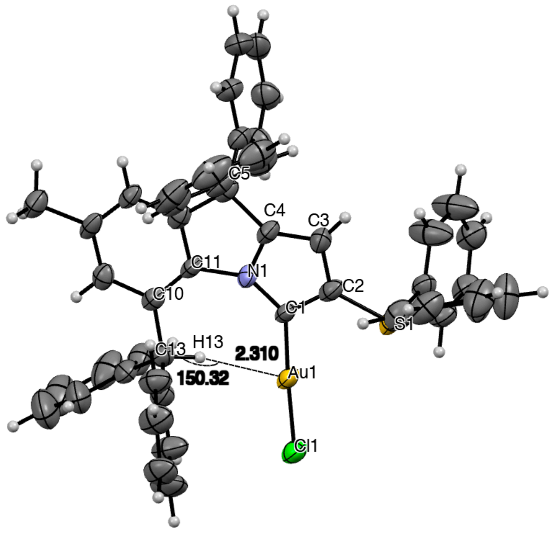

Side View:

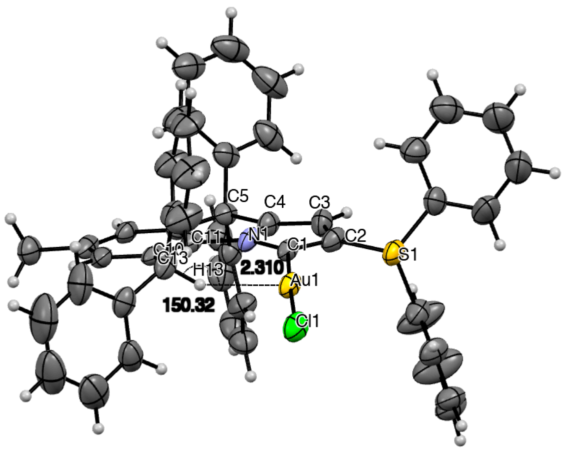

X-ray crystal structure of complex **9a1-[Au]**. Hydrogen atoms and solvents have been omitted for clarity. Selected bond lengths [Å] and angles [°]: Au1–Cl1, 2.289(2); Au1–C1, 2.012(7); C1–C2, 1.389(10); N1–C1, 1.376(8); S1–C2, 1.729(7), Au1–H13, 2.310; C1–Au1–C11, 177.38(19); N1–C1–C2 103.3(6); N1–C1–Au1, 134.1(5); C2–C1–Au1, 121.7(5); C13–H13–Au1 150.3.

II) 2nd molecule, **9a2-[Au]**

Front View:

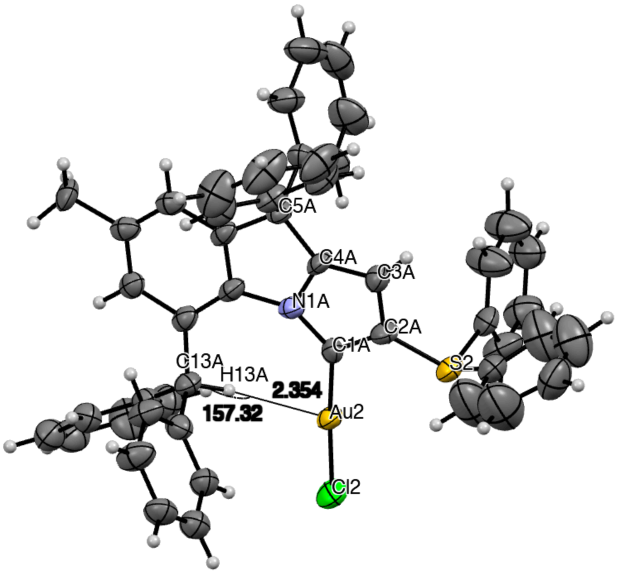

Side view:

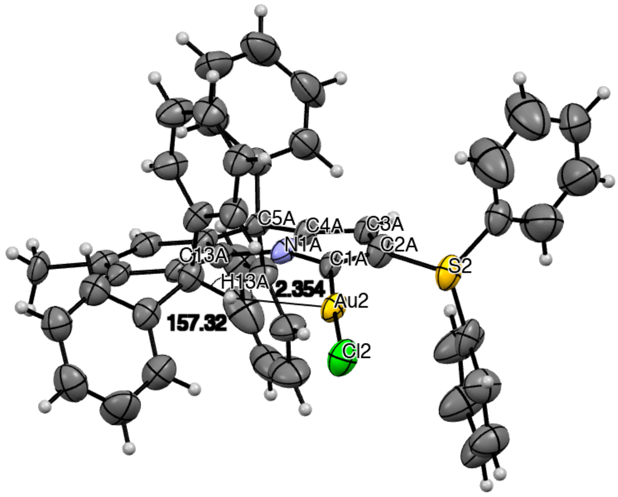

X-ray crystal structure of complex **9a2-[Au]**. Hydrogen atoms and solvents have been omitted for clarity. Selected bond lengths [Å] and angles [°]: Au2–Cl2, 2.275(2); Au2–C1A, 2.011(7); C1A–C2A, 1.379(10); N1A–C1A, 1.381(8); S2–C2A, 1.725(7); Au2–H13A, 2.354; C1A–Au2–Cl2, 176.86(19); C2A–C1A–N1A 103.8(6); N1A–C1A–Au2, 133.1(5); C2A–C1A–Au2, 122.4(5); C13A–H13A–Au1A 157.3.

Figure S2. *ORTEP Structure of* ***9b-[Au]*** *(50% ellipsoids)*. (Crystallographic data has been deposited with the Cambridge Crystallographic Data Center as supplementary publication no. CCDC 2467754).

Front view:

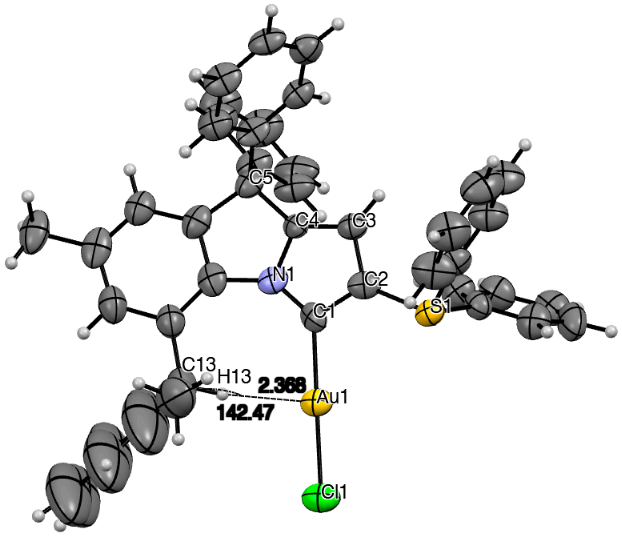

Side view:

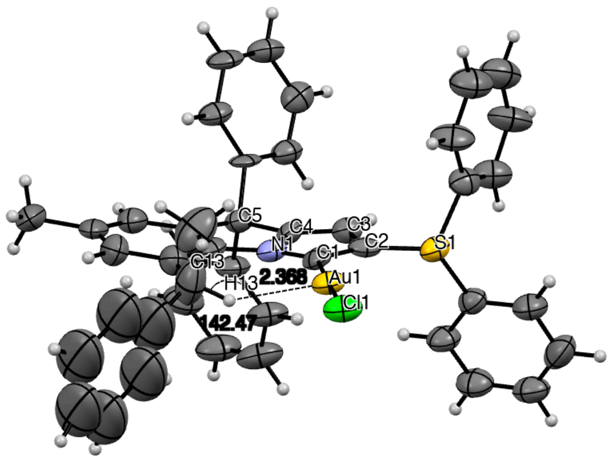

X-ray crystal structure of complex **9b-[Au]**. Hydrogen atoms and solvents have been omitted for clarity. Selected bond lengths [Å] and angles [°]: Au1–Cl1, 2.298(4); Au1–C1, 2.044(13); C1–C2, 1.398(17); N1–C1, 1.342(17); S1–C2, 1.735(13), Au1–H13, 2.368; Cl–Au1–C11, 178.7(4); N1–C1–C2 104.9(11); N1–C1–Au1, 135.8(9); C2–C1–Au1, 119.2(10); C13–H13–Au1 142.5.

Figure S3. *ORTEP Structure of **9c-[Au]** (50% ellipsoids).* (Crystallographic data has been deposited with the Cambridge Crystallographic Data Center as supplementary publication no. CCDC 2467756).

Front view:

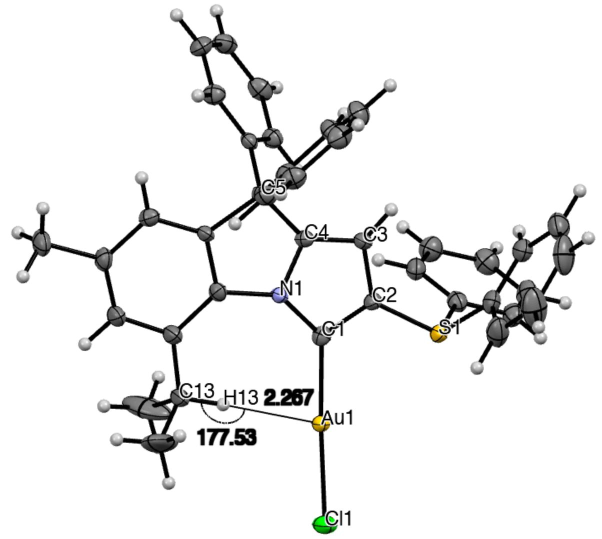

Side view:

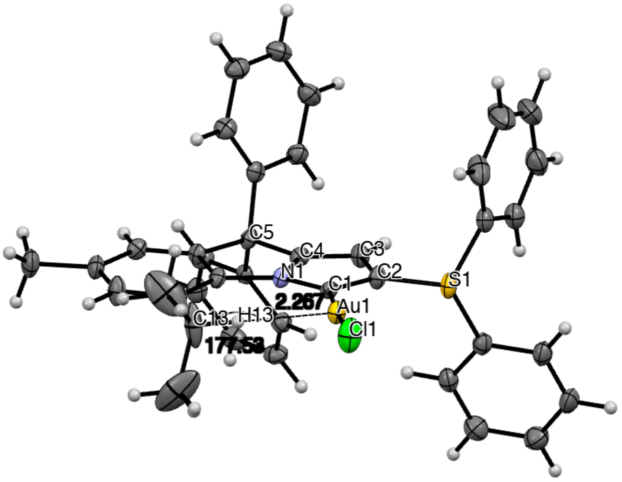

X-ray crystal structure of complex **9c-[Au]**. Hydrogen atoms and solvents have been omitted for clarity. Selected bond lengths [Å] and angles [°]: Au1–Cl1, 2.2885(13); Au1–C1, 1.993(5); C1–C2, 1.389(7); N1–C1, 1.383(6); S1–C2, 1.725(5), Au1–H13, 2.267; Cl–Au1–C11, 177.04(14); N1–C1–C2 103.2(4); N1–C1–Au1, 133.4(3); C2–C1–Au1, 123.1(4); C13–H13–Au1 177.5.

Figure S4. *ORTEP Structure of* ***9d-[Au]*** *(50% ellipsoids).* (Crystallographic data has been deposited with the Cambridge Crystallographic Data Center as supplementary publication no. CCDC 2456304).

I) 1st molecule, **9d1-[Au]**, Front view:

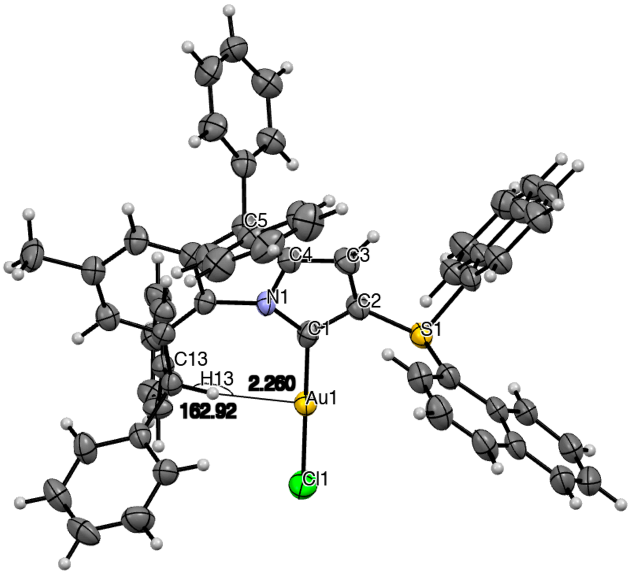

Side view:

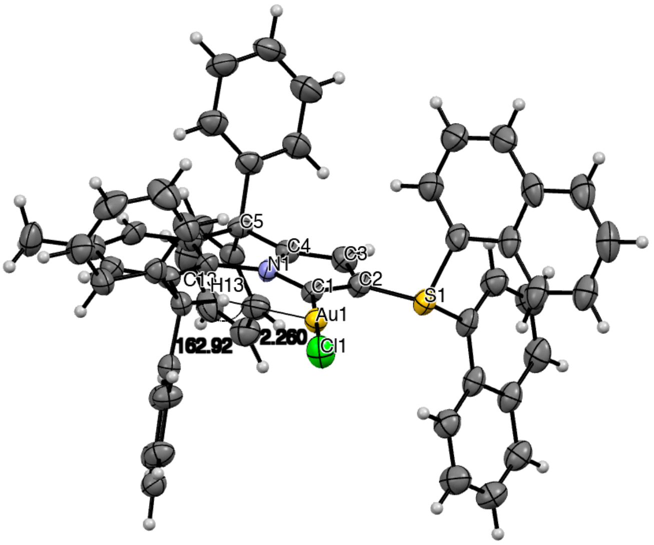

X-ray crystal structure of complex **9d1-[Au]**. Hydrogen atoms and solvents have been omitted for clarity. Selected bond lengths [Å] and angles [°]: Au1–Cl1, 2.307(17); Au1–C1, 1.993(6); C1–C2, 1.408(8); N1–C1, 1.401(7); S1–C2, 1.727(6), Au1–H13, 2.260; C1–Au1–C11, 179.0(17); N1–C1–C2 102.9(5); N1–C1–Au1, 132.7(4); C2–C1–Au1, 124.3(4); C13–H13–Au1 162.9.

II) 2nd molecule, **9d2-[Au]**

Front view:

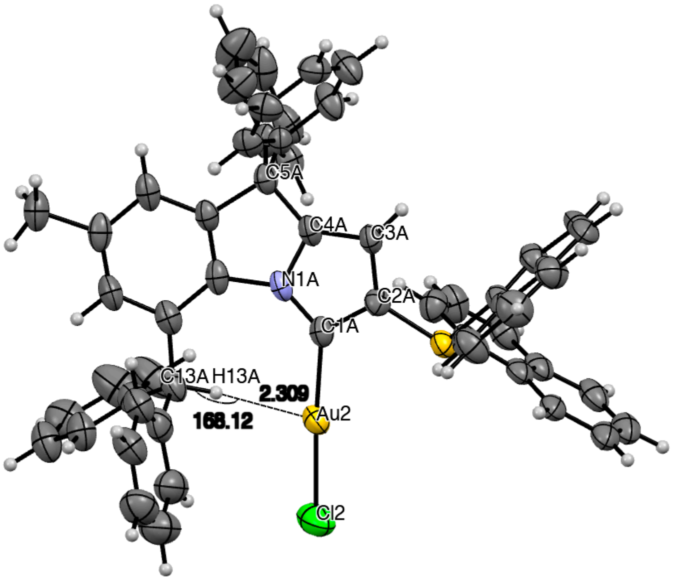

Side view:

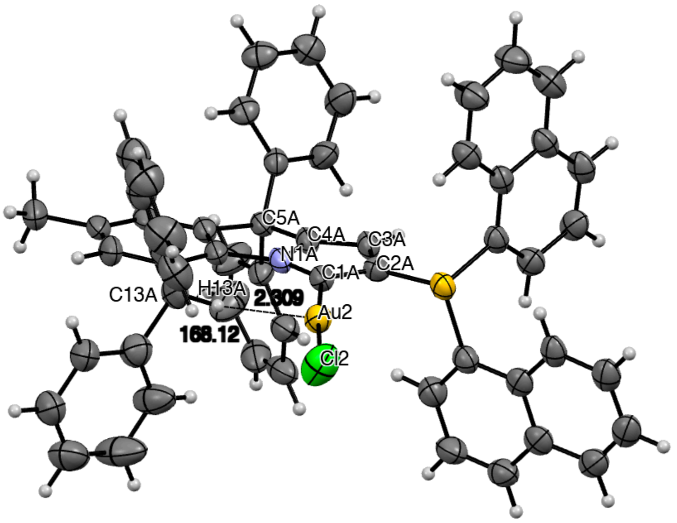

X-ray crystal structure of complex **9d2-[Au]**. Hydrogen atoms and solvents have been omitted for clarity. Selected bond lengths [Å] and angles [°]: Au2–Cl2, 2.280(2); Au2–C1A, 2.013(7); C1A–C2A, 1.398(8); N1A–C1A, 1.367(8); S2–C2A, 1.734(6); Au2–H13A, 2.309; C1A–Au2–Cl2, 176.42(17); C2A–C1A–N1A 103.3(5); N1A–C1A–Au2, 135.2(4); C2A–C1A–Au2, 121.2(5); C13A–H13A–Au1A 168.1.

Figure S5. *ORTEP Structure of* ***9f-[Au]*** *(50% ellipsoids).* (Crystallographic data has been deposited with the Cambridge Crystallographic Data Center as supplementary publication no. CCDC 2467757).

Front view:

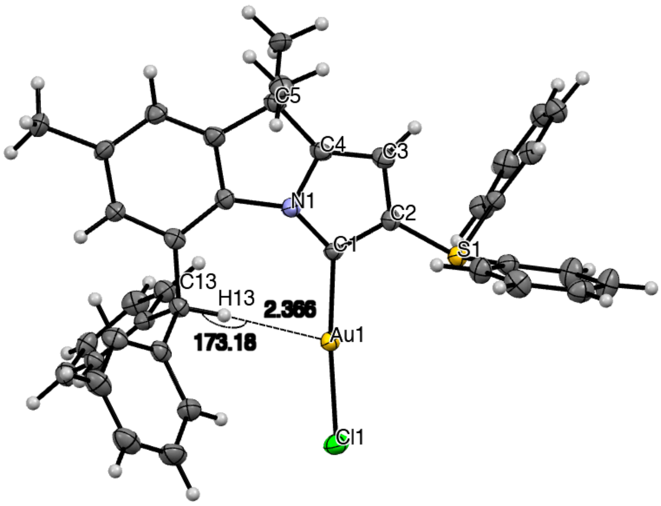

Side view:

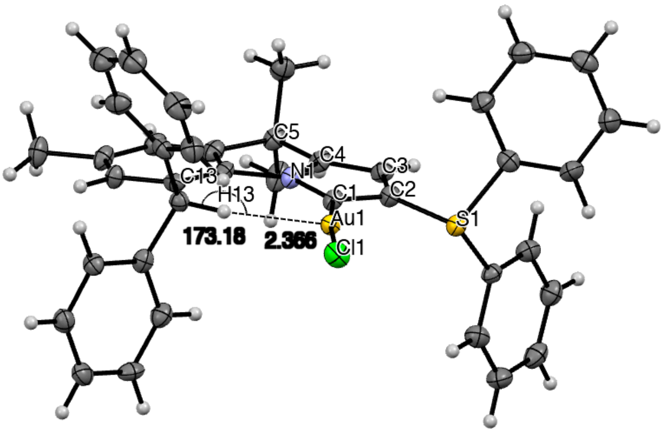

X-ray crystal structure of complex **9f-[Au]**. Hydrogen atoms and solvents have been omitted for clarity. Selected bond lengths [Å] and angles [°]: Au1–Cl1, 2.2907(10); Au1–C1, 1.992(4); C1–C2, 1.409(5); N1–C1, 1.382(5); S1–C2, 1.734(4), Au1–H13, 2.366; Cl–Au1–C11, 174.68(11); N1–C1–C2 102.9(3); N1–C1–Au1, 135.7(3); C2–C1–Au1, 121.0(3); C13–H13–Au1 173.2.

3.3. X-Ray Crystallographic Comparison

Table S2. Detailed comparison of complexes **9a-[Au]**, **9b-[Au]**, **9c-[Au]**, **9d-[Au]**, **9f-[Au]**.

		Au1–Cl1	Au1–C1	C1–C2	N1–C1	S1–C2	Au1–H13	C1–Au1–C11	N1–C1–C2	N1–C1–Au1	C2–C1–Au1	C13–H13–Au1
9a-[Au]	9a1-[Au]	2.289(2)	2.012(7)	1.389(10)	1.376(8)	1.729(7)	2.310	177.38(19)	103.3(6)	134.1(5)	121.7(5)	150.3
	9a2-[Au]	2.275(2)	2.011(7)	1.379(10)	1.381(8)	1.725(7)	2.354	176.86(19)	103.8(6)	133.1(5)	122.4(5)	157.3
9b-[Au]		2.298(4)	2.044(13)	1.398(17)	1.342(17)	1.735(13)	2.368	178.7(4)	104.9(11)	135.8(9)	119.2(10)	142.5
9c-[Au]		2.2885(13)	1.993(5)	1.389(7)	1.383(6)	1.725(5)	2.267	177.04(14)	103.2(4)	133.4(3)	123.1(4)	177.5
9d-[Au]	9d1-[Au]	2.307(17)	1.993(6)	1.408(8)	1.401(7)	1.727(6)	2.260	179.0(17)	102.9(5)	132.7(4)	124.3(4)	162.9
	9d2-[Au]	2.280(2)	2.013(7)	1.398(8)	1.367(8)	1.734(6)	2.309	176.42(17)	103.3(5)	135.2(4)	121.2(5)	168.1
9f-[Au]		2.2907(10)	1.992(4)	1.409(5)	1.382(5)	1.734(4)	2.366	174.68(11)	102.9(3)	135.7(3)	121.0(3)	173.2

3.4. Topographical Steric Maps from Crystallographic Data

Figure S6. Topographical steric maps of **9a-[Au]**, **9b-[Au]**, **9c-[Au]**, **9d-[Au]**, **9f-[Au]** showing % V_{bur} per quadrant.

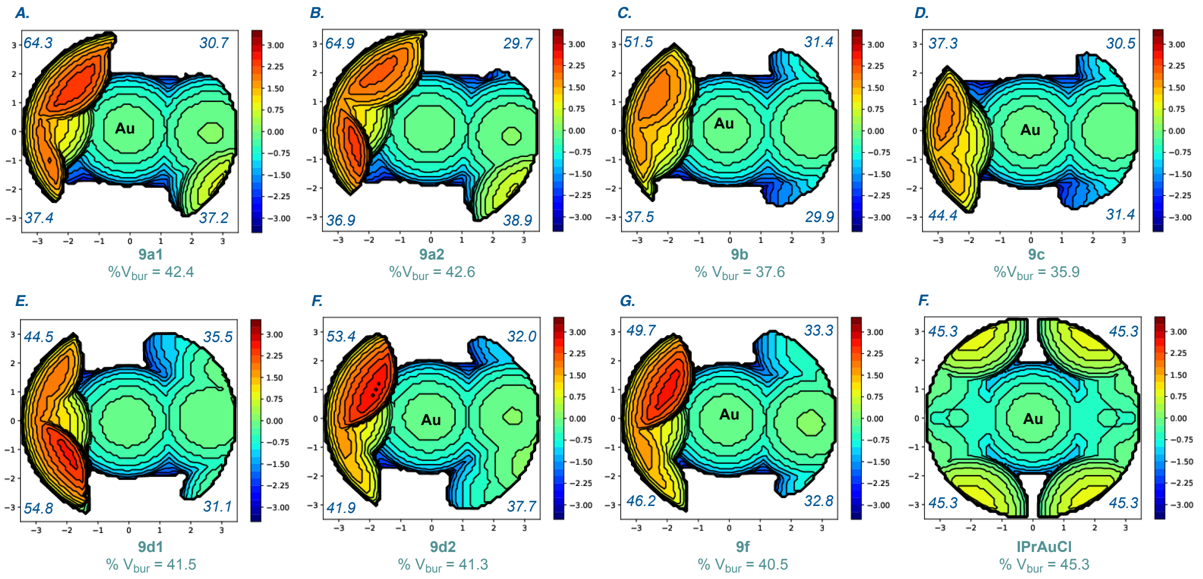

Table S3. Distribution of %V_{bur} in different directions in topographical steric maps of compound **9a-[Au]**, **9b-[Au]**, **9c-[Au]**, **9d-[Au]**, **9f-[Au]**.

Compound	%V_{bur} (%)	SW (%)	NW (%)	NE (%)	SE (%)
**9a1-[Au]**	*42.4*	*37.4*	*64.3*	*30.7*	*37.2*
**9a2-[Au]**	*42.6*	*36.9*	*64.9*	*29.7*	*38.9*
**9b-[Au]**	*37.6*	*37.5*	*51.5*	*31.4*	*29.9*
**9c-[Au]**	*35.9*	*44.4*	*37.3*	*30.5*	*31.4*
**9d1-[Au]**	*41.5*	*54.8*	*44.5*	*35.5*	*31.1*
**9d2-[Au]**	*41.3*	*41.9*	*53.4*	*32.0*	*37.7*
**9f-[Au]**	*40.5*	*46.2*	*49.7*	*33.3*	*32.8*

3.5. The Torsional Angle Between Carbene Plane and Analogous C-H Plane

In ligand **8a**, the torsional angle between the carbene plane and the C-H plane is ~45°. However, in [Au]-complex, the angles come down to ~21°, which means they are more aligned to the Au centre.

Figure S7. Torsional angle difference between carbene plane and analogous C-H plane

8a **9a-[Au]**

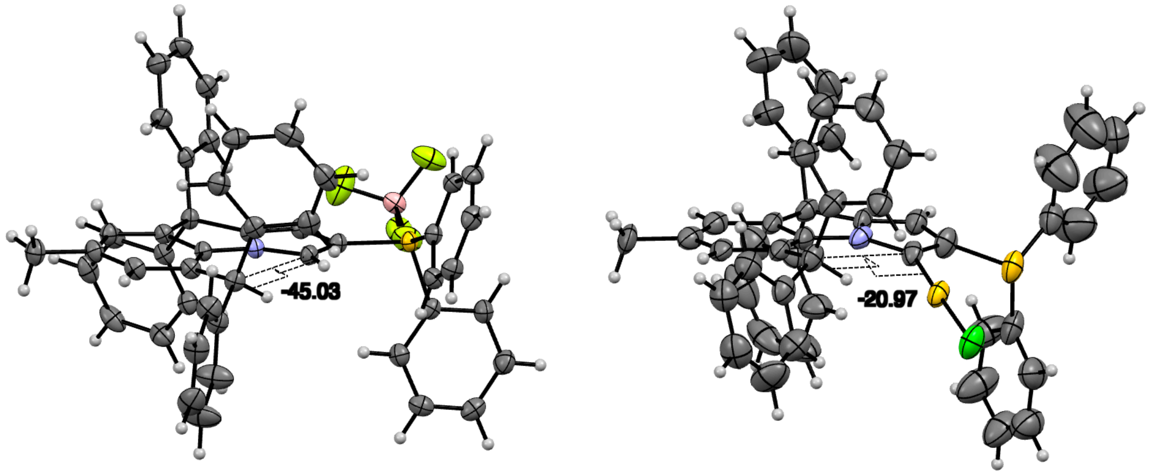

4. NMR Studies

4.1. C−H···Au(I) Interactions in Solution via NMR Spectroscopy Study

Figure S8. Downfield shift in ^{1}H NMR for complexes **9a-[Au]**, with respect to corresponding ligands **8a** in Δδ ppm.

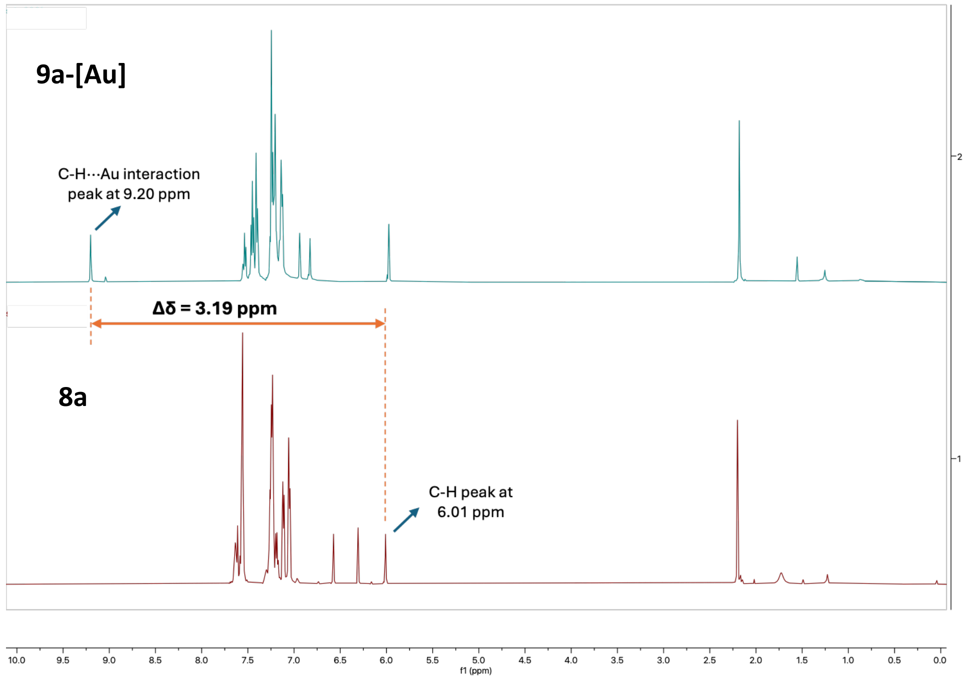

Figure S9. Downfield shift in ^{1}H NMR for complexes **9b-[Au]**, with respect to corresponding ligands **8b** in Δδ ppm.

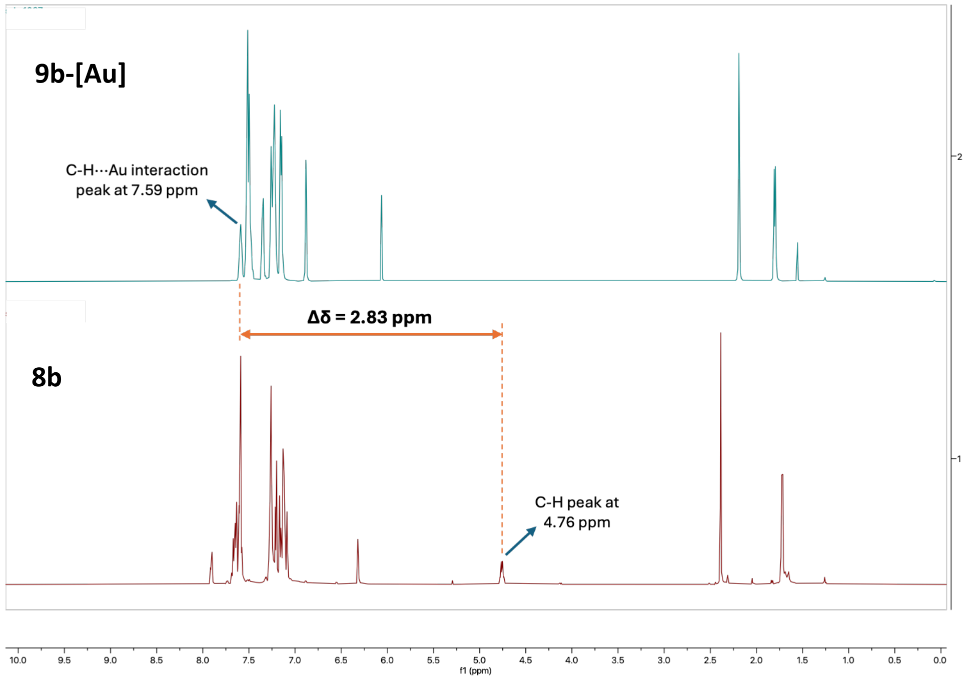

Figure S10. Downfield shift in ^{1}H NMR for complexes **9c-[Au]**, with respect to corresponding ligands **8c** in Δδ ppm.

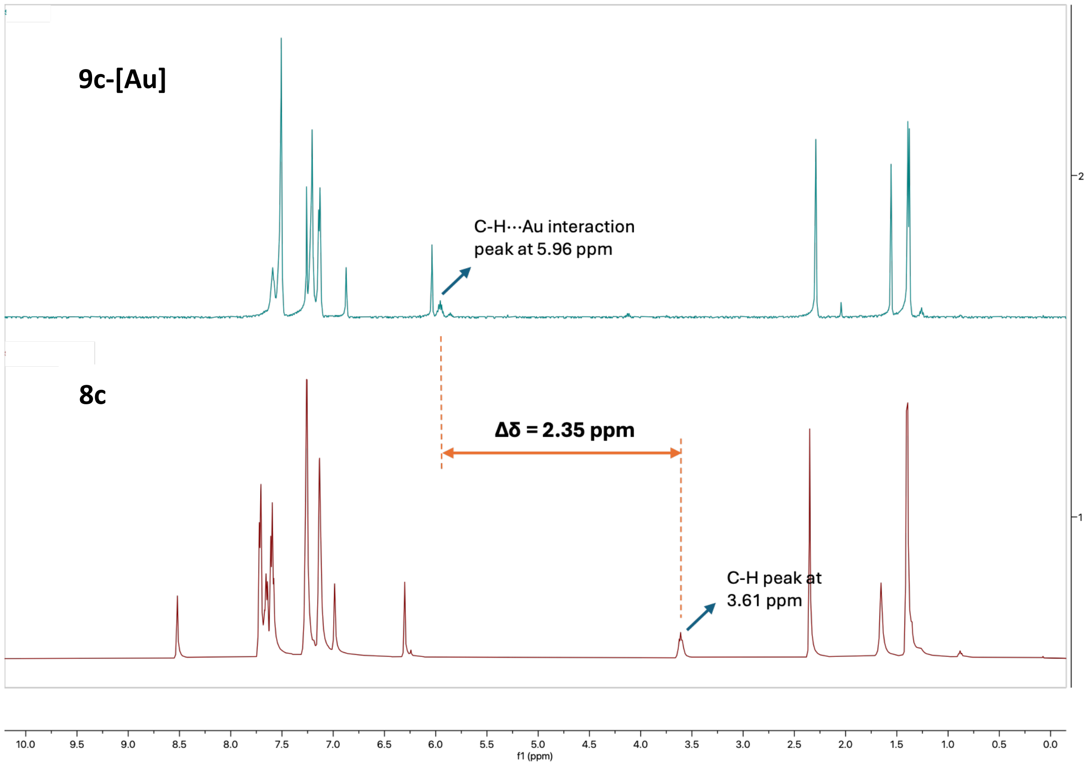

4.2. ^{1}H NMR Analysis of Cationic Au-Complex 9a'-[Au]

An oven-dried 10 mL reaction tube equipped with a stir bar was charged with complex **9a-[Au]** (18 mg, 0.02 mmol) and silver hexafluoroantimonate, $AgSbF_6$ (6.9 mg, 0.02 mmol) in 0.75 mL $CDCl_3$. The mixture was stirred at room temperature for 5 minutes and subjected to ^{1}H NMR analysis.

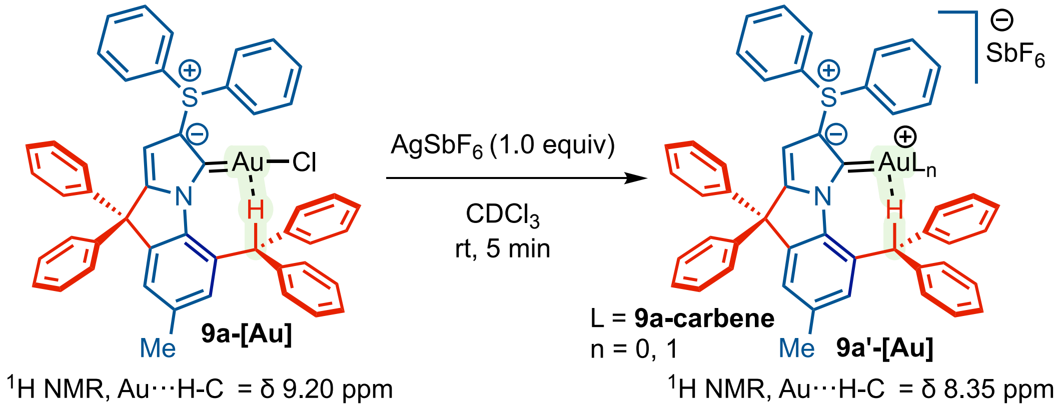

Figure S11: ^{1}H NMR study of Au···H-C bond between **9a-[Au]** and its possible cationic complex **9a'-[Au]**

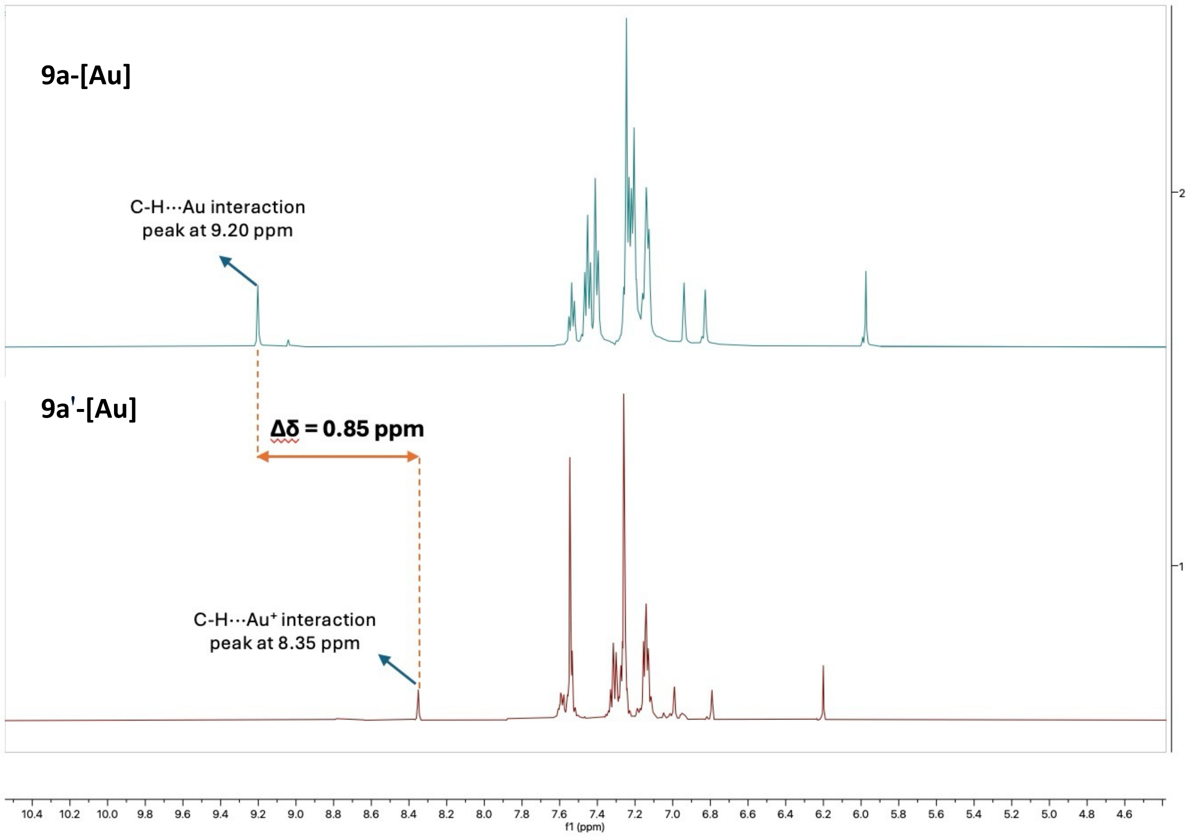

4.3a. Measurement of σ-donating capability ($^1J_{CH}$) through ^{1}H NMR Analysis of 8a in $CDCl_3$

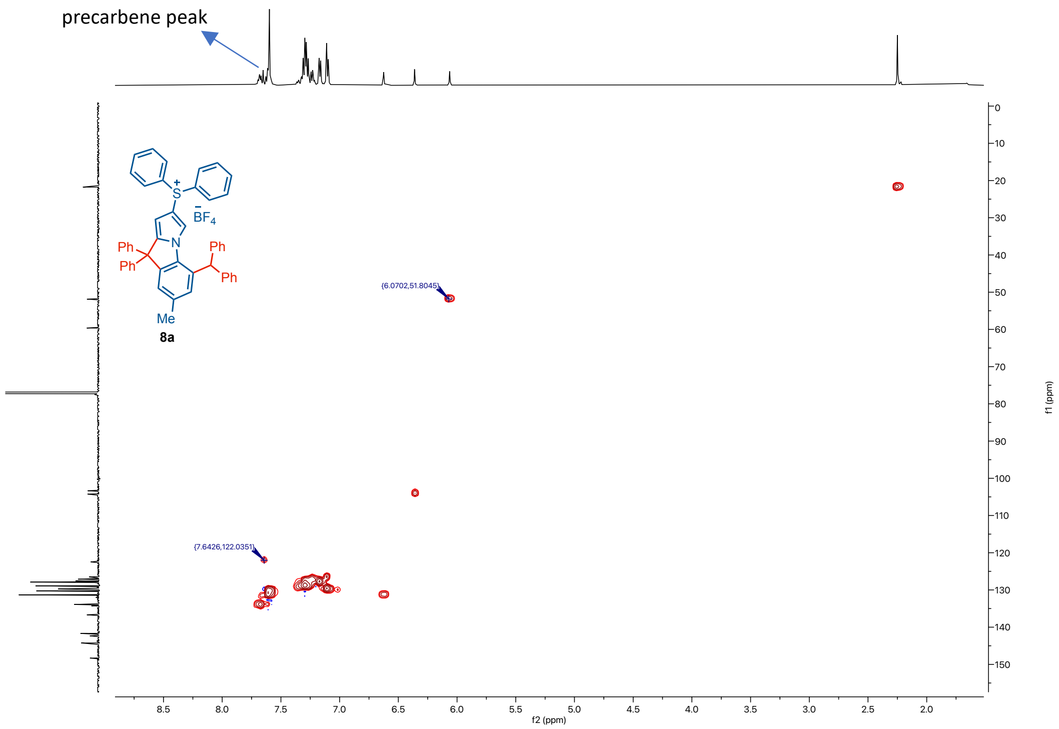

^{1}H^{13}C-HSQC-NMR spectrum of **8a** in $CDCl_3$

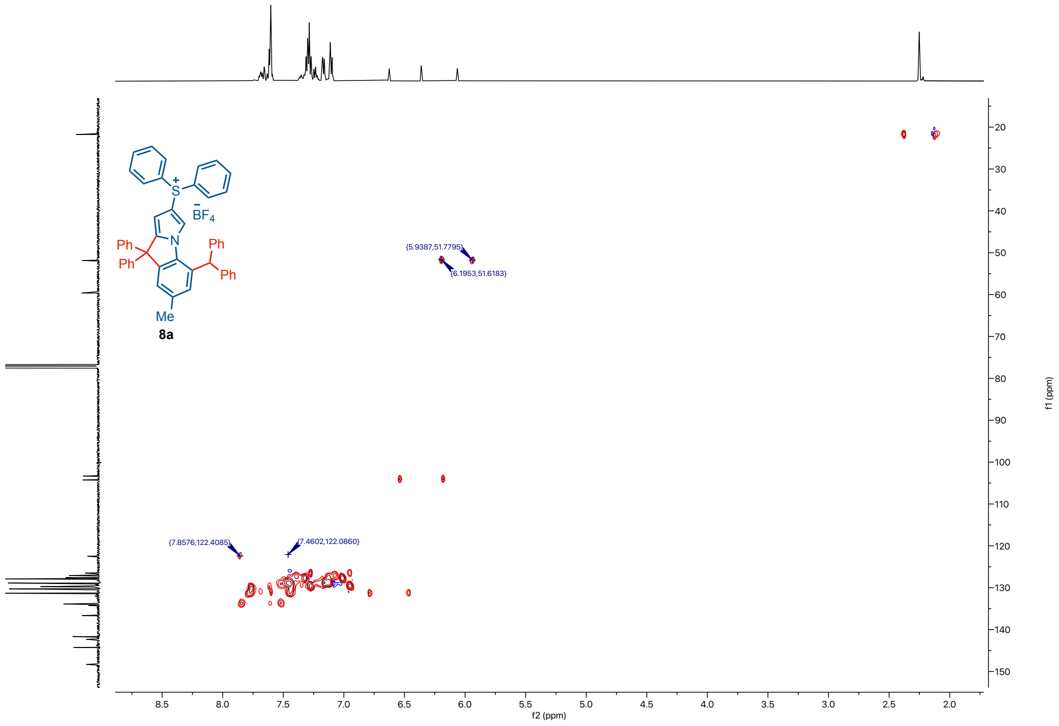

^{1}H^{13}C HSQC (coupled) NMR spectrum of **8a** in $CDCl_3$

In $CDCl_3$, the $^1J_{CH}$ value for precarbene centre was calculated as 198.2 Hz. The $^1J_{CH}$ value for the C(13)-H(13) for ligand **8a** was calculated as 128.3 Hz.

4.3b. Measurement of σ-donating capability ($^{1}J_{CH}$) through ^{1}H NMR Analysis of 8a in D6-DMSO

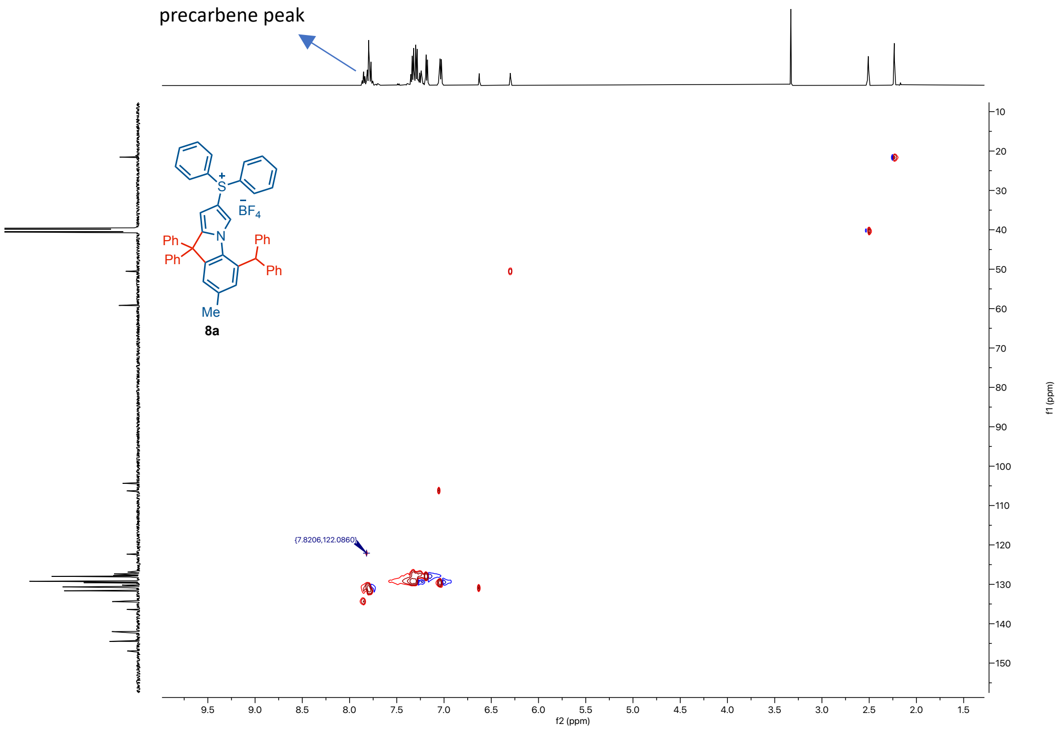

^{1}H^{13}C-HSQC-NMR spectrum of **8a** in D$_6$-DMSO

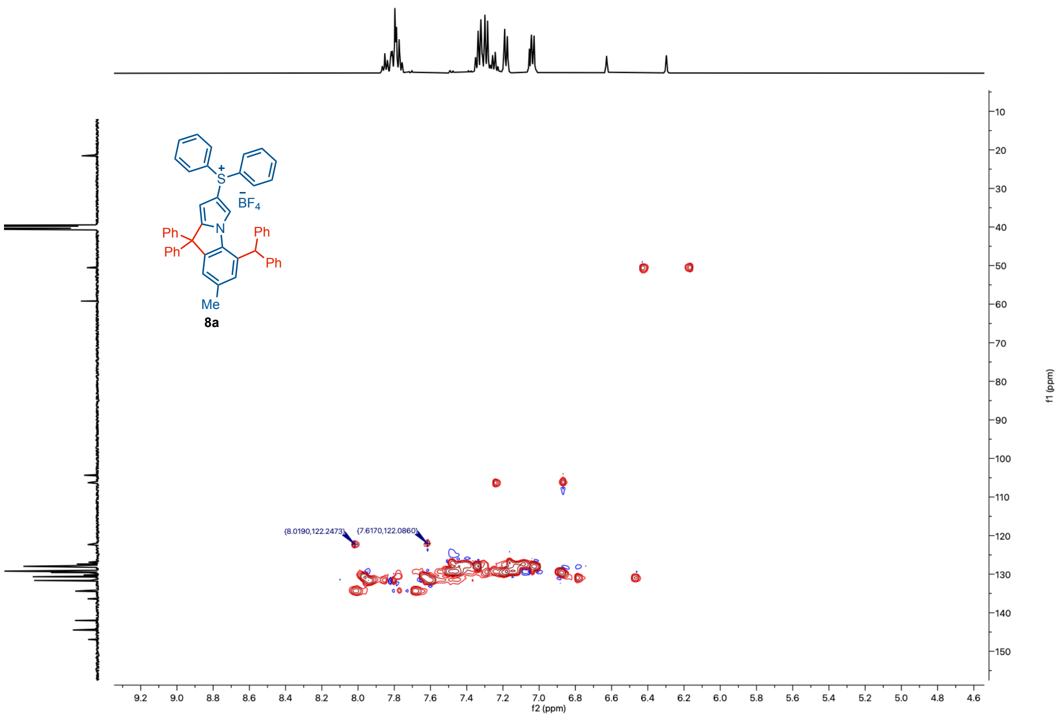

^{1}H^{13}C HSQC (coupled) NMR spectrum of **8a** in D$_6$-DMSO

In D$_6$-DMSO, the $^{1}J_{CH}$ value for precarbene centre was calculated as 201.0 Hz.

4.4. Measurement of $^1J_{CH}$ at C(13)-H(13) in complex 9a-[Au] through ^{1}H NMR Analysis in $CDCl_3$

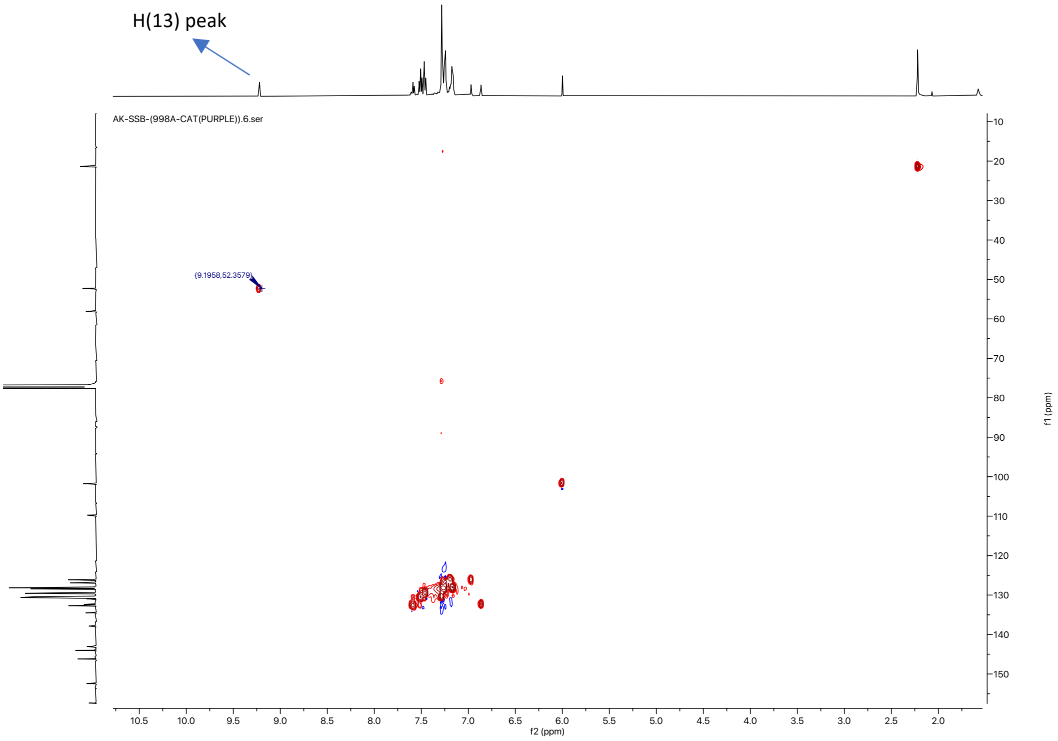

^{1}H^{13}C-HSQC-NMR spectrum of **9a-[Au]** in $CDCl_3$

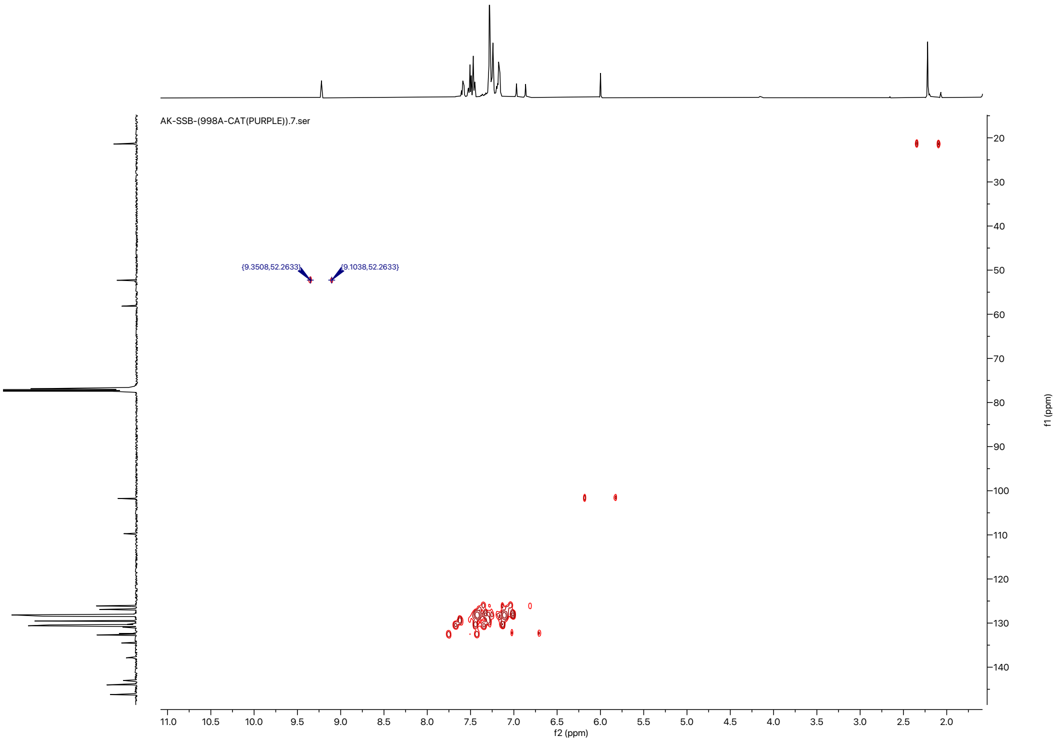

^{1}H^{13}C HSQC (coupled) NMR spectrum of **8a** in $CDCl_3$

The $^1J_{CH}$ value for the C(13)-H(13) for complex **9a** was calculated as 123.5 Hz.

4.3. Variable Temperature Experiment

In an oven-dried NMR tube, complex **9a-[Au]** was dissolved in 0.6 mL of $CDCl_3$ solvent and the ^{1}H NMR was taken at variable temperature at 55 °C, 45 °C, 35 °C, 25 °C, 15 °C, 0 °C, -10 °C, -20 °C, -30 °C, -41 °C, -54 °C, -65 °C, -75 °C.

Figure S12: Shift in ^{1}H NMR of Au···H-C peak at variable temperature

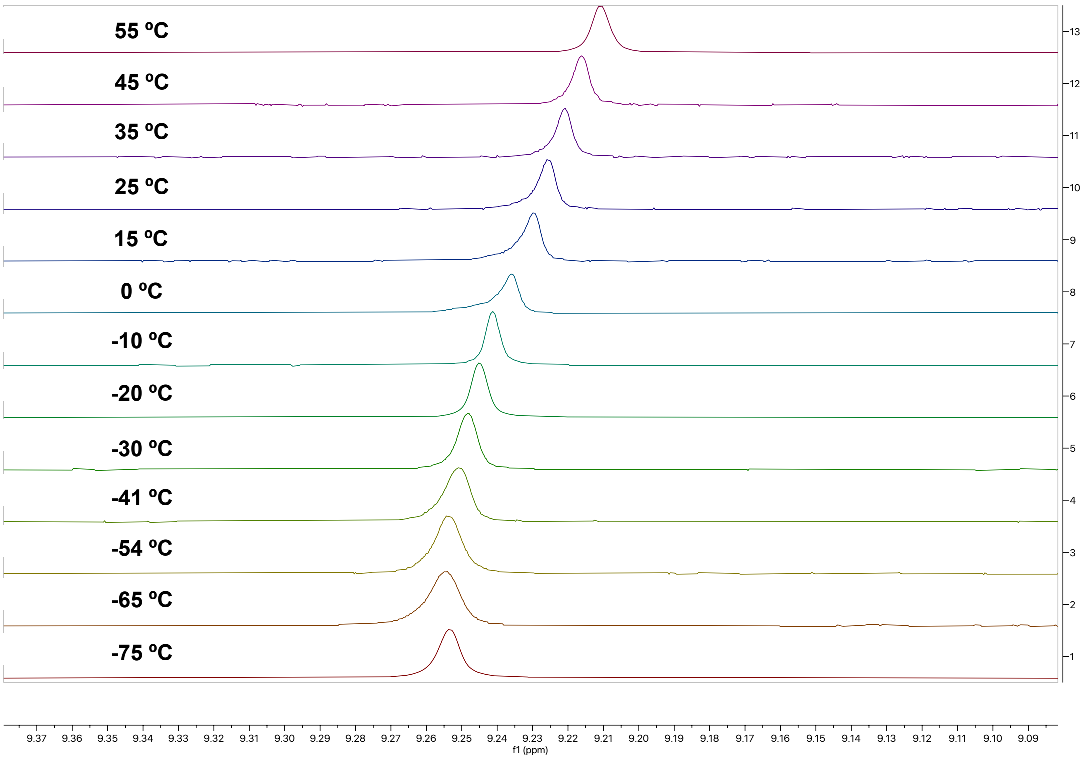

Typically, as the temperature rises, the strength of hydrogen bonding tends to diminish, which can be attributed to the increased vibrational motion and kinetic energy present in the system.

5. Computational Methods

Computational Methods. All of the calculations were performed using Gaussian 16 suite of programs. All of the geometry optimizations were performed at the B3LYP level of theory in the gas phase with the QZVP basis set for gold and the 6-311++G(d,p) basis set for the other atoms using Grimme D3 correction. For geometry optimizations, we employed the X-ray structures of [Au(NHC)Cl] complexes of *9*H-pyrrolo[1,2-*a*]indol-3-ylidenes **9a-[Au]**–**9c-[Au]** as the starting geometry and performed full optimization. The absence of imaginary frequencies was used to characterize the structures as minima on the potential energy surface. All of the optimized geometries were verified as minima (no imaginary frequencies). NBO calculations were performed at the DFT/B3LYP level using NBO program implemented in Gaussian software package. Wiberg bond indices were calculated by the NBO method. Energetic parameters were calculated under standard conditions (298.15 K and 1 atm). Quantum theory of atoms in molecule and noncovalent interaction analyses were performed for the optimized structures using Multiwfn to depict the topological properties of the complexes and results were visualized using the VMD 1.9.4a51 software.[7-10] Structural representations were generated using CYLview software (Legault, C. Y. CYLview version 1.0 BETA, University of Sherbrooke). All other representations were generated using GaussView (GaussView, version 5, Dennington, R.; Keith, T.; Millam, J. Semichem Inc., Shawnee Mission, KS, 2009) or ChemCraft software (Andrienko, G. L. ChemCraft version b562a, https://www.chemcraftprog.com).

5.1. Atoms In Molecules (AIM), Noncovalent Interaction (NCI) and Natural Bond Orbital (NBO) Analysis

Table S4. AIM data

	$d_{C\text{-}H}$	$d_{H\text{-}Au}$	ρ(r)	∇²ρ(r)	*Potential Energy V*	E (KJ/mol)	*ν* (CH)	*Δν* (CH)
**9a-[Au]**	1.0960	2.3113	0.0275	0.0695	-0.01946	-25.5	2969.3	-
**9a-carbene**	1.0946						3028.0	58.7
**9b-[Au]**	1.0949	2.3253	0.0268	0.0685	-0.01877	-24.6	2988.8	-
**9b-carbene**	1.0944						3032.5	43.7
**9c-[Au]**	1.0948	2.3663	0.0249	0.0651	-0.01713	-22.5	2997.1	-
**9c-carbene**	1.0918						3049.3	52.1

Results of the computational studies on the secondary ligand gold interactions in the gold complexes **9a-[Au]**, **9b-[Au]**, **9c-[Au]**, and their corresponding free carbene ligand. For further details, see the Supporting Information. Calculations were performed at the B3LYP level of theory in the gas phase with the QZVP basis set for gold and the 6-311++G(d,p) basis set for the other atoms using Grimme D3 correction.

Table S5. NBO study and Wiberg bond order

	C-H bond order	H-Au bond order
**9a-[Au]**	0.8419	0.0399
**9a-carbene**	0.8553	
**9b-[Au]**	0.8557	0.0363
**9a-carbene**	0.8622	
**9c-[Au]**	0.8670	0.0312
**9a-carbene**	0.8709	

NBO calculations were performed at the DFT/B3LYP level using the NBO program implemented in Gaussian software package.

Figure S13: Contour plot of the Laplacian distribution ∇2ρ(r) for **9a-[Au]** with relevant bond paths and BCPs (blue spheres). Hydrogen atoms have been omitted for clarity, except for that on the carbon atom (13C) related to H-Au bonding

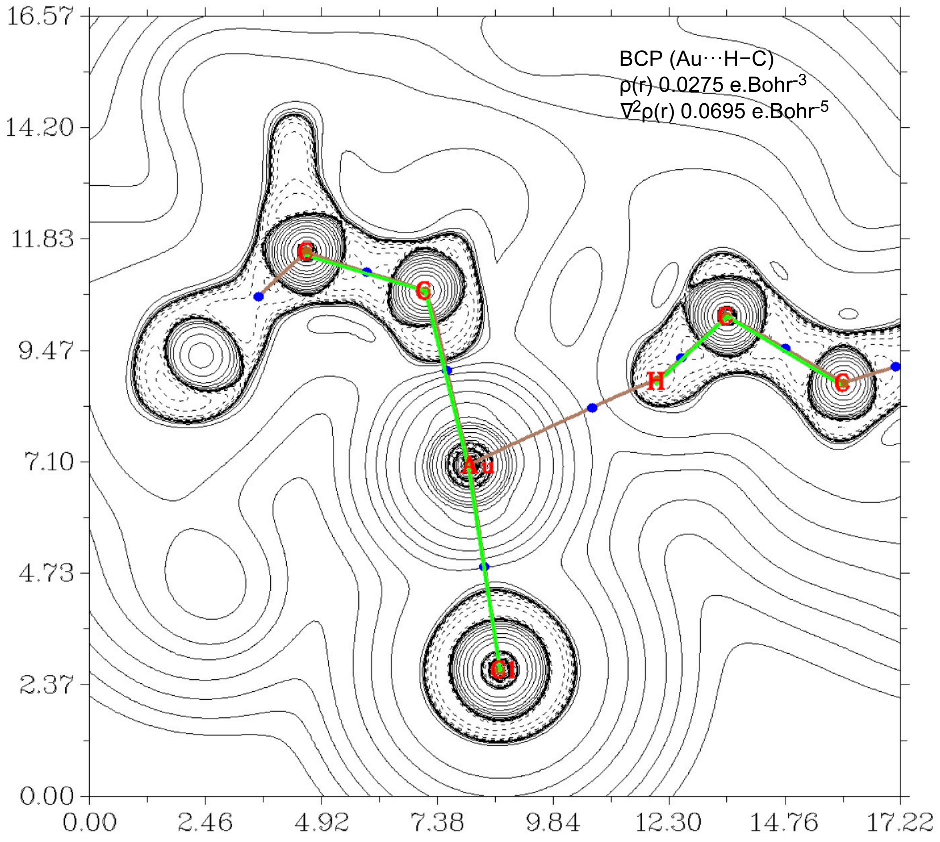

Figure S14: Coloured in a blue-green-red scheme over the range of (-0.05 < sign(λ_2)ρ <0.05) and isosurface of RDG=0.5. Blue indicates strong attraction, green indicates weak interaction, and red indicates repulsion.

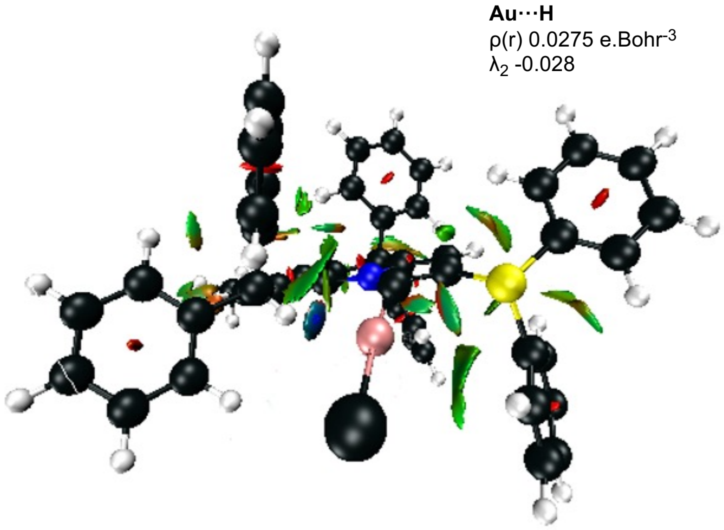

5.2. HOMO-LUMO Orbitals and Corresponding Energy Levels

Figure S15: HOMO orbitals and orbital energies (eV) of of **9a-[Au]**, **9b-[Au]** and **9c-[Au]**. B3LYP 6-311++g(d,p) level.

9a-[Au]

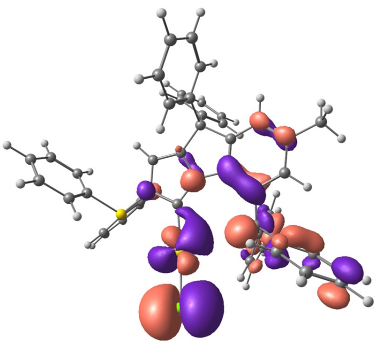

HOMO-1
(-5.93 eV)

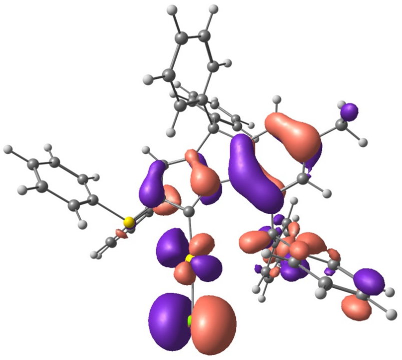

HOMO-2
(-6.05 eV)

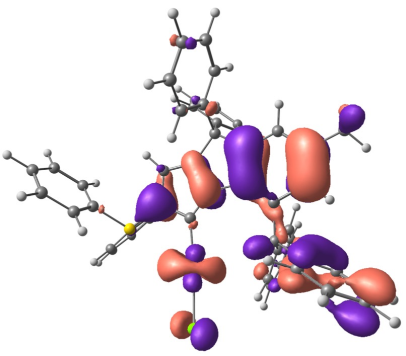

HOMO-3
(-6.20 eV)

9b-[Au]

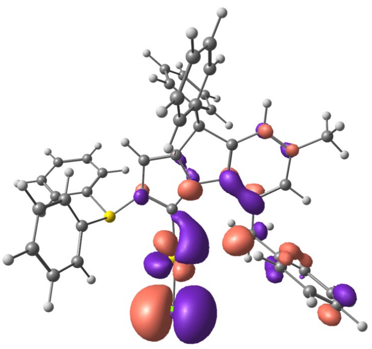

HOMO-1
(-5.95 eV)

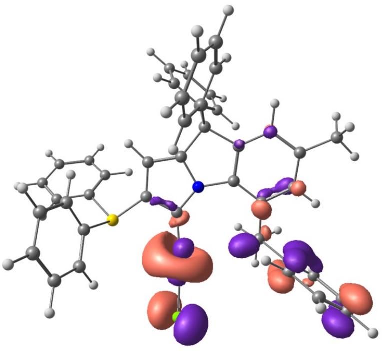

HOMO-3
(-6.16 eV)

9c-[Au]

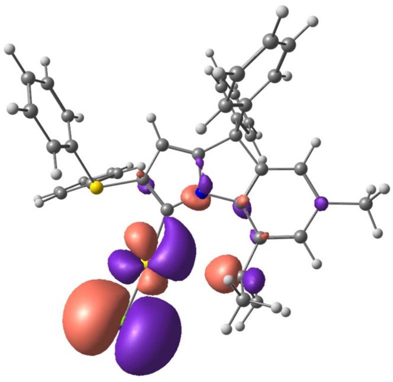

HOMO-1
(-5.90 eV)

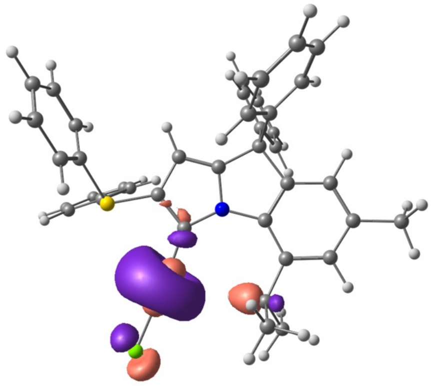

HOMO-3
(-6.18 eV)

Figure S16: HOMO and LUMO orbitals and orbital energies (eV) of **9a-carbene**, **9b-carbene** and **9c-carbene**. B3LYP 6-311++g(d,p) level.

9a-carbene

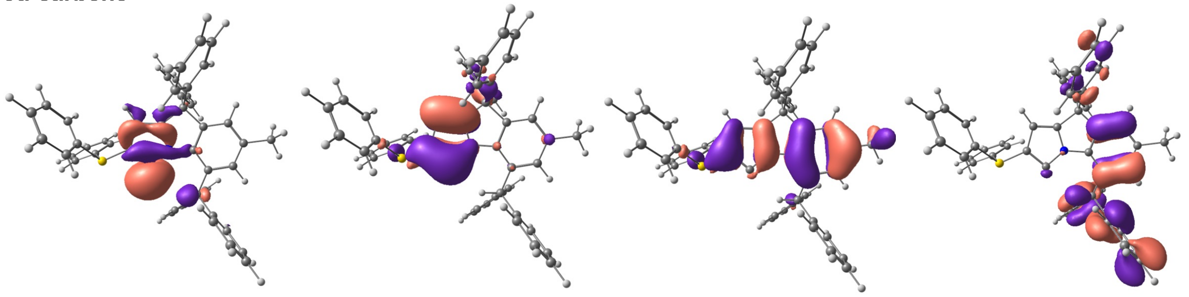

HOMO (-5.07 eV)	HOMO-1 (-5.43 eV)	HOMO-2 (-5.50 eV)	HOMO-3 (-6.09 eV)

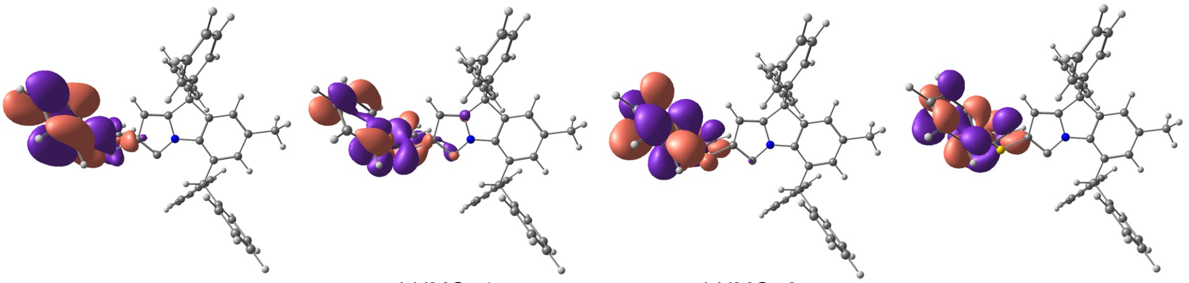

LUMO (-1.62 eV)	LUMO+1 (-1.45 eV)	LUMO+2 (-1.36 eV)	LUMO+3 (-1.00 eV)

9b-carbene

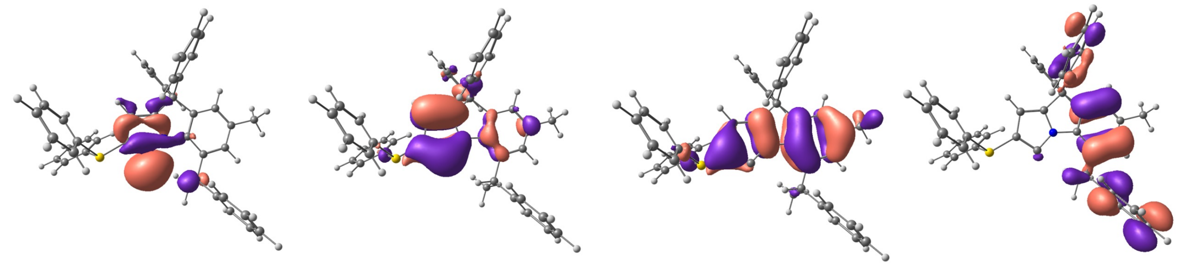

HOMO (-5.04 eV)	HOMO-1 (-5.40 eV)	HOMO-2 (-5.48 eV)	HOMO-3 (-6.11 eV)

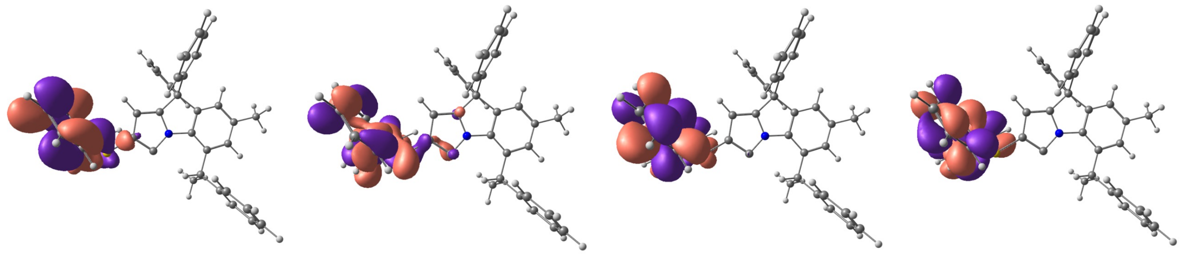

LUMO (-1.61 eV)	LUMO+1 (-1.45 eV)	LUMO+2 (-1.36 eV)	LUMO+3 (-1.01 eV)

9c-carbene

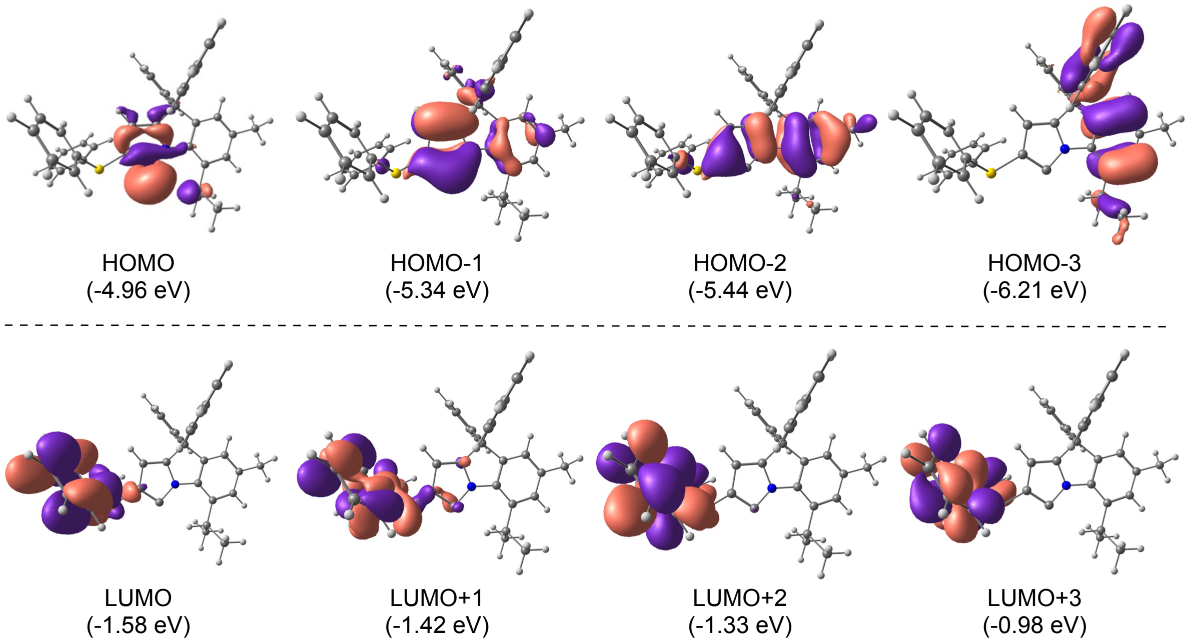

Figure 17. HOMO and LUMO energy levels (eV) of **9a-carbene**, **9b-carbene**, **9c-carbene** and related NHC Ligands at B3LYP 6-311++g(d,p) level.

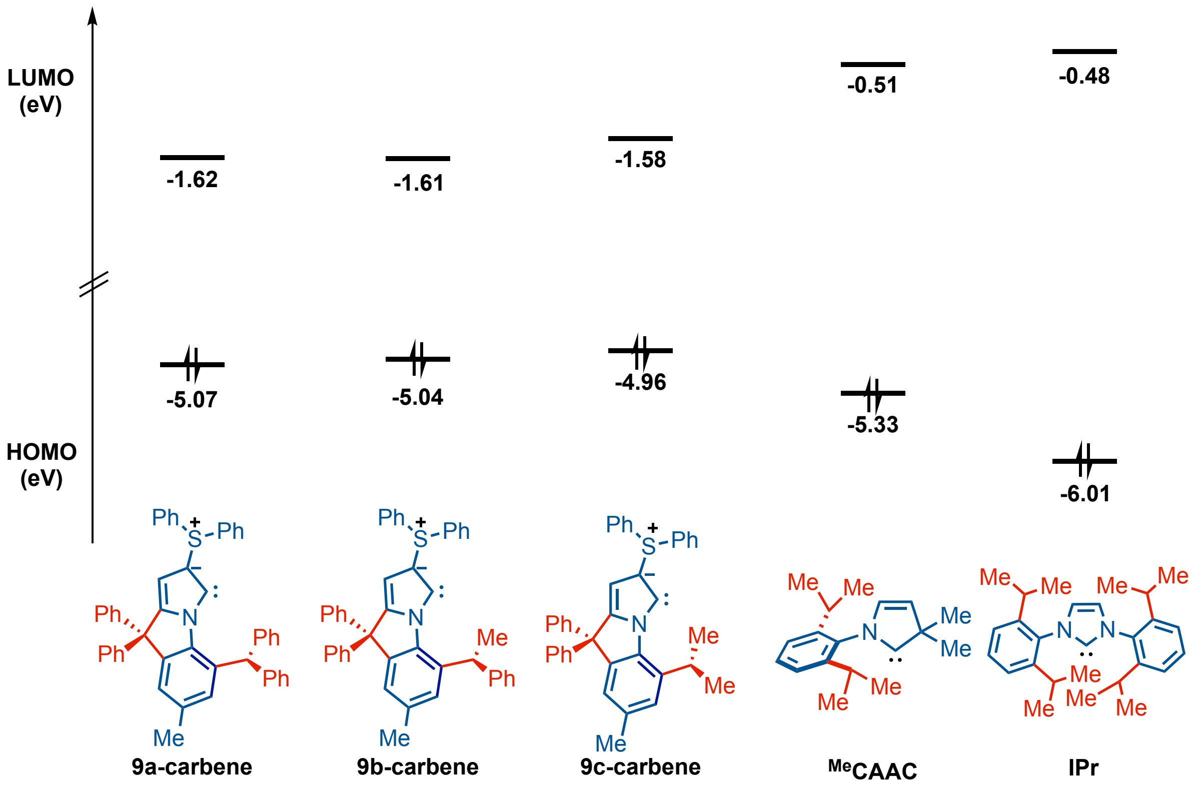

Table S6. HOMO and LUMO Energy Levels of **9a-carbene**, **9b-carbene**, **9c-carbene** and related NHC Ligands. Calculated at the B3LYP/6-311++g(d,p) Level[a,b]

entry	compound	orbital	E [au]	E [eV]	E [kcal/mol]	[eV]
1.	**9a-carbene**	HOMO	-0.1862	-5.07	-116.84	
2.	**9a-carbene**	LUMO	-0.0596	-1.62	-37.40	-3.45
3.	**9b-carbene**	HOMO	-0.1851	-5.04	-116.15	
4.	**9b-carbene**	LUMO	-0.0592	-1.61	-37.15	-3.43
5.	**9c-carbene**	HOMO	-0.1822	-4.96	-114.33	
6.	**9c-carbene**	LUMO	-0.0581	-1.58	-36.46	-3.38
7.	^{Me}CAAC	HOMO		-5.33		
8.	^{Me}CAAC	LUMO		-0.51		-4.82
9.	IPr	HOMO	-0.2210	-6.01	-139.78	
10[a].	IPr	LUMO+1	-0.0177	-0.48	-12.21	-5.53
11.	IPr*	HOMO	-0.2249	-6.12	-141.12	
12.	IPr*	LUMO	-0.0330	-0.90	-20.71	-5.22

[a]LUMO+1 due to required orbital symmetry. [b]See, Falivene, L.; Cavallo, L. *Coord. Chem. Rev.* **2017**, *344*, 101-114.

5.3. Topographical Steric Maps from DFT Calculations

To eliminate effects potentially resulting from crystal packing, the %percentage buried volume (%V_{bur}) was calculated from the optimized structures of Au-complexes **9a-[Au]**–**9c-[Au]** at the B3LYP 6-311++g(d,p) level. The calculation showed the %V_{bur} of the NHC in **9a-[Au]** as 39.2% (SW, 36.0%; NW, 59.1%; NE, 32.4%; SE, 29.2%); in **9b-[Au]** as 38.3% (SW, 35.1%; NW, 47.9%; NE, 30.8%; SE, 39.5%); in **9c-[Au]** as 36.2% (SW, 44.3%; NW, 35.7%; NE, 34.4%; SE, 30.2%). The data clearly show that %V_{bur} decreases as the steric decreases from **9a-[Au]** → **9b-[Au]** → **9c-[Au]** (39.2%, 38.3% vs. 36.2%). The trends are well-matched with the %V_{bur} from crystal structures. Significant differentiation was noticed with a gradual decrease in the NW quadrant from **9a-[Au]** → **9b-[Au]** → **9c-[Au]**: 59.1% vs. 47.9% vs.

35.7%. A noteworthy change was also observed for **9a-[Au]** and **9c-[Au]** at SW quadrant 36.0% vs. 44.3%. Additionally, the NE quadrant was the least affected by steric variation from **9a-[Au]** → **9b-[Au]** → **9c-[Au]**: 32.4% vs 30.8% vs 34.4%. This unsymmetrical distribution of flexible steric hindrance in combination with the exceedingly strong s-donation are key factors in catalysis.

Figure S18. (A-C) Topographical steric maps of **9a-[Au]**, **9b-[Au]**, and **9c-[Au]** showing %V*bur* per quadrant at the B3LYP 6-311++g(d,p) level.

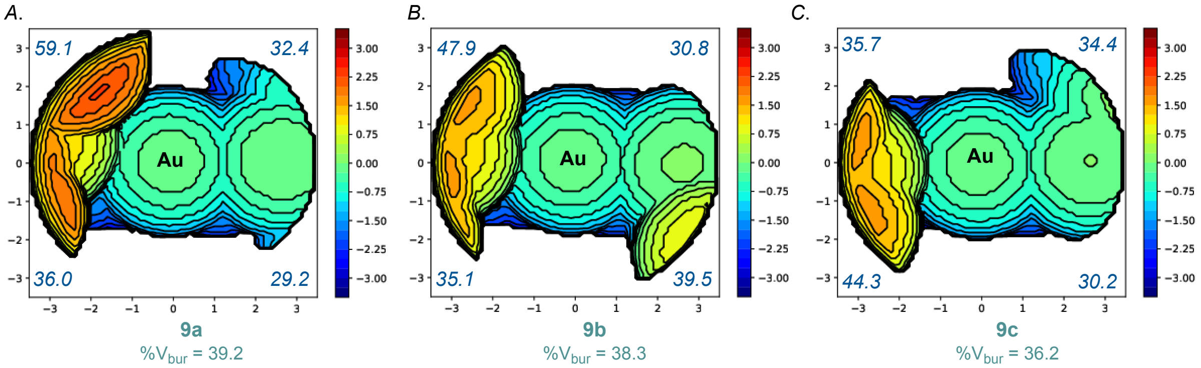

Table S7. %Vbur and Quadrant Distribution for Linear Au-Complexes **9a-[Au]**–**9c-[Au]** at the B3LYP 6-311++g(d,p) Level (Falivene, L. et al. Nat. Chem. 2019, 11, 872

Compound	%V_{bur} (%)	SW (%)	NW (%)	NE (%)	SE (%)
**9a-[Au]**	*39.2*	*36.0*	*59.1*	*32.4*	*29.2*
**9b-[Au]**	*38.2*	*35.1*	*47.9*	*30.8*	*39.5*
**9c-[Au]**	*36.2*	*44.3*	*35.7*	*34.4*	*30.2*

6. References

[1] J. Xia, S. Kou, H. Mu, Z. Jian, *Eur. Polym. J.* **2022**, *166*, 111022.

[2] K. Grudzien, B. Trzaskowski, M. Smolen, R. Gajda, K. Wozniak, K. Grela, *Dalton Trans.* **2017**, *46*, 11790–11799.

[3] Y. T. Hong, J. Park, H. Y. Kim, K. Oh, Adv. Synth. Catal. **2024**, *366*, 1212–1217.

[4] X. Li, Y. Sun, X. Huang, L. Zhang, L. Kong, B. Peng, *Org. Lett.* **2017**, *19*, 838–841.

[5] S. Yazdani, G. P. Junor, J. L. Peltier, M. Gembicky, M. Jazzar, D. B. Grotjahn, G. Bertrand, *ACS Catal*. **2020**, *10*, 5190−5201.

[6] a) O.V. Dolomanov, L.J. Bourhis, R. J. Gildea, J. A. K. Howard, H. Puschmann, *J. Appl. Cryst*. **2009**, *42*, 339−341; b) L. J. Bourhis, O. V. Dolomanov, R. J. Gildea, J. A. K. Howard, H. Puschmann, *Acta Cryst. A* **2015**, 71, 59−75; c) G. M. Sheldricka, *Acta Cryst. A* **2008**, *64*, 112−122.

[7] R. F. W. Bader, *Chem. Rev.* **1991**, *91*, 893−928.

[8] E. R. Johnson, S. Keinan, P. Mori-Sánchez, J. Contreras-García, A. J. Cohen, W. Yang, *J. Am. Chem. Soc.* **2010**, *132*, 6498−6506.

[9] T. Lu, F. Chen, *J. Comput. Chem.* **2012**, *33*, 580−592.

[10] W. Humphrey, A. Dalke, K. Schulten, *J. Molec. Graphics* **1996**, *14*, 33−38.

7. ^{1}H and ^{13}C NMR Spectra

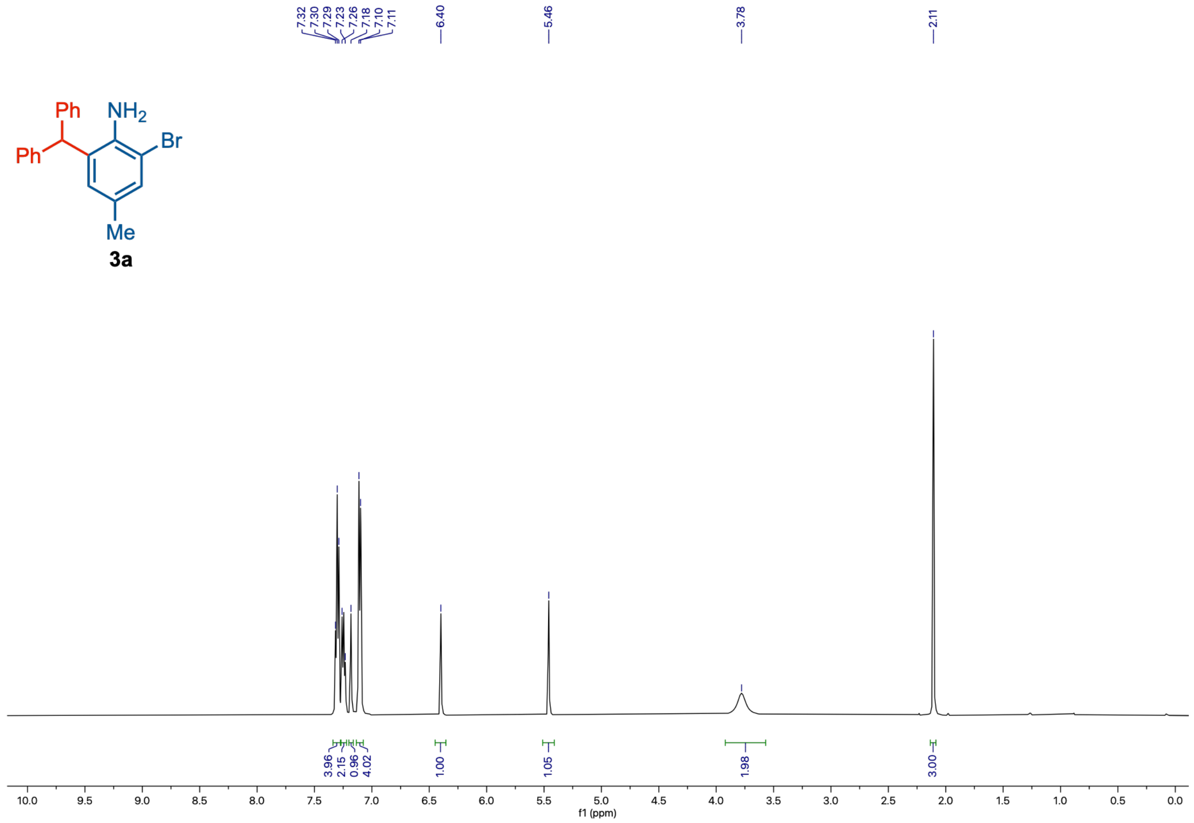

^{1}H NMR (500 MHz, $CDCl_3$) spectrum of **3a**

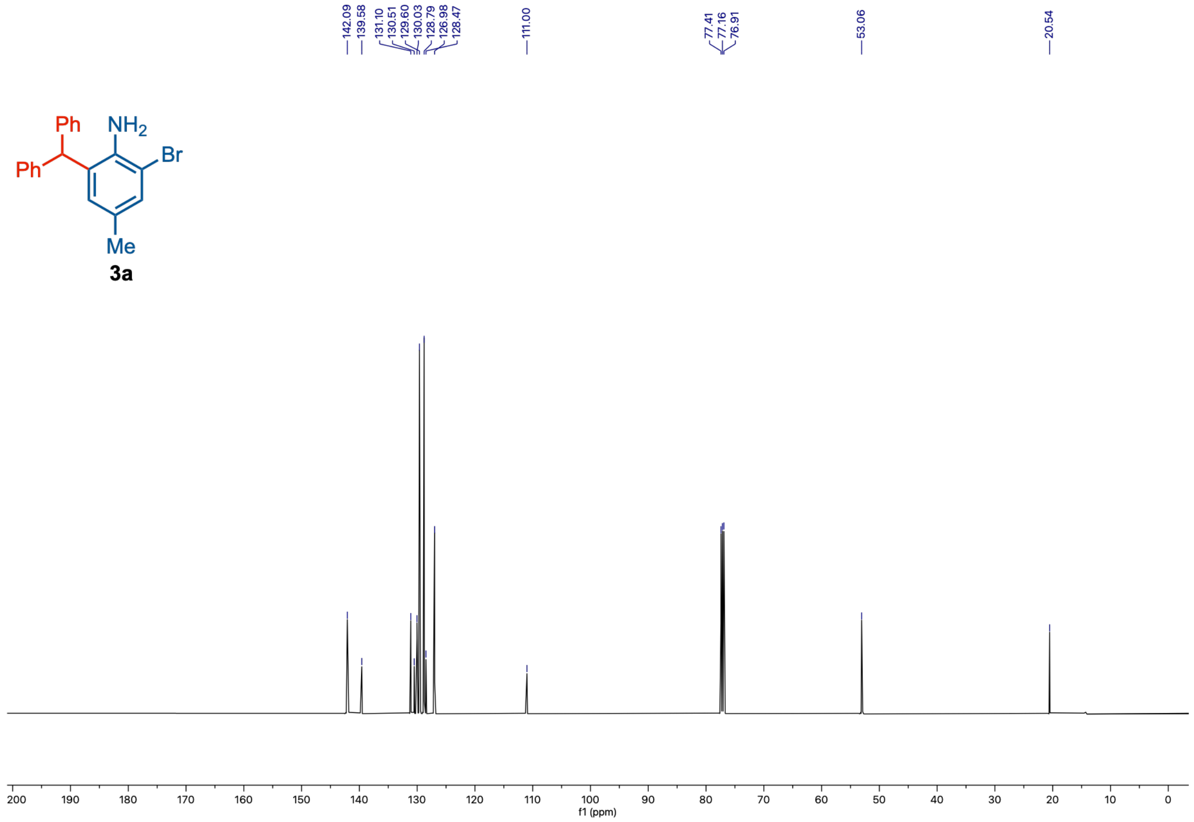

^{13}C NMR (126 MHz, $CDCl_3$) spectrum of **3a**

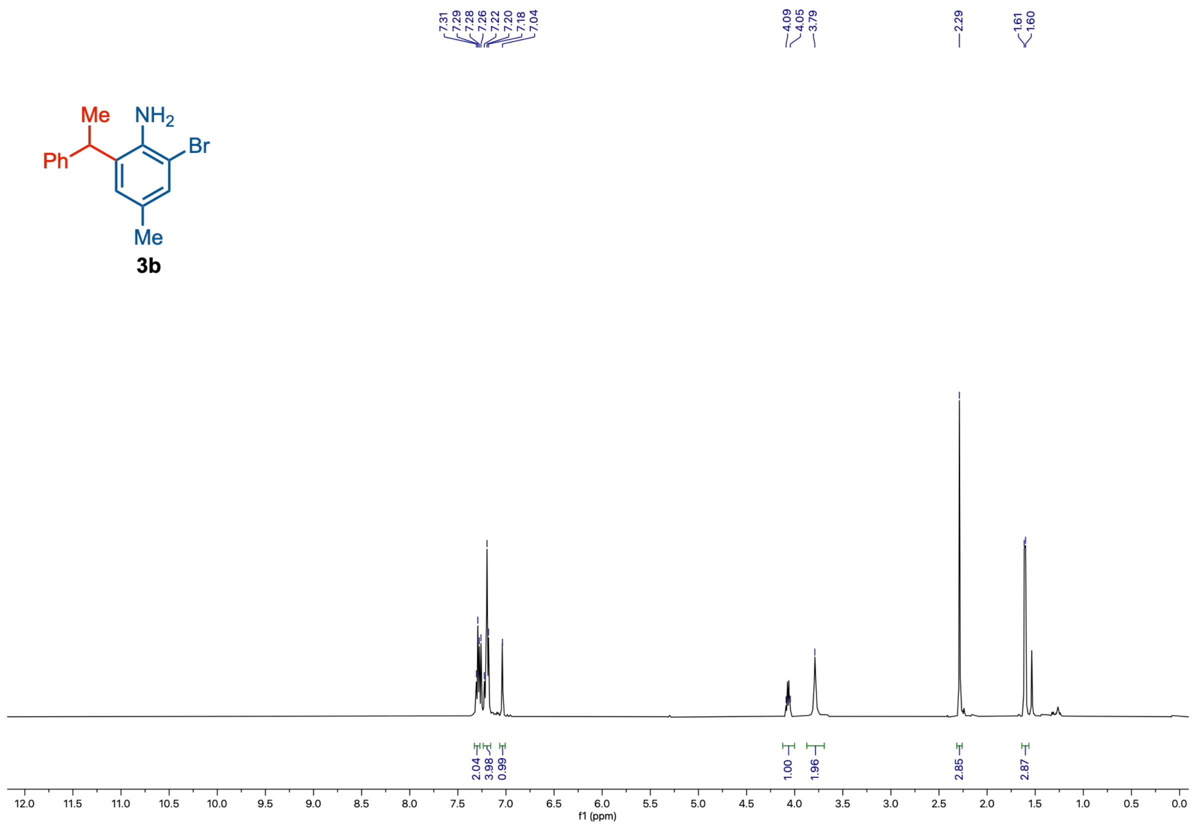

^{1}H NMR (500 MHz, $CDCl_3$) spectrum of **3b**

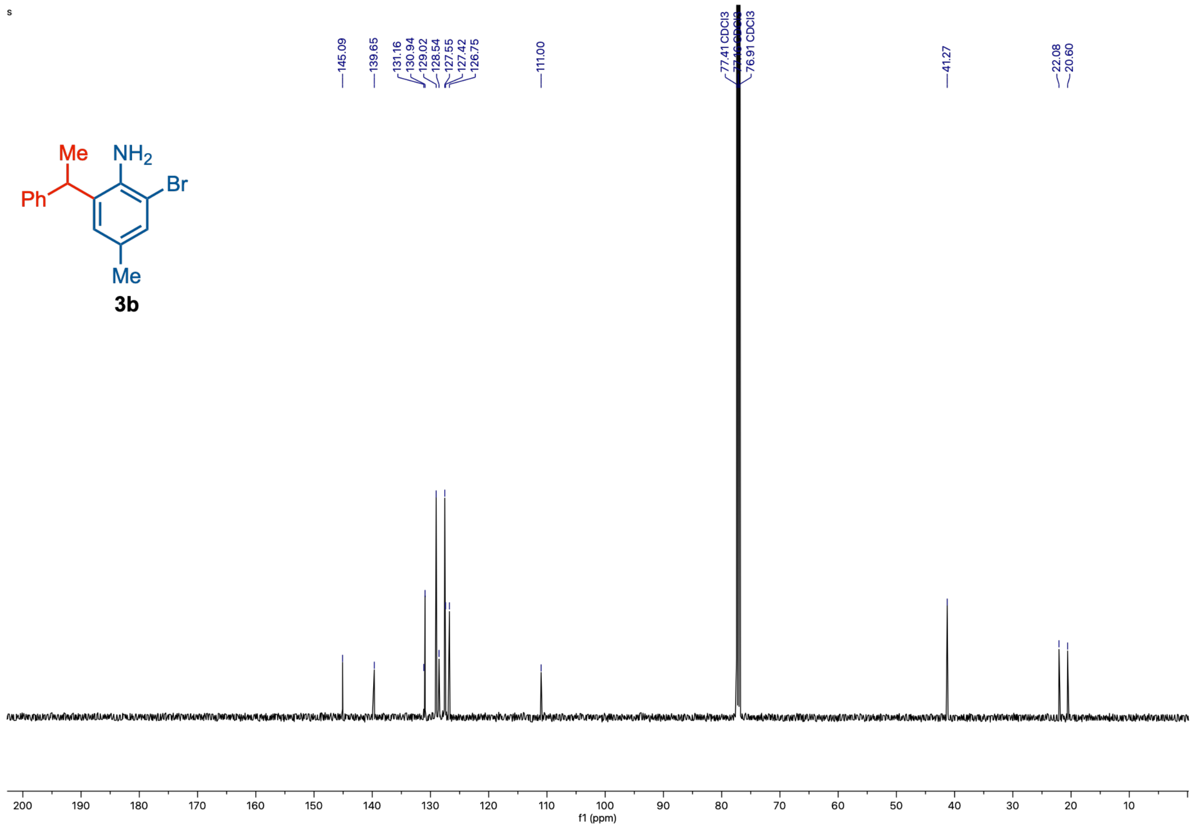

^{13}C NMR (126 MHz, $CDCl_3$) spectrum of **3b**

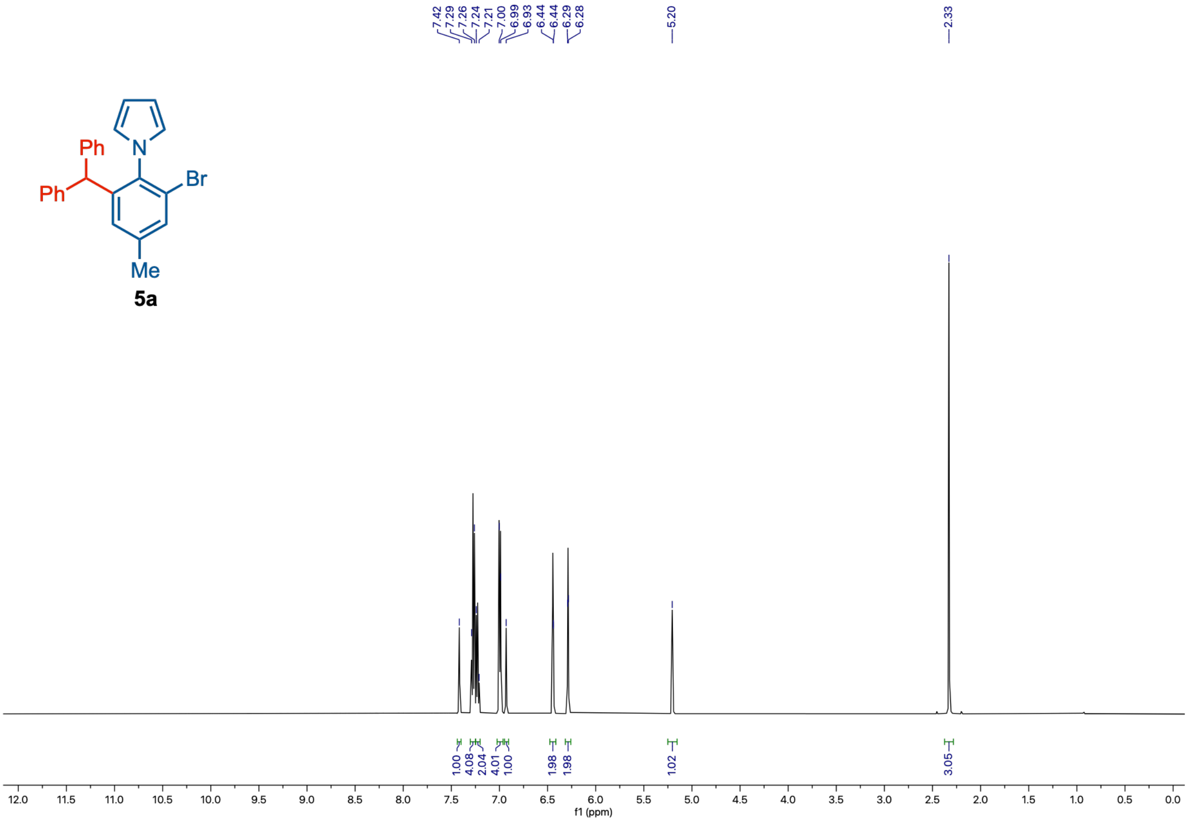

^{1}H NMR (500 MHz, $CDCl_3$) spectrum of **5a**

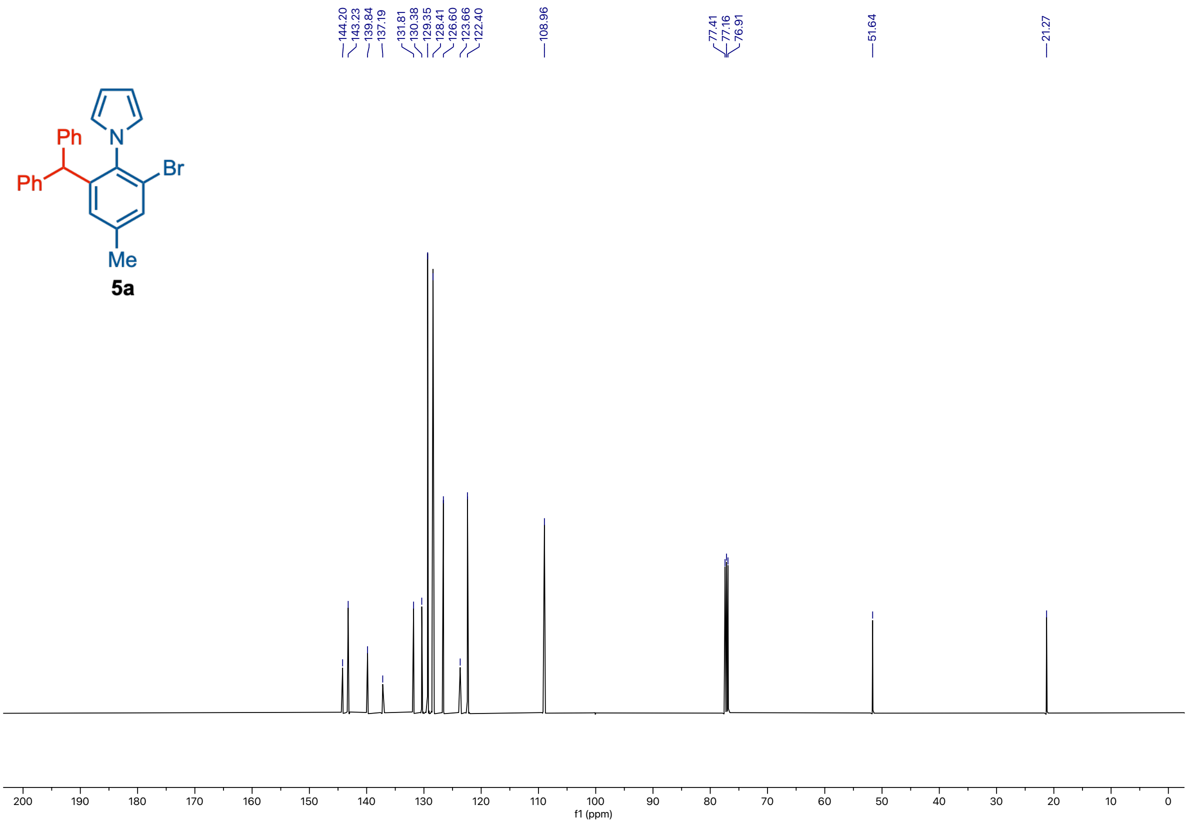

^{13}C NMR (126 MHz, $CDCl_3$) spectrum of **5a**

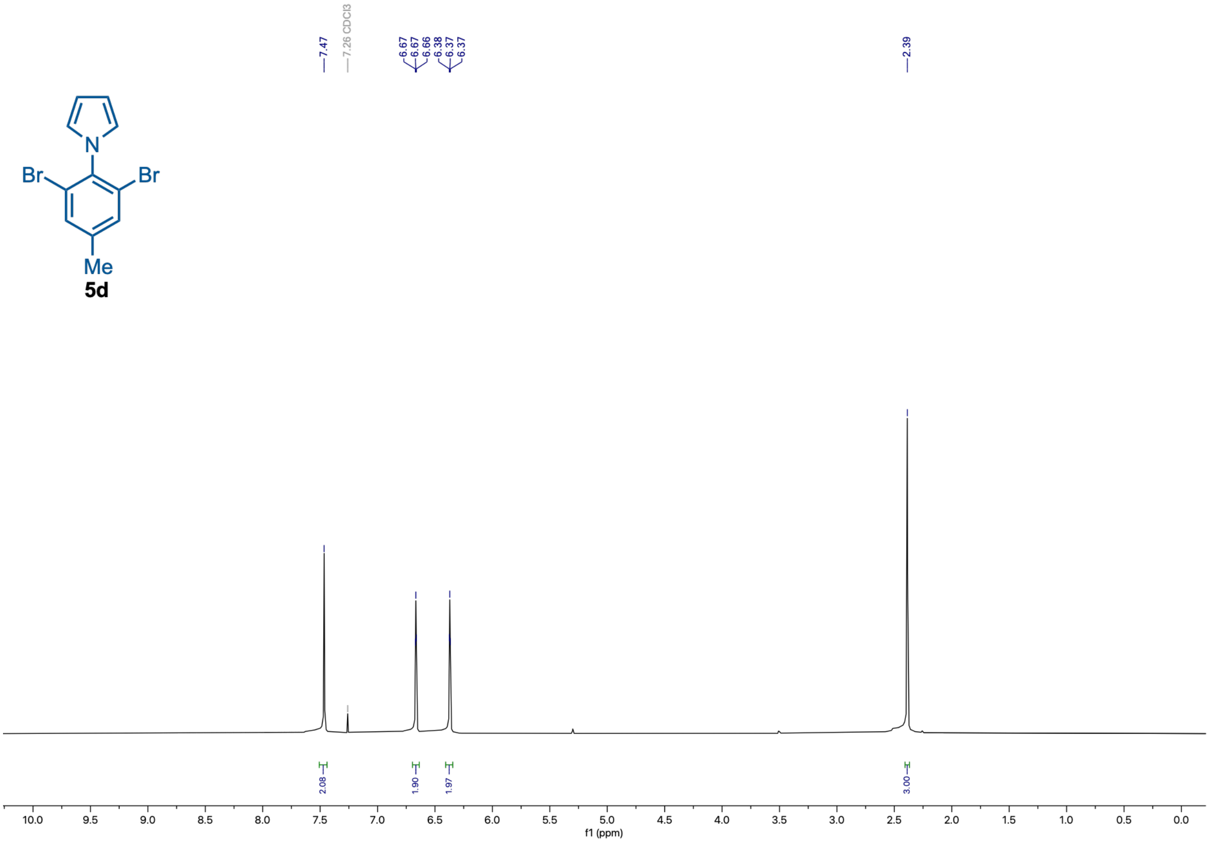

^{1}H NMR (500 MHz, $CDCl_3$) spectrum of **5d**

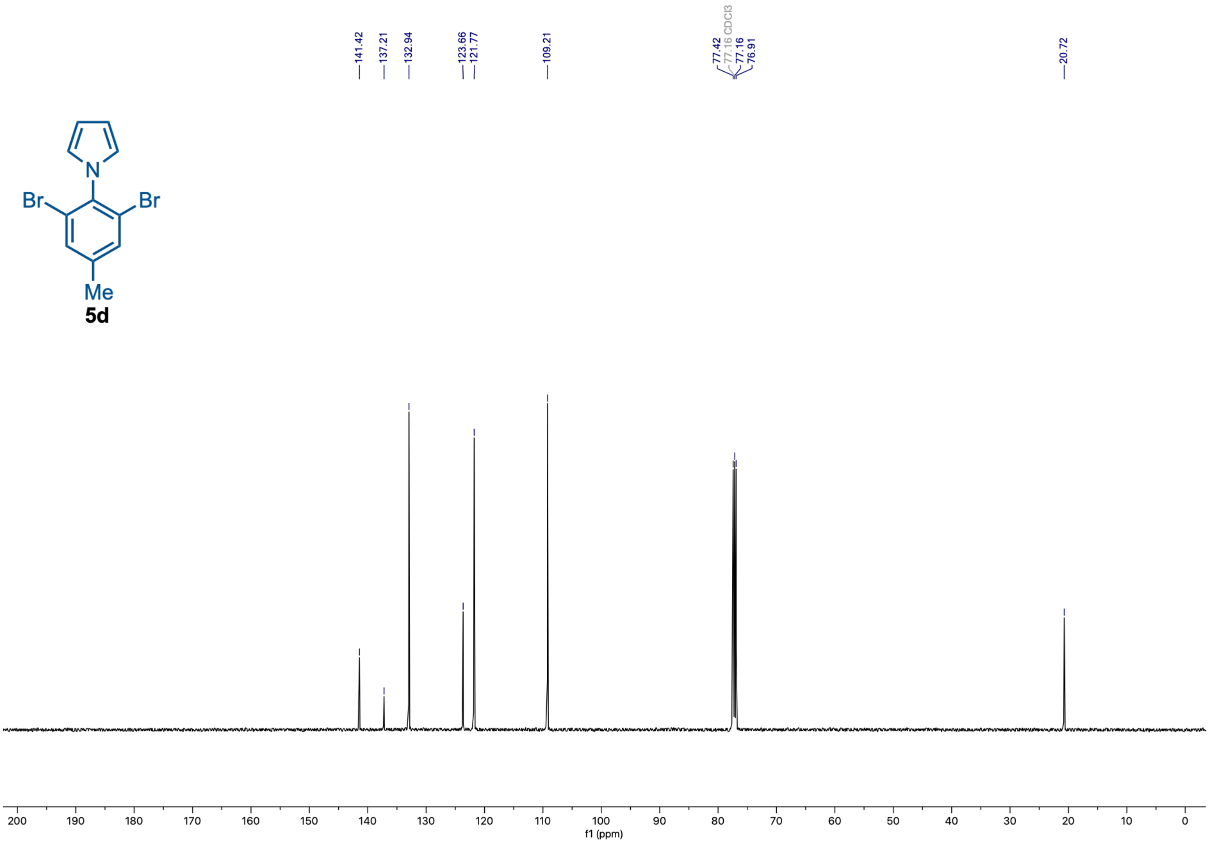

^{13}C NMR (126 MHz, $CDCl_3$) spectrum of **5d**

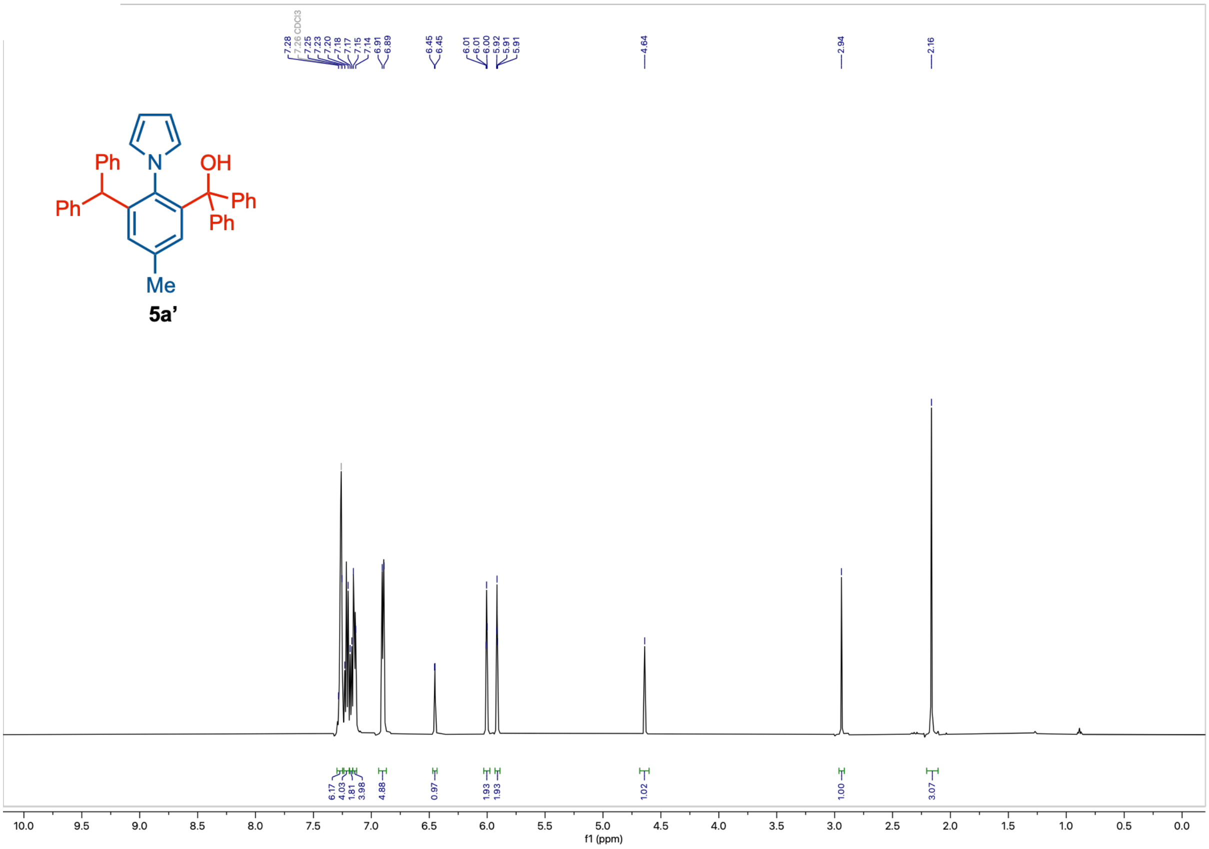

^{1}H NMR (500 MHz, $CDCl_3$) spectrum of **5a'**

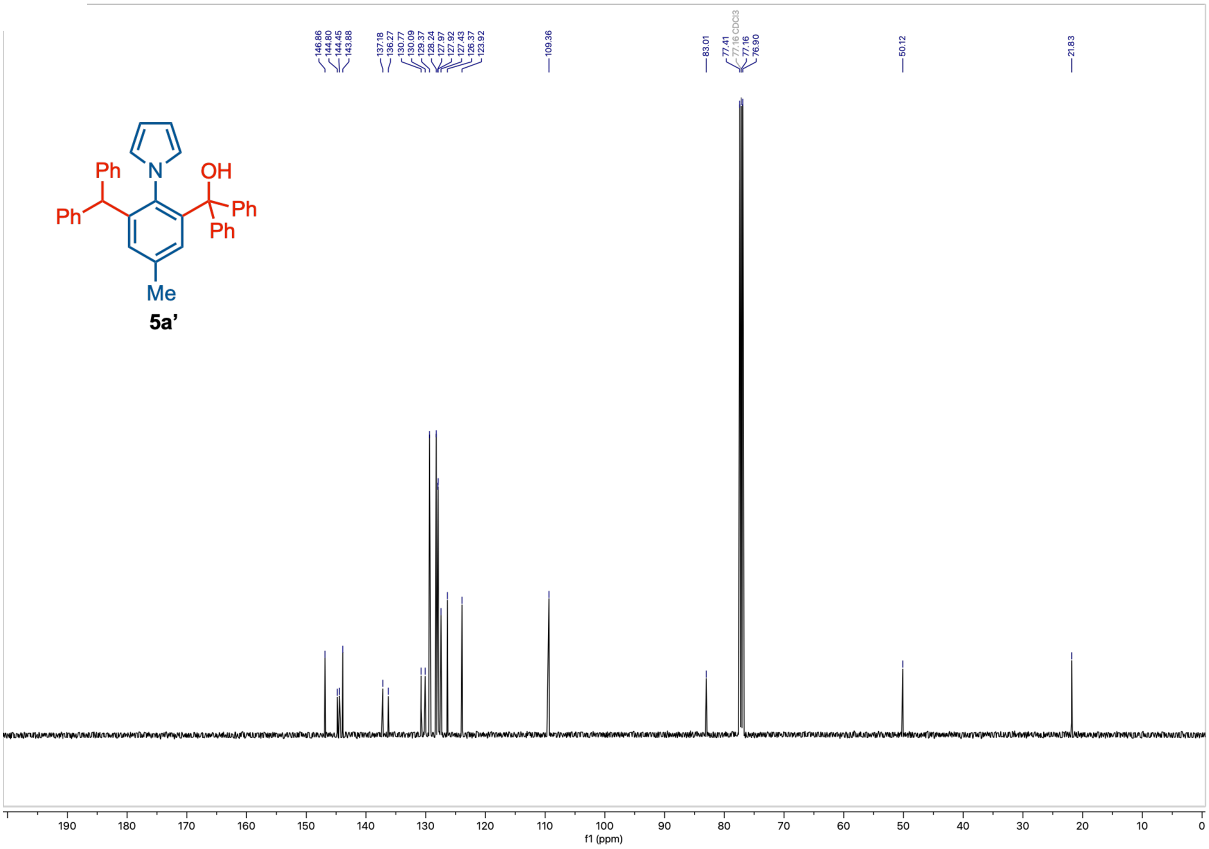

^{13}C NMR (126 MHz, $CDCl_3$) spectrum of **5a'**

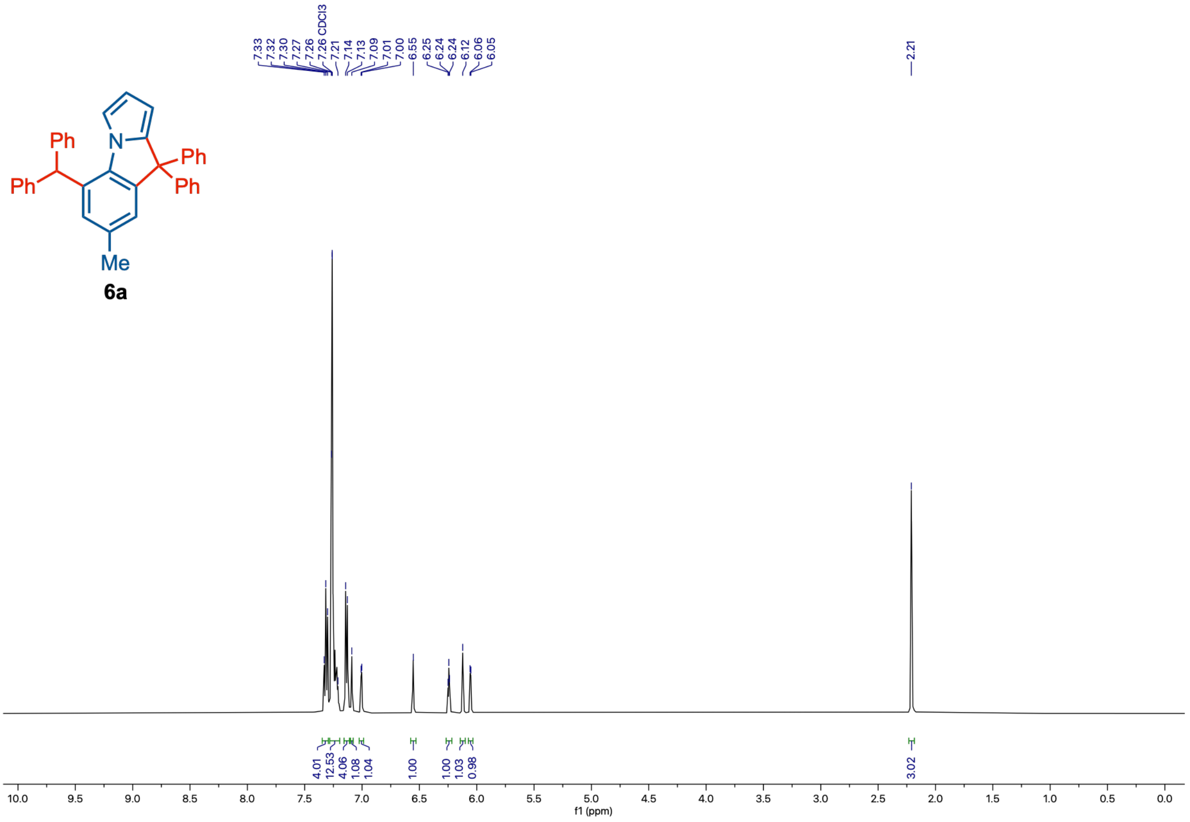

^{1}H NMR (126 MHz, $CDCl_3$) spectrum of **6a**

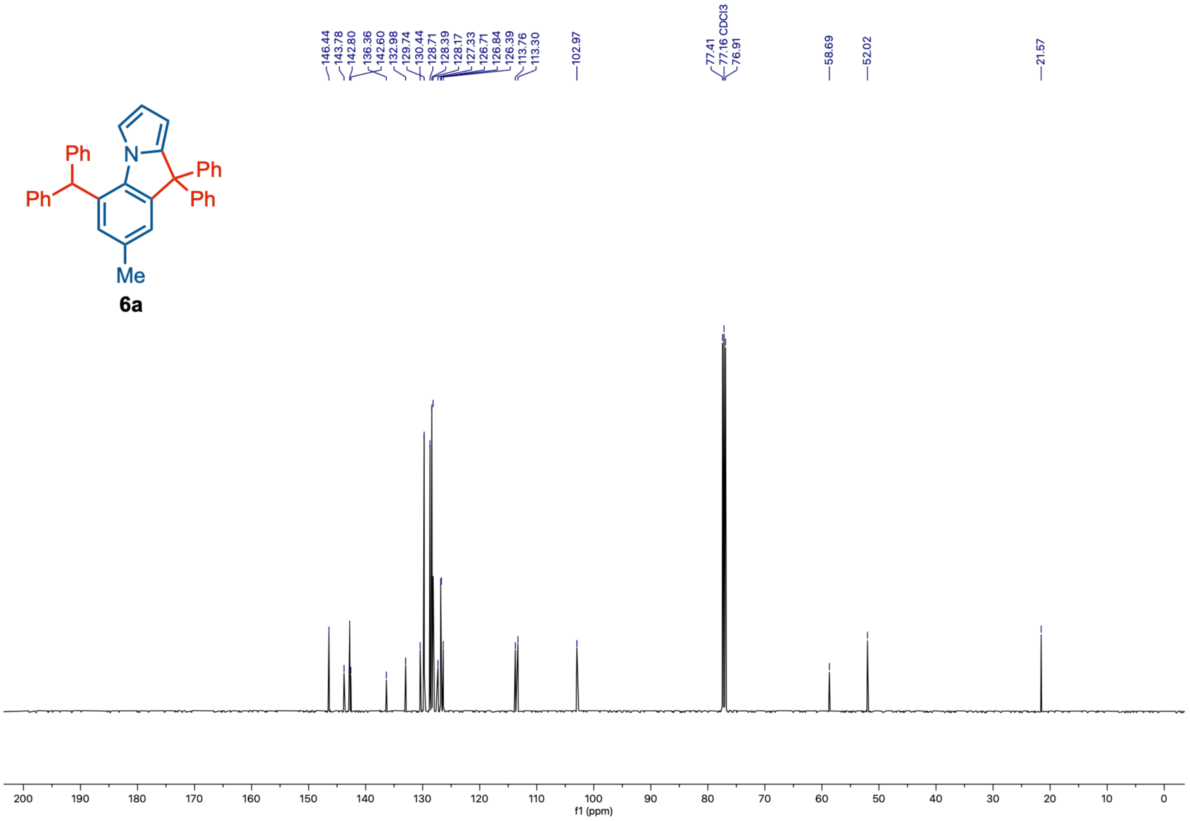

^{13}C NMR (126 MHz, $CDCl_3$) spectrum of **6a**

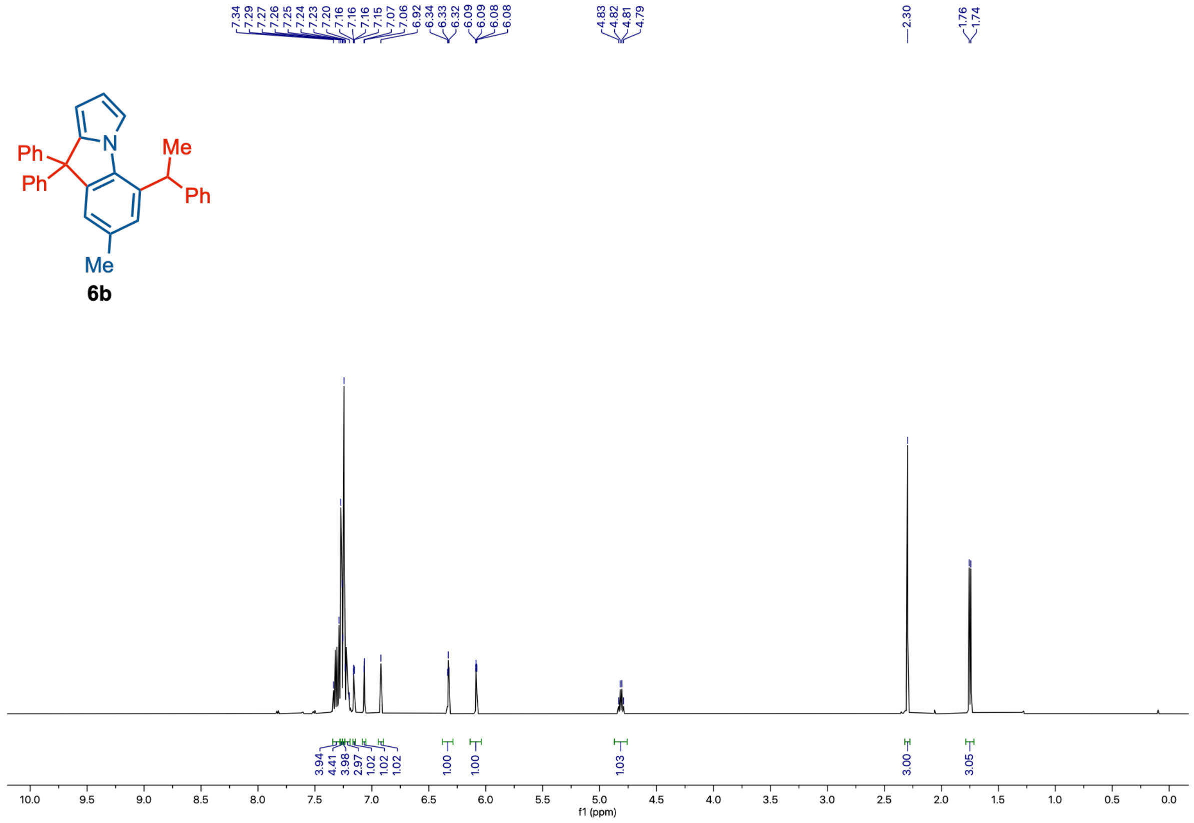

^{1}H NMR (126 MHz, $CDCl_3$) spectrum of **6b**

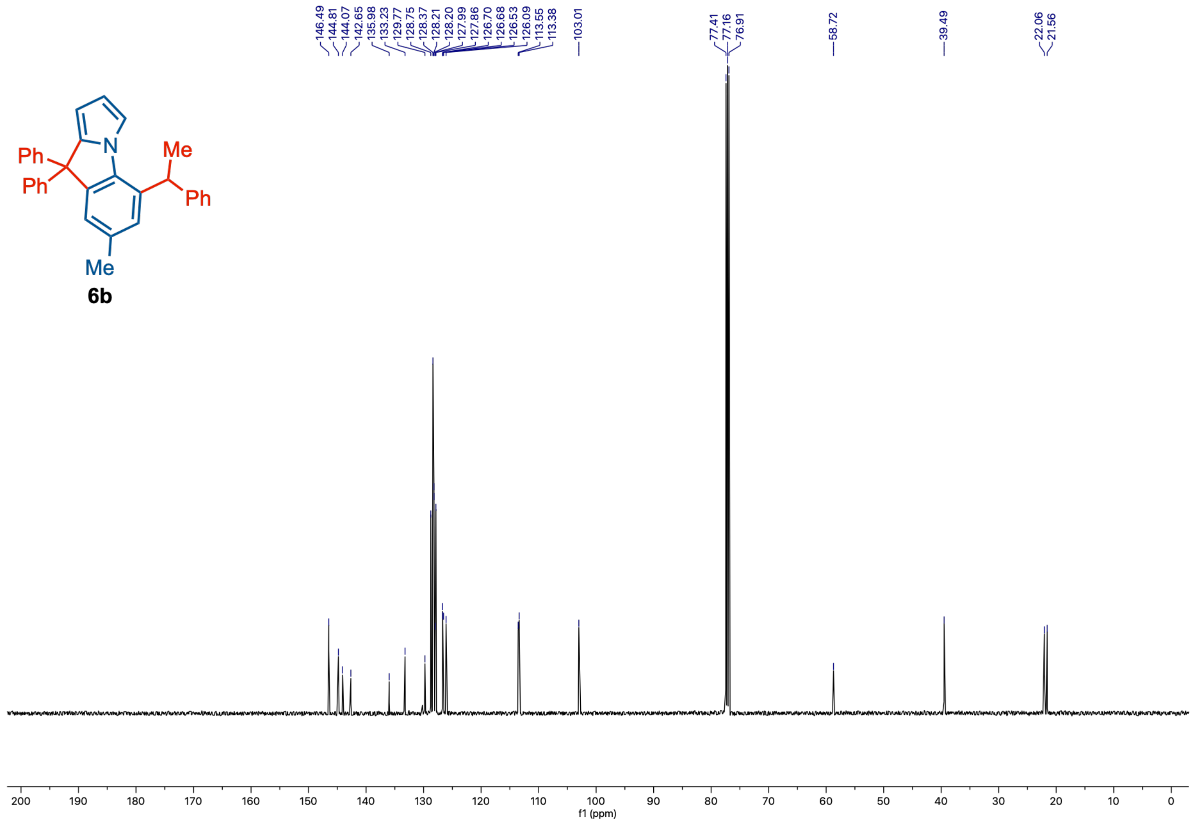

^{13}C NMR (126 MHz, $CDCl_3$) spectrum of **6b**

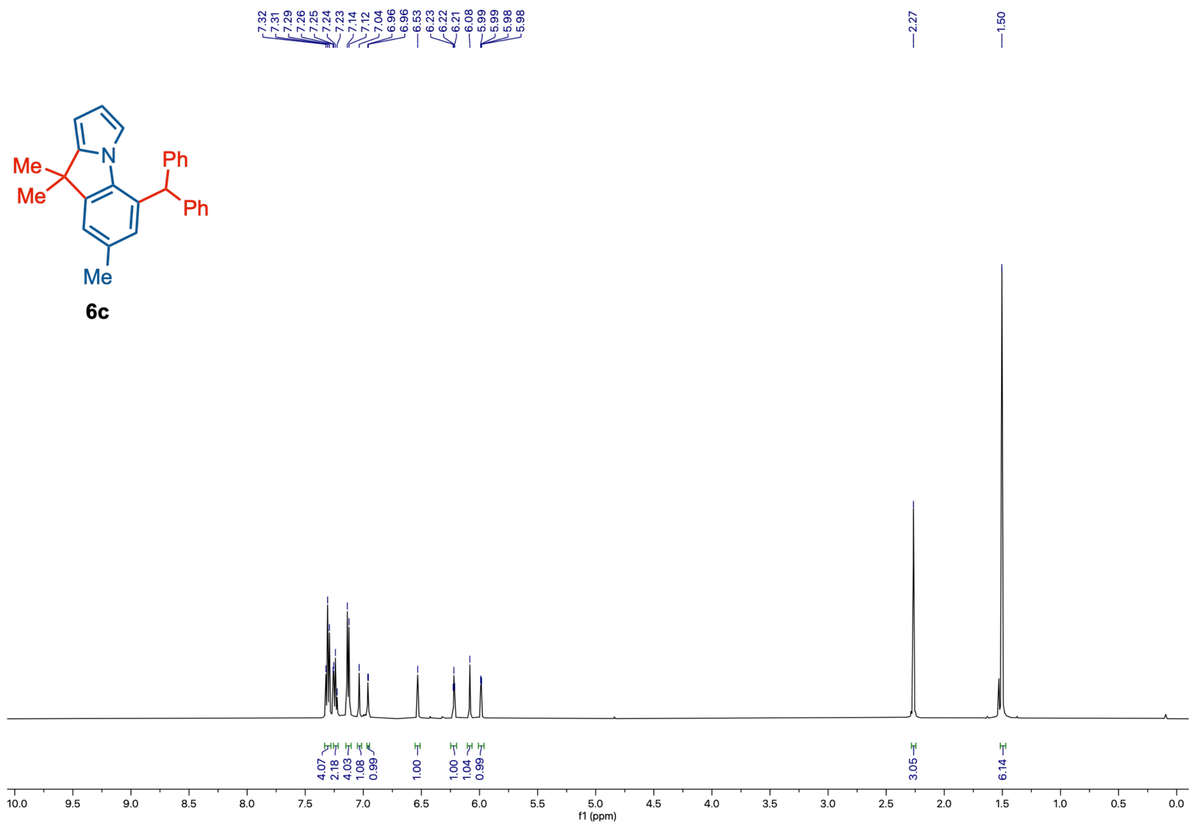

^{1}H NMR (126 MHz, $CDCl_3$) spectrum of **6c**

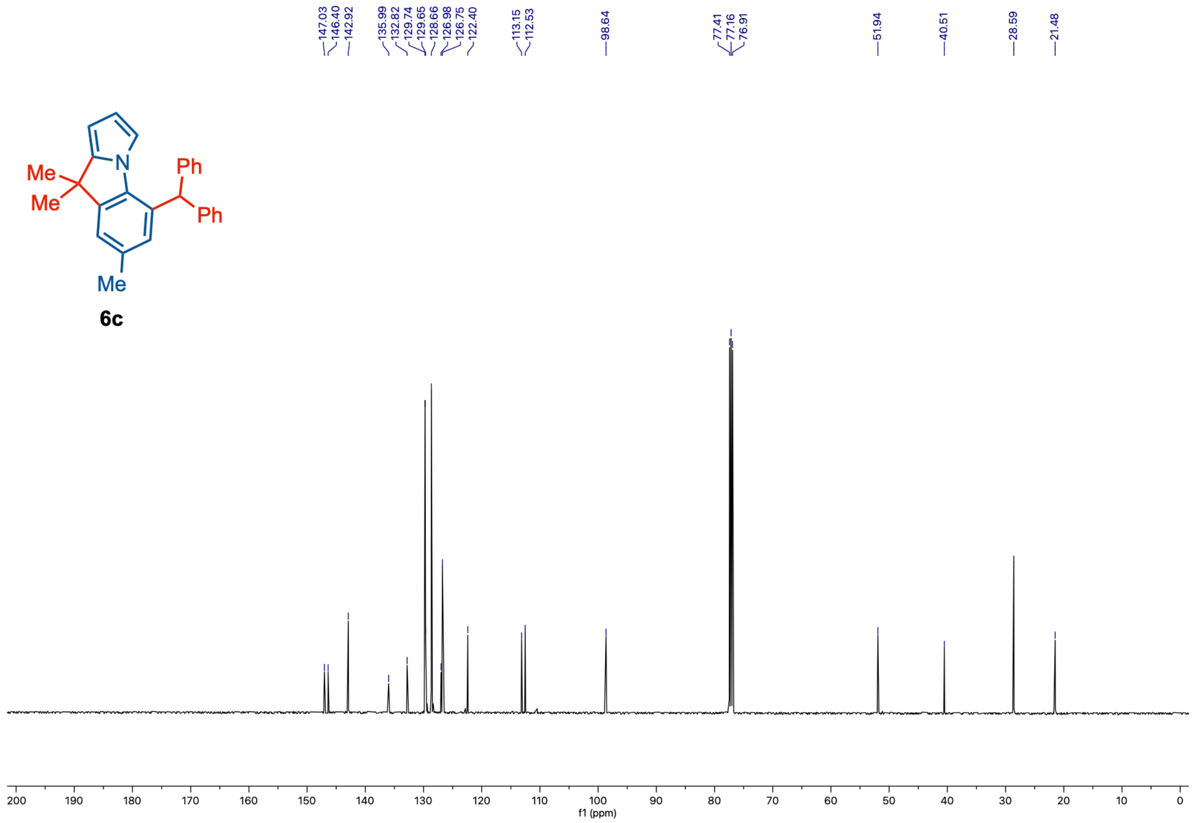

^{13}C NMR (126 MHz, $CDCl_3$) spectrum of **6c**

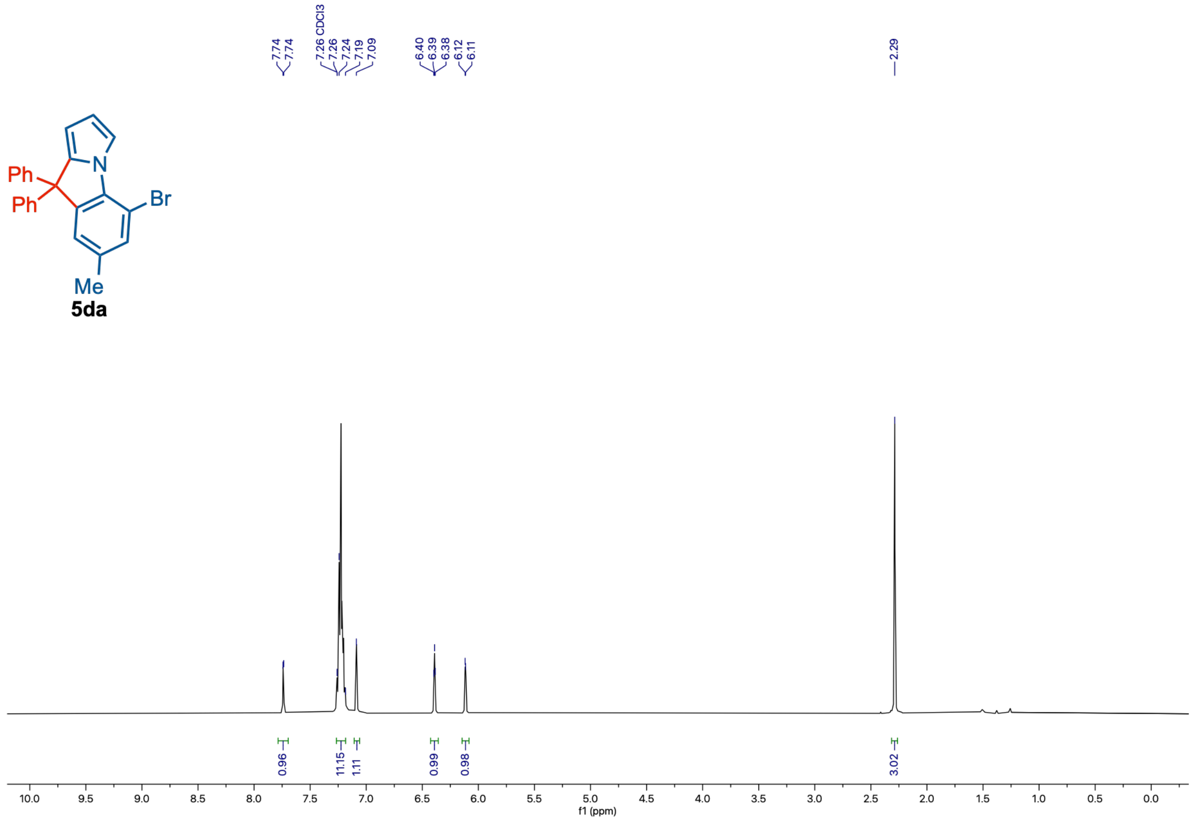

^{1}H NMR (500 MHz, $CDCl_3$) spectrum of **5da**

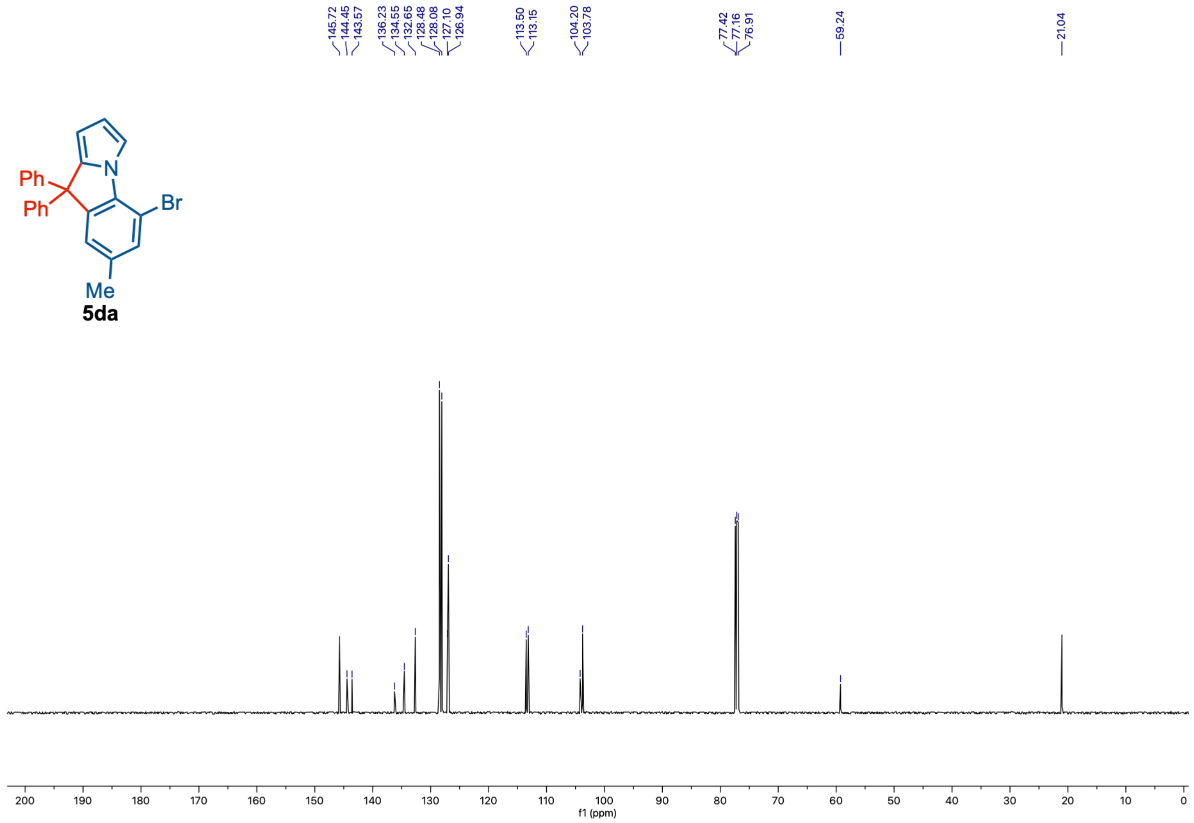

^{13}C NMR (126 MHz, $CDCl_3$) spectrum of **5da**

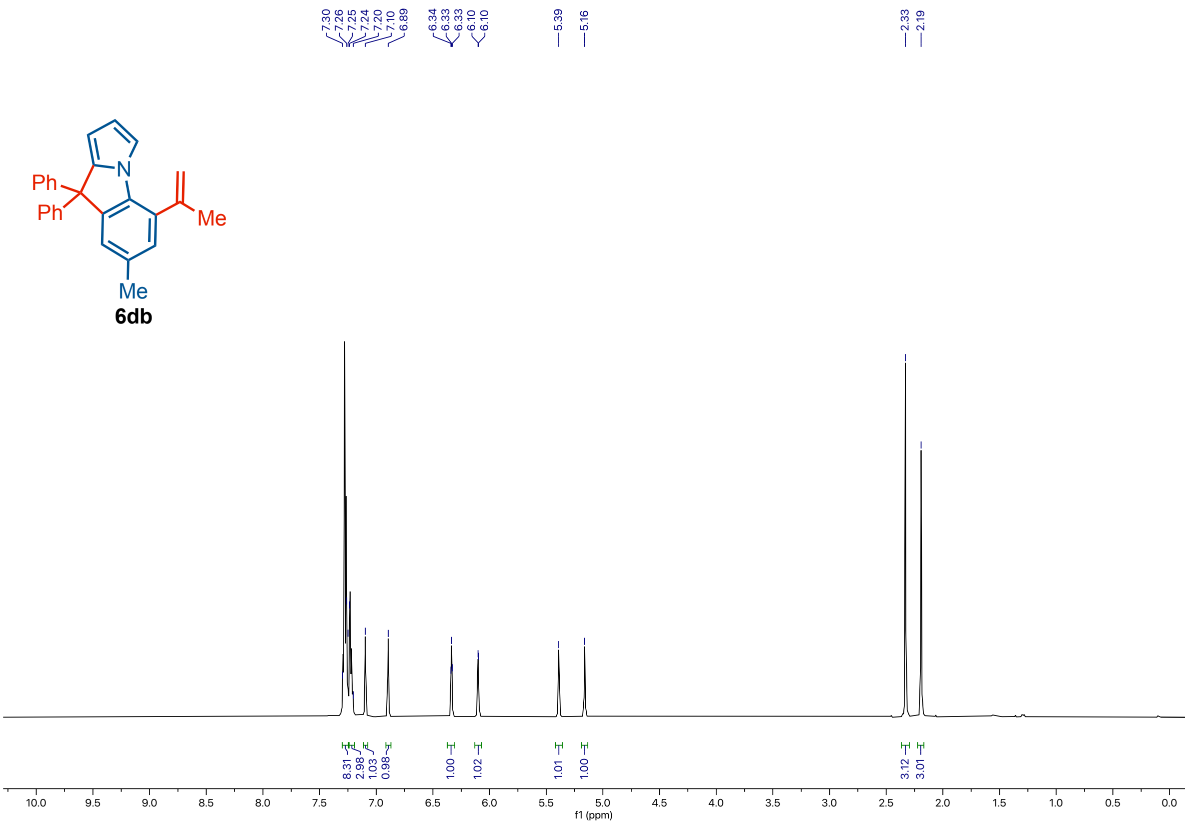

^{1}H NMR (500 MHz, $CDCl_3$) spectrum of **5db**

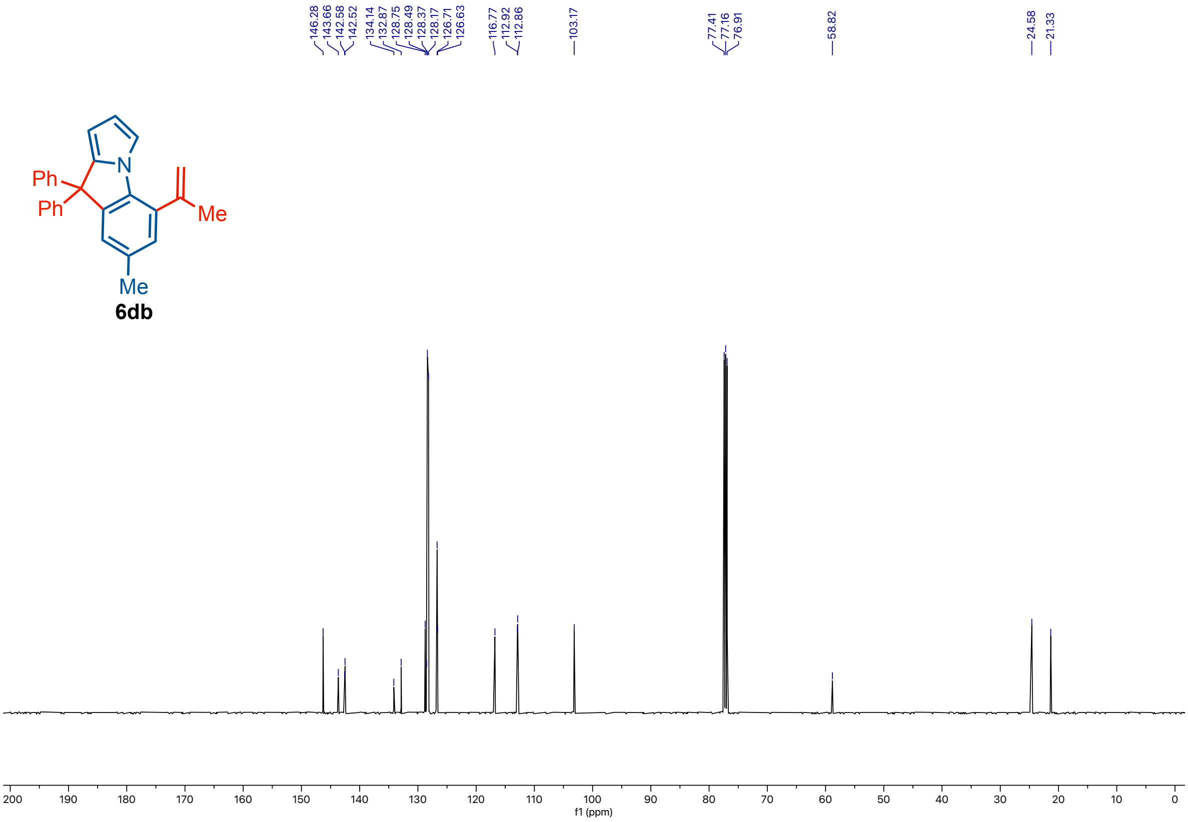

^{13}C NMR (126 MHz, $CDCl_3$) spectrum of **5db**

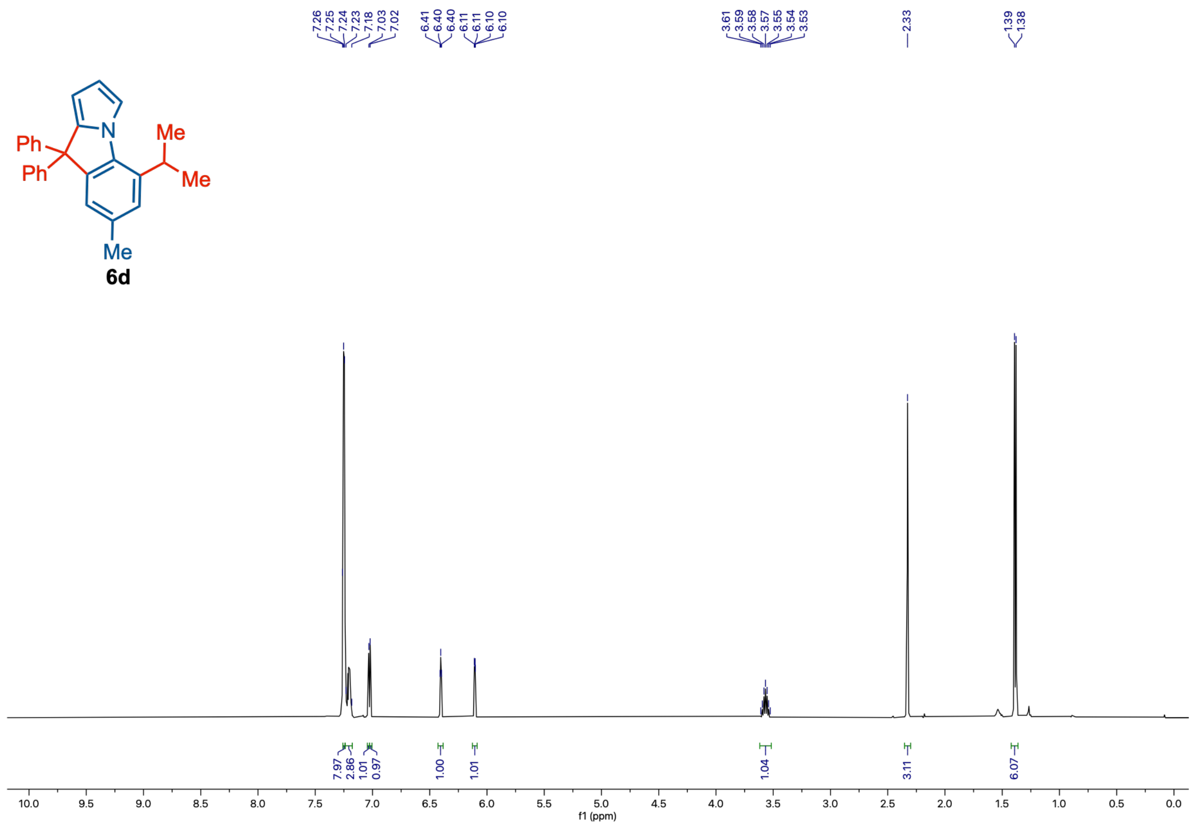

^{1}H NMR (500 MHz, $CDCl_3$) spectrum of **6d**

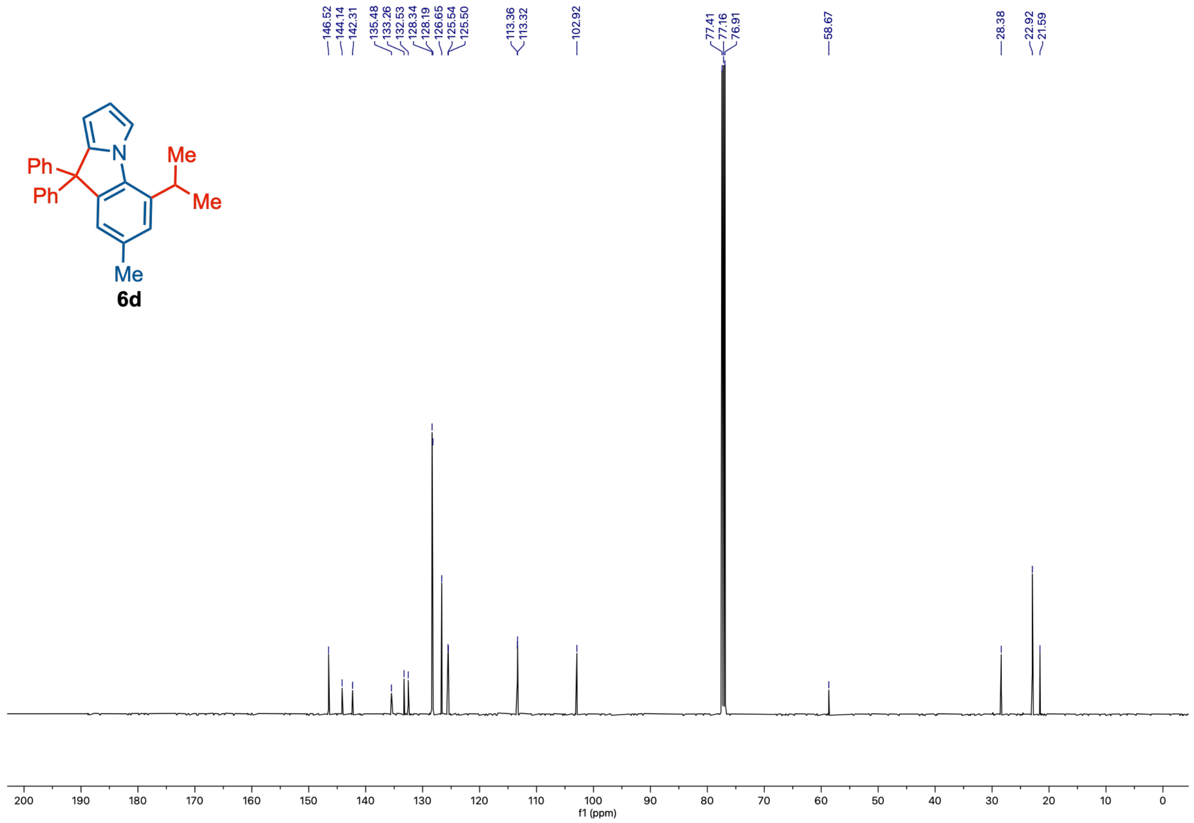

^{13}C NMR (126 MHz, $CDCl_3$) spectrum of **6d**

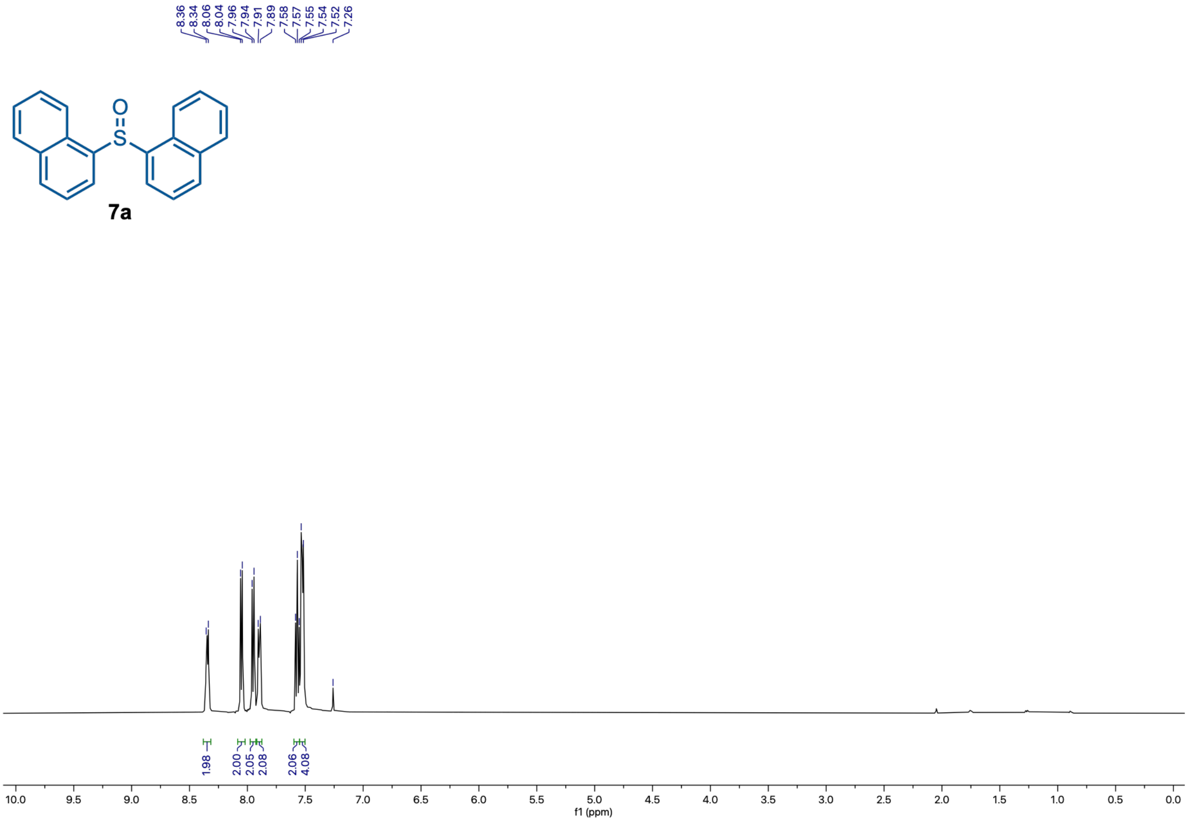

^1H NMR (500 MHz, $CDCl_3$) spectrum of **7a**

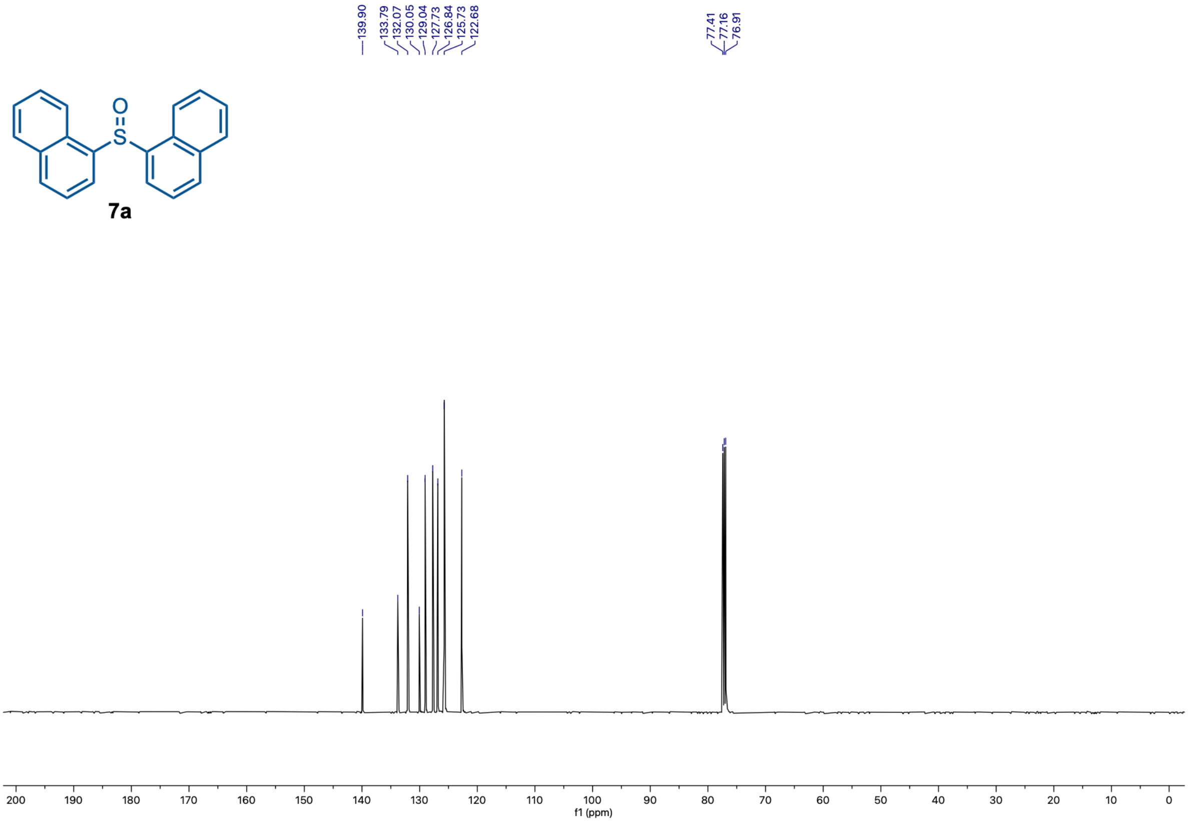

^{13}C NMR (126 MHz, $CDCl_3$) spectrum of **7a**

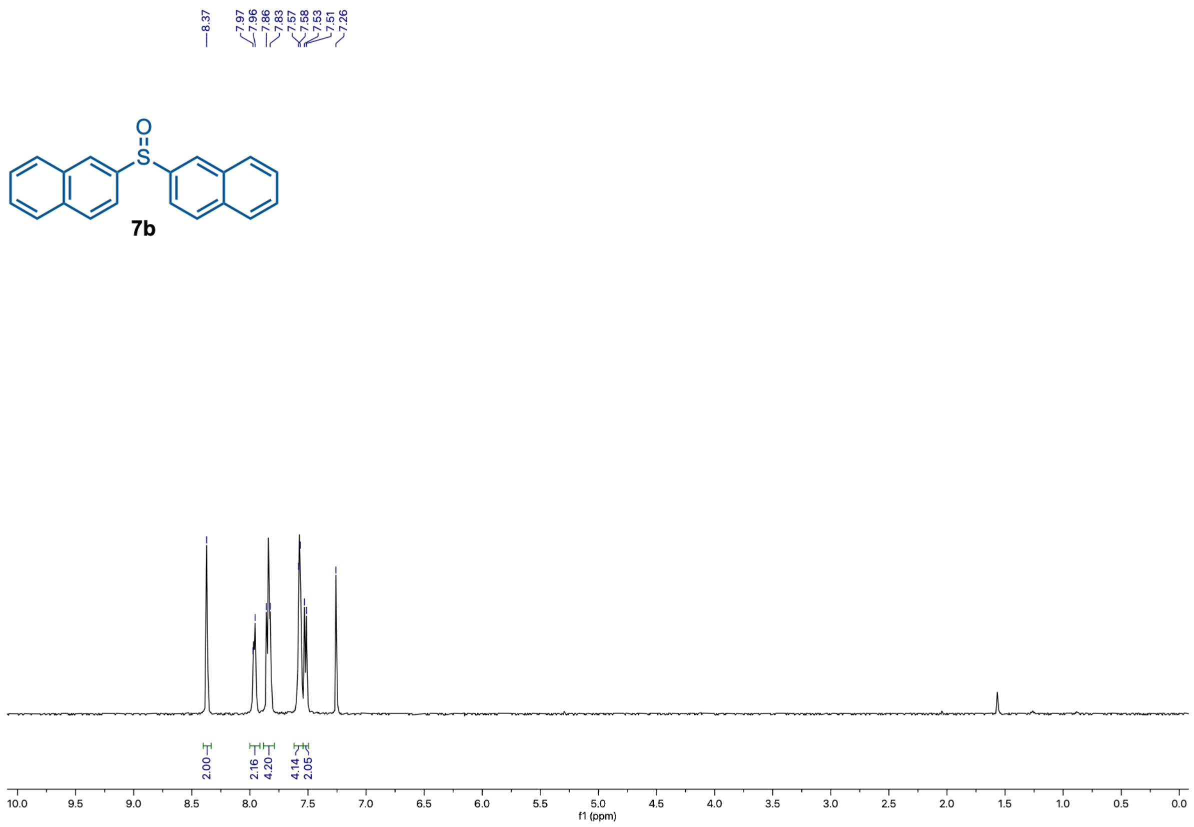

^{1}H NMR (500 MHz, $CDCl_3$) spectrum of **7b**

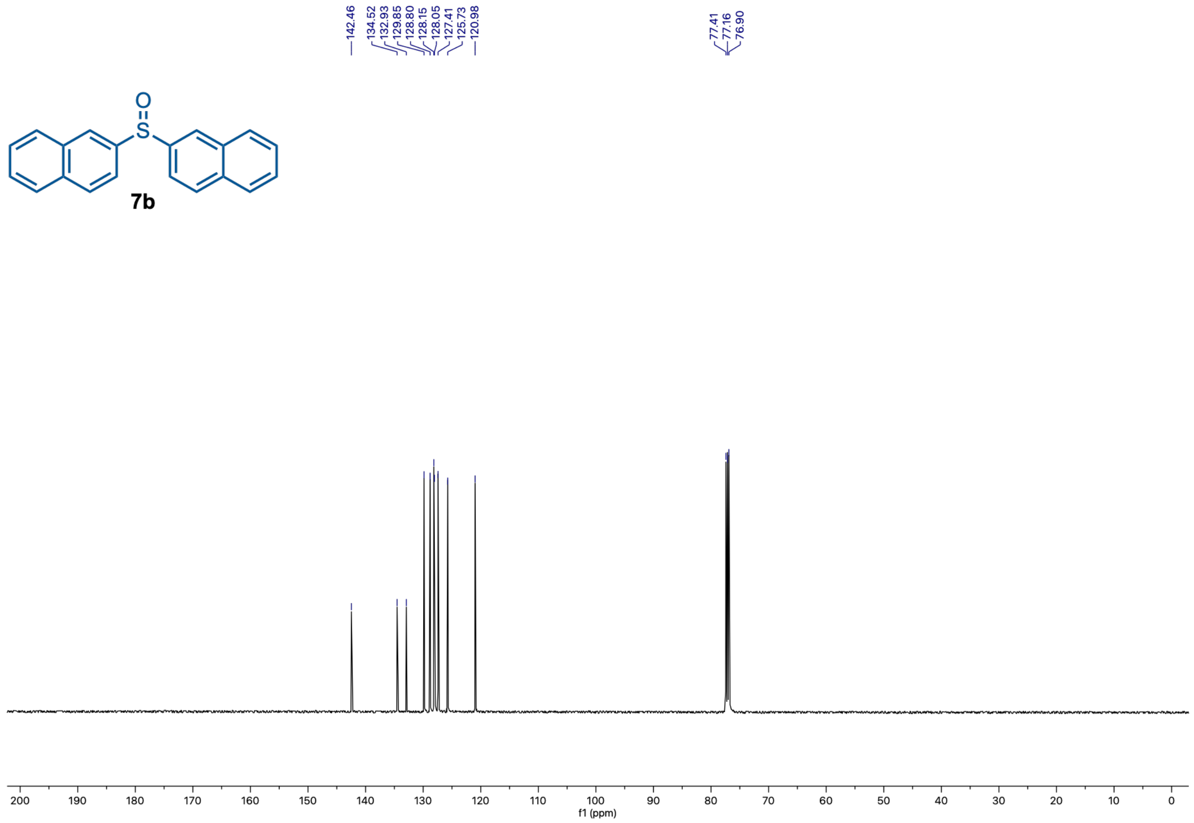

^{13}C NMR (126 MHz, $CDCl_3$) spectrum of **7b**

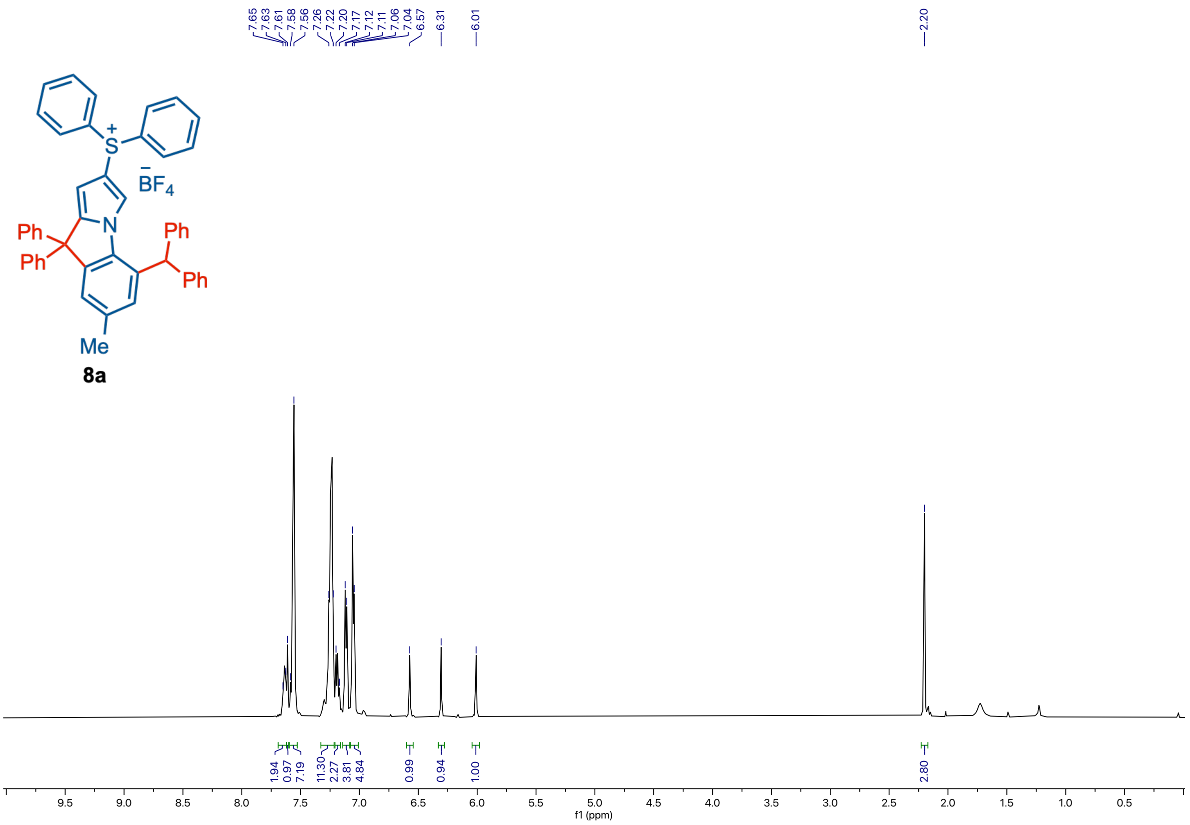

^{1}H NMR (500 MHz, $CDCl_3$) spectrum of **8a**

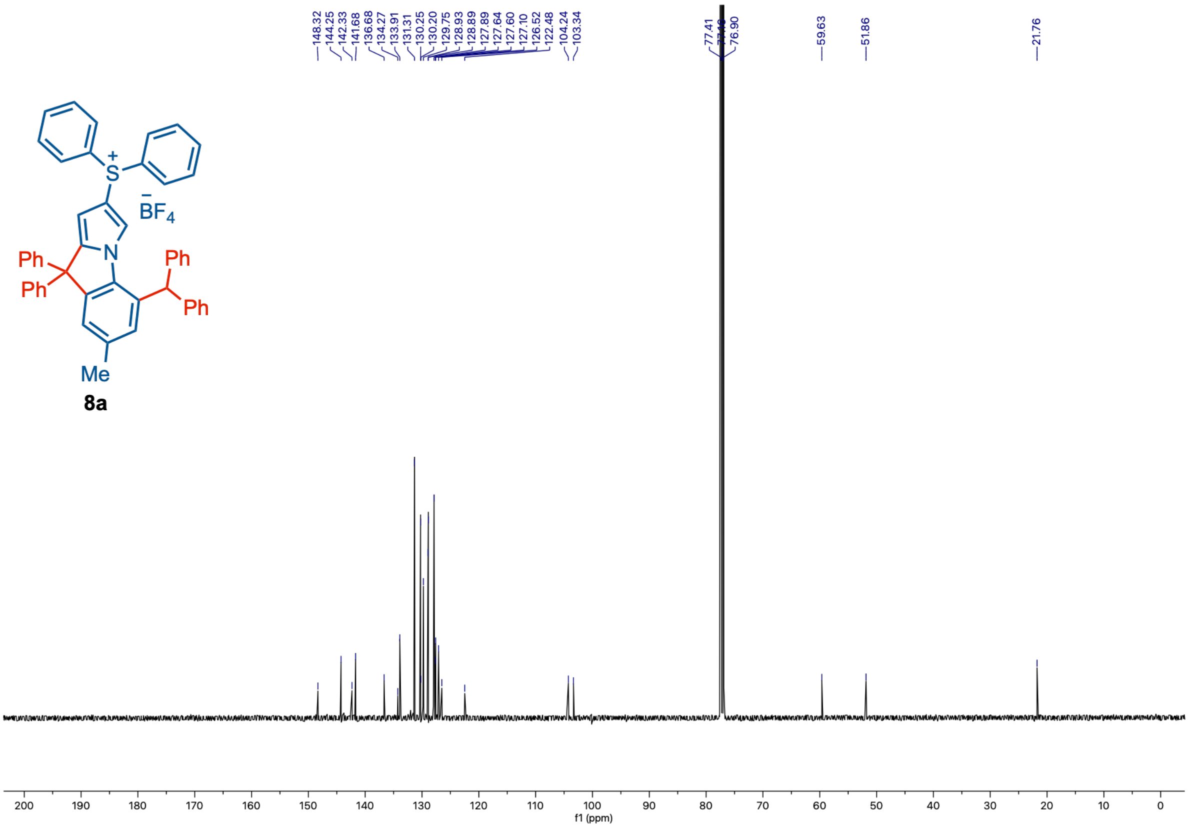

^{13}C NMR (126 MHz, $CDCl_3$) spectrum of **8a**

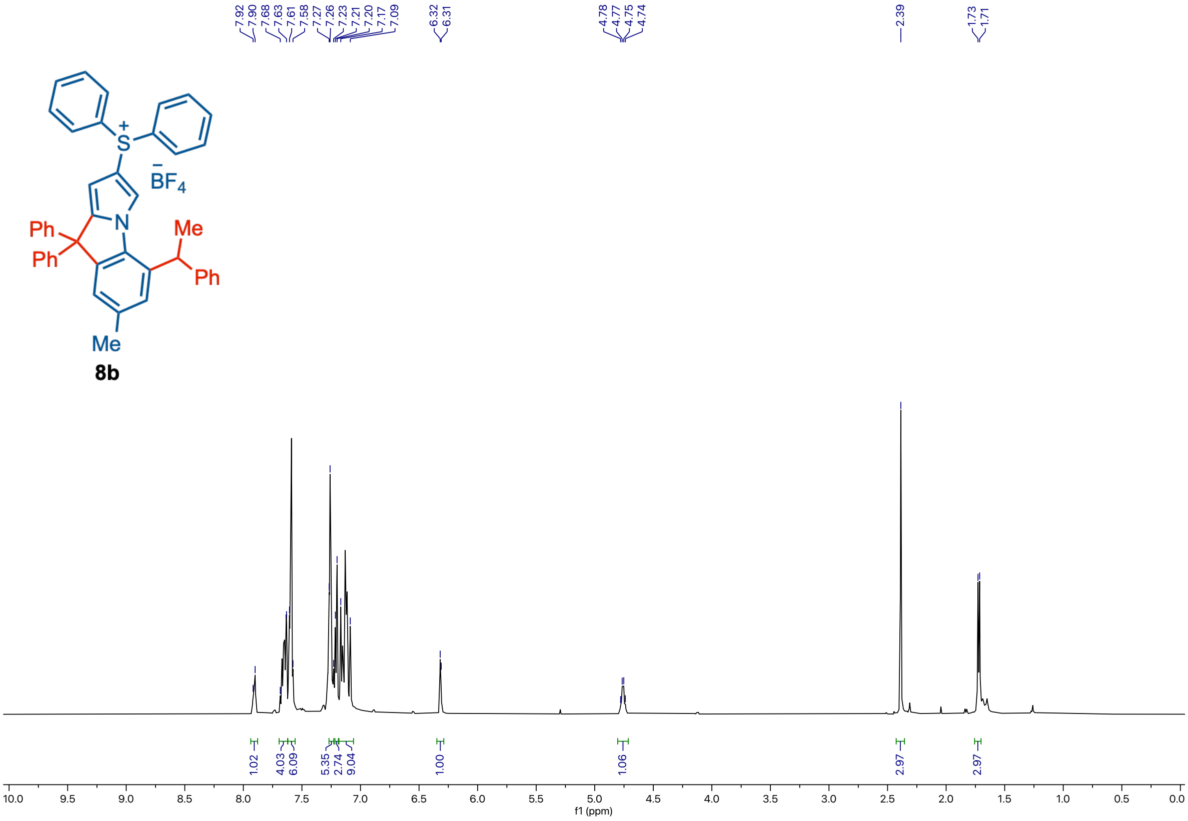

^{1}H NMR (500 MHz, $CDCl_3$) spectrum of **8b**

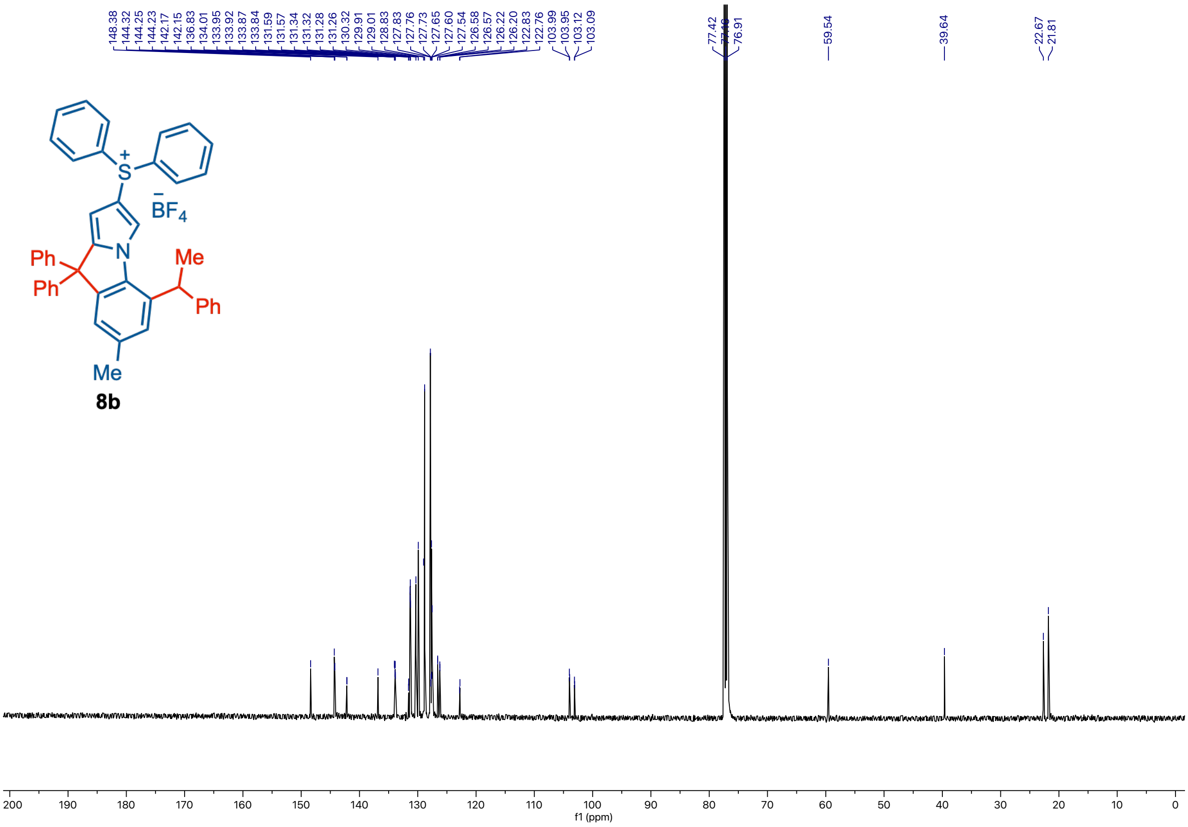

^{13}C NMR (126 MHz, $CDCl_3$) spectrum of **8b**

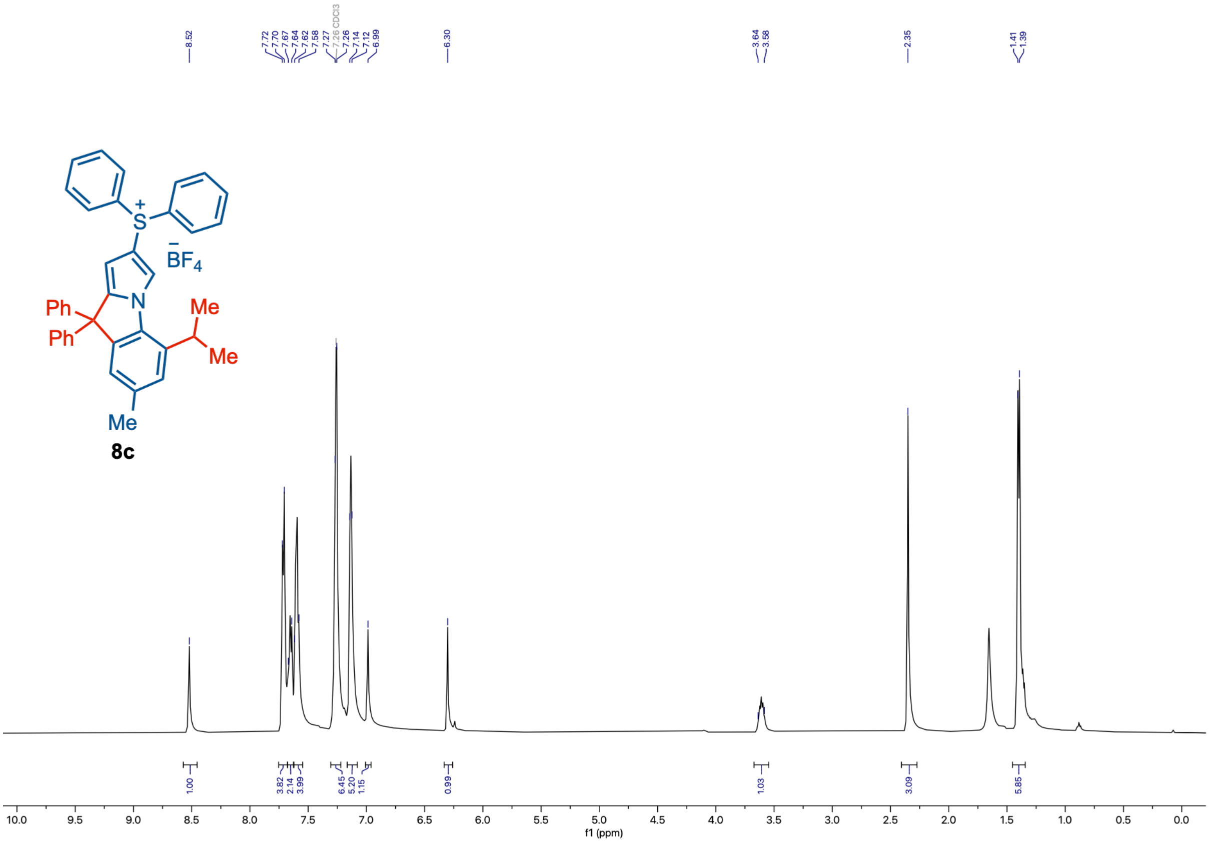

^{1}H NMR (500 MHz, $CDCl_3$) spectrum of **8c**

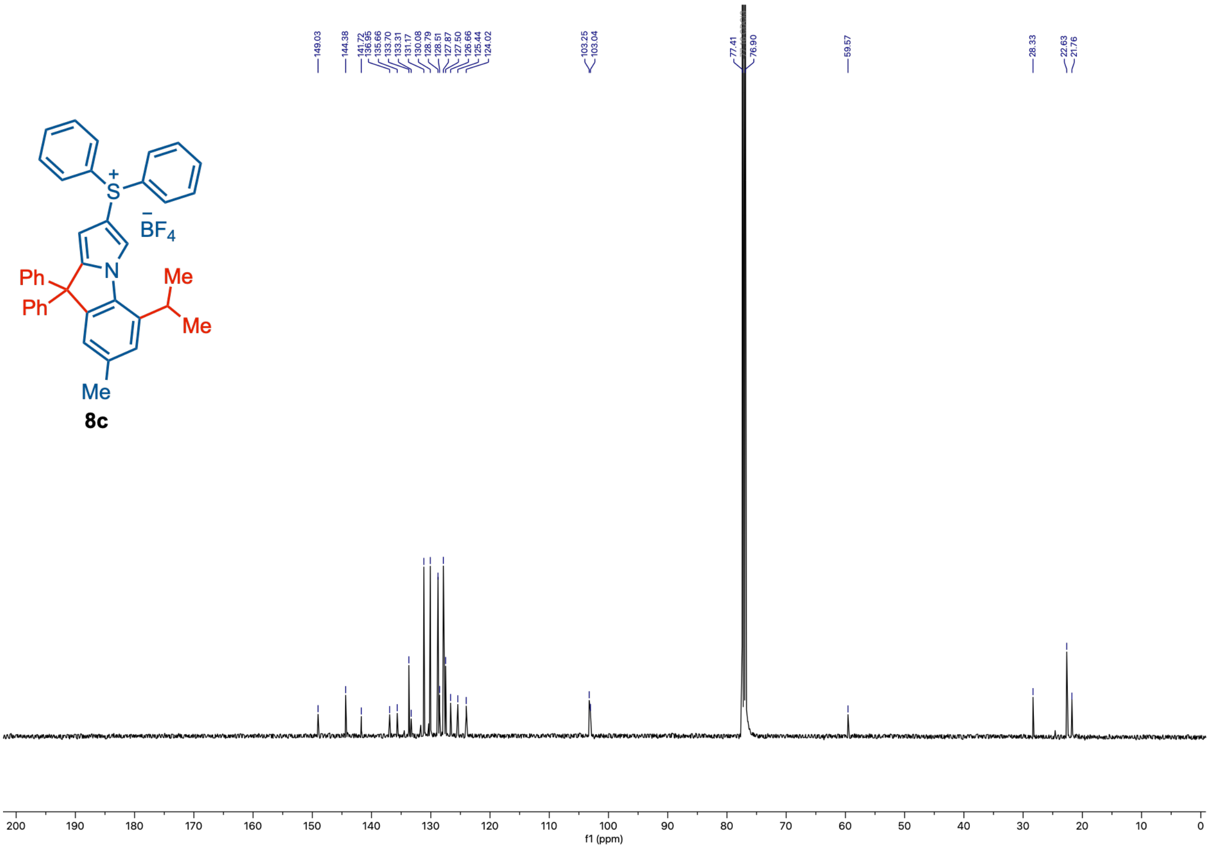

^{13}C NMR (126 MHz, $CDCl_3$) spectrum of **8c**

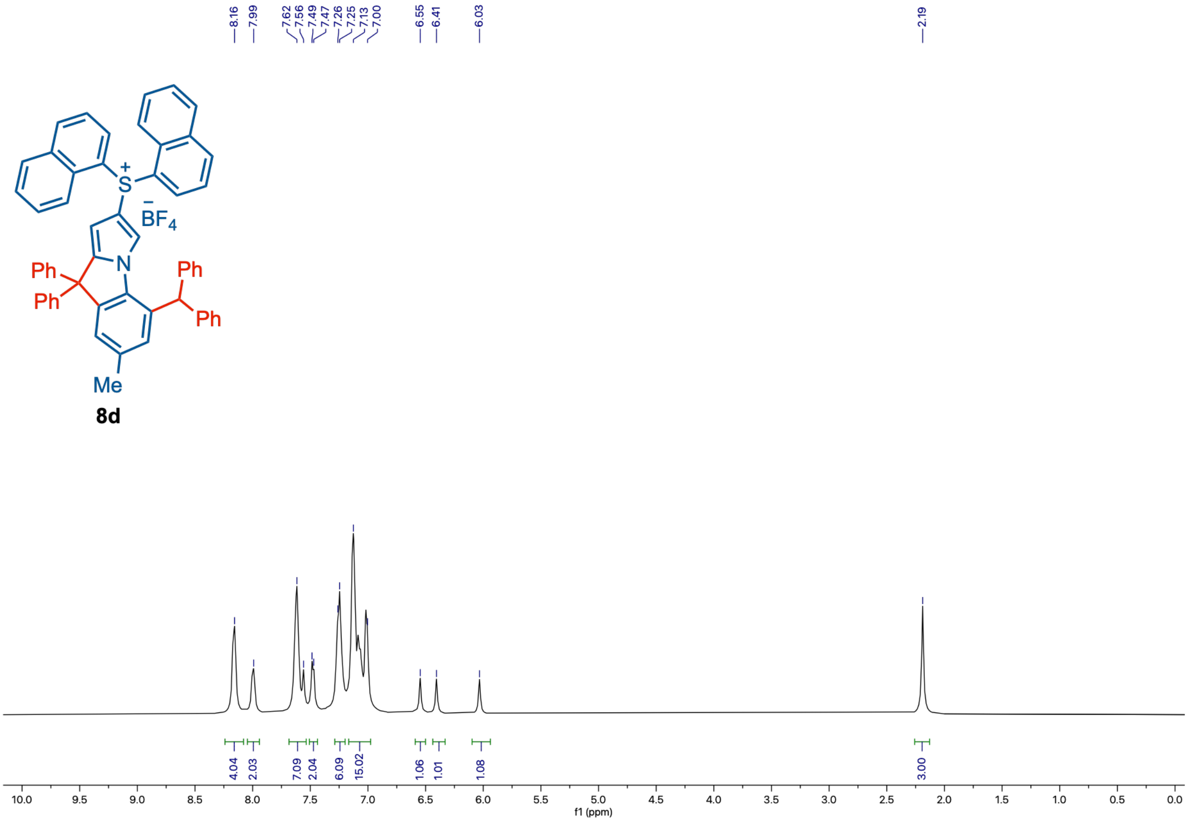

^{1}H NMR (500 MHz, CDCl$_3$) spectrum of **8d**

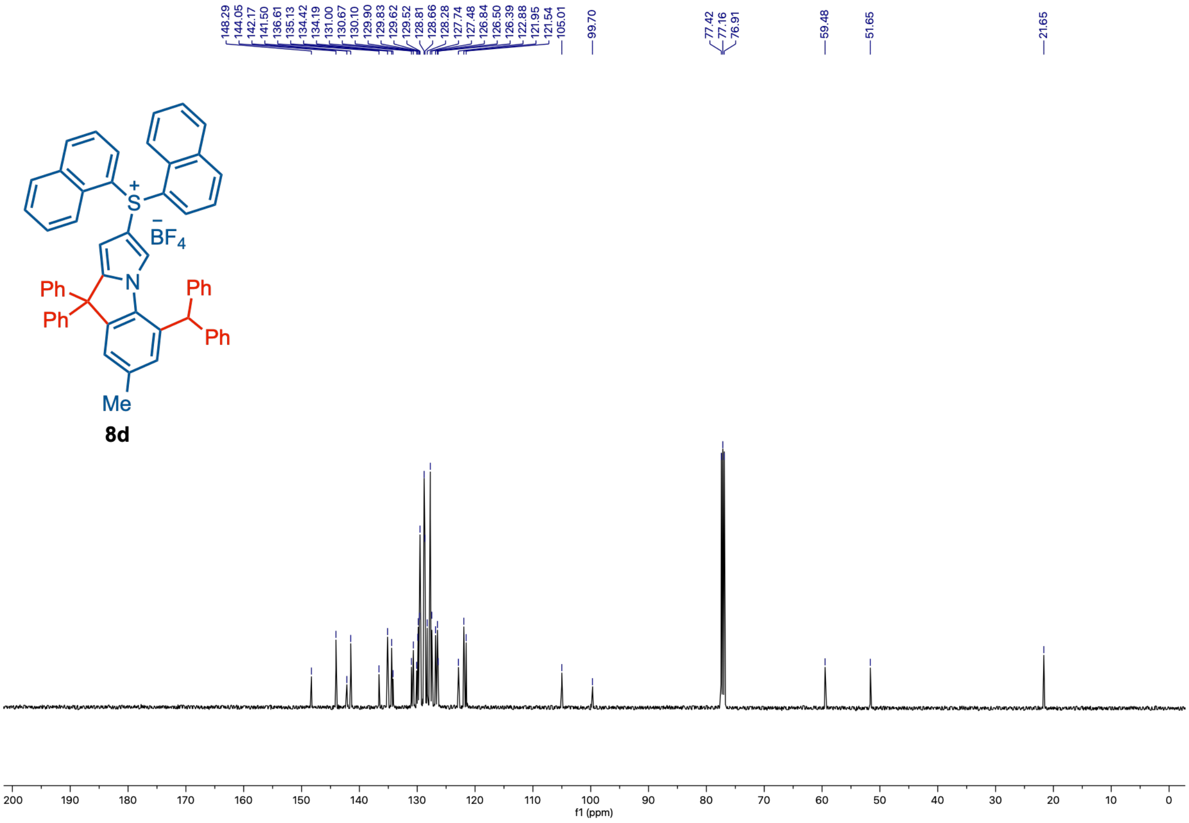

^{13}C NMR (126 MHz, CDCl$_3$) spectrum of **8d**

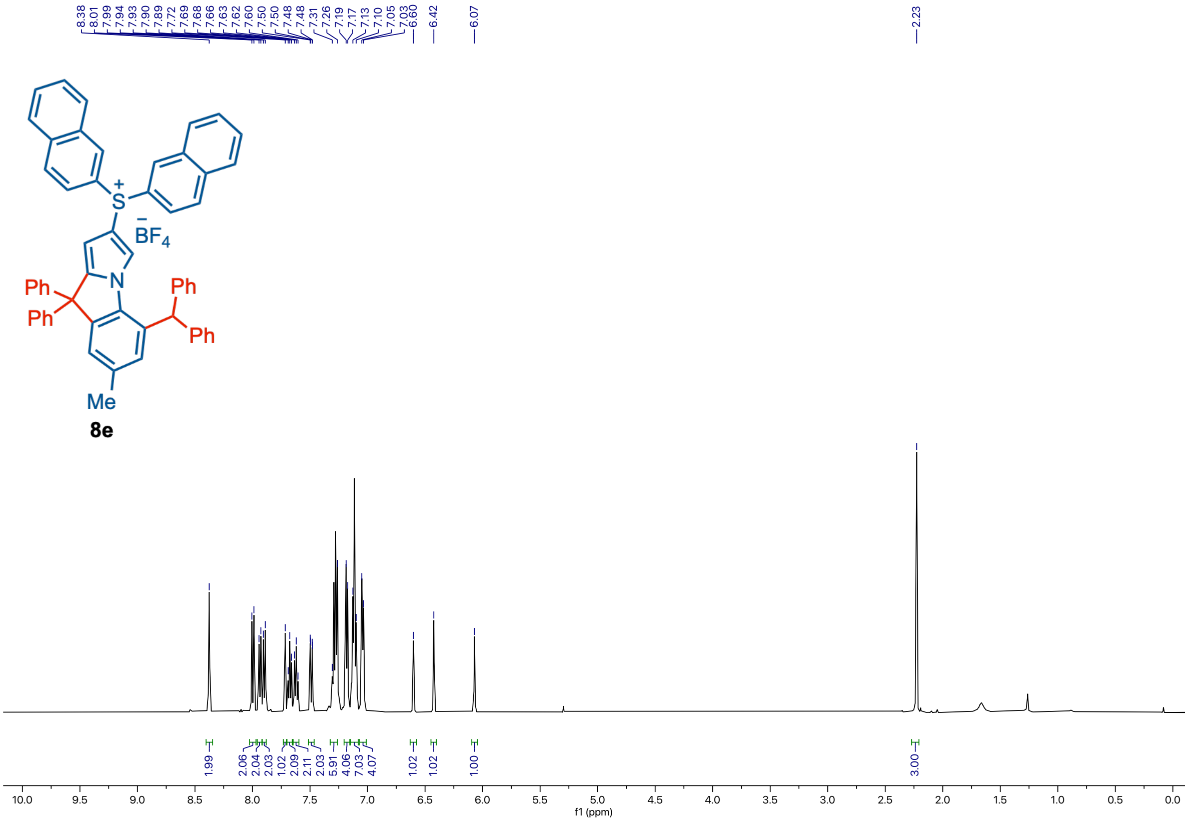

^{1}H NMR (500 MHz, $CDCl_3$) spectrum of **8e**

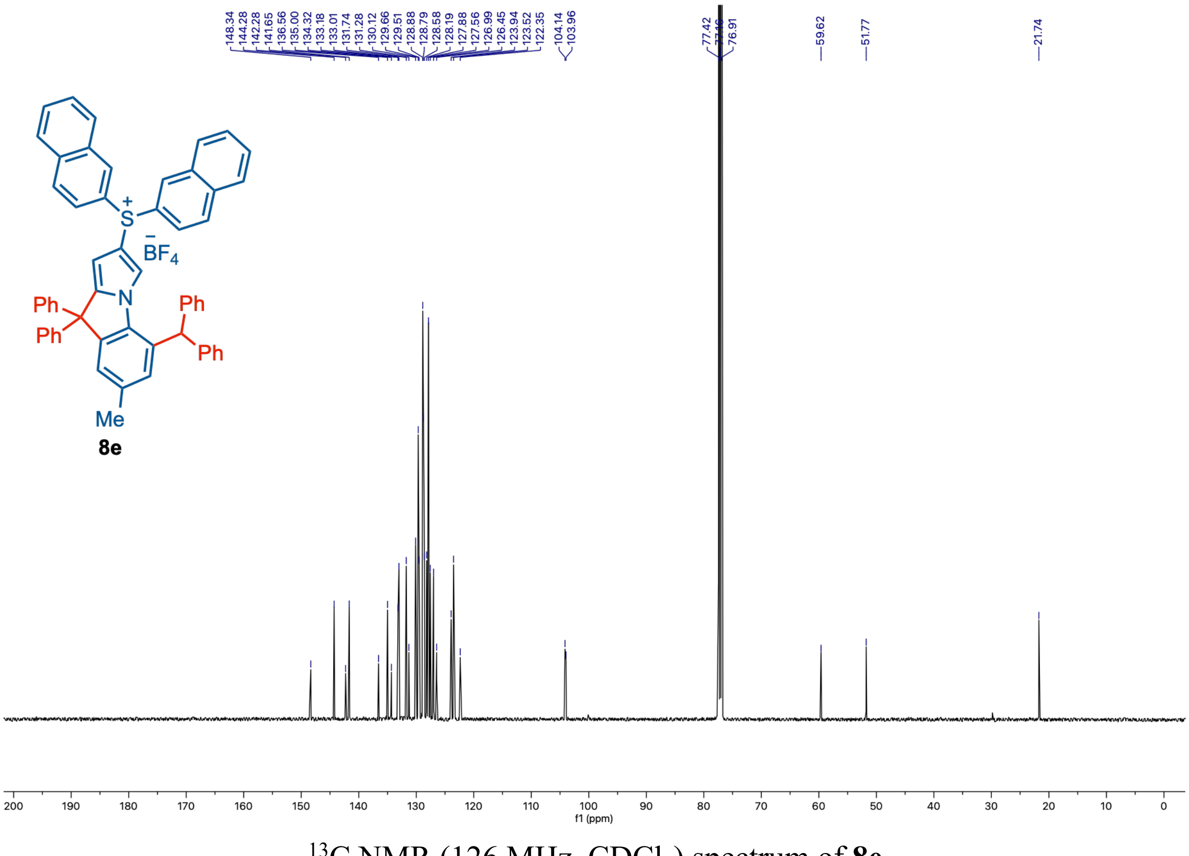

^{13}C NMR (126 MHz, $CDCl_3$) spectrum of **8e**

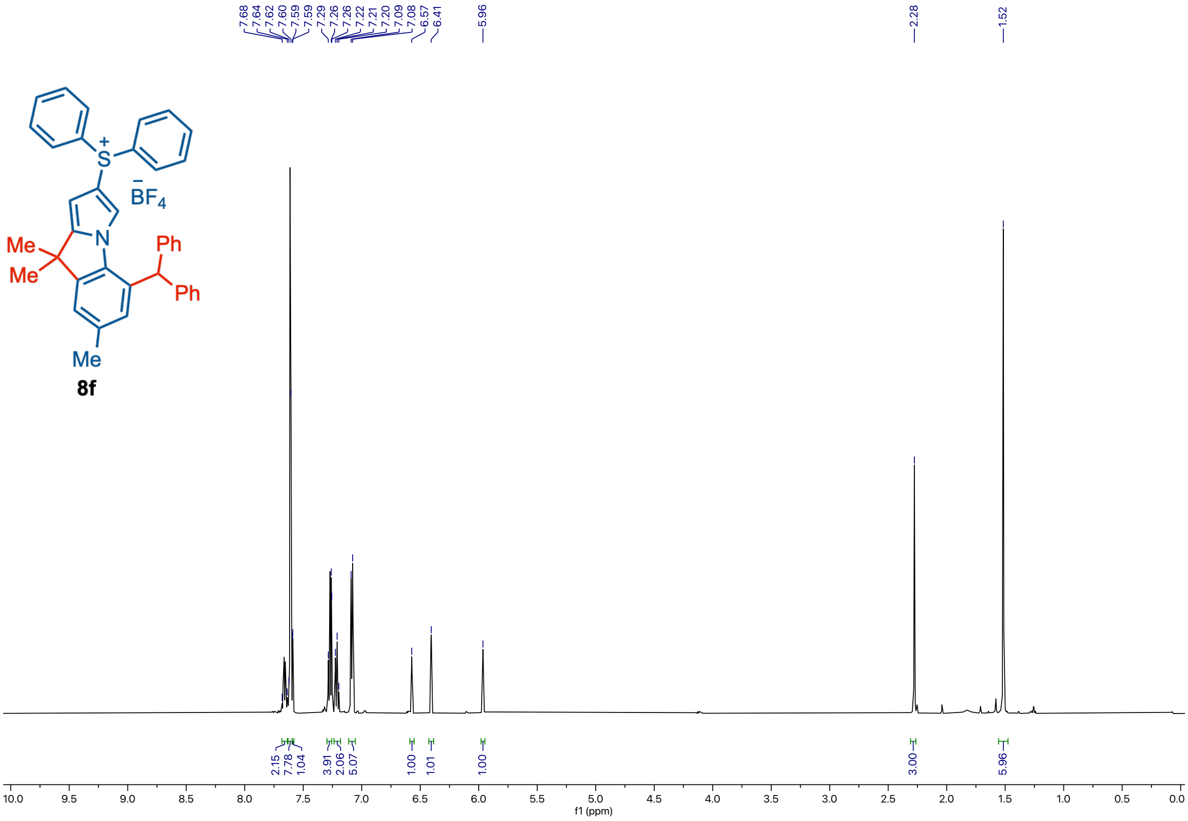

^{1}H NMR (500 MHz, $CDCl_3$) spectrum of **8f**

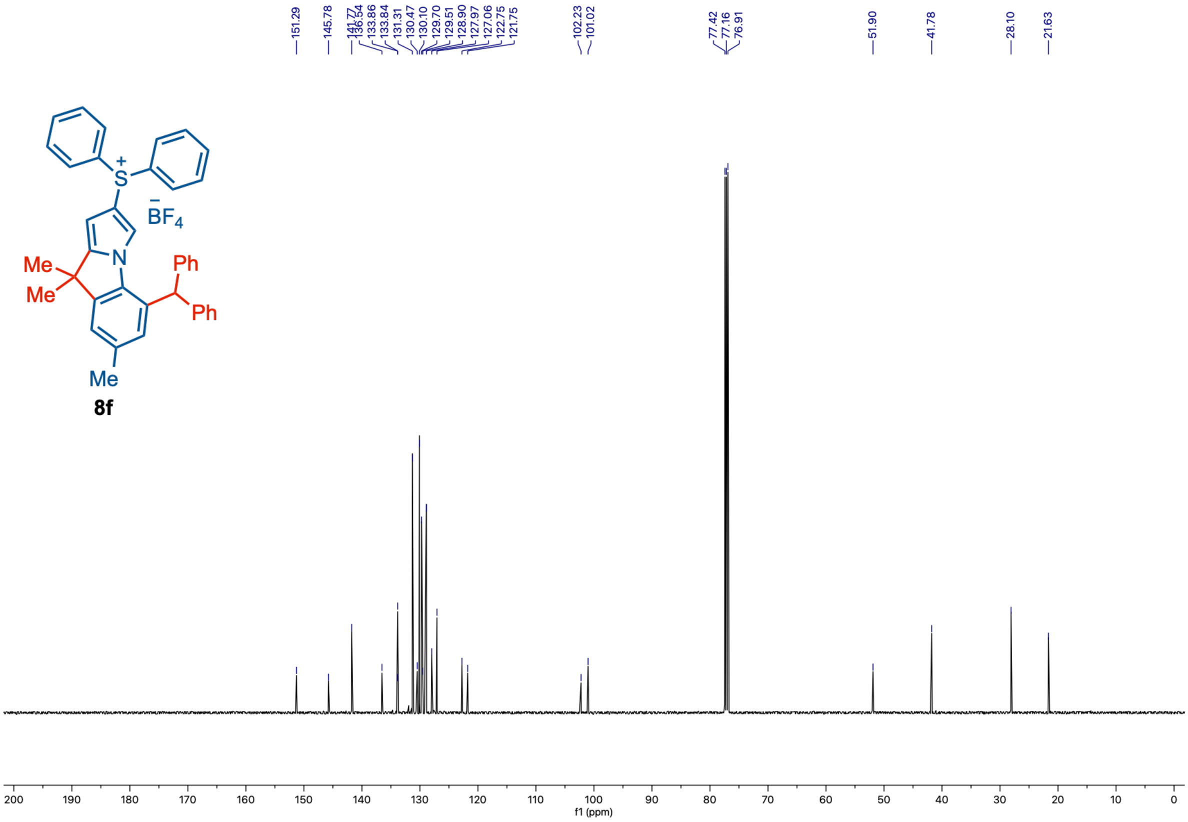

^{13}C NMR (126 MHz, $CDCl_3$) spectrum of **8f**

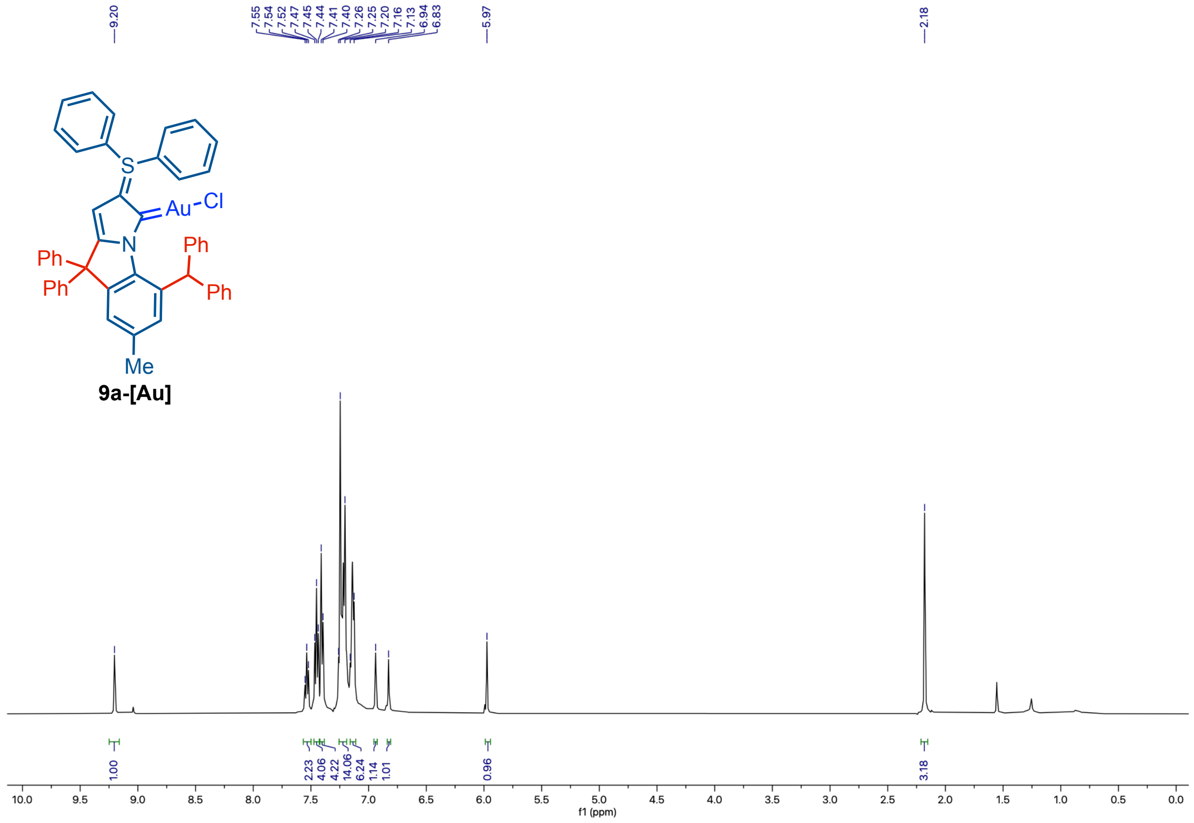

^{1}H NMR (500 MHz, $CDCl_3$) spectrum of **9a-[Au]**

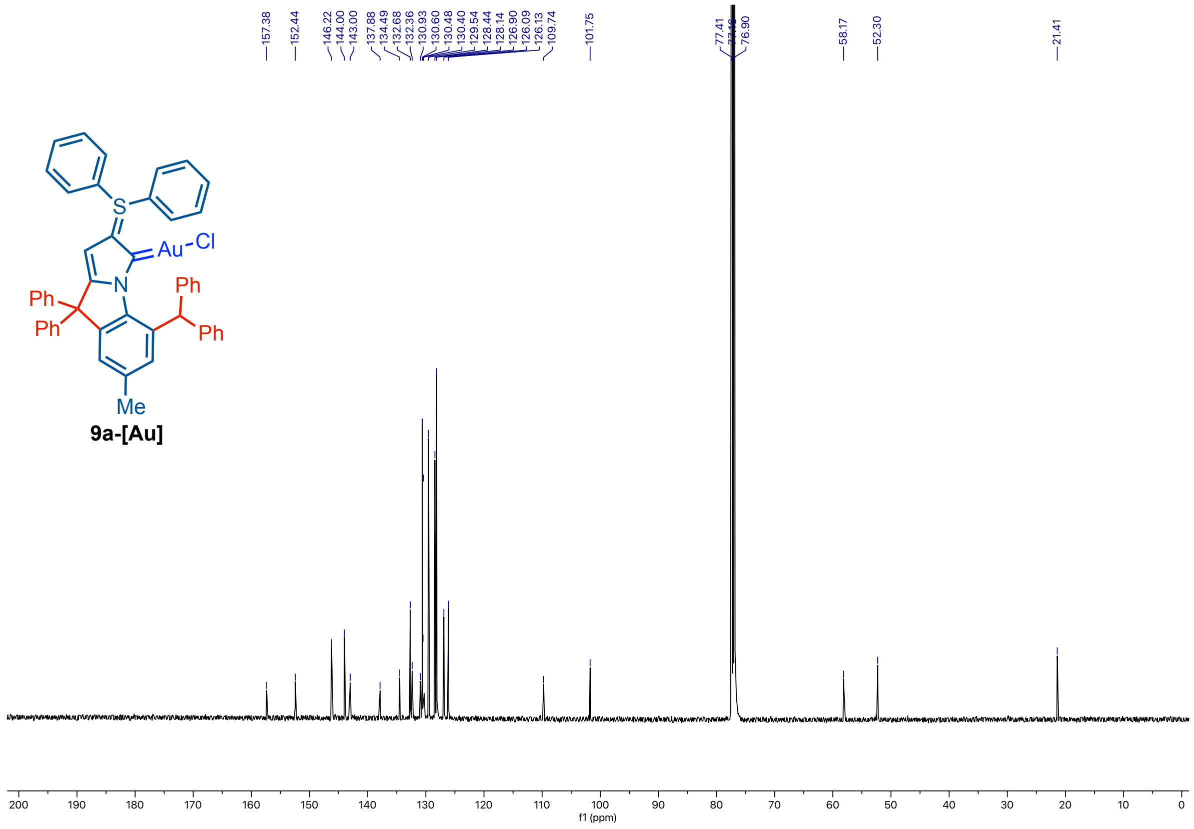

^{13}C NMR (126 MHz, $CDCl_3$) spectrum of **9a-[Au]**

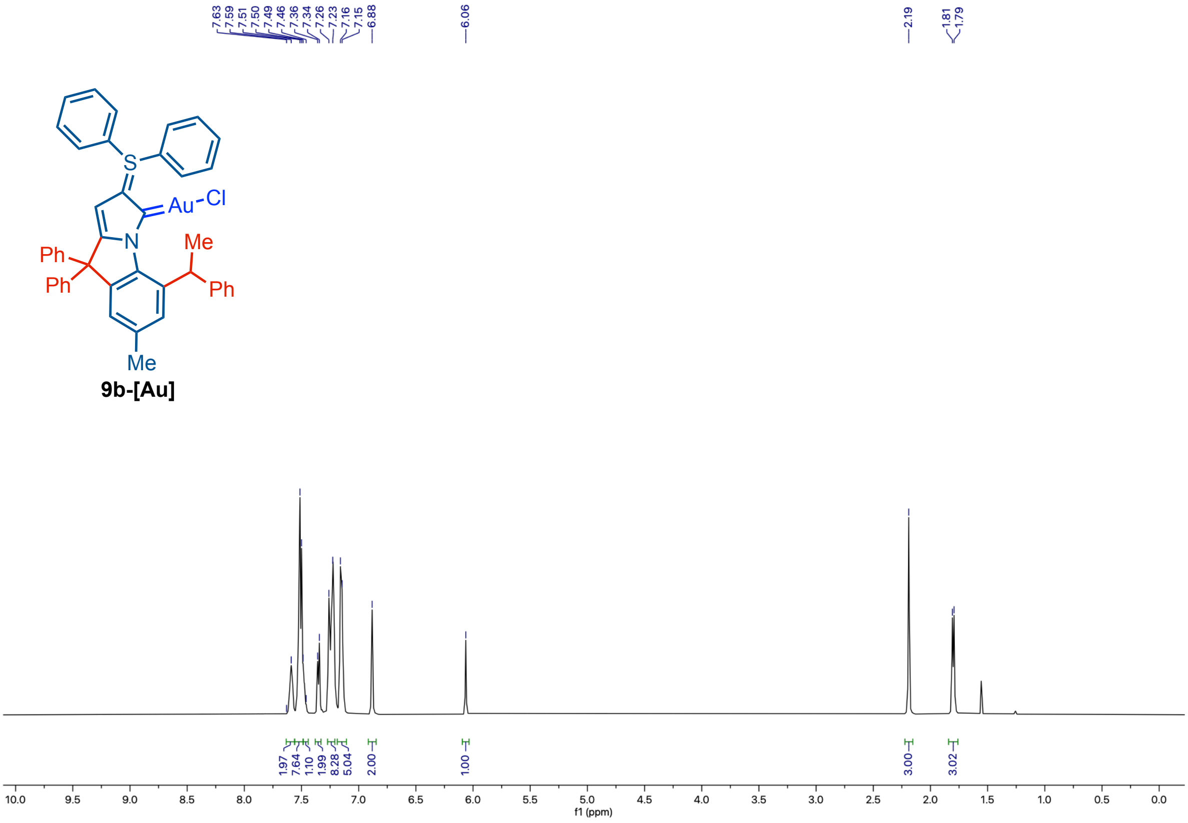

^{1}H NMR (500 MHz, CDCl$_3$) spectrum of **9b-[Au]**

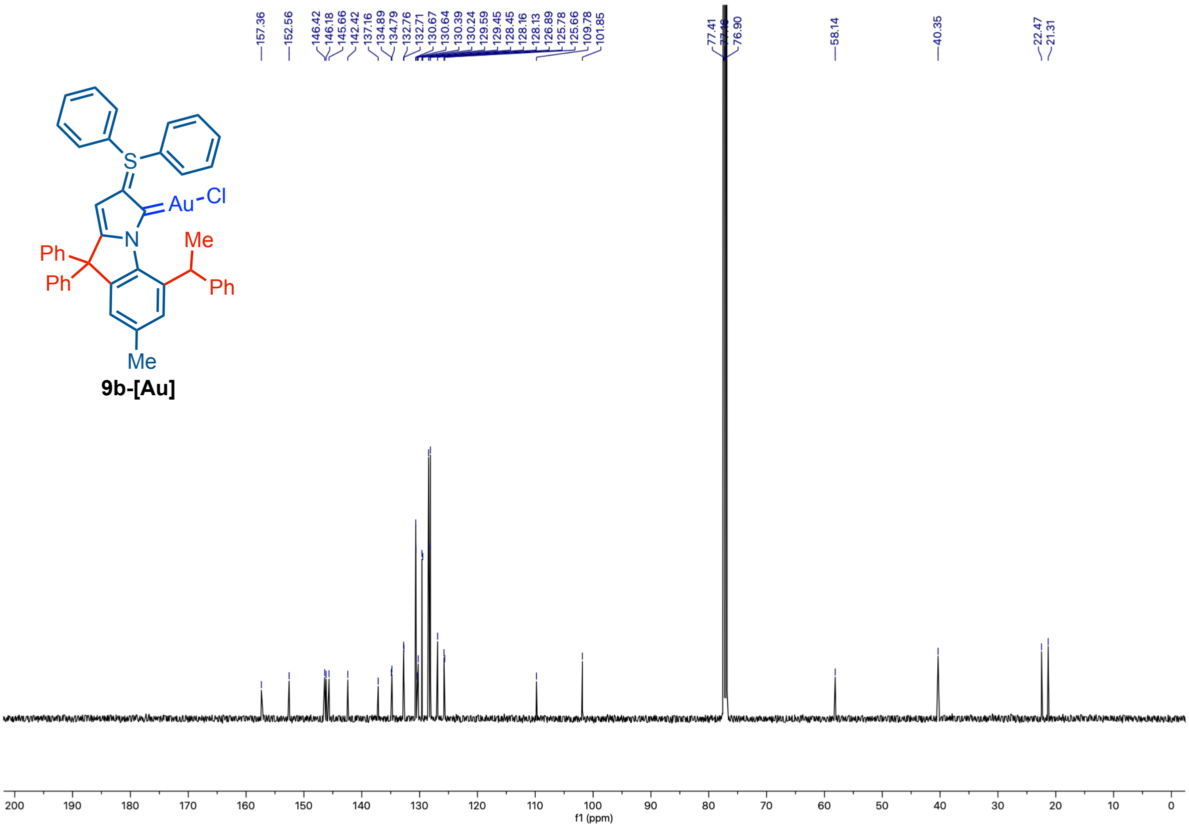

^{13}C NMR (126 MHz, CDCl$_3$) spectrum of **9b-[Au]**

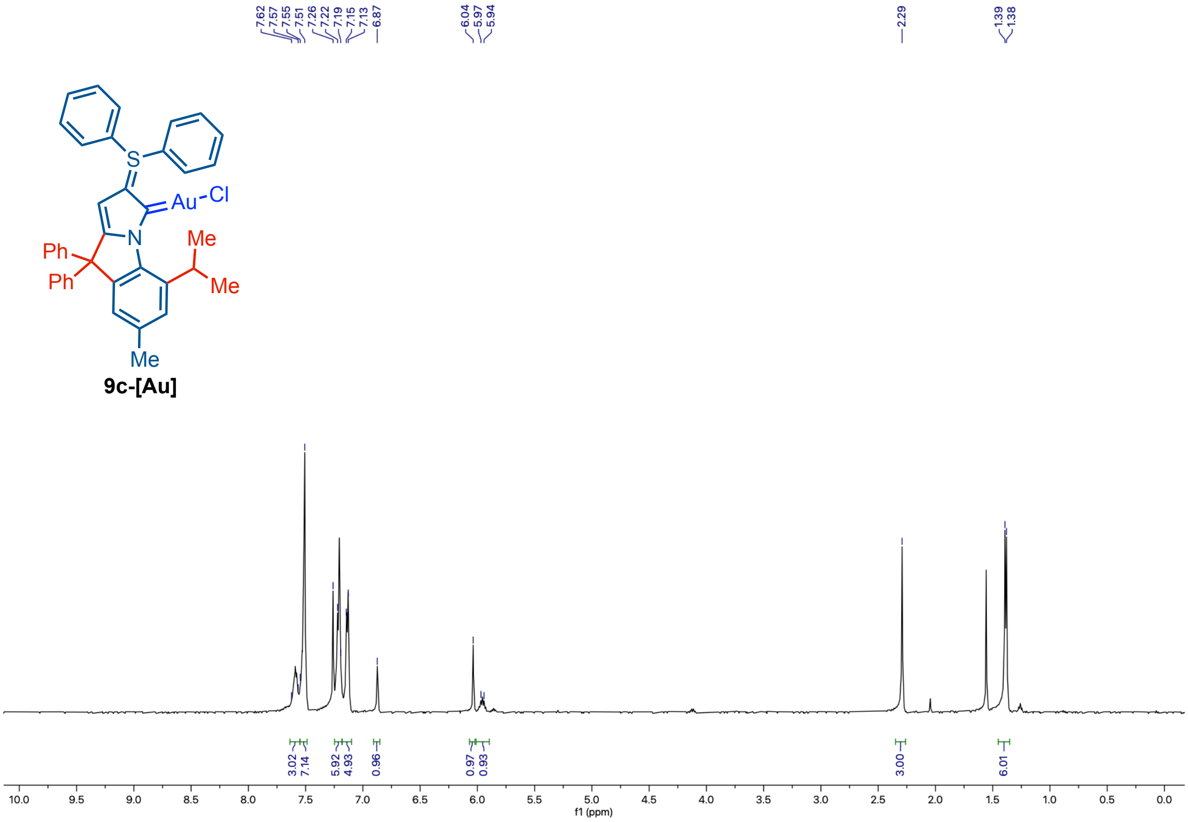

^{1}H NMR (500 MHz, $CDCl_3$) spectrum of **9c-[Au]**

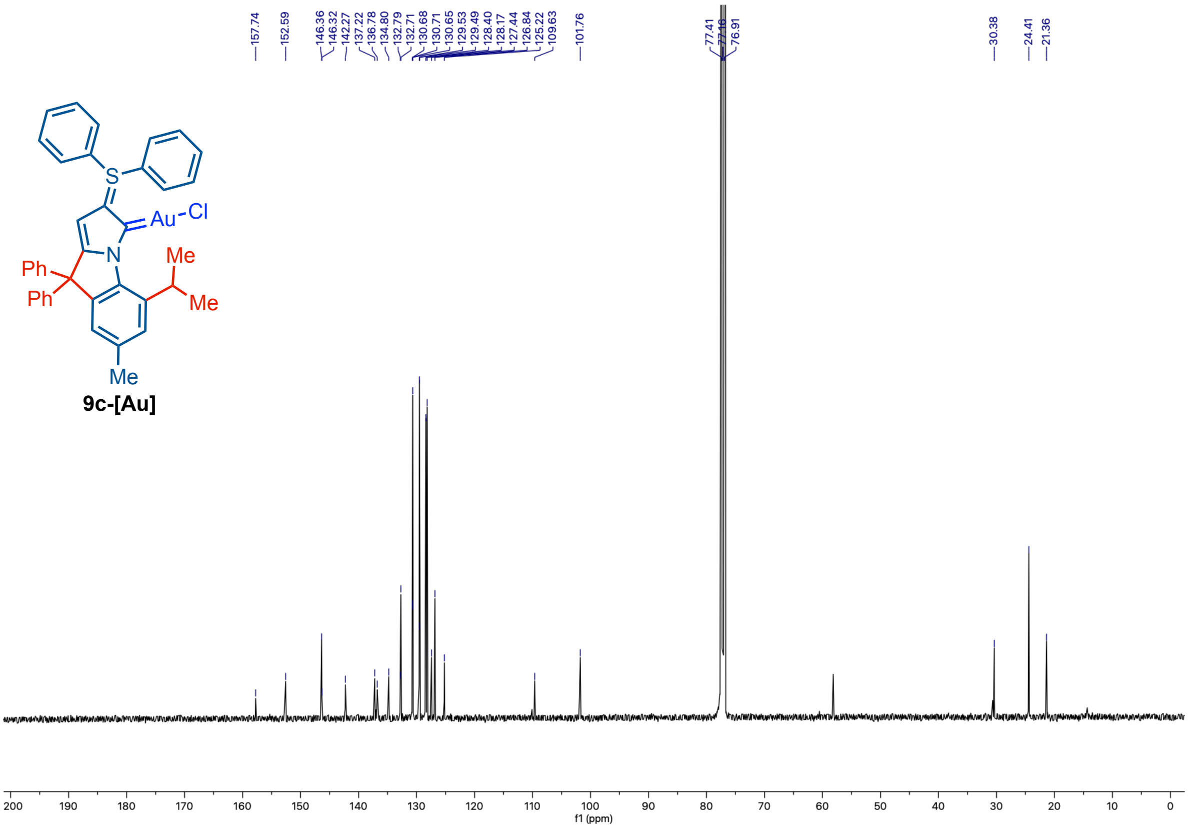

^{13}C NMR (126 MHz, $CDCl_3$) spectrum of **9c-[Au]**

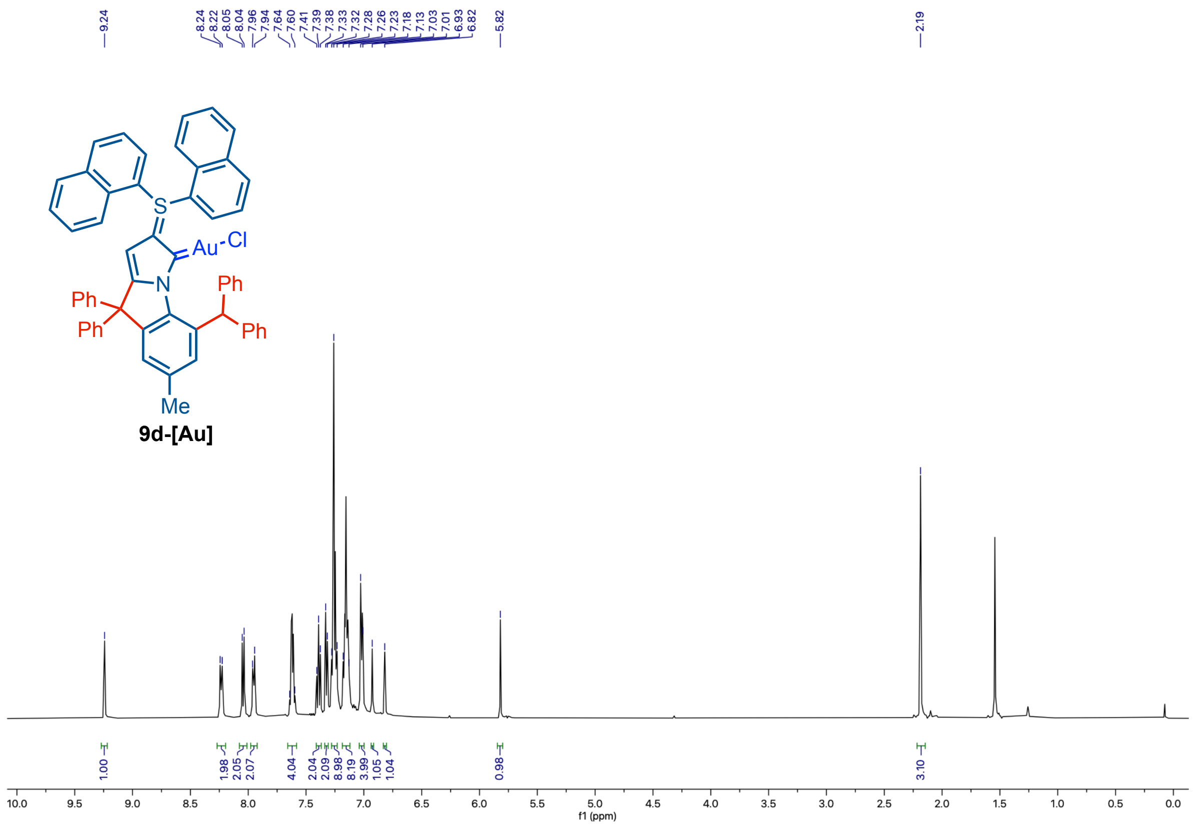

^1H NMR (500 MHz, $CDCl_3$) spectrum of **9d-[Au]**

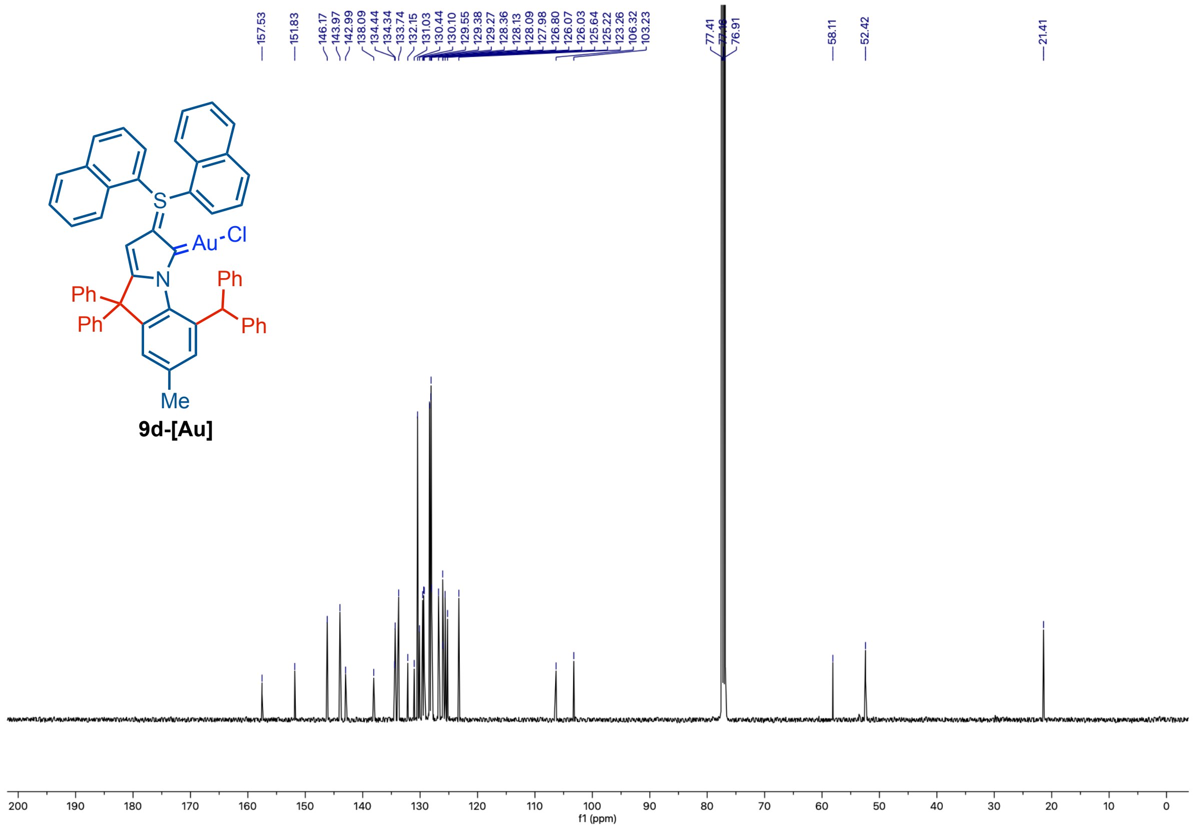

^{13}C NMR (126 MHz, $CDCl_3$) spectrum of **9d-[Au]**

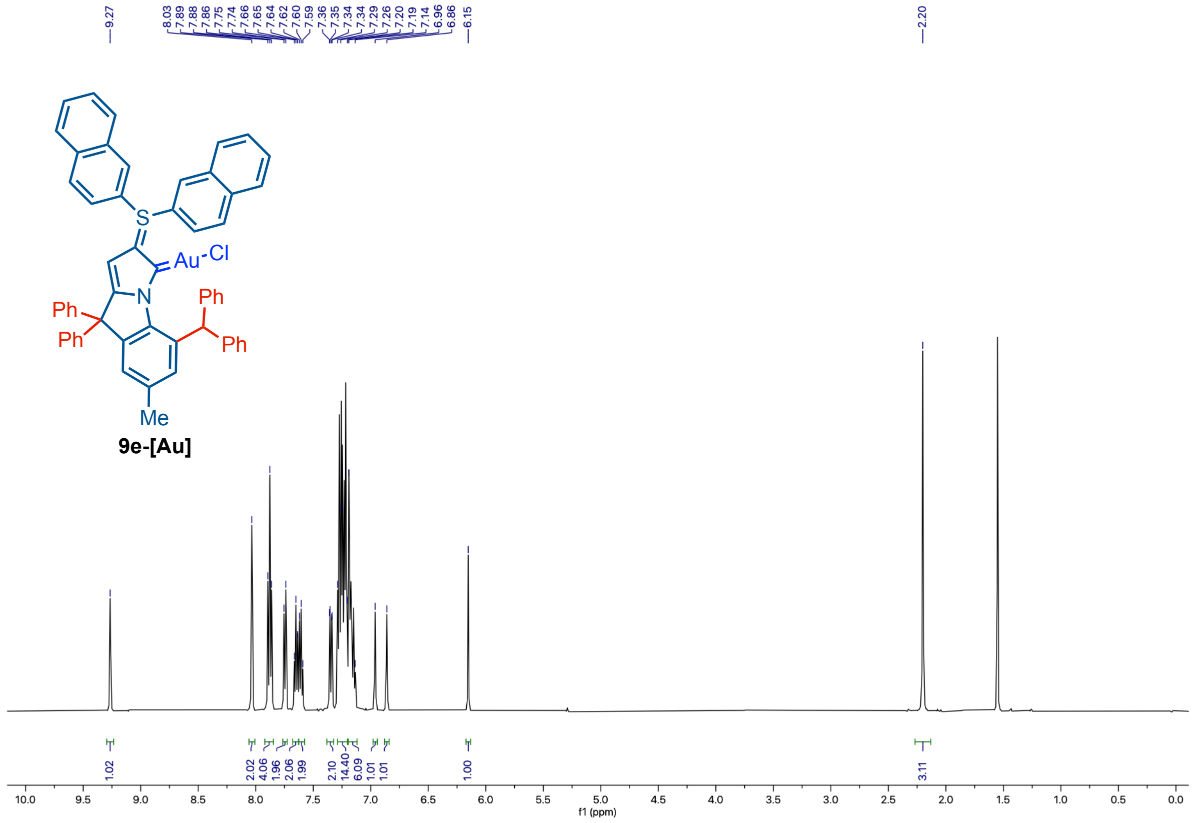

^{1}H NMR (500 MHz, $CDCl_3$) spectrum of **9e**

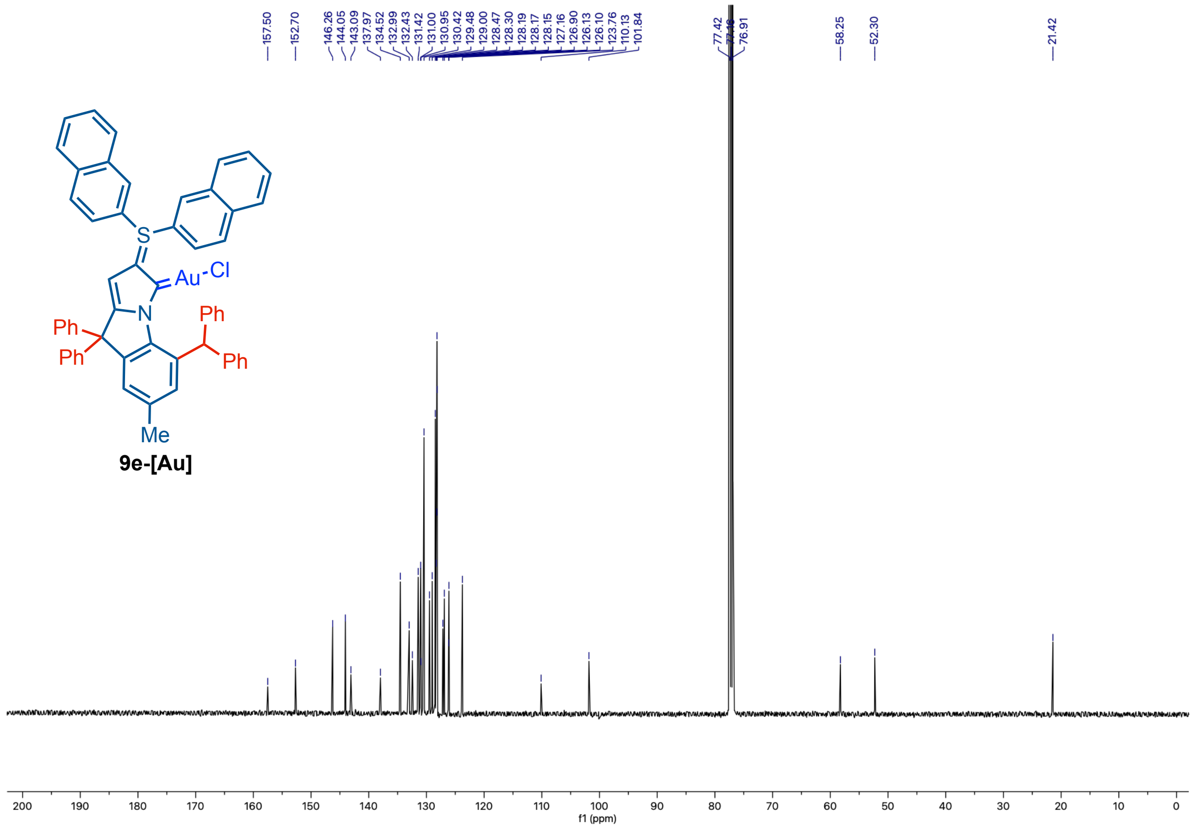

^{13}C NMR (126 MHz, $CDCl_3$) spectrum of **9e**

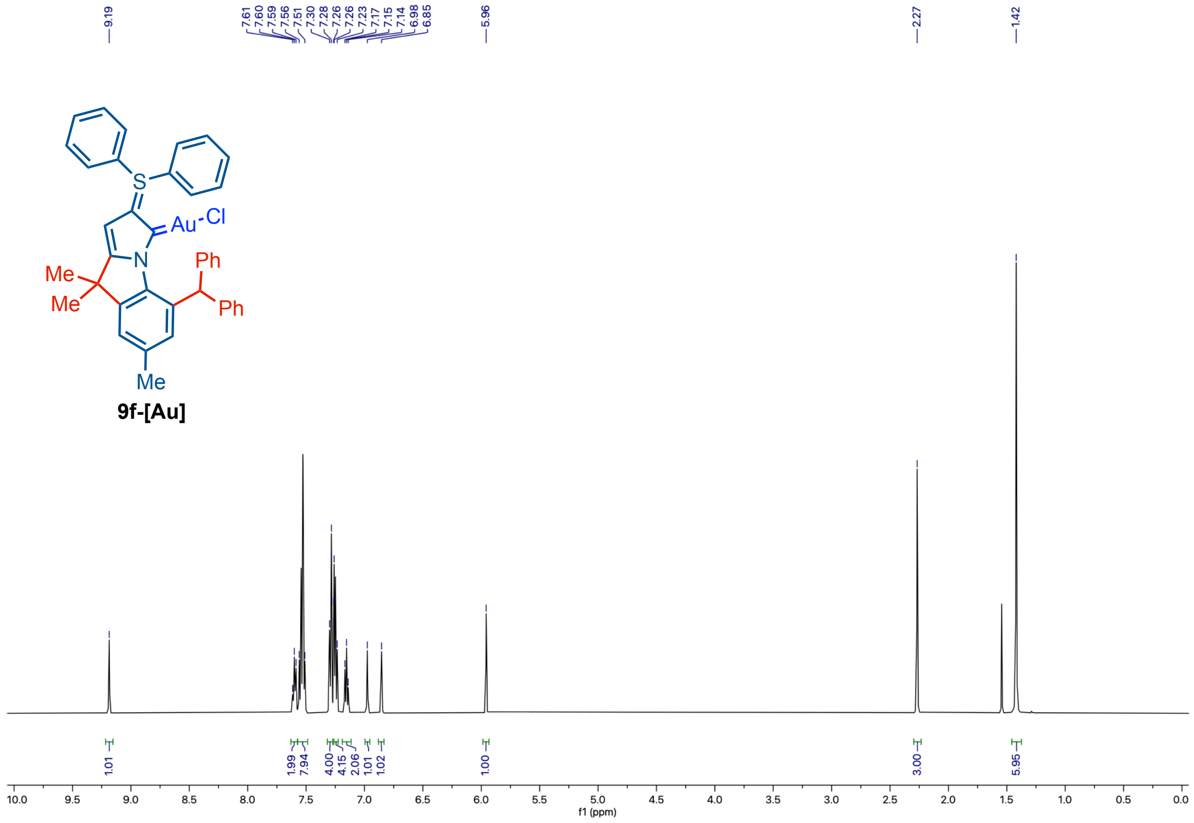

^{1}H NMR (500 MHz, $CDCl_3$) spectrum of **9f**

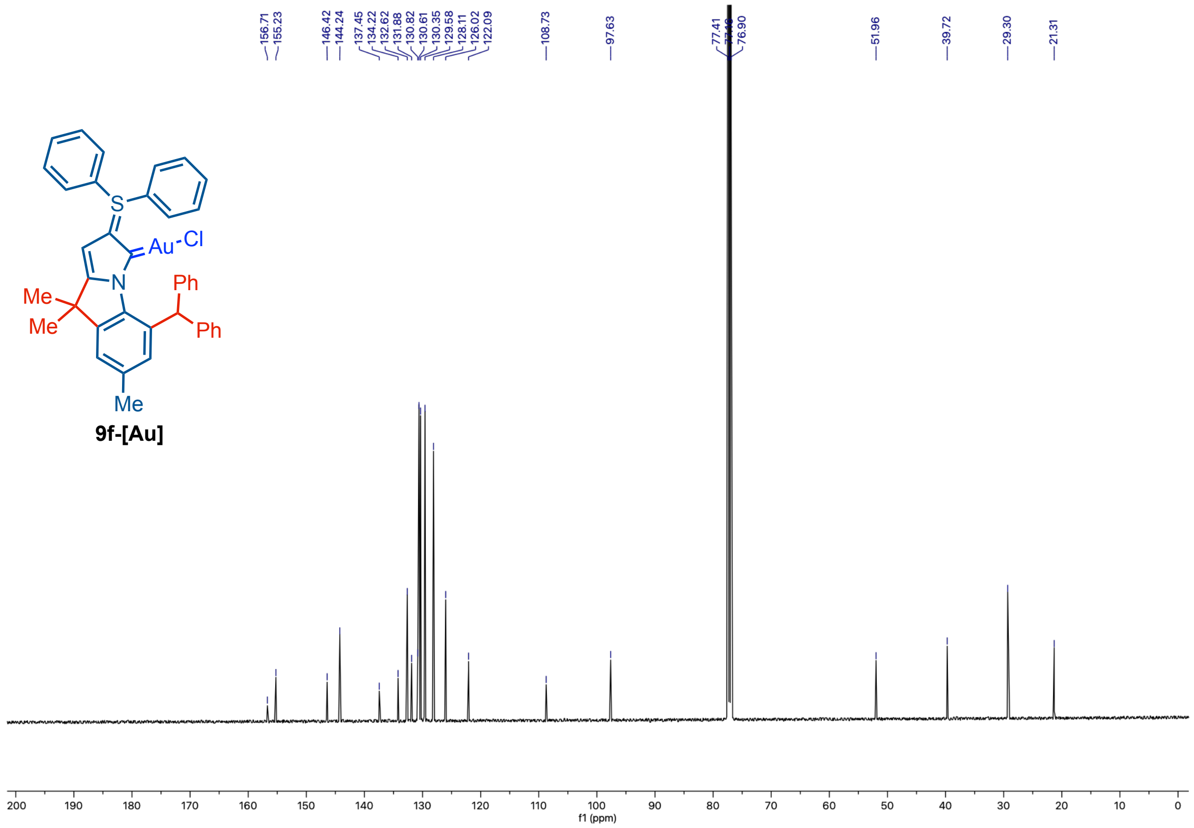

^{13}C NMR (126 MHz, $CDCl_3$) spectrum of **9f**

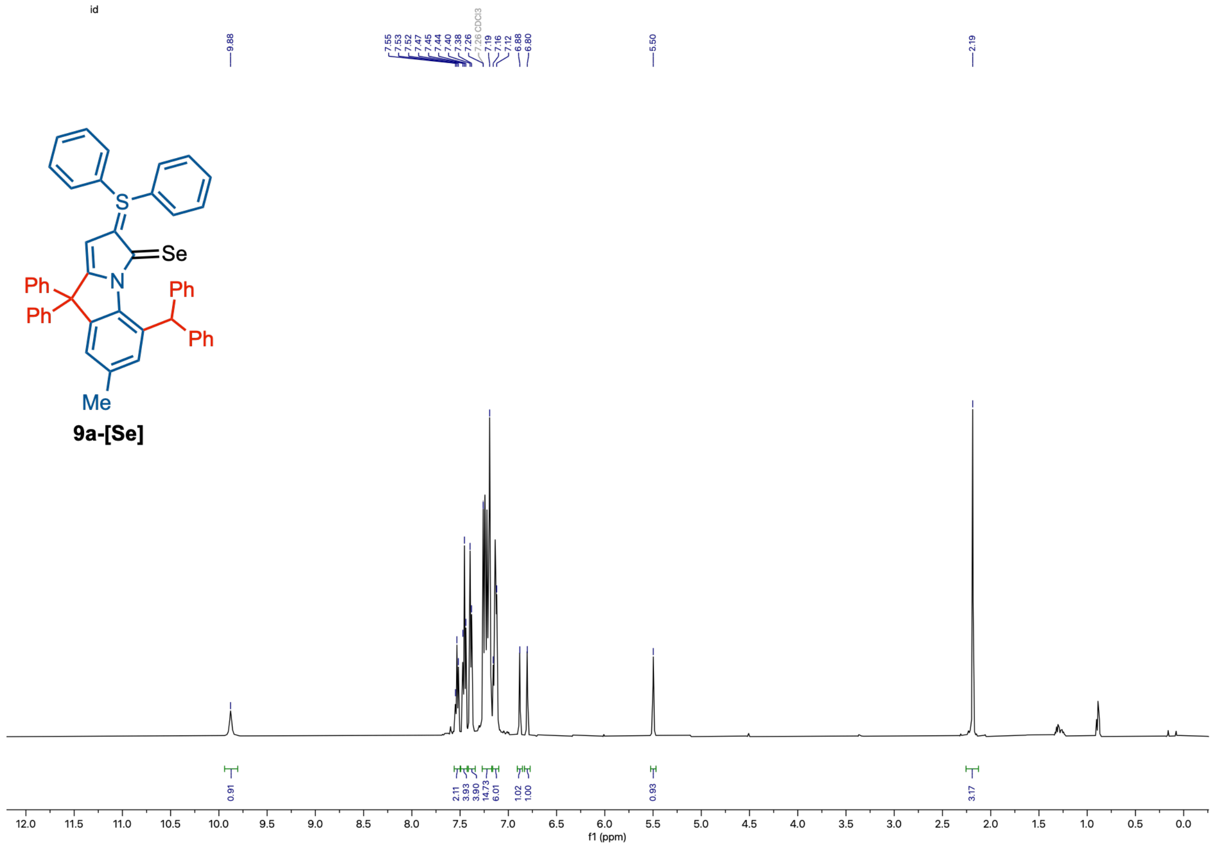

^{1}H NMR (500 MHz, $CDCl_3$) spectrum of **9a-[Se]**

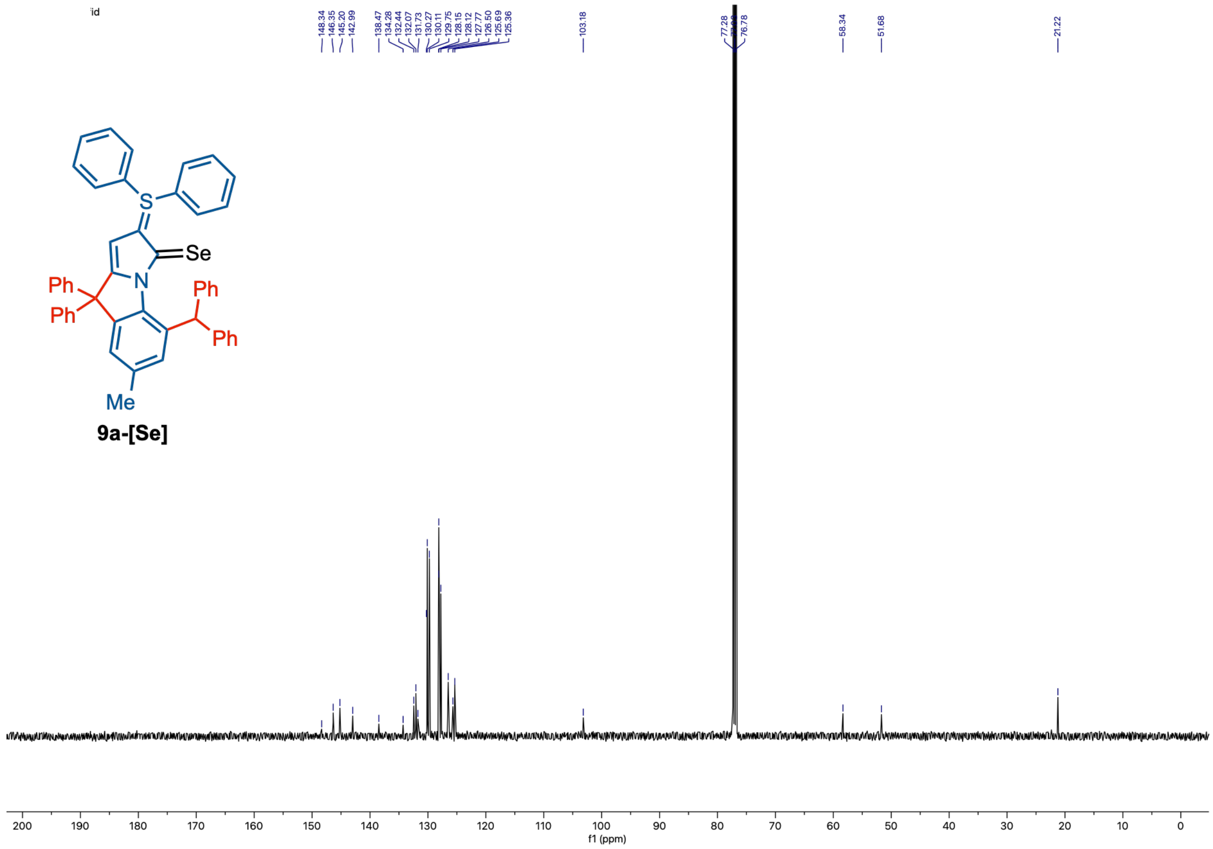

^{13}C NMR (126 MHz, $CDCl_3$) spectrum of **9a-[Se]**

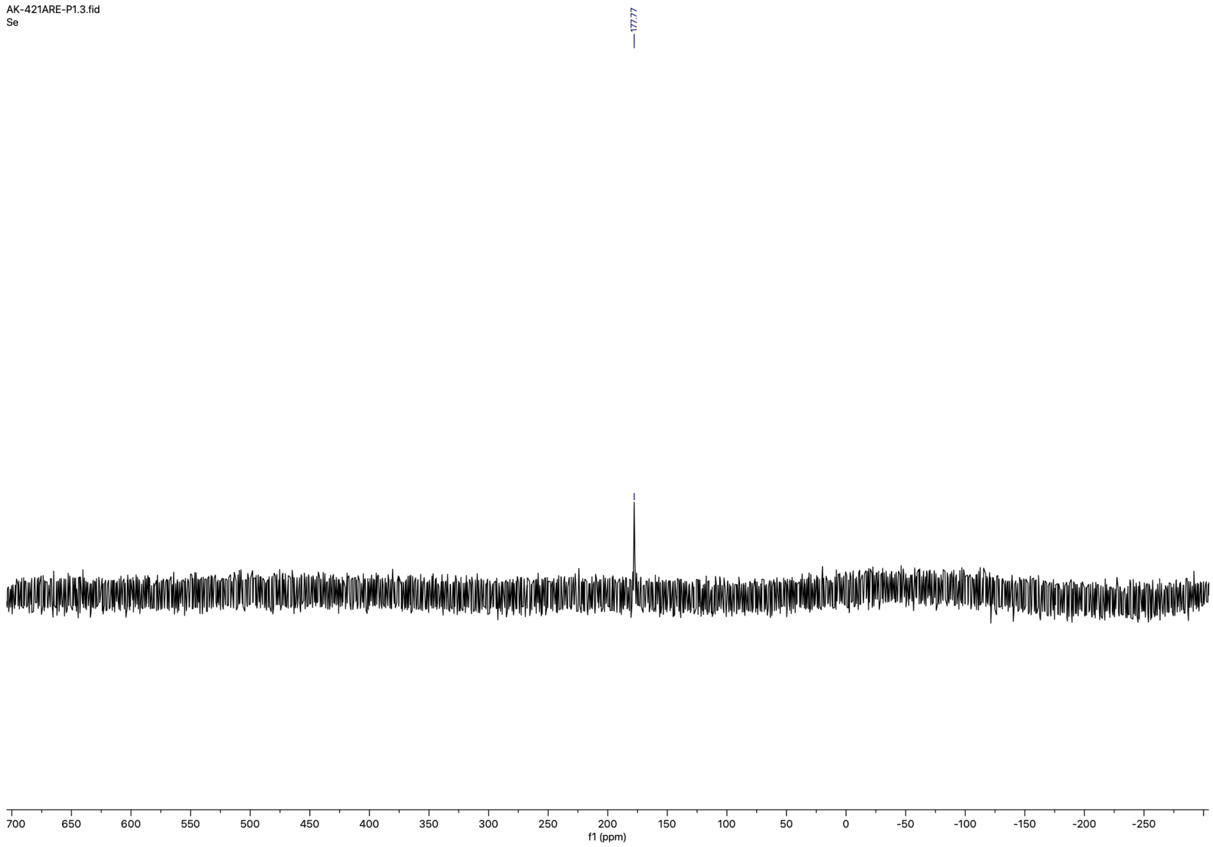

^{77}C NMR (95 MHz, $CDCl_3$) spectrum of **9a-[Se]**

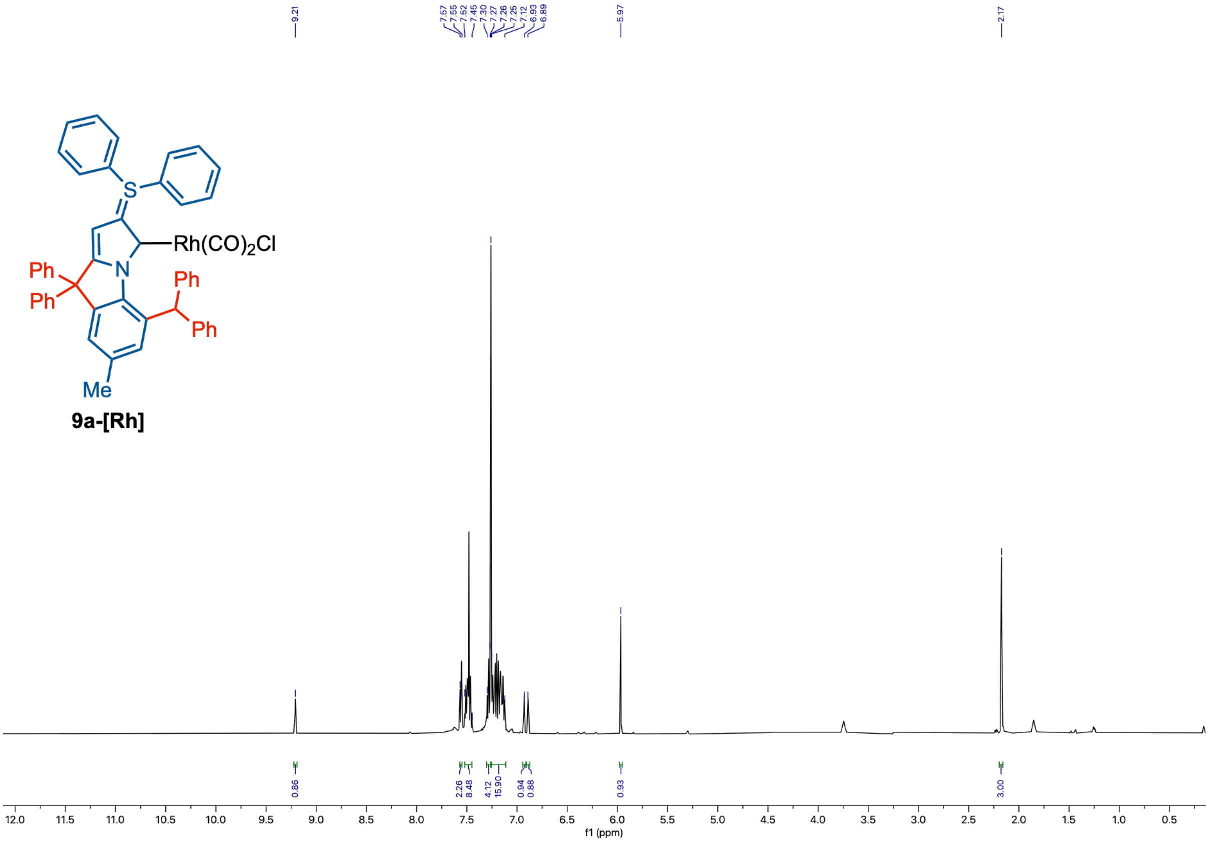

^{1}H NMR (500 MHz, $CDCl_3$) spectrum of **9a-[Rh]**

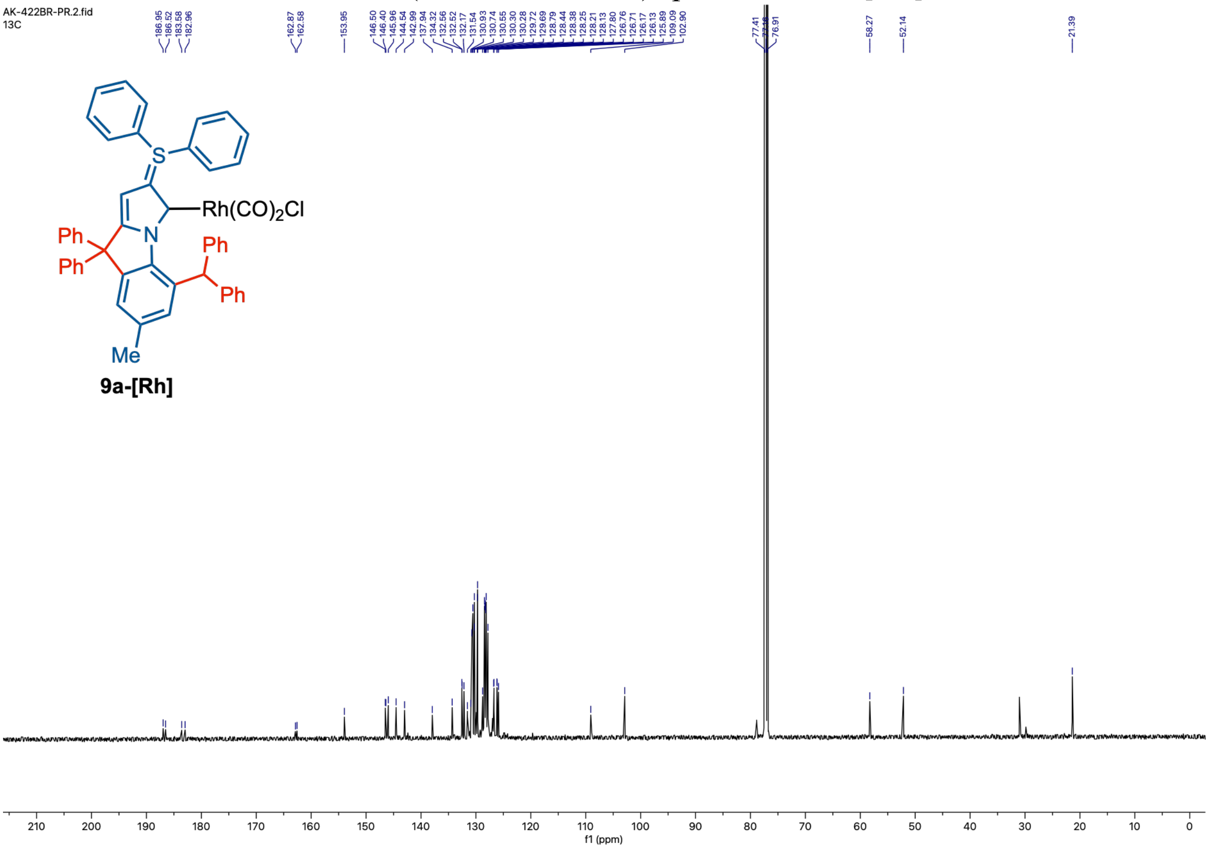

^{13}C NMR (126 MHz, $CDCl_3$) spectrum of **9a-[Rh]**

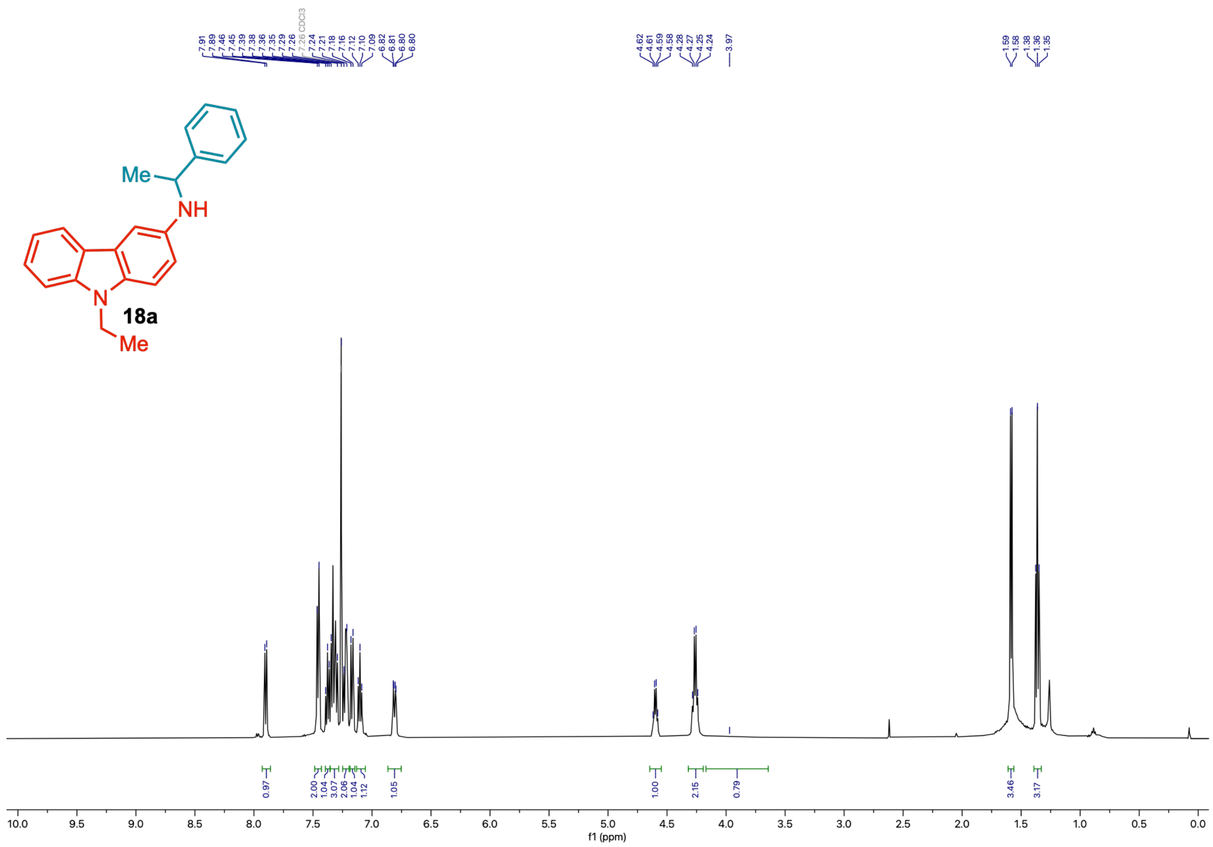

^{1}H NMR (500 MHz, $CDCl_3$) spectrum of **18a**

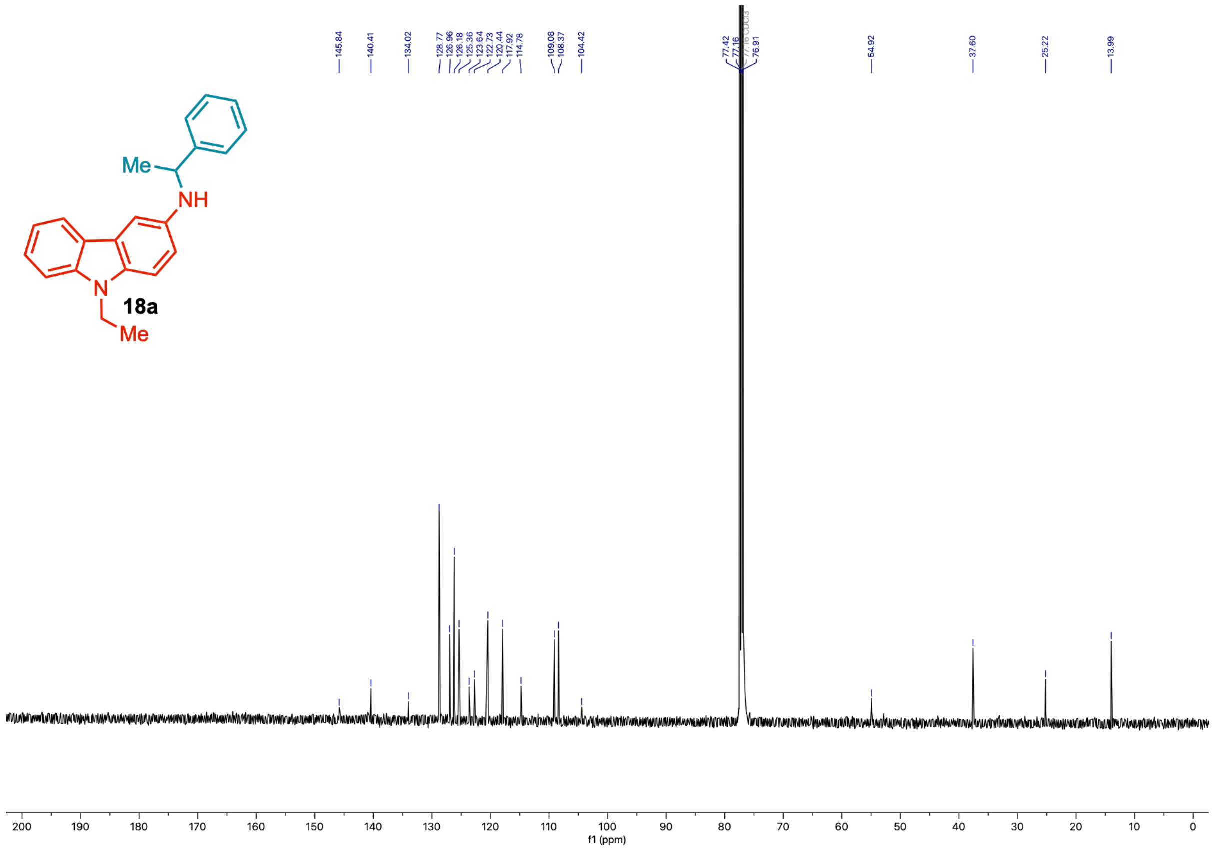

^{13}C NMR (126 MHz, $CDCl_3$) spectrum of **18a**

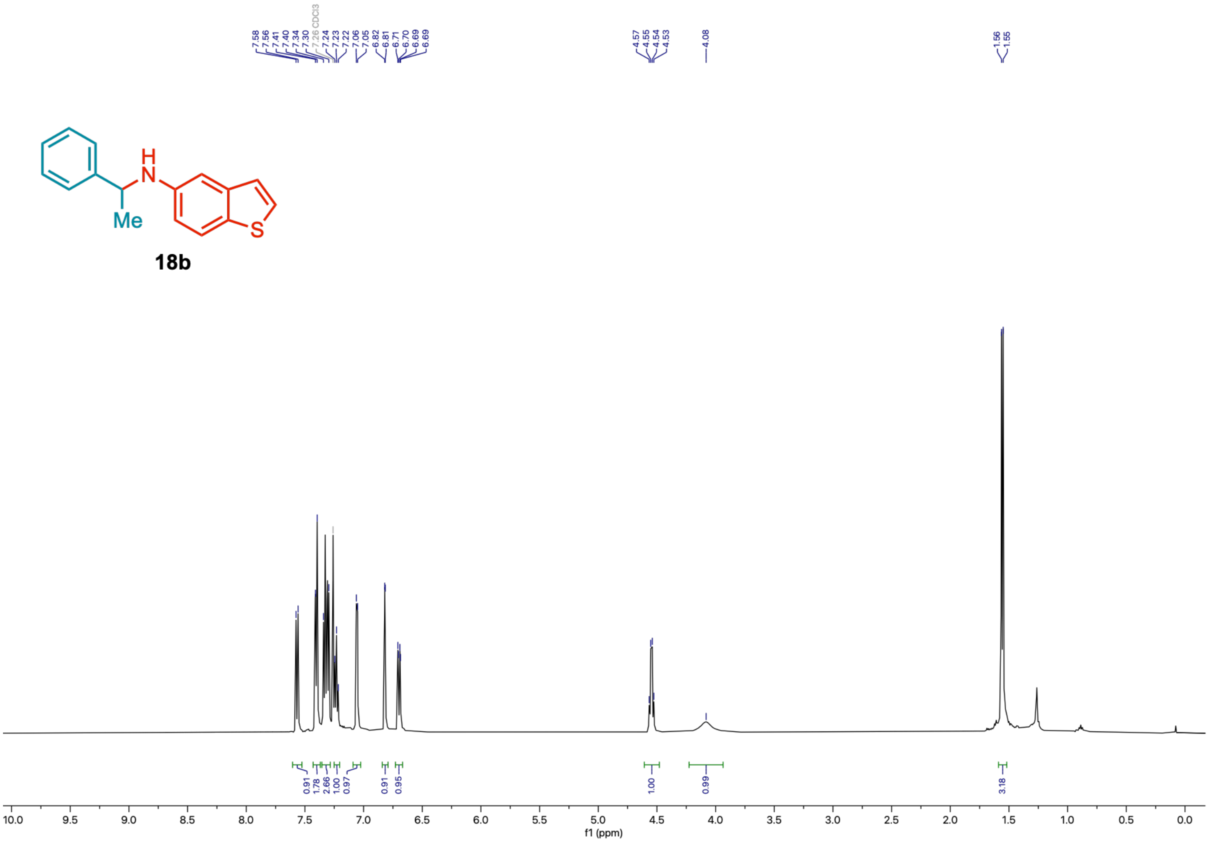

^{1}H NMR (500 MHz, $CDCl_3$) spectrum of **18b**

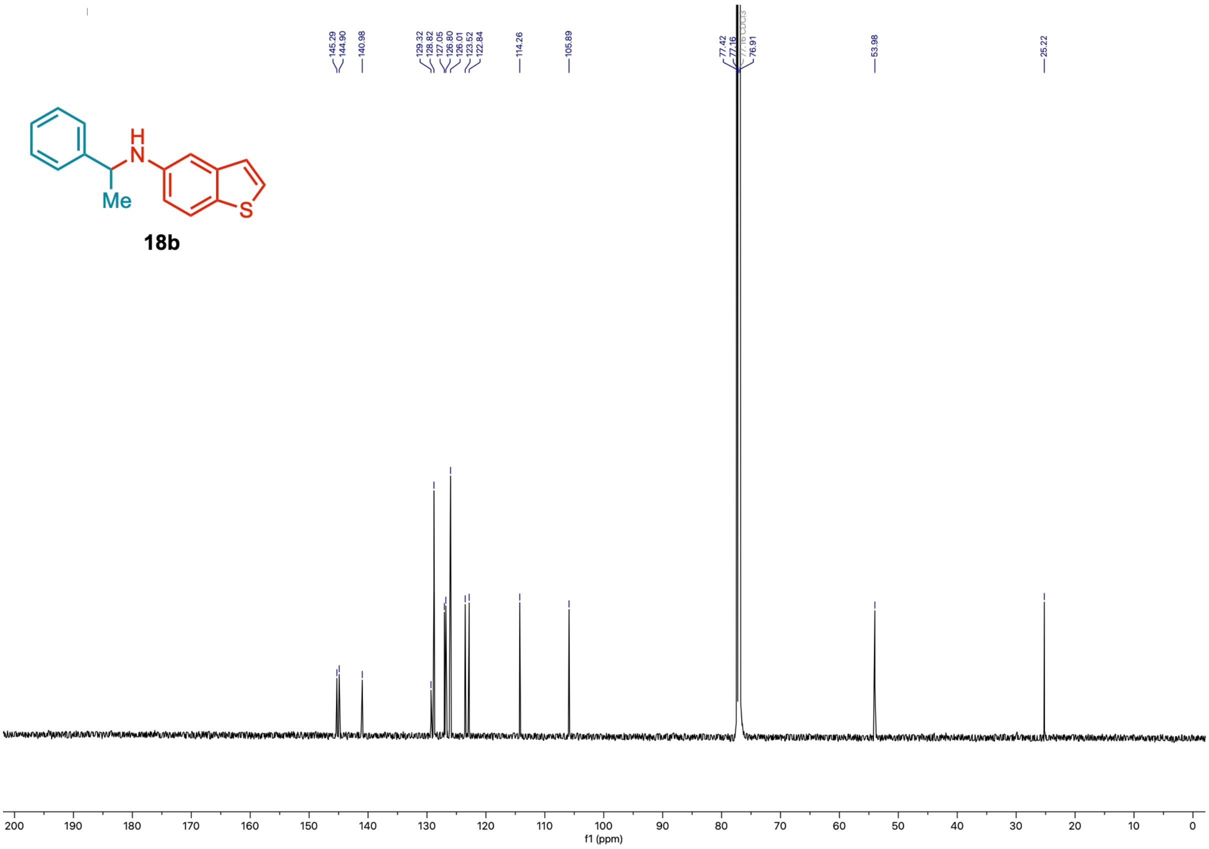

^{13}C NMR (126 MHz, $CDCl_3$) spectrum of **18b**

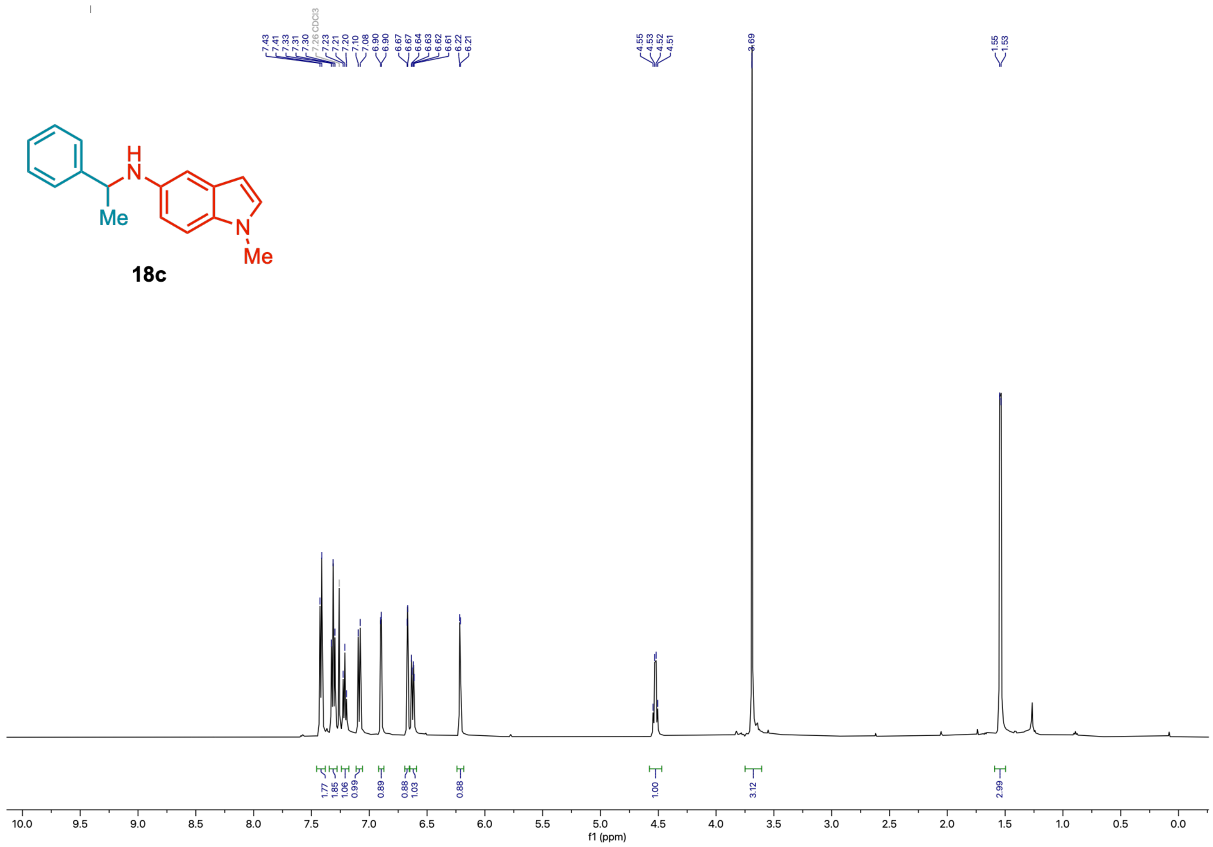

^{1}H NMR (500 MHz, $CDCl_3$) spectrum of **18c**

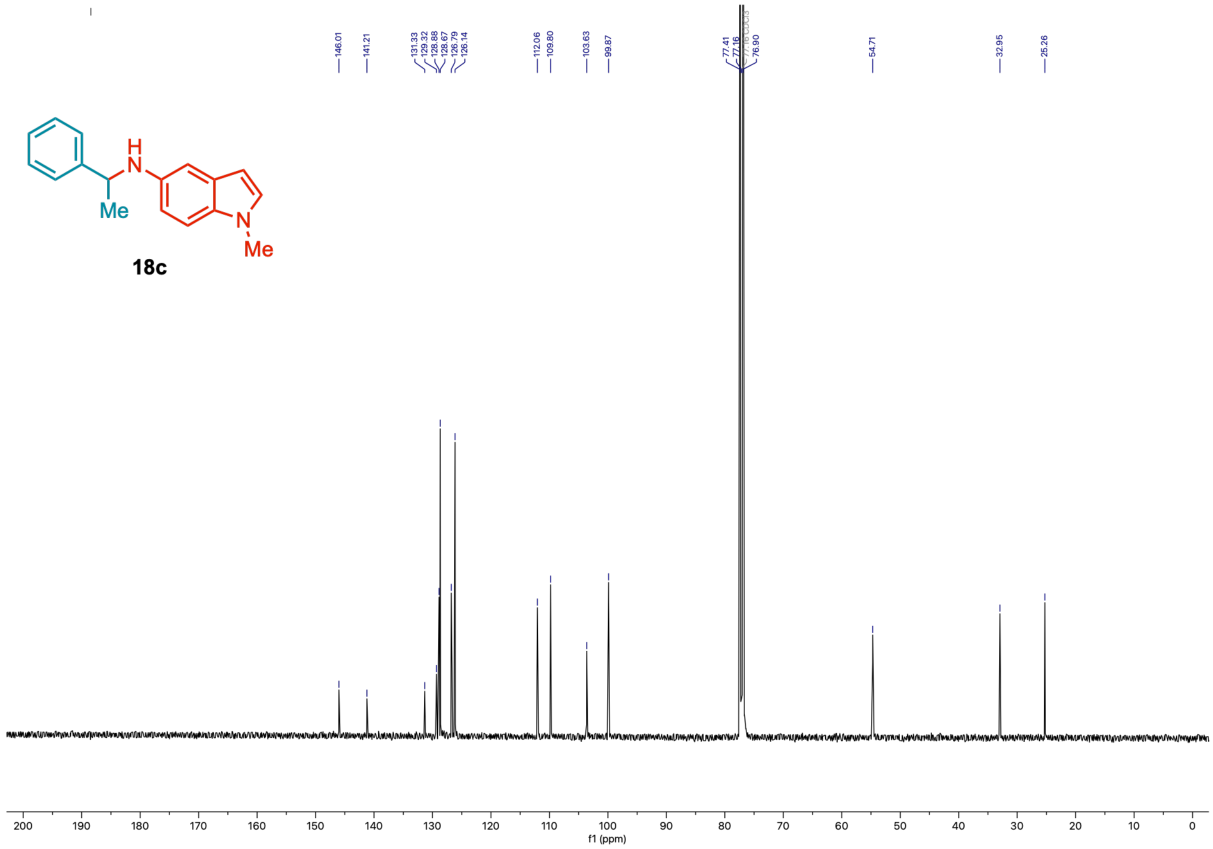

^{13}C NMR (126 MHz, $CDCl_3$) spectrum of **18c**

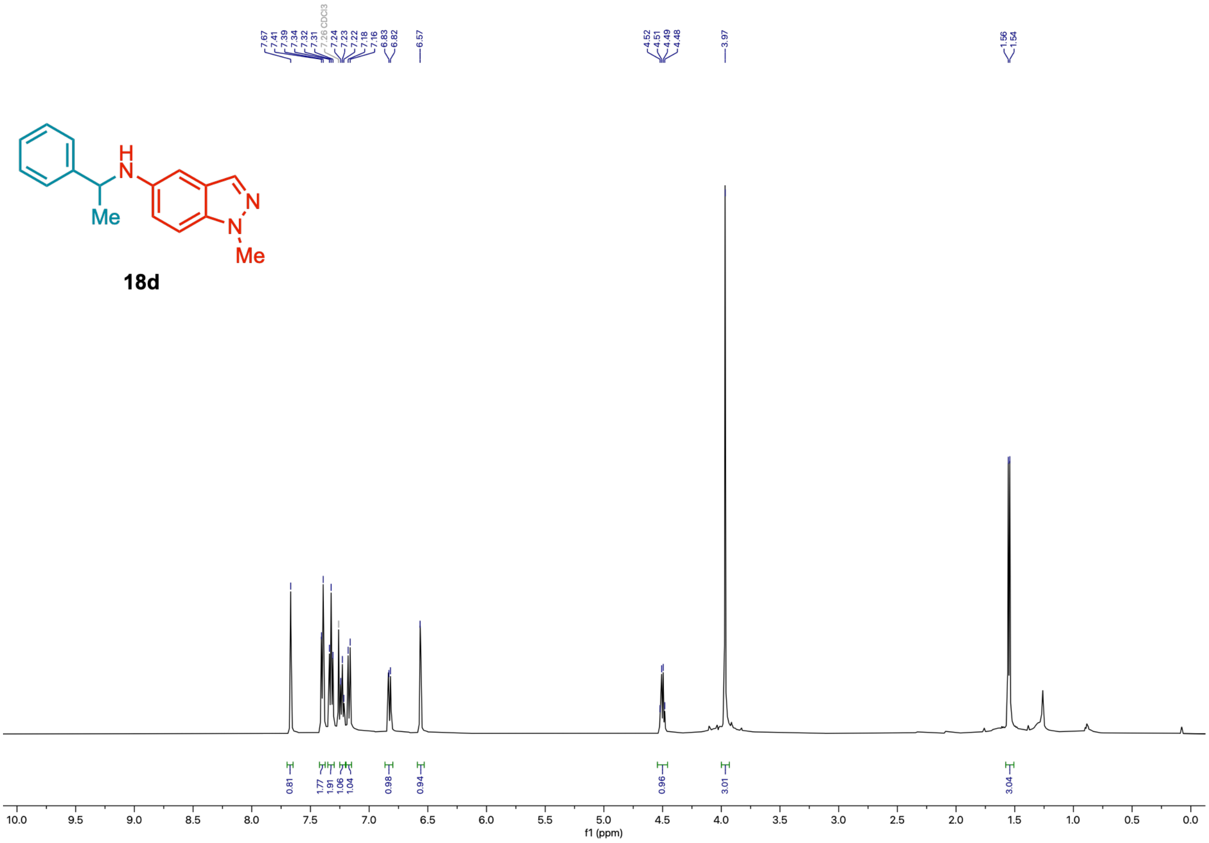

^{1}H NMR (500 MHz, $CDCl_3$) spectrum of **18d**

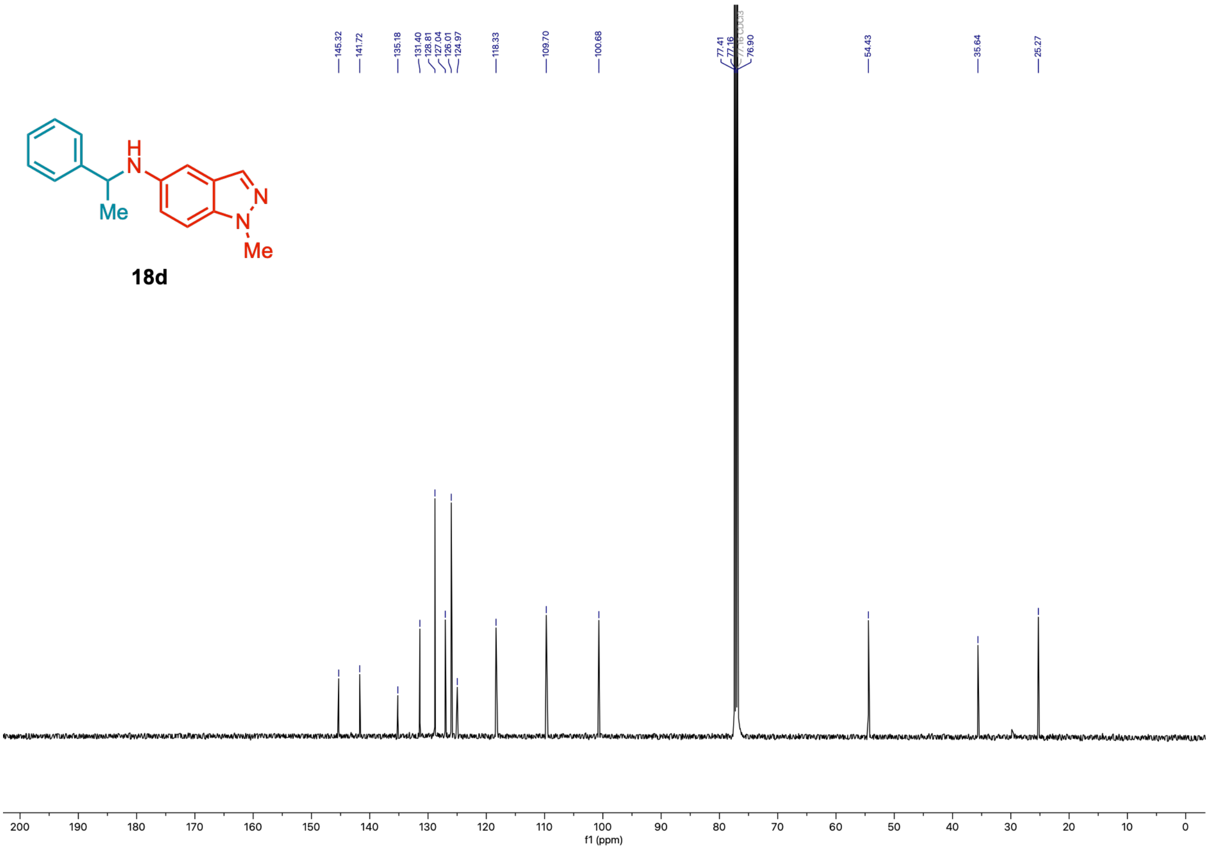

^{13}C NMR (126 MHz, $CDCl_3$) spectrum of **18d**

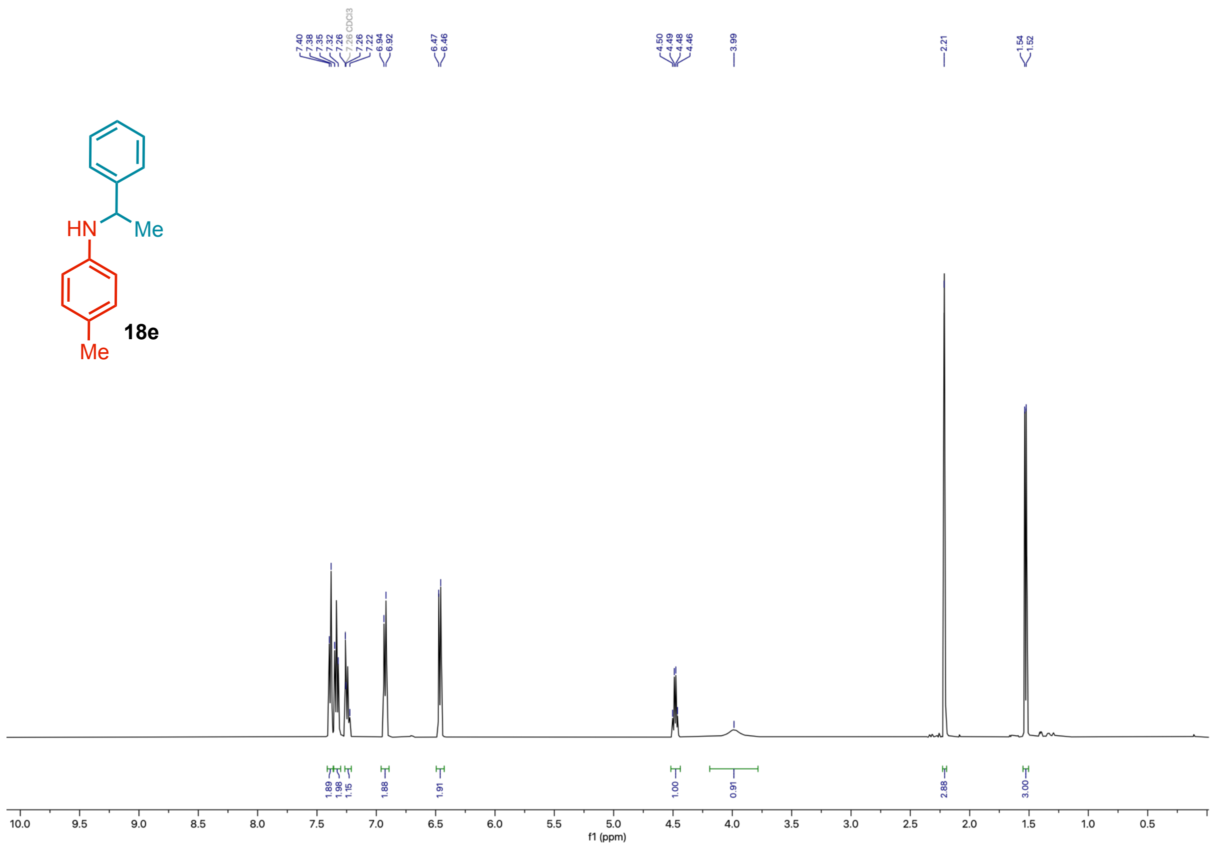

^{1}H NMR (500 MHz, $CDCl_3$) spectrum of **18e**

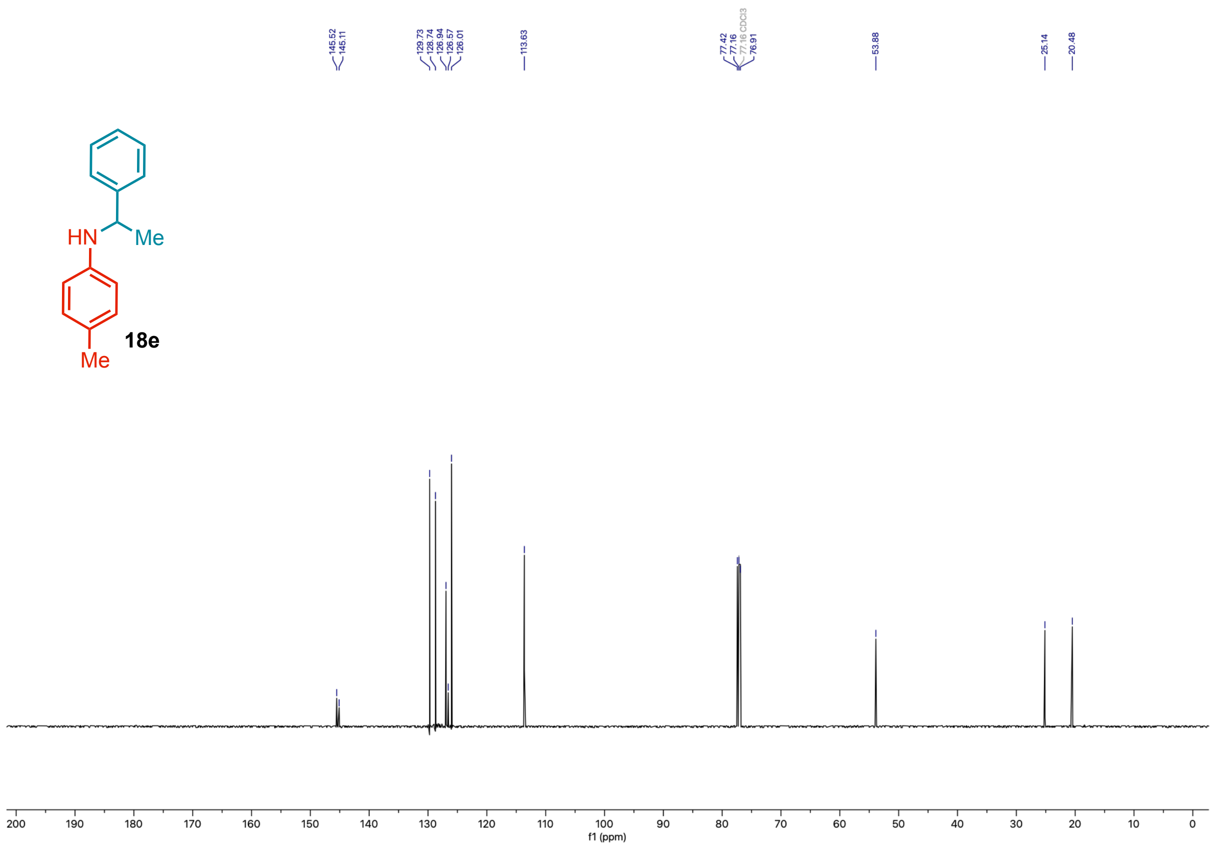

^{13}C NMR (126 MHz, $CDCl_3$) spectrum of **18e**

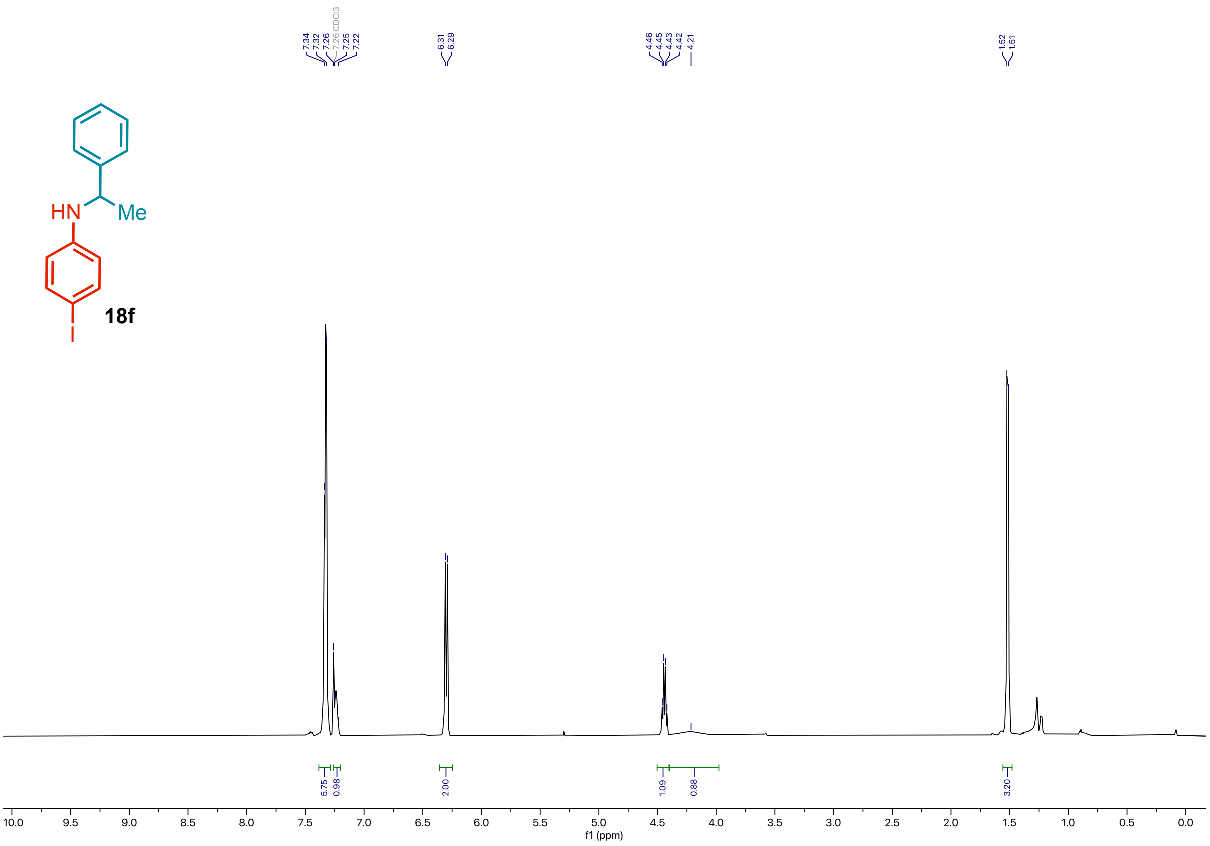

^{1}H NMR (500 MHz, $CDCl_3$) spectrum of **18f**

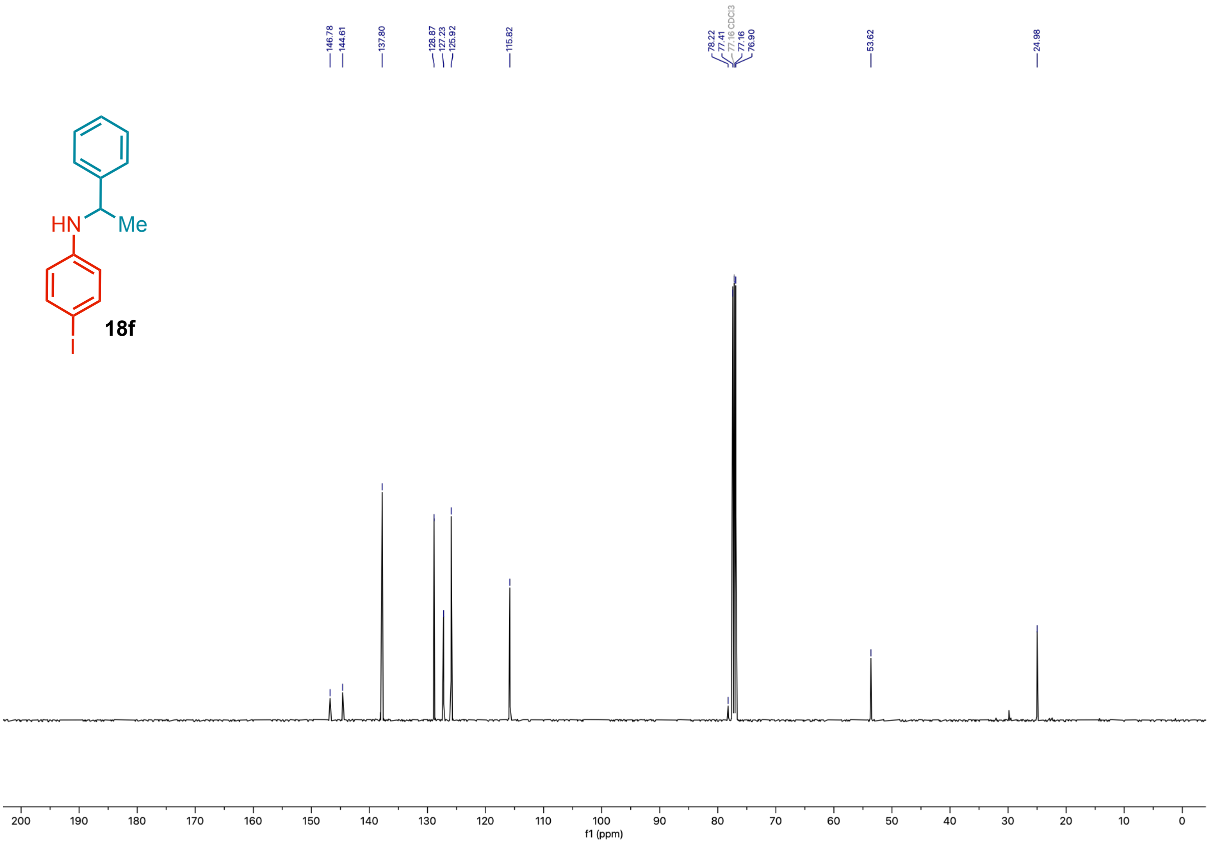

^{13}C NMR (126 MHz, $CDCl_3$) spectrum of **18f**

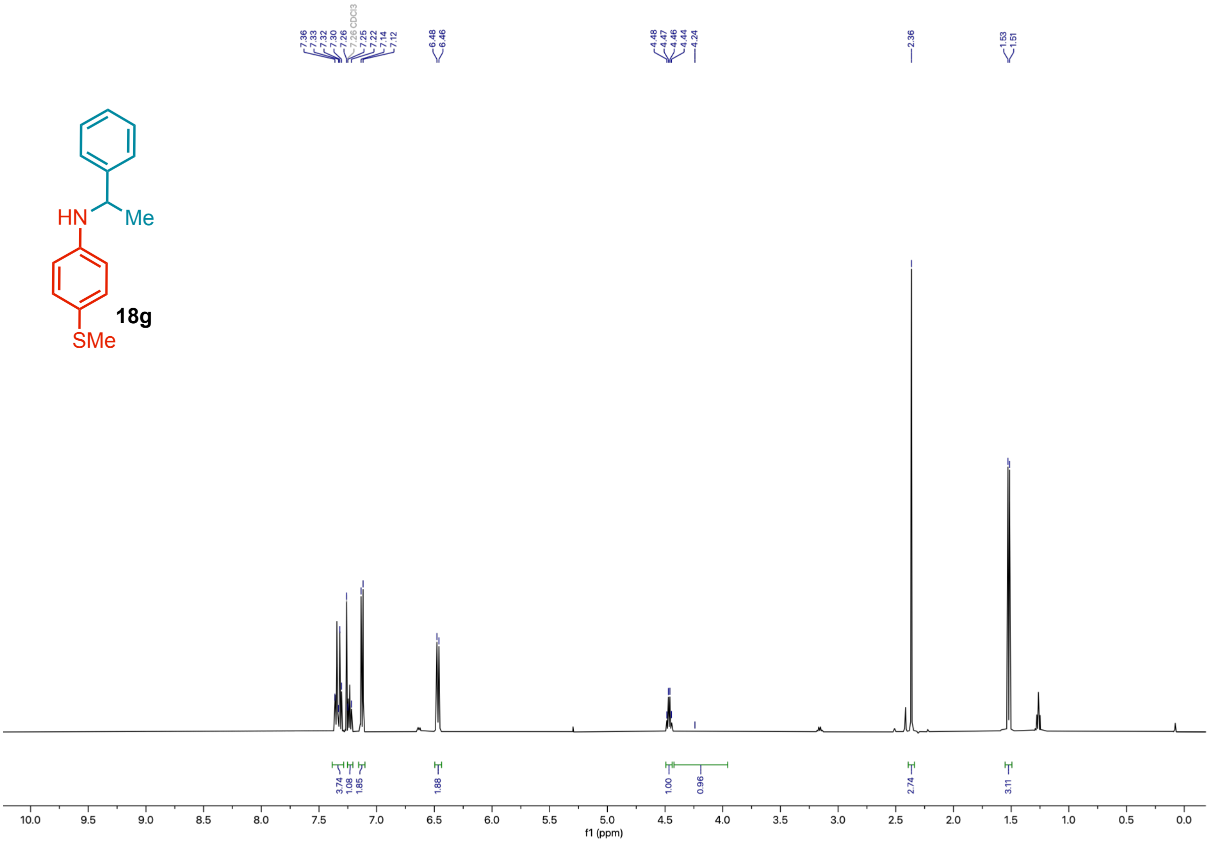

^{1}H NMR (500 MHz, CDCl$_3$) spectrum of **18g**

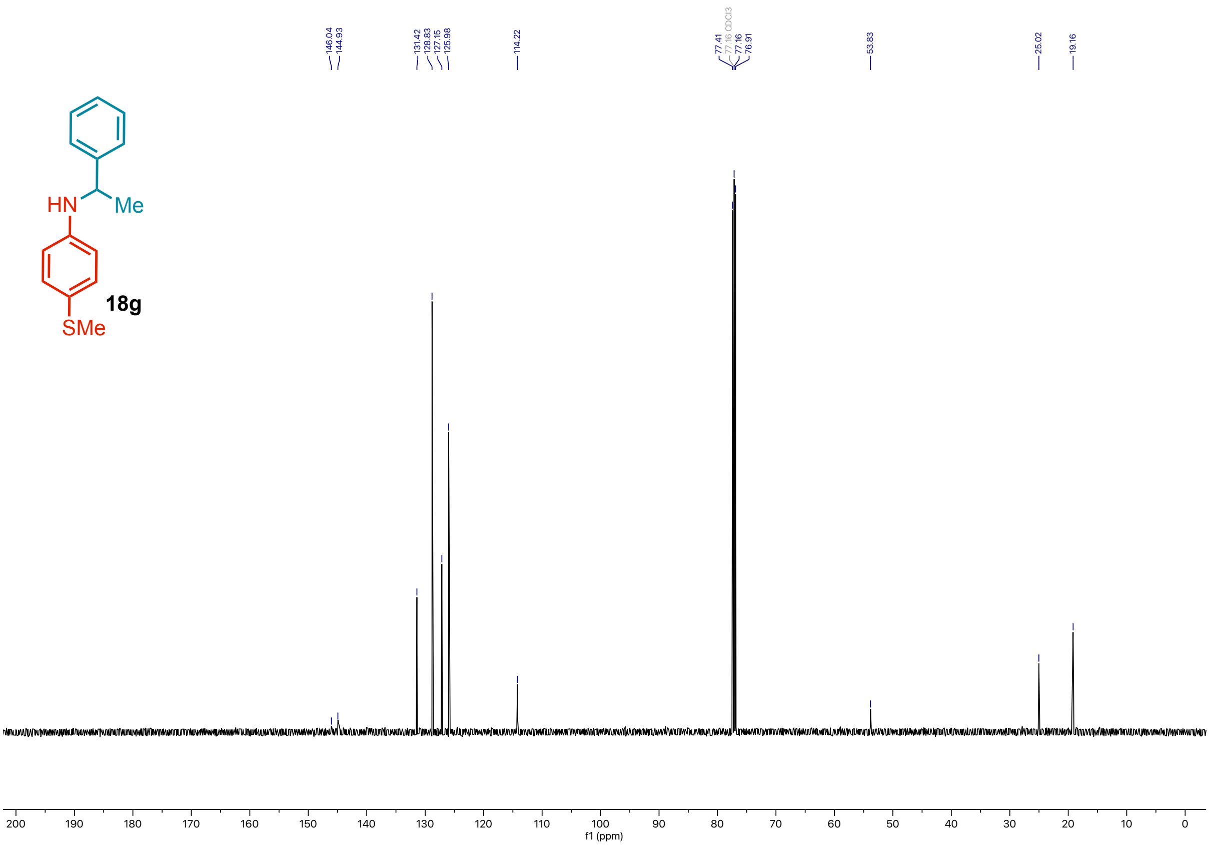

^{13}C NMR (126 MHz, CDCl$_3$) spectrum of **18g**

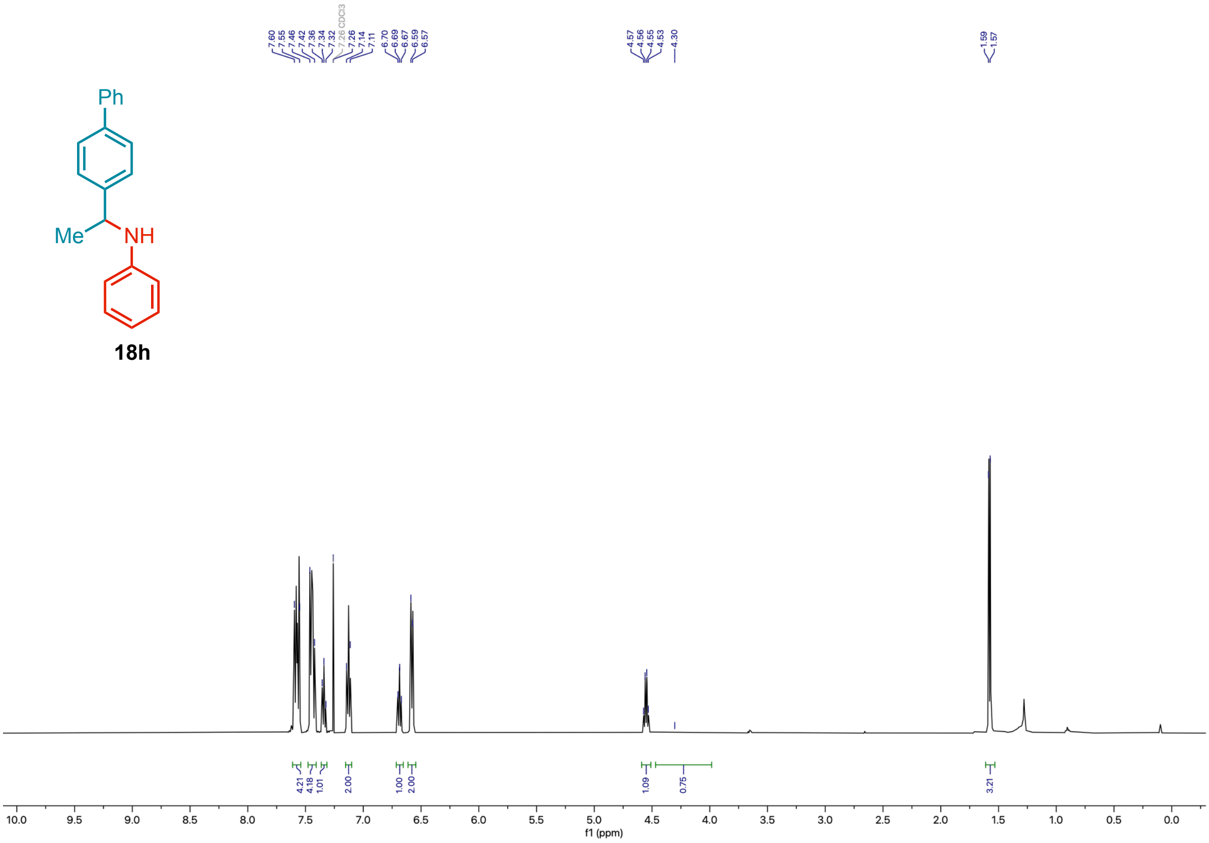

^{1}H NMR (500 MHz, $CDCl_3$) spectrum of **18h**

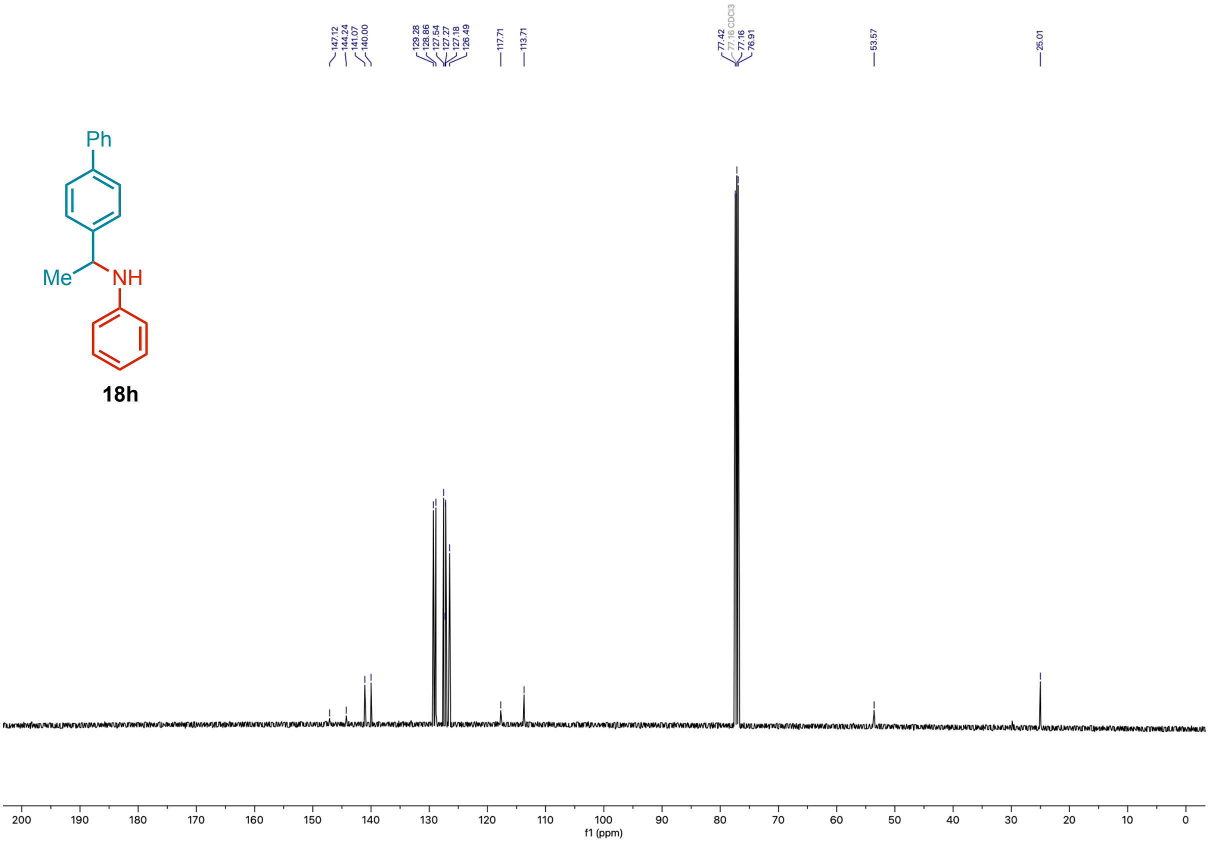

^{13}C NMR (126 MHz, $CDCl_3$) spectrum of **18h**

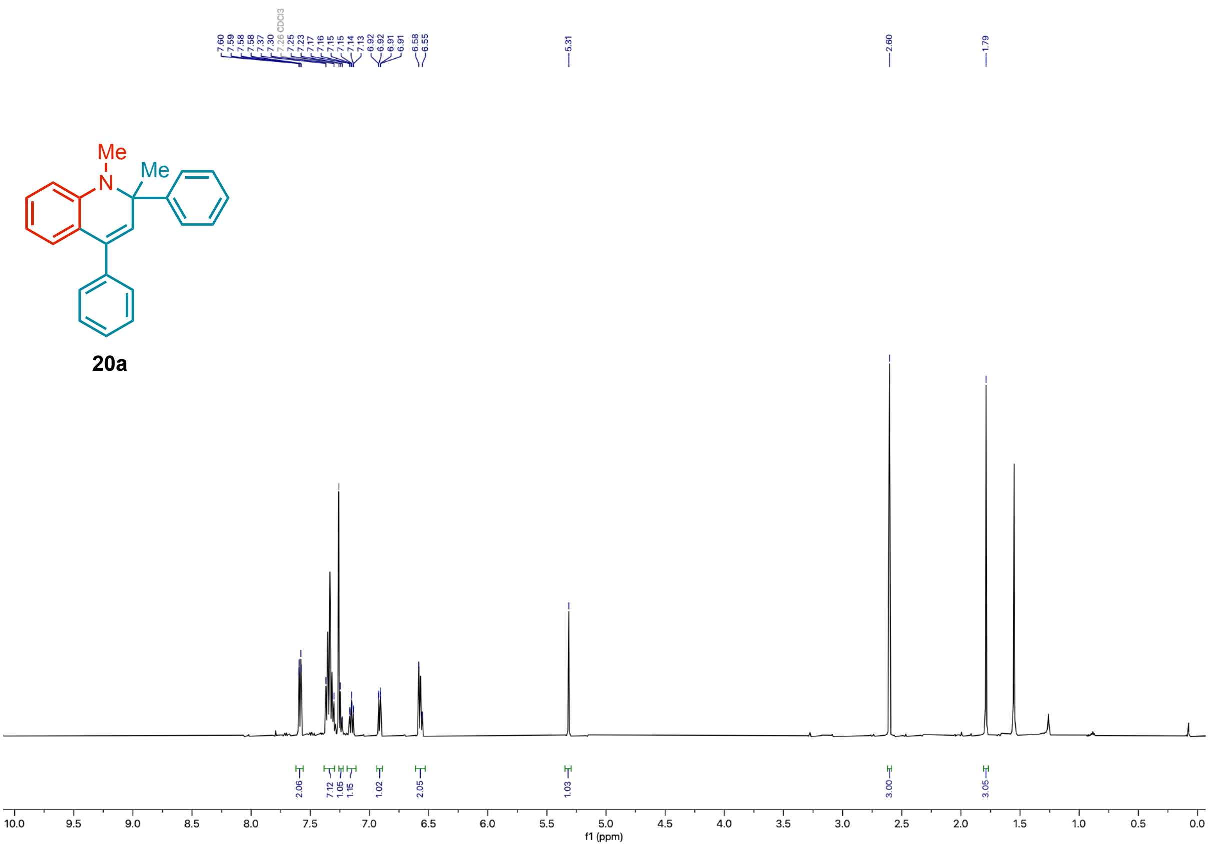

^{1}H NMR (500 MHz, CDCl$_3$) spectrum of **20a**

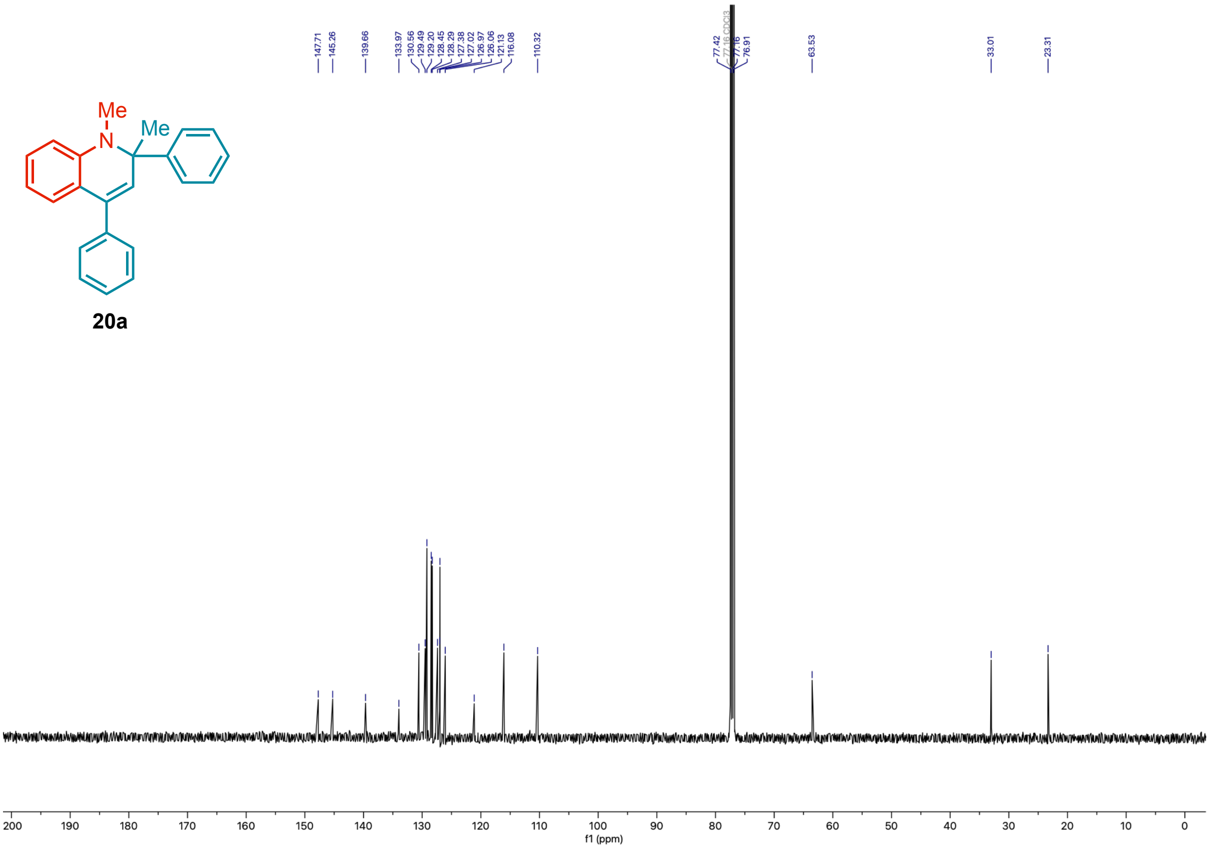

^{13}C NMR (126 MHz, CDCl$_3$) spectrum of **20a**

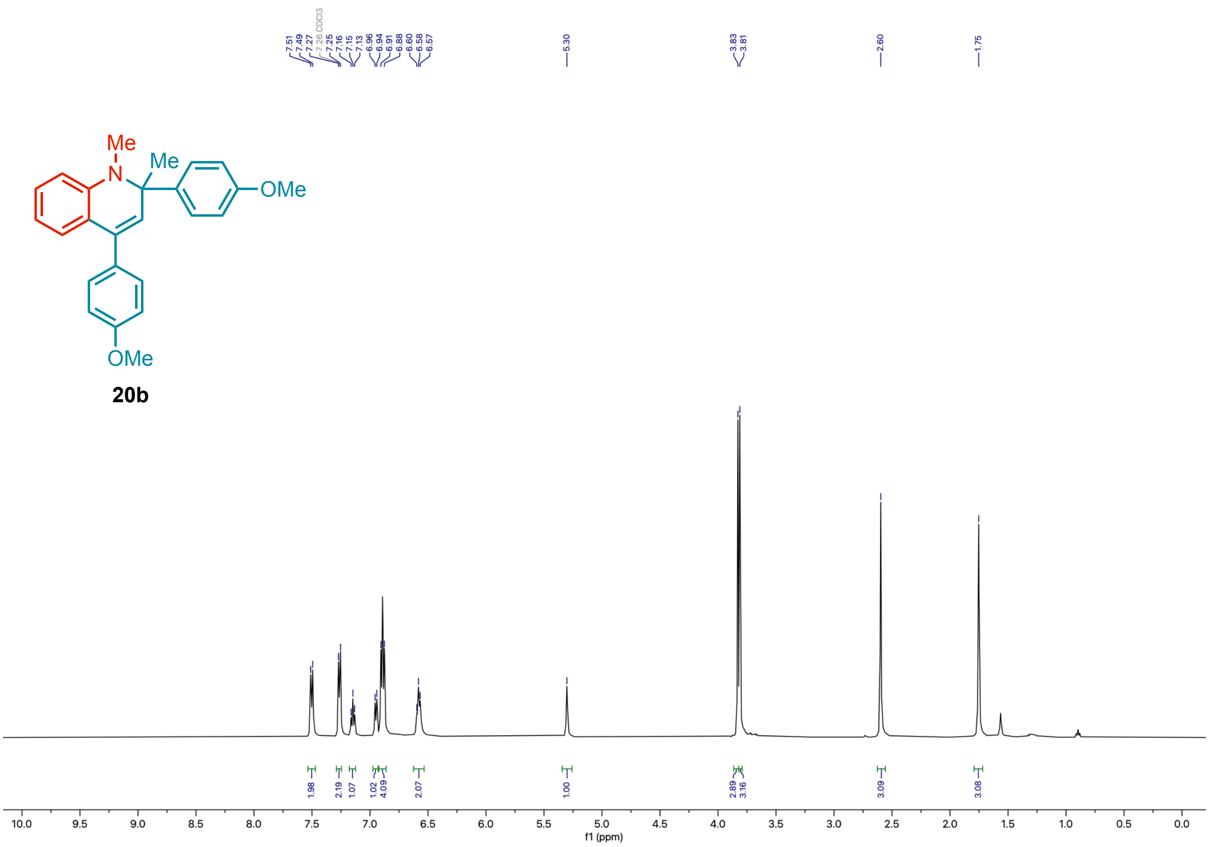

^{1}H NMR (500 MHz, $CDCl_3$) spectrum of **20b**

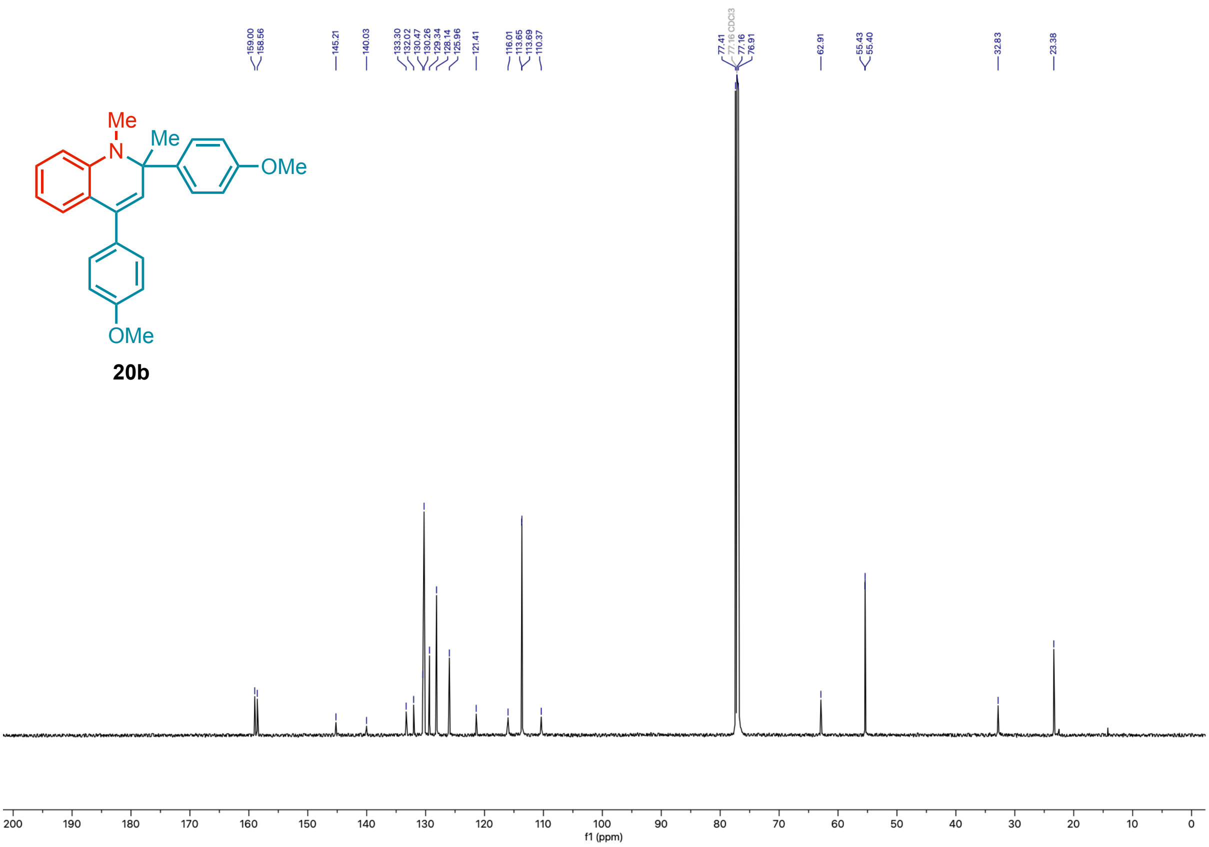

^{13}C NMR (126 MHz, $CDCl_3$) spectrum of **20b**

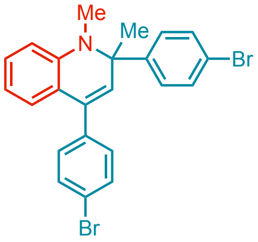

20c

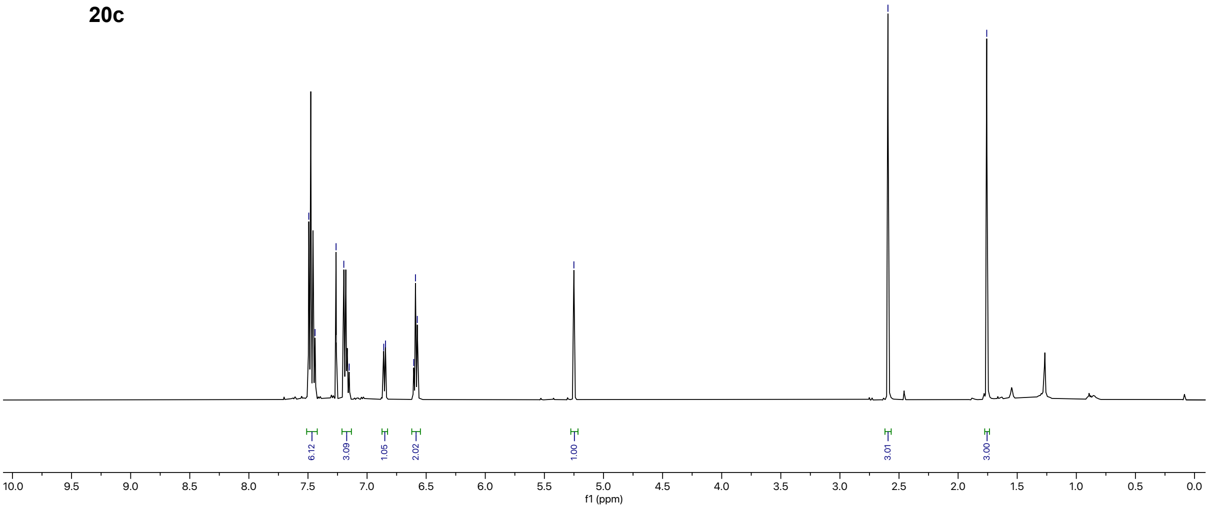

^{1}H NMR (500 MHz, $CDCl_3$) spectrum of **20b**

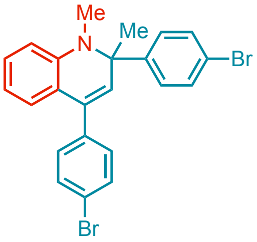

20c

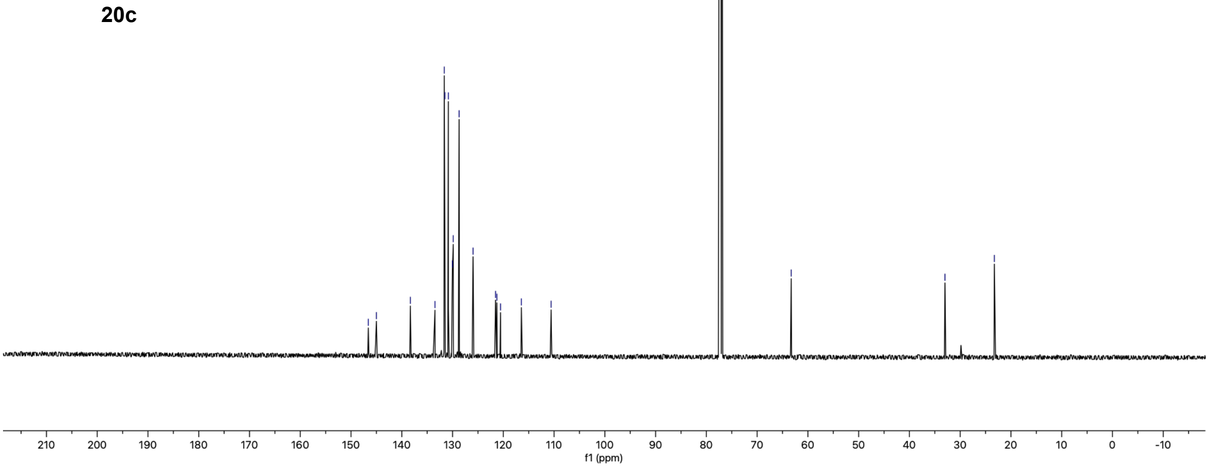

^{13}C NMR (126 MHz, $CDCl_3$) spectrum of **20c**

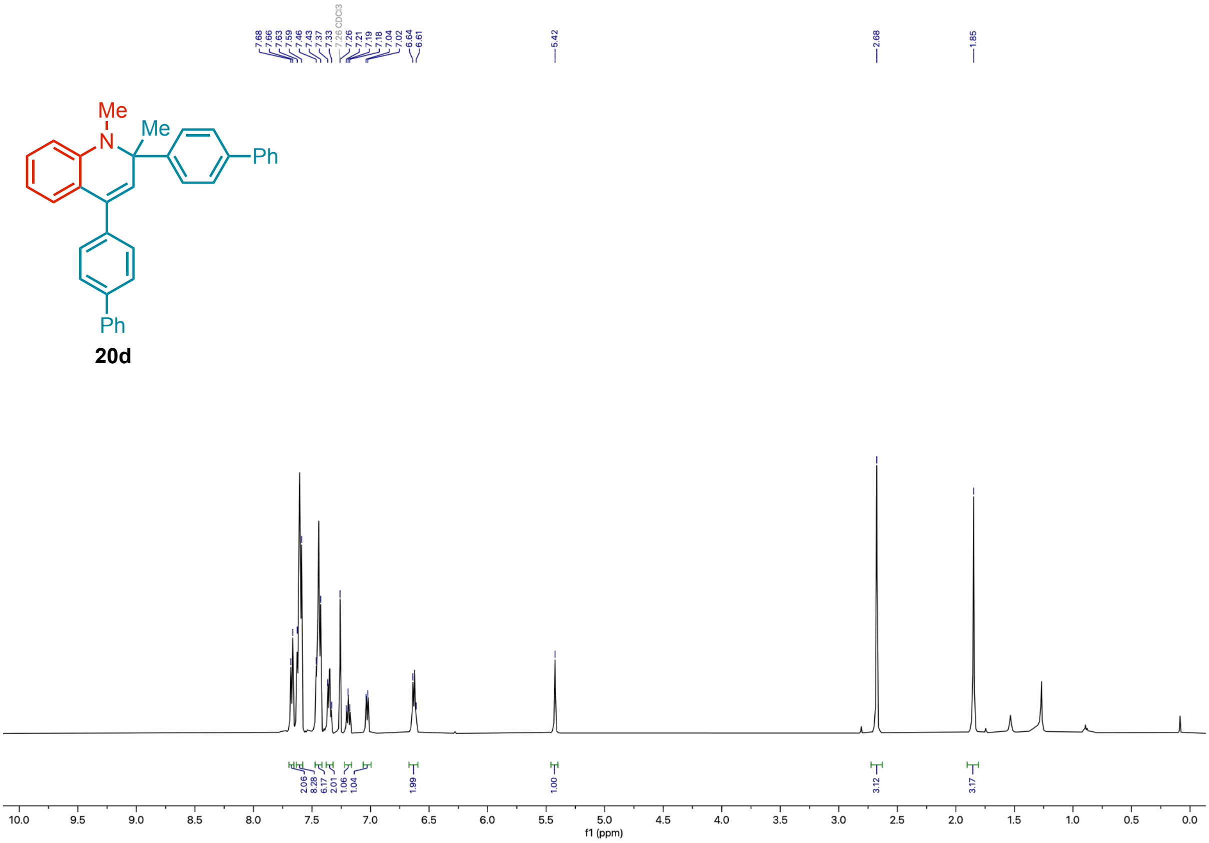

^{1}H NMR (500 MHz, $CDCl_3$) spectrum of **20d**

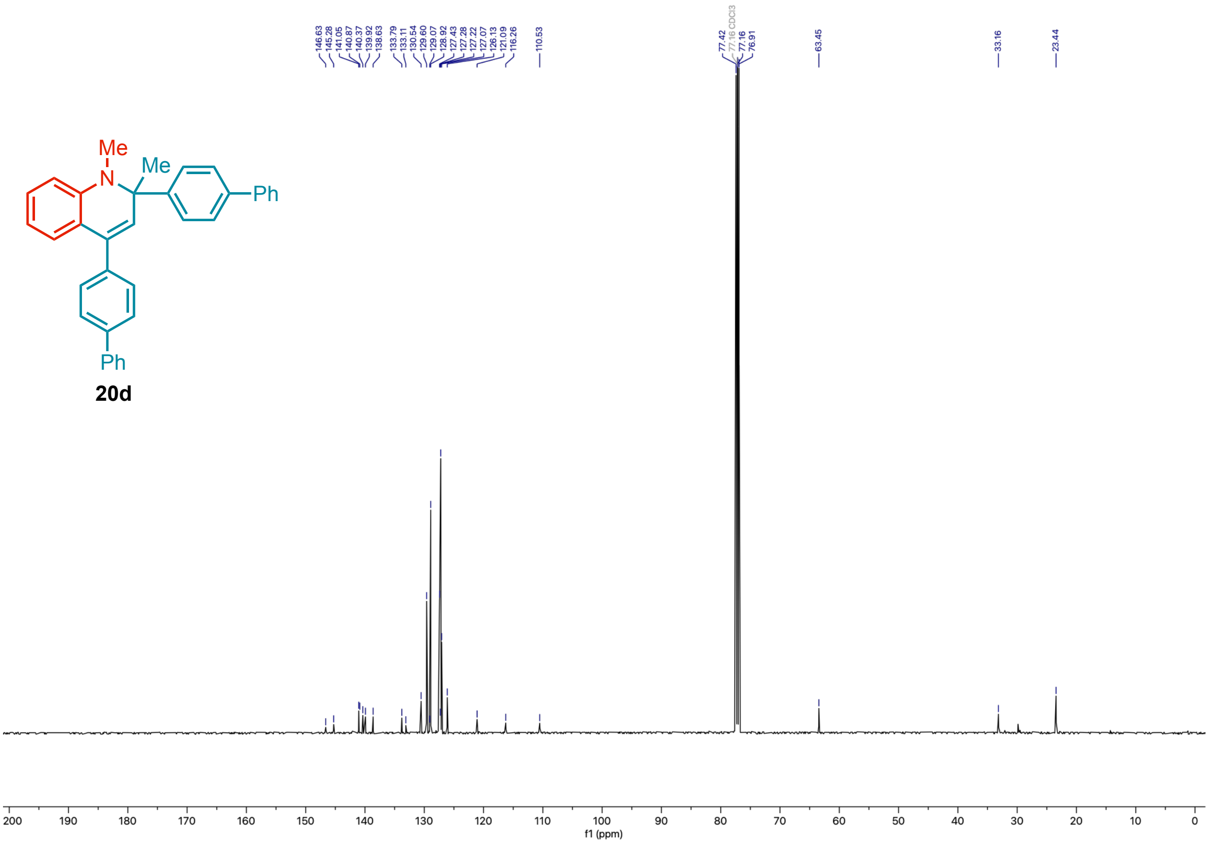

^{13}C NMR (126 MHz, $CDCl_3$) spectrum of **20d**

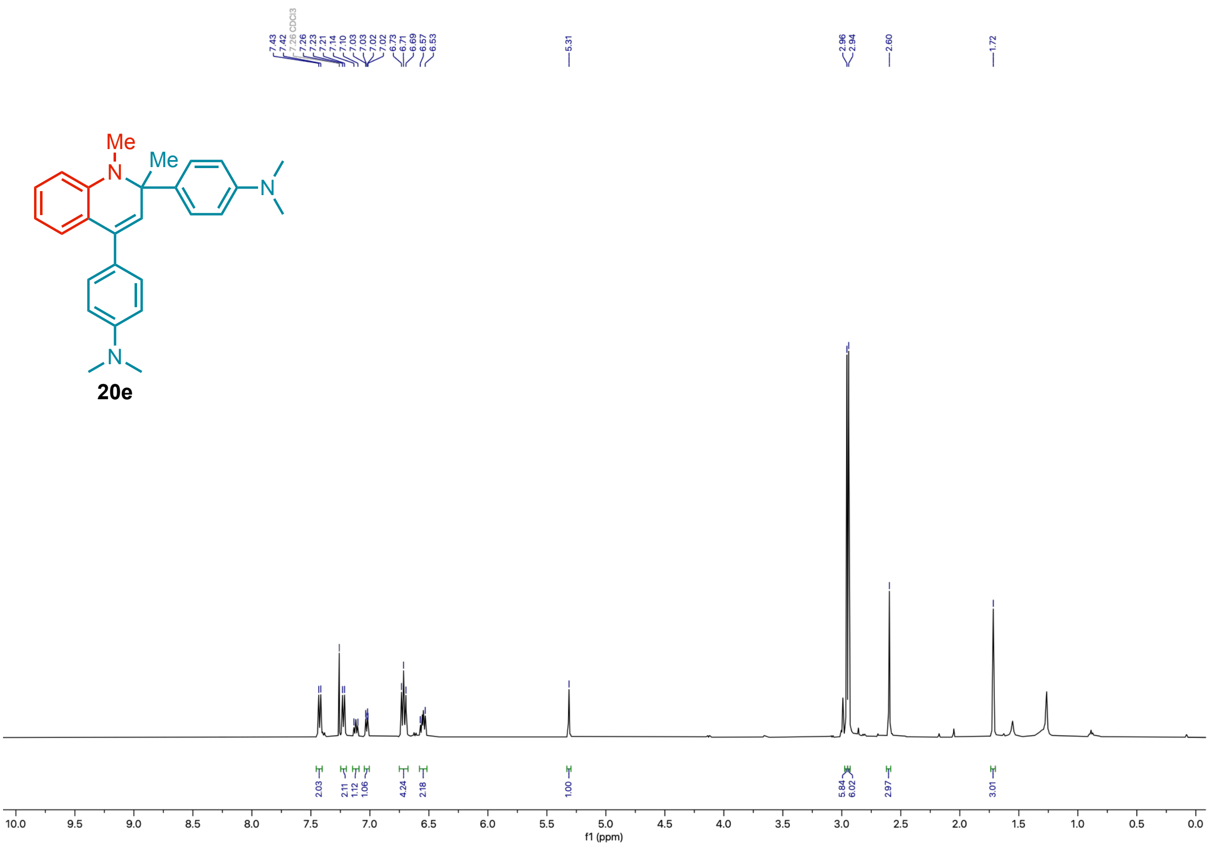

^{1}H NMR (500 MHz, $CDCl_3$) spectrum of **20e**

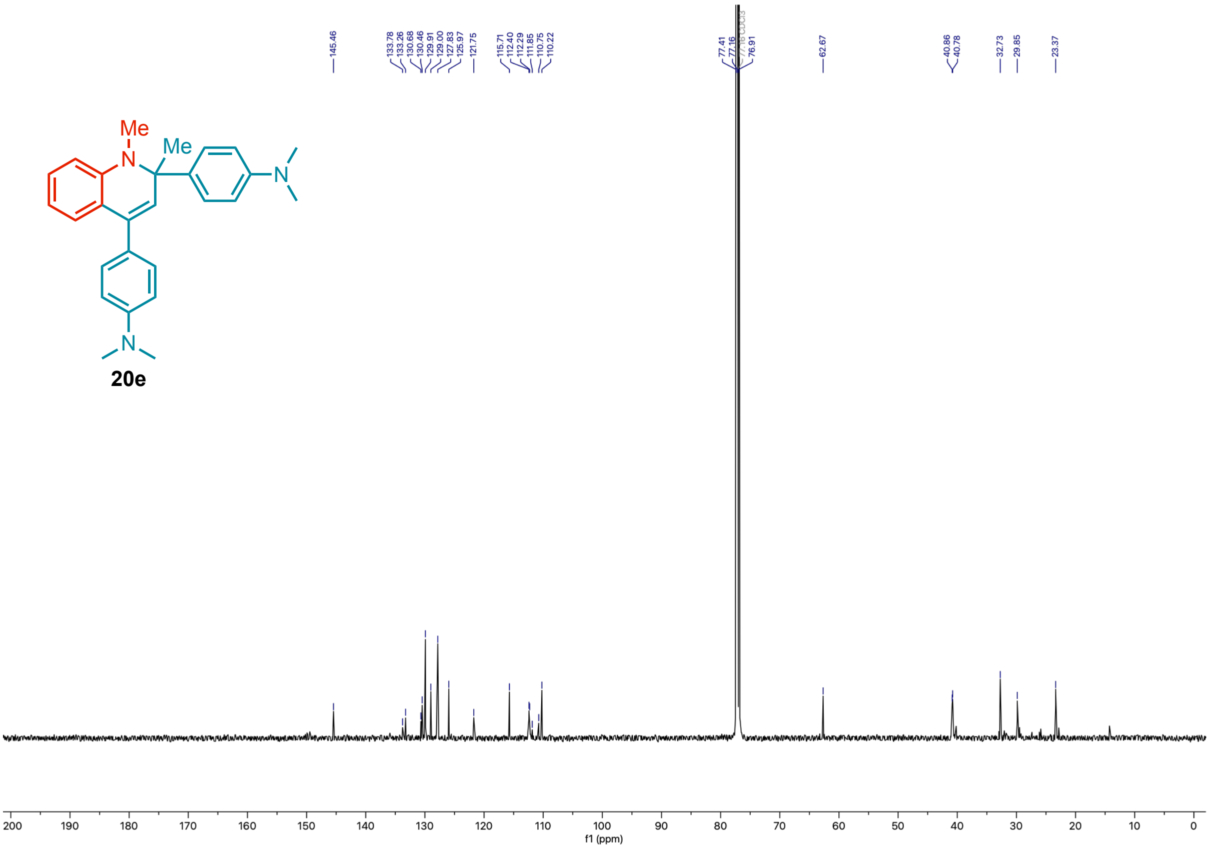

^{13}C NMR (126 MHz, $CDCl_3$) spectrum of **20e**

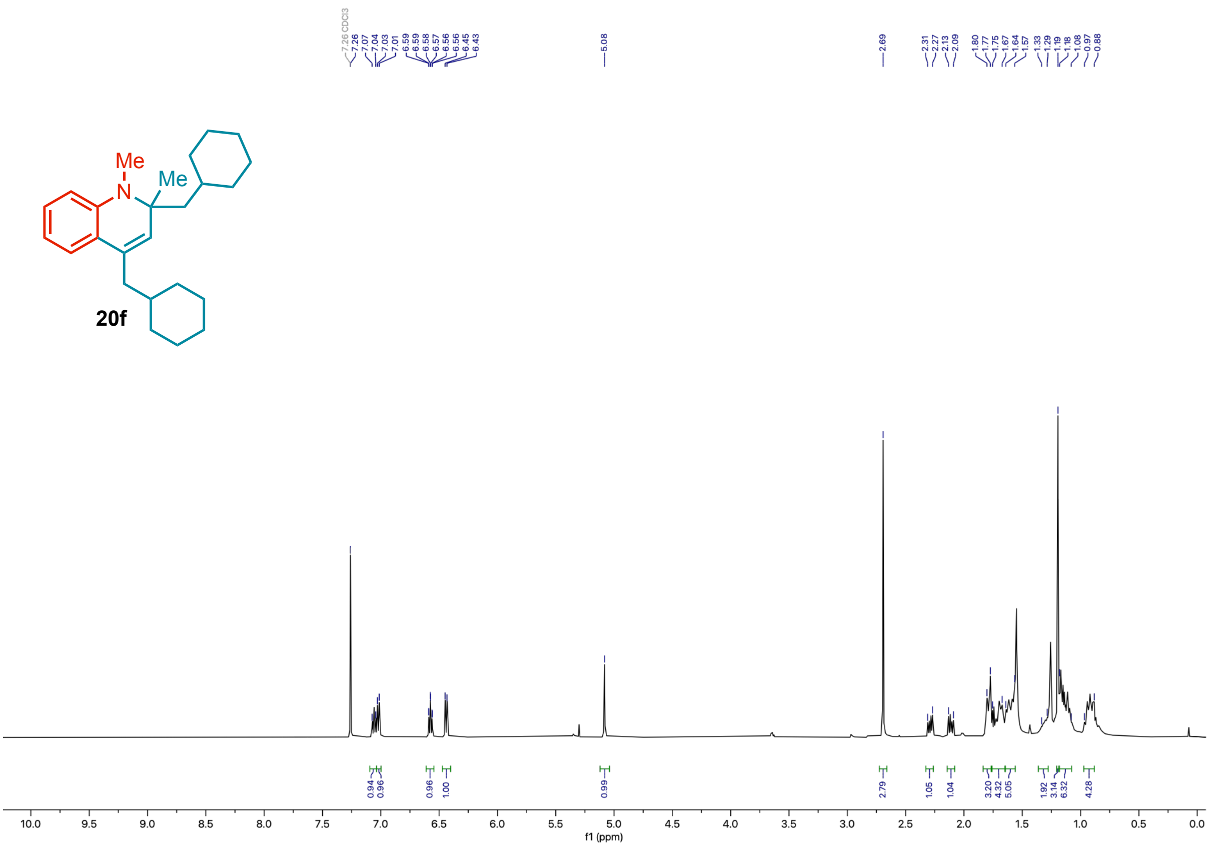

^{1}H NMR (500 MHz, $CDCl_3$) spectrum of **20f**

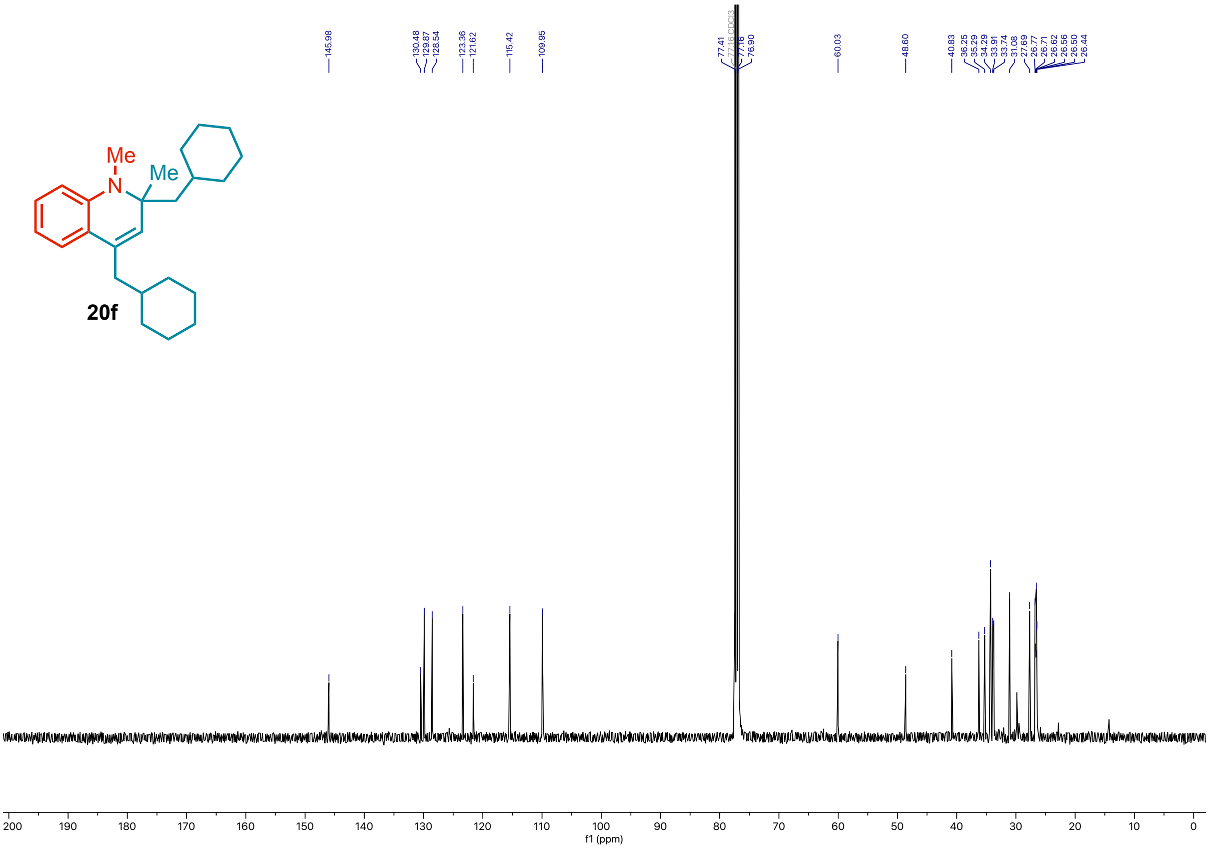

^{13}C NMR (126 MHz, $CDCl_3$) spectrum of **20f**

8. Cartesian Coordinates with Zero-Point Energies and Thermal Corrections

9a-carbene

Energy: -2342.923173 au

Sum of electronic and thermal Energies: -2342.174482 au

Geometry:

S	2.59260600	-1.94086100	-1.43601600
N	-0.30056400	0.30611300	-0.63264800
C	-3.83610200	2.24540900	0.44138200
C	-1.58059800	0.80983900	-0.32211300
C	0.18333900	-0.83915300	-1.23374600
C	-2.56409800	2.81088100	0.60143600
H	-2.45893700	3.80661500	1.01809700
C	-2.82168900	0.18091800	-0.42568700
C	0.68193600	1.21625800	-0.21940200
C	-1.44342800	2.08688100	0.23072700
C	0.31640800	3.74430400	-0.43031900
C	-2.89721000	-1.29808900	-0.78622300
H	-2.16279700	-1.46228500	-1.58107000
C	-3.93712900	0.93831100	-0.05070900
H	-4.91858800	0.48476400	-0.13538000
C	-5.07688600	3.03475800	0.78555600

H	-4.89424800	3.72570800	1.61283800
H	-5.41112200	3.63116200	-0.07077100
C	0.04779900	2.46206300	0.37227000
C	-2.41847100	-2.15907200	0.38368800
C	-4.26462200	-1.70536700	-1.32269400
C	0.50774600	2.56367300	1.83893600
C	1.56773900	-0.58274900	-1.14051600
C	1.89869400	0.66772700	-0.48531400
H	2.86873000	1.09763800	-0.29691900
C	-1.51727600	-3.80202600	2.48052900
H	-1.16827400	-4.43612500	3.28845900
C	2.86334500	-2.77199900	0.17899400
C	-2.63291100	-1.79824400	1.71621900
H	-3.14255700	-0.86891300	1.94348200
C	-0.21850800	1.97229300	2.87623700
H	-1.16703700	1.49576200	2.66290200
C	1.73826100	3.16017500	2.14114500
H	2.31491600	3.62403000	1.34923700
C	-5.21697300	-2.37303100	-0.54916200
H	-4.97741400	-2.65372100	0.46946500

C	4.23450900	-1.23959400	-1.74287900
C	-1.73530500	-3.35061900	0.11714500
H	-1.53037000	-3.61938500	-0.91225900
C	0.05409500	4.99741300	0.13724200
H	-0.26239800	5.06070000	1.17182700
C	-4.59537400	-1.36679800	-2.64098500
H	-3.86393200	-0.84635500	-3.25106000
C	-1.29085800	-4.16707800	1.15260400
H	-0.75246000	-5.08070700	0.92515900
C	-6.78915700	-2.34445600	-2.38743200
H	-7.76164100	-2.59272800	-2.79794700
C	-2.18803700	-2.61341500	2.75882700
H	-2.36615500	-2.31615700	3.78693200
C	-6.47024200	-2.68970400	-1.07657700
H	-7.19557800	-3.20983400	-0.46009500
C	0.74105300	3.68988900	-1.76018800
H	0.94971000	2.72829200	-2.21315600
C	4.65523000	-1.18433200	-3.07029700
H	4.03018300	-1.58679600	-3.85902700
C	5.88410200	-0.59863500	-3.36950100

H	6.21975600	-0.55316900	-4.39886200
C	-5.84318500	-1.68122300	-3.17088400
H	-6.07776000	-1.41205200	-4.19513500
C	6.67745800	-0.07861900	-2.34834500
H	7.63313900	0.37522900	-2.58385900
C	2.22591900	3.17075000	3.44492300
H	3.17929100	3.64242200	3.65778800
C	3.14368300	-4.15043800	2.55993300
H	3.25476700	-4.68941200	3.49393300
C	0.63797600	6.10021900	-1.93174400
H	0.76419000	7.00857600	-2.51025200
C	0.26850900	1.98272900	4.18327500
H	-0.31288000	1.52061500	4.97382900
C	0.21275700	6.16467200	-0.60496500
H	0.00777700	7.12546700	-0.14530900
C	0.90058700	4.85813800	-2.50502500
H	1.23205700	4.79425200	-3.53578000
C	1.49119800	2.58259900	4.47479800
H	1.86777500	2.59409200	5.49156300
C	6.24497200	-0.14144500	-1.02329400

H	6.86090400	0.26453700	-0.22927900
C	5.01913300	-0.72371500	-0.71209700
H	4.67930800	-0.78052600	0.31419100
C	4.01765900	-4.40776900	1.50130800
H	4.80655200	-5.14312600	1.61078900
C	3.87715500	-3.72234100	0.29770100
H	4.55547700	-3.91539400	-0.52582500
C	1.97490700	-2.51485100	1.21509600
H	1.17213600	-1.80056500	1.08054200
C	2.12544200	-3.21152500	2.41414400
H	1.43156800	-3.02108000	3.22374700
H	-5.90314900	2.37777800	1.06789300

[9a-[Au]Cl]

Energy: -2939.086712 au

Sum of electronic and thermal Energies: -2938.330710 au

Geometry:

S	2.59083700	-1.95787800	0.65809000
N	-0.07944300	0.68772900	-0.05839500
C	-3.14660400	3.31525600	-1.21096300

C	-1.25950800	1.42029000	-0.39457600
C	0.29220300	-0.61292500	0.22635700
C	-1.79883900	3.67282600	-1.11734300
H	-1.47672800	4.67832800	-1.36396200
C	-2.61273900	1.05586400	-0.37662100
C	1.02233100	1.54798800	-0.05899700
C	-0.87416600	2.73379300	-0.69697000
C	1.28578500	3.42190000	-1.75937700
C	-3.09459300	-0.25564400	0.22558300
H	-2.43776300	-1.04190300	-0.16376800
C	-3.52073100	2.02733000	-0.81724500
H	-4.57147000	1.76572400	-0.83641100
C	-4.17489700	4.29416100	-1.72244100
H	-3.89788600	5.32462100	-1.48583400
H	-4.27048200	4.22187300	-2.81138200
C	0.62601900	2.95106700	-0.45125300
C	-2.91544900	-0.28247400	1.74394300
C	-4.50264400	-0.64971400	-0.19994900
C	0.91902800	3.88007700	0.74299900
C	1.66911500	-0.48957100	0.48550600

C	2.14054300	0.84895100	0.27488000
H	3.14139200	1.23159700	0.38340600
C	-2.60808900	-0.43947100	4.53236200
H	-2.48538400	-0.50064100	5.60815600
C	4.08042700	-1.46022800	1.56783000
C	-2.80315600	0.87631800	2.51418400
H	-2.82939700	1.84672900	2.03265300
C	-0.05634200	4.18807400	1.69406800
H	-1.06943700	3.83121600	1.56105400
C	2.22417800	4.34530500	0.94867200
H	2.99309600	4.11931500	0.21878100
C	-5.63385000	-0.26343200	0.52519600
H	-5.51230800	0.30736700	1.43896600
C	3.20910500	-2.35588200	-0.99895400
C	-2.87691600	-1.52428400	2.39028900
H	-2.96092100	-2.43199700	1.80262800
C	1.32650200	4.78412900	-2.07821500
H	0.96853600	5.51362600	-1.36146500
C	-4.67374000	-1.41508900	-1.35743300
H	-3.80082300	-1.74779100	-1.90942200

C	-2.72301300	-1.60365900	3.77023900
H	-2.68708600	-2.57517800	4.25073800
C	-7.07204700	-1.36951500	-1.07149200
H	-8.06443600	-1.65134600	-1.40559500
C	-2.65027400	0.80003600	3.89982600
H	-2.56210700	1.71157200	4.48131800
C	-6.91056900	-0.61629900	0.09066200
H	-7.77834600	-0.31001600	0.66481900
C	1.76423100	2.50192200	-2.69714600
H	1.73473000	1.44274400	-2.47206900
C	2.69343100	-3.51477000	-1.57410200
H	2.00407700	-4.14794300	-1.02738100
C	3.03836500	-3.81436200	-2.89233100
H	2.63448600	-4.70570200	-3.35677700
C	-5.94819100	-1.77281100	-1.79183300
H	-6.06169300	-2.37640700	-2.68549400
C	3.87741500	-2.96315700	-3.60686300
H	4.13778200	-3.19840300	-4.63242100
C	2.54323400	5.10373000	2.07092000
H	3.55868700	5.45930700	2.20803300

C	6.28372500	-0.84578900	3.13515900
H	7.14634600	-0.60106500	3.74373000
C	2.31629000	4.29111500	-4.22709200
H	2.71531300	4.62639500	-5.17776300
C	0.26204900	4.94817700	2.81993800
H	-0.51080800	5.17778000	3.54518600
C	1.83748900	5.21572500	-3.30009300
H	1.86367100	6.27610700	-3.52589200
C	2.27561600	2.93260900	-3.92027000
H	2.63945200	2.20221300	-4.63470600
C	1.56089600	5.41065100	3.01317000
H	1.80678800	6.00466300	3.88616600
C	4.37446300	-1.79822300	-3.01547600
H	5.01756700	-1.13207800	-3.57878300
C	4.03614700	-1.48229600	-1.70446400
H	4.40103500	-0.57389200	-1.24181000
C	6.40424700	-1.76282300	2.09238800
H	7.35784700	-2.23510200	1.88773600
C	5.30063300	-2.08088600	1.30355700
H	5.39665800	-2.79088100	0.49214000

C	3.93936200	-0.55294500	2.61791900
H	2.98309200	-0.08557700	2.81529800
C	5.05298900	-0.24568000	3.39639500
H	4.95284000	0.46618700	4.20716500
H	-5.16070900	4.09834300	-1.29440600
Au	-0.56558400	-2.39692200	-0.19176500
Cl	-1.32520800	-4.51195100	-0.79277100

9b-carbene

Energy: -2151.131428 au

Sum of electronic and thermal Energies: -2150.438585 au

Geometry:

S	-2.68810100	-2.26435800	0.34210400
N	0.53067500	-0.33607900	-0.05395700
C	4.27941600	1.40408800	-0.68255900
C	1.86492100	0.07443400	-0.28486600
C	-0.11911500	-1.55006700	0.04137700
C	3.10520400	2.14110400	-0.47619900
H	3.13671400	3.22521600	-0.46133900
C	3.00995900	-0.69695700	-0.48595800

C	-0.28848200	0.78840100	0.12234700
C	1.90813300	1.47524200	-0.28473300
C	0.55609200	2.93423700	1.23582800
C	2.95199700	-2.21993500	-0.54787900
H	2.11690800	-2.52965700	0.08810800
C	4.20497000	0.00854200	-0.68525100
H	5.11616100	-0.56180000	-0.83167000
C	5.60439100	2.10861500	-0.85577000
H	5.49512200	3.02573700	-1.44125200
H	6.02872300	2.38971500	0.11457000
C	0.50662900	2.07000900	-0.03410500
C	4.21657000	-2.85623000	0.00850600
C	-0.06714500	2.82894200	-1.24499400
C	-1.43018300	-1.09073900	0.30063000
C	-1.55807400	0.35303500	0.34197800
H	-2.43764900	0.95059400	0.51928400
C	-3.69648200	-2.06857700	-1.17219500
C	0.47580000	2.69474700	-2.52484500
H	1.36948400	2.10040800	-2.66803600
C	-1.22717100	3.59907200	-1.08884900

H	-1.65867400	3.71708000	-0.10139800
C	5.18471200	-3.46771500	-0.79208000
H	5.04442100	-3.52145400	-1.86484600
C	-3.86250700	-1.66988800	1.59565800
C	0.96124500	4.27249600	1.16320300
H	1.16492500	4.71859900	0.19675200
C	4.43776600	-2.81226100	1.39231400
H	3.69624900	-2.33680900	2.02659200
C	6.54690000	-3.96244200	1.14496600
H	7.44307300	-4.38901400	1.58179900
C	6.34040800	-4.01618200	-0.23037800
H	7.07794600	-4.48621800	-0.87231500
C	0.28254200	2.38709700	2.49204300
H	-0.03450000	1.35372900	2.56402000
C	-3.72956100	-2.21296200	2.87244400
H	-2.98979800	-2.98145000	3.06527700
C	-4.55030700	-1.74680200	3.89728700
H	-4.45523400	-2.16569800	4.89222800
C	5.58593800	-3.35696100	1.95738300
H	5.73272500	-3.31198000	3.03126300

C	-5.49019800	-0.74919600	3.64203500
H	-6.12711400	-0.38851700	4.44137200
C	-1.82574300	4.21932100	-2.18097000
H	-2.72071000	4.81513600	-2.03693500
C	-5.18008200	-1.88321400	-3.50154900
H	-5.76168400	-1.80937200	-4.41323100
C	0.81354700	4.48826400	3.56531400
H	0.91062300	5.08788500	4.46350800
C	-0.12305800	3.31662600	-3.62170300
H	0.31712900	3.20248500	-4.60645300
C	1.08946800	5.04309900	2.31597400
H	1.40168300	6.07885000	2.23751900
C	0.41000200	3.15734100	3.64738500
H	0.19185500	2.71395100	4.61293300
C	-1.27461900	4.08148500	-3.45596800
H	-1.73668500	4.56858700	-4.30746700
C	-5.61256800	-0.21437500	2.35977000
H	-6.34160300	0.56262200	2.16090600
C	-4.79861600	-0.67329600	1.32675000
H	-4.89505800	-0.26594100	0.32837600

C	-5.58062800	-2.75876300	-2.49032800
H	-6.46990400	-3.36580800	-2.61486200
C	-4.83588800	-2.85930100	-1.31865300
H	-5.14476200	-3.53508200	-0.52880400
C	-3.27555900	-1.20250000	-2.17425100
H	-2.37701300	-0.61292100	-2.03889300
C	-4.03125200	-1.11100600	-3.34384400
H	-3.71582100	-0.43376500	-4.12893300
H	6.33347500	1.47002800	-1.35975600
C	2.58043800	-2.66393800	-1.97347300
H	3.29322000	-2.28941900	-2.71396700
H	2.54178000	-3.75464500	-2.04247700
H	1.59000800	-2.27613200	-2.21859700

[9b-[Au]Cl]

Energy: -2747.293574 au

Sum of electronic and thermal Energies: -2746.593364 au

Geometry:

S	-2.55704000	-1.87173300	-0.65909800
N	0.28417800	0.67218400	-0.28779300
C	3.56686400	3.22376200	0.35443300

C	1.52759200	1.37066700	-0.14085100
C	-0.17249800	-0.60474800	-0.56434800
C	2.22996300	3.61293100	0.47471500
H	1.97443200	4.62566900	0.76579200
C	2.85470400	0.95942800	-0.32684100
C	-0.77616600	1.56023000	-0.08507600
C	1.22918300	2.69519400	0.21547100
C	-0.67213400	3.37922200	1.68535100
C	3.23562700	-0.40105800	-0.89558100
H	2.56729300	-1.13906100	-0.44007500
C	3.84223000	1.91763300	-0.05440300
H	4.87772000	1.62023000	-0.16801800
C	4.68444400	4.18540100	0.67729700
H	4.41512300	5.21222800	0.41653600
H	4.91314500	4.16877700	1.74854500
C	-0.28446400	2.94402000	0.25969300
C	4.64191800	-0.83872100	-0.52281000
C	-0.78608200	3.91856800	-0.82196400
C	-1.56964700	-0.43499900	-0.58427200
C	-1.95628100	0.90399700	-0.25130600

H	-2.94792700	1.31842700	-0.18292700
C	-4.17436600	-1.28298100	-1.23726500
C	-0.00058100	4.27054200	-1.92165800
H	1.01791000	3.91039000	-1.99281000
C	-2.10316000	4.39226400	-0.76126000
H	-2.72465500	4.13419200	0.08861000
C	5.74455100	-0.65228800	-1.36169100
H	5.61386400	-0.19360100	-2.33461300
C	-2.86464900	-2.35531600	1.06061700
C	-0.62726800	4.73019200	2.04797100
H	-0.39941900	5.47942100	1.29912400
C	4.84914200	-1.44832900	0.72026300
H	3.99896300	-1.62053600	1.37222300
C	7.21473400	-1.64898700	0.27873400
H	8.20556700	-1.96515900	0.58540000
C	7.02144900	-1.05198200	-0.96471200
H	7.86347900	-0.90040800	-1.63152100
C	-0.98012600	2.43370500	2.66799400
H	-1.01410100	1.38247400	2.40847600
C	-2.31964600	-3.57489100	1.45448000

H	-1.78227300	-4.19931900	0.74990900
C	-2.42532000	-3.94857500	2.79458700
H	-1.99305700	-4.88750700	3.11830100
C	6.12028500	-1.84962100	1.12038300
H	6.25526500	-2.32908900	2.08364800
C	-3.06033100	-3.11024300	3.70717500
H	-3.13424100	-3.40355600	4.74806200
C	-2.61868400	5.20040400	-1.76978100
H	-3.63900300	5.56167200	-1.70025200
C	-6.61392400	-0.52382100	-2.31660500
H	-7.56765000	-0.22268400	-2.73382400
C	-1.19801100	4.17624100	4.33007400
H	-1.40247500	4.48375400	5.34940500
C	-0.51609500	5.08163700	-2.93373400
H	0.11142800	5.34594700	-3.77775500
C	-0.88836900	5.12607800	3.35770400
H	-0.85215000	6.17845800	3.61708600
C	-1.24113600	2.82854500	3.97911500
H	-1.47557000	2.07872100	4.72683300
C	-1.82482700	5.55092200	-2.86261600

H	-2.22298500	6.18453800	-3.64713300
C	-3.58874600	-1.88381700	3.29381100
H	-4.07056600	-1.22819300	4.00980900
C	-3.48707700	-1.49353900	1.96335800
H	-3.87474300	-0.53726300	1.63504300
C	-6.56701500	-1.48024400	-1.30397200
H	-7.48140300	-1.92722700	-0.93197400
C	-5.34500200	-1.87080000	-0.75999000
H	-5.31111800	-2.61150400	0.02848200
C	-4.20346300	-0.33669100	-2.26194700
H	-3.28563400	0.10631900	-2.62673600
C	-5.43339100	0.04341100	-2.79395800
H	-5.46340300	0.78618700	-3.58233800
H	5.60122500	3.92681200	0.14279600
C	2.94674600	-0.42935500	-2.40799800
H	3.50967300	0.34303900	-2.93850500
H	3.20383500	-1.40564300	-2.82508200
H	1.88379200	-0.25560400	-2.58646200
Cl	1.29607200	-4.64998200	-0.01707600
Au	0.64625300	-2.44851500	-0.39462300

9c-carbene

Energy: -1959.341429 au

Sum of electronic and thermal Energies: -1958.704506 au

Geometry:

S	-3.04084400	1.20723600	-0.90617400
N	0.71409300	1.02114900	-0.56526500
C	4.87688100	1.26702400	-0.19774600
C	2.10580300	1.28742100	-0.51287200
C	-0.40696300	1.71935600	-0.96779900
C	4.12533500	0.14324600	0.17473900
H	4.61901200	-0.73473300	0.57724900
C	2.81378800	2.42683000	-0.89564100
C	0.46012900	-0.27181800	-0.08516300
C	2.75201000	0.16232300	0.02314400
C	2.09539000	-2.20797400	-0.44439400
C	2.14205900	3.66506500	-1.46800100
H	1.07301500	3.45215800	-1.52923800
C	4.20583600	2.37572900	-0.71640000
H	4.79101500	3.24471100	-1.00062200

C	6.38156900	1.25572300	-0.06475400
H	6.69436200	0.81503200	0.88628200
H	6.84260300	0.66443900	-0.86367700
C	1.73895200	-0.94963900	0.36335400
C	1.61395000	-1.23109300	1.87220600
C	-1.39622900	0.74686600	-0.69854100
C	-0.88123600	-0.48833300	-0.14212600
H	-1.41704600	-1.37855900	0.14523300
C	-3.80311000	1.35662900	0.75060200
C	2.10668600	-0.34405300	2.83203200
H	2.65691000	0.53390000	2.51769200
C	0.89953000	-2.35546000	2.30656500
H	0.51375800	-3.05615200	1.57489300
C	-3.88527800	-0.28165600	-1.51927500
C	3.04355400	-3.11540700	0.04347900
H	3.46845300	-2.96756600	1.02944300
C	1.54914200	-2.43043100	-1.71106600
H	0.81247600	-1.73847100	-2.10096700
C	-4.04775100	-0.37389800	-2.90052400
H	-3.72288300	0.43544700	-3.54428800

C	-4.62036200	-1.52304100	-3.44158500
H	-4.75270600	-1.60148800	-4.51432700
C	-5.02290300	-2.56417400	-2.60621600
H	-5.46781600	-3.45695900	-3.03012100
C	0.68679200	-2.58785800	3.66173700
H	0.13526700	-3.46743600	3.97621100
C	-4.96630800	1.70487100	3.23810200
H	-5.42180900	1.83970000	4.21239200
C	2.88376400	-4.42954500	-1.97795900
H	3.18589500	-5.28727000	-2.56853900
C	1.89465800	-0.57567900	4.19210700
H	2.28918000	0.12416300	4.92088600
C	3.43513700	-4.21570400	-0.71499200
H	4.16880700	-4.90846500	-0.31715400
C	1.93968400	-3.53185600	-2.47175300
H	1.50284400	-3.68690900	-3.45237000
C	1.18568700	-1.69733500	4.61377700
H	1.02556900	-1.87963200	5.67064700
C	-4.85388100	-2.45907200	-1.22581000
H	-5.16427900	-3.26892600	-0.57575400

C	-4.28389000	-1.31452300	-0.67312300
H	-4.15691200	-1.22598500	0.39833700
C	-5.74727500	1.80764300	2.08522200
H	-6.80673800	2.02348400	2.16138500
C	-5.16669300	1.63781600	0.83156500
H	-5.76960300	1.71287500	-0.06667100
C	-3.00834400	1.26510700	1.88694300
H	-1.94845000	1.06275900	1.79184400
C	-3.60275900	1.43716700	3.13775000
H	-2.99345800	1.36135100	4.03079400
H	6.79581900	2.26502000	-0.12050900
C	2.64521000	3.97123000	-2.88913000
H	3.71414000	4.20840900	-2.90019900
H	2.48357000	3.11742500	-3.55251700
H	2.10861300	4.82996400	-3.30427100
C	2.32617600	4.87610800	-0.53696800
H	3.38188300	5.14618800	-0.42847400
H	1.79665500	5.74671200	-0.93621200
H	1.92827900	4.66537400	0.45934000

[9c-[Au]Cl]

Energy: -2555.504625 au

Sum of electronic and thermal Energies: -2554.860318 au

Geometry:

S	2.57567000	-1.19774500	-0.60275500
N	-0.81176100	0.56347000	-0.24275500
C	-4.39994400	2.70606700	0.27228100
C	-1.91502400	1.47527300	-0.12570200
C	0.54829800	0.58565500	-0.50670100
C	-4.28610700	1.31874100	0.38440200
H	-5.15041500	0.71236900	0.63092000
C	-1.99518300	2.86797100	-0.28127300
C	-1.26960600	-0.74443800	-0.05296600
C	-3.05832100	0.71851500	0.17412100
C	-3.08270700	-1.30558800	1.63845600
C	-0.82803900	3.72350400	-0.73265400
H	0.06285700	3.34750700	-0.21939700
C	-3.25936600	3.43741400	-0.06658400
H	-3.35919300	4.51069600	-0.17051300
C	-5.71543200	3.39818800	0.53473100
H	-6.56134500	2.77000300	0.24420400

H	-5.82879500	3.62750200	1.60002100
C	-2.75035300	-0.78304300	0.22868200
C	-3.42967200	-1.59785800	-0.88792000
C	0.88333400	-0.78102500	-0.51663000
C	-0.23601300	-1.61634400	-0.20071500
H	-0.27607900	-2.69070900	-0.13884000
C	2.58959600	-2.92358200	-1.16777000
C	-3.98452300	-0.98710700	-2.01459200
H	-4.00748700	0.09301900	-2.08457700
C	-3.40561100	-2.99739300	-0.82895000
H	-2.98464200	-3.48793100	0.04126500
C	3.15568400	-1.30014200	1.11100900
C	-4.37532300	-1.74824200	1.94178100
H	-5.12242500	-1.79969900	1.15867500
C	-2.13413400	-1.26078900	2.66425600
H	-1.12973200	-0.91607200	2.44976500
C	4.11827500	-0.36464000	1.48211300
H	4.50903800	0.34720800	0.76395200
C	4.52477200	-0.32235100	2.81614300
H	5.26225100	0.40940400	3.12263200

C	3.96955200	-1.19840700	3.74523600
H	4.28530800	-1.15685000	4.78134000
C	-3.92683000	-3.76496100	-1.86585600
H	-3.90388400	-4.84718800	-1.79743900
C	2.72966500	-5.48130300	-2.23206100
H	2.78029100	-6.48264400	-2.64329700
C	-3.75582100	-2.09489900	4.25186600
H	-4.01484000	-2.40115200	5.25911000
C	-4.50822300	-1.75566400	-3.05521900
H	-4.93703500	-1.26174300	-3.92019400
C	-4.70955000	-2.14011700	3.23597600
H	-5.71579100	-2.48379600	3.44923200
C	-2.46739900	-1.65211400	3.96001300
H	-1.71720000	-1.60762100	4.74198400
C	-4.48326600	-3.14588700	-2.98592000
H	-4.89430300	-3.74207000	-3.79282900
C	2.99421000	-2.12055400	3.35473900
H	2.55501800	-2.79156500	4.08377900
C	2.57374500	-2.17222900	2.03060300
H	1.80488100	-2.86846700	1.71949800

C	3.60563000	-5.09931700	-1.21772100
H	4.34070100	-5.79937500	-0.83838900
C	3.54503300	-3.81457400	-0.68116600
H	4.22468000	-3.52114500	0.10835200
C	1.71733900	-3.28541900	-2.19491700
H	0.98507700	-2.58068900	-2.56759000
C	1.79006100	-4.57380500	-2.71900300
H	1.10753600	-4.86423600	-3.50891900
H	-5.78854300	4.34065400	-0.01299900
C	-0.94020100	5.20978100	-0.37541400
H	-1.71842300	5.71708500	-0.95437700
H	-1.15219600	5.35440800	0.68723800
H	0.00819600	5.70370800	-0.59966900
C	-0.59562100	3.54172900	-2.24477900
H	-1.45120500	3.92482600	-2.81037700
H	0.30187800	4.08225400	-2.55672600
H	-0.45413700	2.48972700	-2.50070200
Cl	3.83851700	3.39434500	-0.12925800
Au	1.99695200	1.99597600	-0.39766
